# The zinc finger transcription factor, KLF2, protects against COVID-19 associated endothelial dysfunction

**DOI:** 10.1038/s41392-021-00690-5

**Published:** 2021-07-12

**Authors:** Suowen Xu, Yujie Liu, Yu Ding, Sihui Luo, Xueying Zheng, Xiumei Wu, Zhenghong Liu, Iqra Ilyas, Suyu Chen, Shuxin Han, Peter J. Little, Mukesh K. Jain, Jianping Weng

**Affiliations:** 1 Institute of Endocrine and Metabolic Diseases, The First Affiliated Hospital of USTC, Division of Life Sciences and Medicine, University of Science and Technology of China, Hefei, China; 2 Department of Obstetrics and Gynecology, The First Affiliated Hospital of USTC, Division of Life Sciences and Medicine, University of Science and Technology of China, Hefei, Anhui P.R. China; 3 Department of Endocrinology and Metabolism, The Third Affiliated Hospital of Sun Yat-sen University, Guangzhou, China; 4 Anhui Province Key Laboratory of Hepatopancreatobiliary Surgery, The First Affiliated Hospital of USTC, Division of Life Sciences and Medicine, University of Science and Technology of China, Hefei, China; 5 Sunshine Coast Health Institute, University of the Sunshine Coast, Birtinya, QLD Australia; 6 School of Pharmacy, Pharmacy Australia Centre of Excellence, the University of Queensland, Woolloongabba, QLD Australia; 7 Department of Medicine, Case Cardiovascular Research Institute, Case Western Reserve University, Cleveland, OH USA; 8 Department of Medicine, Harrington Heart and Vascular Institute, University Hospitals Cleveland Medical Center, Cleveland, OH USA; 9 The First Affiliated Hospital, Bengbu Medical College, Bengbu, China

**Keywords:** Cardiology, Target identification

## Abstract

Coronavirus disease 2019 (COVID-19) is regarded as an endothelial disease (endothelialitis) with its patho-mechanism being incompletely understood. Emerging evidence has demonstrated that endothelial dysfunction precipitates COVID-19 and its accompanying multi-organ injuries. Thus, pharmacotherapies targeting endothelial dysfunction have potential to ameliorate COVID-19 and its cardiovascular complications. The objective of the present study is to evaluate whether kruppel-like factor 2 (KLF2), a master regulator of vascular homeostasis, represents a therapeutic target for COVID-19-induced endothelial dysfunction. Here, we demonstrate that the expression of KLF2 was reduced and monocyte adhesion was increased in endothelial cells treated with COVID-19 patient serum due to elevated levels of pro-adhesive molecules, ICAM1 and VCAM1. IL-1β and TNF-α, two cytokines elevated in cytokine release syndrome in COVID-19 patients, decreased KLF2 gene expression. Pharmacologic (atorvastatin and tannic acid) and genetic (adenoviral overexpression) approaches to augment KLF2 levels attenuated COVID-19-serum-induced increase in endothelial inflammation and monocyte adhesion. Next-generation RNA-sequencing data showed that atorvastatin treatment leads to a cardiovascular protective transcriptome associated with improved endothelial function (vasodilation, anti-inflammation, antioxidant status, anti-thrombosis/-coagulation, anti-fibrosis, and reduced angiogenesis). Finally, knockdown of KLF2 partially reversed the ameliorative effect of atorvastatin on COVID-19-serum-induced endothelial inflammation and monocyte adhesion. Collectively, the present study implicates loss of KLF2 as an important molecular event in the development of COVID-19-induced vascular disease and suggests that efforts to augment KLF2 levels may be therapeutically beneficial.

## Introduction

Coronavirus disease 2019 (COVID-19) is a severe, pernicious and highly infectious disease caused by a new type of coronavirus, SARS-CoV-2.^1^ This new coronavirus pandemic has had a great global public health impact and imposed tremendous economic burden worldwide. Limited pharmacotherapies are effective against COVID-19.

Cardiovascular complications have emerged as a new threat in COVID-19, indicating the necessity of assessing the long-term cardiovascular outcome of COVID-19 in infected patients.^2^ COVID-19 patients have a unique inflammatory profile with elevated levels of cytokines, chemokines/growth factors, and markers of hyperactivated endothelial cells (such as ICAM-1 and VCAM-1), which is correlated with the severity of COVID-19.^3^ In addition, it is observed that flow-mediated dilatation (FMD) in the brachial artery was reduced in COVID-19 patients, even after hospitalization for SARS-CoV-2 infection.^4^ A recent study has demonstrated that, in COVID-19 convalescents, the levels of circulating endothelial cells, a biomarker of vascular injury, was higher than that of healthy controls. This phenomenon was more pronounced in patients with pre-existing comorbidities (such as hypertension or diabetes).^5^ These evidences highlights the importance of monitoring cardiovascular complications of COVID-19 patients.

The vascular endothelium is not a static bystander, but a metabolically active paracrine, endocrine, and autocrine organ critical for the regulation of vascular tone and homoeostasis. Thus, involvement of the endothelium in the catastrophic clinical sequalae of COVID-19 represents the Achilles’ heel in COVID-19 patients.^6^ Since the discovery of viral inclusion structures and inflammatory cell infiltration as well as interaction with endothelium from various vascular beds from patient tissues,^7^ COVID-19 is considered as an endothelial disease,^8^ encompassing multiple aspects of endothelial dysfunction including oxidative stress, mitochondrial dysfunction, endothelial cell death, endothelial-to-mesenchymal transition (EndoMT), inflammation, glycocalyx disruption, and altered cell metabolism.^9^ After the initiation of the cascade of injurious responses triggered by a cytokine storm accompanying COVID-19, the vascular endothelium becomes dysfunctional, leading to an imbalance of tissue homeostasis and ensuing injury. However, the mechanisms underlying SARS-CoV-2-infection-induced dysfunction in the cardiovascular system, remain largely unknown. Further, elucidation of the driving mechanism(s) is important for developing effective targeted therapies.

In this study, we sought to identify the underlying mechanism of endothelial dysfunction in COVID-19 by treating human endothelial cells with serum from COVID-19 patients. We observed that the gene and protein expression of kruppel-like factor 2 (KLF2), a master regulator of vascular homeostasis, was decreased in endothelial cells treated with serum from COVID-19 patients. Furthermore, we show that genetic or pharmacological activation of KLF2 reverses multiple aspects of endothelial dysfunction. Our study offers a new target for therapeutic intervention of endothelial dysfunction in COVID-19.

## Results

### Demographic data from COVID-19 patients

We first collected serum samples from COVID-19 patients. A total of eight severe COVID-19 patients were used for collecting serum. Demographic data of patients are summarized in Supplementary Table S1. The age of the COVID-19 patients ranges from 55 to 93 years (median 69.75). The median values of white blood cell number and lymphocyte percentage is 8.23 × 10^9^/L and 15.75%, respectively. We also enrolled eight healthy volunteers. The age of the control subjects ranges from 48 to 58 years (median 50.625).

### COVID-19 patient-serum-treated human endothelial cells show KLF2 and eNOS downregulation, and endothelial inflammation

**Fig. 1:**
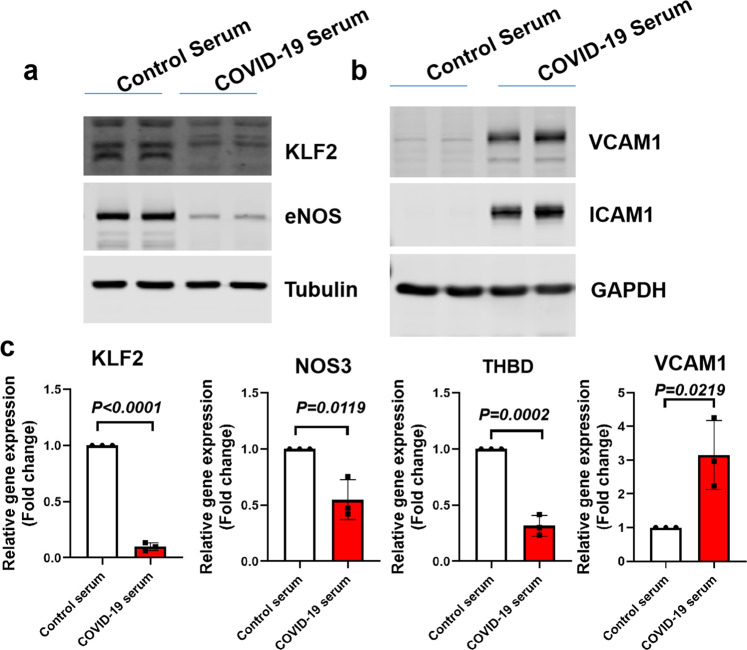
COVID-19 patient-serum-treated human endothelial cells show KLF2 downregulation, eNOS downregulation, and endothelial inflammation. **a** HUVECs were treated with control serum (20%) or COVID-19 serum (20%) for 24 h before protein was collected for western blot analysis of KLF2 and eNOS protein expression. *N* = 3. **b** HUVECs were treated with control serum (20%) or COVID-19 serum (20%) for 24 h before protein was collected for western blot analysis of ICAM1 and VCAM1 protein expression. *N* = 3. **c** HUVECs were treated with control serum (20%) or COVID-19 serum (20%) for 24 h before RNA was collected for the analysis of genes indicated. N = 3

As the release of multiple cytokines and chemokines is increased in COVID-19 patients, which may lead to multiple aspects of endothelial dysfunction, including hyperinflammation, coagulation, and thrombosis, we speculated that serum derived from COVID-19 patients could trigger endothelial dysfunction. To test the hypothesis that COVID-19 patients’ serum can cause endothelial dysfunction, we first treated HUVECs with control serum or patient serum for 24 h. Protein and RNA were collected for western blot and quantitative real-time PCR (qPCR) analysis, respectively. Our data demonstrate that patient serum treatment leads to decreased KLF2 and eNOS protein expression, while increasing ICAM1 and VCAM1 protein expression. qPCR analysis revealed that KLF2, eNOS (also known as NOS3), Thrombomodulin (THBD) were decreased by patient serum treatment, while VCAM1 gene expression was increased by patient serum treatment (Fig. 1a–c). The effect is specific to KLF2 as patient serum does not impact the expression of other 16 KLF family members (Supplementary Fig. 1). All these results suggest that patient serum causes the elevation of markers of endothelial inflammation, while decreasing the expression of KLF2, a critical master regulator of vascular homeostasis.

### KLF2 is downregulated by components of the cytokine storm -TNF-α and IL-1β

**Fig. 2:**
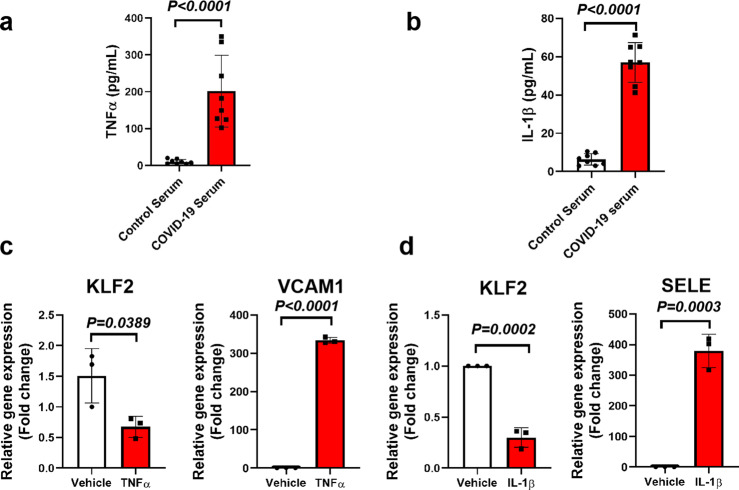
KLF2 is downregulated by components of cytokine storm- TNF-α and IL-1β. **a**, **b** Circulating levels of TNF-α and IL-1β were determined by ELISA. *N* = 8. **c** HUVECs were treated with vehicle (PBS) or TNF-α (10 ng/ml in PBS) for 6 h before RNA was collected for real-time PCR analysis of KLF2 gene expression using VCAM1 gene as the positive control. *N* = 3. **d** HUVECs were treated with vehicle (PBS) or IL-1β (10 ng/ml in PBS) for 6 h before RNA was collected for real-time PCR analysis of KLF2 gene expression using E-selectin (SELE) gene as the positive control. *N* = 3

COVID-19 is characterized as a cytokine release syndrome, in which heightened secretion of TNF-α, IL-1β, IL-6 and many other factors can trigger inflammation, local tissue damage as well as systemic multi-organ failure.^10^ We first evaluated the circulating levels of TNF-α and IL-1β in COVID-19 patients. Our data showed that, compared with control subjects, COVID-19 patients have elevated levels of TNF-α and IL-1β (Fig. 2a, b). We next evaluated whether TNF-α, and IL-1β, two cytokines observed in COVID-19 patients, can decrease KLF2 gene expression. Our data showed that both TNF-α, and IL-1β significantly decreased KLF2 gene expression (Fig. 2c, d). To demonstrate whether KLF2 plays an important role in COVID-19-induced endothelial dysfunction, we treated endothelial cells with KLF2 siRNA. Two independent siRNA against KLF2 reduced KLF2 gene expression by 50% (Supplementary Fig. 2). We next select KLF2 siRNA#1 to treat HUVECs. Our data indicate that KLF2 depletion by siRNA further aggravates COVID-19-patient-serum-induced monocyte adhesion to endothelial cell (Supplementary Fig. 3). These data indicate that existing TNF-α and IL-1β in patient serum is possible to augment endothelial inflammation via suppressing endogenous KLF2.

### Transcriptional profiling of human endothelial cells treated with atorvastatin in the presence of COVID-19 patient serum

**Fig. 3:**
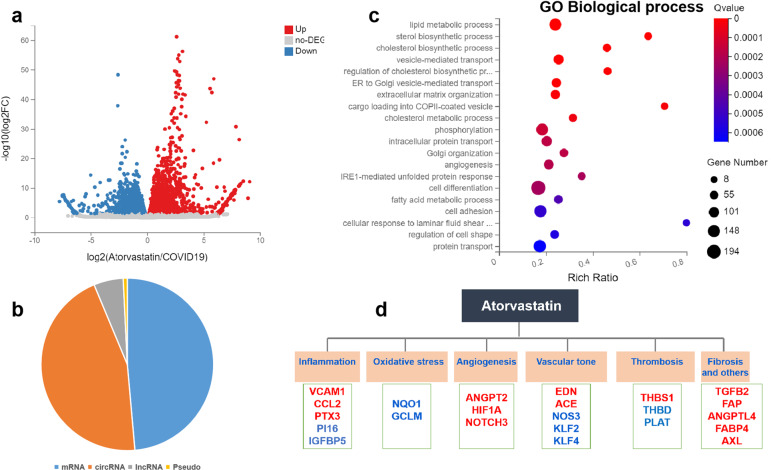
Transcriptional profiling of human endothelial cells treated with atorvastatin in the presence of patient serum. **a** Volcano plot of atorvastatin treated HUVECs exposed to COVID-19 patient serum. Four different donors of HUVECs were treated with vehicle (0.1% DMSO) or Atorvastatin (10 µM in 0.1%DMSO) for 24 h in the presence of COVID-19 patient serum before RNA was collected for next-generation RNA-sequencing (RNA-seq). **b** Categorization of differentially expressed transcripts in HUVECs treated as described in **a**. **c** Gene ontology (GO) analysis of changes in biological process. **d** Summary of differentially expressed genes in response to atorvastatin treatment in the presence of patient serum. Red, downregulated genes; blue, upregulated genes

Since statins have reported benefits in cardiovascular outcome in COVID-19 patients^11,12^ and statins are potent pharmacological activators of KLF2,^13,14^ we postulated that KLF2 activation by statins could counteract COVID-19-associated endothelial dysfunction. We thus performed RNA-sequencing in HUVECs treated with atorvastatin in the presence of patient serum. Our data showed that atorvastatin treatment leads to an overall protective transcriptomic profile (antioxidant, anti-inflammatory, vasodilatory, anti-fibrotic, anti-angiogenesis, and anti-thrombotic), which may underscore the potential benefits of statins in COVID-19 patients (Fig. 3a and Supplementary Table 2). Treatment with atorvastatin leads to altered expression of different types of transcripts, the majority of which are mRNAs and circular RNAs (Fig. 3b). Gene ontology (GO)-biological process analysis revealed that the top process is a lipid metabolism (Fig. 3c). Detailed analysis of the transcriptional profile associated with atorvastatin treatment revealed that atorvastatin treatment reduces the expression of markers of inflammation (VCAM1, CCL2, and PTX3), angiogenesis (ANGPT2, HIF1α, and NOTCH3), thrombosis and vasoconstriction (EDN1, ACE, and THBS1), and fibrosis (TGFB2 and FAP). In contrast, atorvastatin treatment also leads to the upregulated expression of genes associated with anti-inflammation (PI16 and IGFBP5), antioxidant (NQO1 and GCLM), anti-thrombotic effects (THBD and PLAT) and vascular homeostasis (KLF2, KLF4, and NOS3). Atorvastatin treatment also leads to decreased gene expression of ANGPTL4, FABP4, and AXL (Fig. 3d). Taken together, the endothelial protective transcriptome could partially explain the clinical benefits of statins in COVID-19 patients.

### Atorvastatin regulates the expression of genes relevant to endothelial dysfunction in human endothelial cells exposed to COVID-19 patient serum

**Fig. 4:**
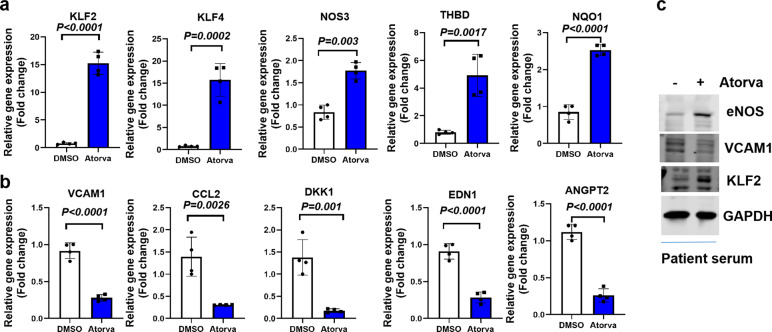
Atorvastatin regulates the expression of genes and proteins relevant to endothelial dysfunction in endothelial cells exposed to patient serum. **a** HUVECs were treated with vehicle (0.1% DMSO) or Atorvastatin (10 µM in 0.1%DMSO) for 24 h in the presence of COVID-19 patient serum before RNA was collected for real-time PCR analysis of gene expression. Genes related to vascular homeostasis and anti-thrombosis (KLF2, KLF4, NOS3, and Thbd) and antioxidant status (NQO1) were presented as fold changes over control. *N* = 4. **b** HUVECs were treated as described in **a**, and expression of genes related to inflammation (VCAM1, CCL2, and DKK1), vascular tone (EDN1 or ET1), and angiogenesis (ANGPT2) were presented as fold changes over control. *N* = 4. **c** HUVECs were treated as described in **a** before whole-cell lysate was collected for western blot to determne protein expression of eNOS, VCAM1, and KLF2 using GAPDH as the loading control. *N* = 3

We further performed qPCR to validate our RNA-sequencing data. Our qPCR data showed that atorvastatin treatment leads to the upregulation of vascular homeostasis related genes (KLF2, KLF4, NOS3, and THBD) and antioxidant genes (NQO1). However, atorvastatin treatment reduced the expression of pro-inflammatory (VCAM1, CCL2, and DKK1), vasoconstrictive (EDN1) and pro-angiogenic (ANGPT2) targets (Fig. 4a, b). These data are of translational relevance as increased pulmonary vascular endothelialitis, thrombosis, and angiogenesis are observed in COVID-19 patients.^15,16^ In addition, atorvastatin reduces the expression of ANGPT2, which emerges as a biomarker of endothelial activation in COVID-19 and predicts COVID-19 patients to be admitted to ICU.^17^ Analysis of protein expression revealed that atorvastatin treatment leads to increased protein expression of KLF2 and eNOS, while decreases the expression of VCAM1 (Fig. 4c). These data collectively provide new mechanistic insights into statin-mediated protective effects against COVID-19-induced endothelial dysfunction.

### KLF2 overexpression modulates the expression of genes relevant to endothelial dysfunction in human endothelial cells exposed to COVID-19 patient serum

**Fig. 5:**
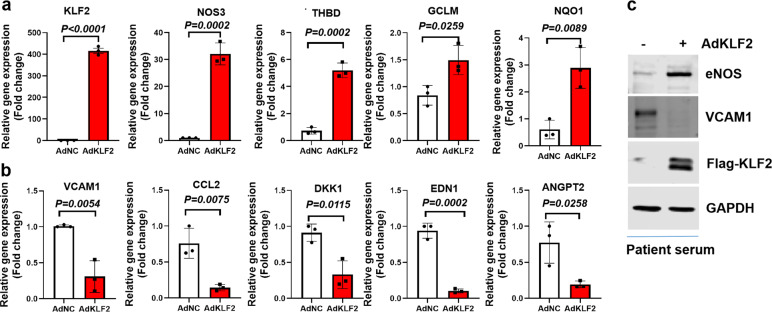
KLF2 overexpression modulates the expression of genes and proteins relevant to endothelial dysfunction in endothelial cells exposed to patient serum. **a** HUVECs were treated with control adenovirus (AdNC) or KLF2 overexpressing adenovirus (Ad-KLF2) for 24 h in the presence of COVID-19 patient serum before RNA was collected for real-time PCR analysis of gene expression. Genes related to vascular homeostasis and anti-thrombosis (KLF2, NOS3, and THBD) and antioxidant status (GCLM and NQO1) were presented as fold changes over control. *N* = 3. **b** HUVECs were treated as described in **a**, and expression of genes related to inflammation (VCAM1, CCL2, and DKK1), vascular tone (EDN1 or ET1), and angiogenesis (Angpt2) were presented as fold changes over control. *N* = 3. **c** HUVECs were treated as described in **a** before whole-cell lysate was collected for western blot to determne protein expression of eNOS, VCAM1, and flag-tagged KLF2 using GAPDH as the loading control. *N* = 3

Since atorvastatin is a pharmacological activator of KLF2 via the mevalonate^13^ and MEF2 (myocyte enhancer factor 2)-dependent pathways,^14^ we next asked whether overexpression of KLF2 via an adenoviral vector can also reverse patient-serum-induced endothelial dysfunction. Our qPCR data showed that adenoviral overexpression of KLF2 leads to the upregulation of vascular homeostasis related genes (KLF2, NOS3, and THBD) and antioxidant genes (GCLM and NQO1). Conversely, adenoviral overexpression of KLF2 reduces the expression of pro-inflammatory (VCAM1, CCL2, and DKK1), vasoconstrictive (EDN1) and pro-angiogenic (ANGPT2) genes (Fig. 5a, b). Analysis of protein expression revealed that KLF2 overexpression leads to increased protein expression of KLF2 and eNOS, while decreases the expression of VCAM1 (Fig. 5c). These data indicate that genetic activation of KLF2 could confer vascular homeostatic functions.

### Genetic and pharmacological activation of KLF2-reduced monocyte adhesion to COVID-19-patient-serum-treated endothelial cells

**Fig. 6:**
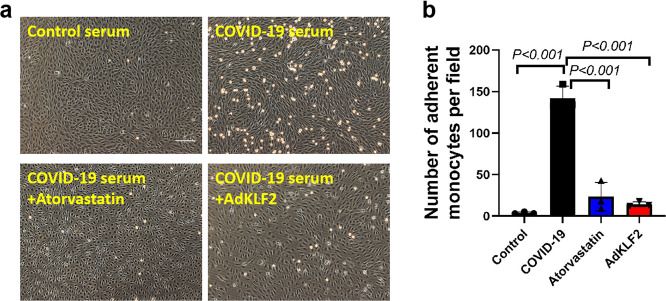
Genetic and pharmacological activation of KLF2-reduced monocyte adhesion to patient-serum-treated endothelial cells. **a** Human pulmonary microvascular endothelial cells (HPMECs) were treated with control serum or COVID-19 serum for 24 h before THP-1 monocyte adhesion assay was performed. Nonadherent cells were washed and photographs of adherent monocytes were taken, *N* = 3. Scale bar = 50 µm. **b** A quantification of adherent monocytes as described in **a**, *N* = 3

A recent study has shown significant accumulation of inflammatory cells associated with the endothelium, as well as apoptotic bodies in COVID-19 patients.^7^ In particular, the myocardial arterioles and venules of the COVID-19 patients showed mild to moderate inflammatory infiltrates rich in lympho-monocytic cells.^18^ However, how these lympho-monocytic cells infiltration in affected tissues/organs was increased in COVID-19 patients is unknown. It has been well established that genetic and pharmacological activation of KLF2 reduces monocyte adhesion to activated endothelium.^19,20^ We thus investigated the role of KLF2 overexpression and atorvastatin on COVID-19-patient-serum-induced monocyte adhesion. Our data demonstrate that both KLF2 overexpression (by KLF2 adenovirus) and activation (by Atorvastatin) significantly reduces monocyte adhesion (Fig. 6). However, treatment of HUVECs with an unrelated adenovirus (encoding GFP) or unrelated drug (sorafenib) does not ameliorate monocyte adhesion, suggesting that the preventive effect of KLF2 activation was specific (Supplementary Fig. 4). In addition, we have previously demonstrated that tannic acid, a naturally occurring KLF2 activator, inhibited TNFα-induced monocyte adhesion to endothelial cells via the ERK5/MEF2 pathway.^21^ We next explored the effect of tannic acid on monocyte adhesion, and we observed the pharmacological activation of KLF2 by tannic acid elicits similar protective effects against monocyte adhesion induced by patient serum (Supplementary Fig. 4). These data suggest that pharmacological activation of KLF2 attenuates COVID-19-patient-serum-induced monocyte adhesion to activated endothelial cells, thus providing a proof-of-concept that KLF2 activation has therapeutic potential in limiting endothelial dysfunction in COVID-19.

### Knockdown of KLF2 attenuates the protective effects of statins

Since atorvastatin is a pleiotropic cardiovascular drug that has endothelial protective actions dependent on KLF2 and others factors, we explored whether the protective effects can be reversed by KLF2 depletion. Our data indicate that KLF2 depletion by KLF2 siRNA partially reverses atorvastatin mediated protective effects against COVID-19-serum-induced monocyte adhesion (Supplementary Fig. 3). This piece of data lends additional support to the fact that the benefits of statins are partially exerted through KLF2 activation.

## Discussion

Emerging histopathological evidence from COVID-19 patients has underscored the pivotal role of endothelial cell dysfunction, thrombosis/coagulation, and systemic inflammation in COVID-19 caused by SARS-CoV-2 infection.^7,22^ Consecutive inflammatory/immune cell recruitment and endothelial dysfunction could explain microcirculation failure and systemic endothelialitis observed in various vascular beds.^18,23^ Mechanistically, elevated levels of TNF-α, interferons, IL-1**β**, IL-6, chemokines (CCL2 and MCP1), and PAI-1 in cytokine storm,^24^ could relay the deleterious cascade of endothelial dysfunction, consisting of vasoconstriction, cell injury/death, hyperpermeability, and leukocyte recruitment in the microvasculature. Therapeutic strategies targeting endothelial inflammatory responses and dysfunction could restore the quiescent endothelium and improve multi-organ endothelialitis and injury.^25^

Our data confirm and expand previous evidence on the standing of endothelial dysfunction in COVID-19 patients. Specifically, we addressed: (1) COVID-19 patient serum downregulated KLF2 expression in human endothelial cells; (2) High level of TNFα and IL-1β in COVID-19 patient serum could partially explain the mechanism of KLF2 downregulation; (3) KLF2 downregulation-induced monocyte adhesion could explain endothelialitis and lympho-monocytic cells infiltration in COVID-19 patients; (4) genetic and pharmacological activation of KLF2 by KLF2 adenovirus, atorvastatin, and tannic acid ameliorates patient-serum-induced monocyte adhesion. Our study provides the proof-of-concept that KLF2 activation could be a potential strategy to ameliorate COVID-19-associated endothelial dysfunction and endothelialitis.

A landmark histopathological study has shown direct SARS-CoV-2 viral infection of endothelial cells evidenced by the presence of viral elements within endothelial cells and the accumulation of inflammatory cells.^7^ These findings suggest that SARS-CoV-2 infection facilitates endothelialitis in several organs. Since this discovery,^7^ the importance of endothelial activation and dysfunction in COVID-19 has been intensively pursued.^8,18,23,26^ More recently, increased markers of vascular inflammation,^27^ oxidative stress,^28^ EndoMT,^29^ coagulation (D-dimer and plasminogen activator inhibitor-1),^15,30^ immunothrombosis,^31^ angiogenesis,^15^ altered endothelial cell metabolism (glycolysis),^32^ glycocalyx disruption^33^ have been observed in patients with COVID-19 or SARS-CoV-2 spike protein treated endothelial cells (reviewed in ref. ^9^). COVID-19 is recently reported to be associated with elevated markers of inflammation, coagulation and endotheliopathy in the liver endothelium driven by IL-6 trans-signaling, which represents a new mechanism of liver injury caused by SARS-CoV-2 infection.^34^ However, the precise mechanism underlying these features of endothelial dysfunction and COVID-19 is largely unknown. Studies focusing on endothelial dysfunction in COVID-19 patients are warranted as to decipher the precise role of the endothelium in severe SARS-CoV-2 infection and multi-organ dysfunction and to identify targets for future interventions.^35^

In a recent study, the spike (S) protein of SARS-CoV-2 alone can damage endothelial cells in an AMPK-dependent manner, evidenced by mitochondrial dysfunction, reduced ACE2 expression and eNOS expression and NO bioavailability, and skewed endothelial cell metabolism towards increased glycolysis.^32^ In addition, chloroquine, a disputed anti-COVID-19 medication, may induce endothelial injury/cytotoxicity through lysosomal dysfunction and oxidative stress, the effect of which can be ameliorated by treatment with lysosomal enzyme α-galactosidase A. This line of evidence suggests that endothelial cell injury may contribute to the failure of chloroquine as effective therapy for COVID-19 partially due to lysosomal dysfunction elicited by chloroquine.^36^ All these lines of evidence indicate that the endothelium is a *bona fide* key target for devising and developing anti-COVID-19 therapeutic agents.

However, there are no effective pharmacotherapies available which specifically target the diseased vascular endothelium in affected organs from COVID-19 patients. Several clinical trials are currently underway to explore this concept. Anti-inflammatory therapies, such as colchicine and tocilizumab (an anti-IL-6 receptor monoclonal antibody), are being evaluated for clinical utility in COVID-19 patients (www.clinicaltrial.gov). In our study, we observed that COVID-19 patient-serum-treated human endothelial cells show features of endothelial dysfunction, including increased monocyte adhesion to activated endothelium, concurrent with decreased expression of vasoprotective molecule KLF2 and eNOS, and increased expression of ICAM1, and VCAM1; these observations raise the possibility that KLF2 serves as a new and promising target for therapeutic intervention of endothelial dysfunction accompanying COVID-19 (Supplementary Fig. 5).

Previous studies have shown that KLF2 is a negative regulator of endothelial activation, dysfunction, and thrombosis.^37–39^ KLF2 expression was downregulated by treatment with various inflammatory cytokines, such as TNF-α^40^ and IL-1β,^20^ two of which are frequently observed cytokine storm in COVID-19 patients.^10^ A recent study from Fang laboratory demonstrated reduced endothelial KLF2 expression in lung autopsies of COVID-19 patients, compared with control subjects. This study provides the first evidence supporting SARS-CoV-2-infection-associated KLF2 downregulation.^41^ In this study, we documented elevated circulating levels of TNF-α and IL-1β in serum from COVID-19 patients, compared with control subjects. We also observed that the expression of KLF2 was downregulated by both cytokines, TNF-α and IL-1β, and further demonstrated that forced overexpression of KLF2 suppressed patient-serum-induced inflammatory gene and protein expression and monocyte adhesion to activated endothelial cells in vitro. In addition, KLF2 overexpression reversed COVID-19-patient-serum-induced eNOS gene and protein downregulation, indicating that SARS-CoV2-infection-induced cytokine storm disrupts endothelial homeostasis. Therefore, based on our data and published literature,^41^ it is plausible that KLF2 suppresses COVID-19-induced endothelial dysfunction by multiple mechanisms, which may involve: (1) enhancing endothelial quiescence and pulmonary vascular integrity; (2) boosting eNOS dependent NO production; (3) inhibiting ICAM-1, VCAM-1, and E-selectin mediated monocyte adhesion via reported suppressing NF-kB signaling pathway; (4) hemostatic and anti-thrombotic function mediated by thrombomodulin upregulation and PAI-1 downregulation. In our study, we observed that KLF2 was downregulated by COVID-19 patient serum, which provides a novel mechanism of KLF2 in suppressing endothelial dysfunction in COVID-19. We also observed that adenovirus mediated KLF2 overexpression or atorvastatin reversed COVID-19-patient-serum-induced monocyte adhesion to endothelial cells, suggesting that pharmacological activation of KLF2 could be a viable strategy of ameliorating endothelial dysfunction in COVID-19.

In addition to statins, several drugs or compounds, such as resveratrol (a wine-derived phytochemical),^42^ metformin^43^ and liraglutide^44^ exert cardiovascular protective actions via KLF2 upregulation. By virtue of the anti-thrombotic and anti-inflammatory properties mediated by KLF2 activation, these drugs/compounds have the potential to lower COVID-19-associated endothelial dysfunction and mortality.

The use of statins was associated with reduced risk for 28-day all-cause mortality, a lower risk of developing severe COVID-19, and faster recovery time.^11,12^ However, the protective mechanism of statins in COVID-19 is unclear. We found that statins may be effective by ameliorating endothelial dysfunction triggered by cytokine storm cytokines/chemokines (IL-6, TNF-α, IL-1β, CCL2, interferons, and related factors) released from COVID-19 patients. The possible reasons for statin-mediated suppressive effects on endothelial dysfunction are partially due to increased KLF2 expression and regulation of the expression of its downstream genes, which include NF-kB-dependent pro-inflammatory genes (VCAM-1, SELE and CCL2), thrombotic genes (THBD and THBS1) and vascular homeostasis-associated genes (eNOS and ET1). Since statins are pleiotropic drugs with both lipid-lowering and cholesterol-lowering independent effects, both of which may be accountable for the pharmacological effects of statins in COVID-19 patients. In our study, we demonstrate that depletion of KLF2 partially reverses atorvastatin mediated protective effects against monocyte adhesion. This evidence, together with the observation that KLF2 activation by another activator, tannic acid, also leads to reduced monocyte adhesion, convergently suggests that KLF2 activation may be therapeutically beneficial. Classically, SARS-CoV-2 uses the ACE2 receptor to achieve entry into host cells. However, the endothelium of the major coronary arteries of COVID-19-positive patients was devoid of ACE2 receptor expression,^18^ raising the existence of alternative receptors in endothelial cells. In our study, we observed that atorvastatin treatment leads to marked decreases in the expression of AXL, a new candidate receptor, which binds to SARS-CoV-2 spike glycoprotein and facilitates SARS-CoV-2 entry into host cells with low levels of ACE2 expression^45^ (such as the coronary artery endothelial cells). Therefore, the effect of statins on SARS-CoV-2 entry into host endothelial cells is also possible.

We recognize that the present study has the following limitations. First, in light of the presence of SARS-CoV-2 viral inclusion elements within endothelial cells,^7^ the transcriptomic profile of SARS-CoV-2 infected human microvascular endothelial cells from different vascular beds are warranted; Secondly, due to inaccessibility of postmortem tissues from COVID-19 patients, further detection of KLF2 expression in vascular endothelium from vascular beds from small vessels (such as cardiac capillaries, arterioles, and venules) and the possibility whether KLF2 expression negatively correlates with increased vascular inflammation, the severity of COVID-19, inflammatory biomarkers and coagulopathy remains to be evaluated. Thirdly, the present study only involves experiments in cultured endothelial cells. Further experiments determining the role of KLF2 in COVID-19-induced endothelial dysfunction in vivo is warranted. Fourthly, age is an independent risk factor for endothelial dysfunction and COVID-19. The age of control subjects is much lower than COVID-19 patients, which can be a study limitation. Last but not least, although endothelial dysfunction is a potential contributing factor to COVID-19, the direct relationship between endothelial dysfunction and the pathogenesis of COVID-19 is lacking in light of the fact that limited tests are available for evaluating endothelial function in clinical practice.^46^ Further work is needed to assess whether the findings in this report are relevant in a clinical setting.

In conclusion, the present study uncovers KLF2 downregulation as an important mechanism driving COVID-19-induced endothelial dysfunction as well as underscoring the importance of examining endothelial function in COVID-19 patients. From a translational perspective, our study suggests that genetic and pharmacological activation of KLF2 may represent a promising therapeutic strategy to ameliorate COVID-19-associated endothelial dysfunction, pinpointing a new direction to treat endothelialitis accompanying the devastating pandemic of COVID-19.

## Materials and methods

### Patient demographic data

Serum samples were collected from eight confirmed COVID-19 patients as described in our previous cohort.^47^ All patients were diagnosed with laboratory-confirmed COVID-19 infection and discharged after meeting the National Recovery Standard of COVID-19 stipulated by the National Health Committee of China. The severity of patients was defined according to National Guidelines for the Diagnosis and Treatment of COVID-19.^48^ Clinical demographic information and laboratory testing data (Supplementary Table 1), including age, sex, comorbidities, smoking history, treatment, complete blood counts, blood biochemistry were collected at the time of admission and throughout the course of the study. A subfraction of patients were followed up for various blood tests. Control sera were collected from normal human subjects. This study was conducted under a clinical protocol approved by the Institutional Review Board (IRB) of First Affiliated Hospital of University of Science and Technology of China (protocol number: 2020-XG(H)-009). All participants agreed to participate in the study and signed informed consents approved by the IRB.

### Drug and adenovirus

Atorvastatin was purchased from Cayman Chemicals (Ann Arbor, MI). Tannic acid and sorafenib was purchased from TargetMol (Shanghai, China). Control adenovirus, GFP adenovirus (Ad-GFP), and KLF2 overexpression adenovirus (Ad-KLF2) with a C-terminal Flag/His tag was custom made at Weizhen Biosciences Inc. (Jinan, Shandong, China).

### Cell culture

HUVECs were isolated from the umbilical cords of normal pregnant women according to our published protocols with patients’ informed consent.^49^ Umbilical cords were collected under a clinical protocol approved by IRB of First Affiliated Hospital of University of Science and Technology of China (protocol number: 2020-ky013). Three to four different donors of HUVECs were used in this study unless specified otherwise. HUVECs were cultured in ECM media supplemented with 1×endothelial cell growth supplement (ScienCell, Carlsbad, CA), 1×penicillin/streptomycin antibiotic, and 5% FBS. Cells at passage number of 3–8 were used in this study. Human pulmonary microvascular endothelial cells (HPMECs) were purchased from ScienCell (Carlsbad, CA) under the identical culture conditions to HUVECs. Endothelial cells were authenticated by staining with endothelial cell marker proteins-CD31 and VE-cadherin as well as DiI-oxLDL uptake.

### RNA interference (RNAi) in human endothelial cells

HUVECs at subconfluence was transfected with control siRNA (siNC, 100 nM) or two independent KLF2 siRNAs (siKLF2, 100 nM) using Lipofectamine 2000 (ThermoFisher, Waltham, MA). siNC (#siN0000001-1-5) and siKLF2#1 (#stB0009473A) and siKLF2#2 (#stB0009473C) were purchased from RiboBio Co., Ltd (Guangzhou, China). Forty eight hours after transfection, RNA was collected to validate the efficiency of gene silencing.

### Real-time quantitative PCR (qRT-PCR)

Total RNA was extracted from cultured human ECs using a RNeasy Mini kit (Qiagen, Hilden, Germany). For reverse transcription, total RNA was converted into first strand complementary DNA (cDNA) using a Reverse Transcription Kit from Takara (Dalian, China) or a HiScript III RT SuperMix (Vazyme, Nanjing, China) following the manufacturer’s instructions. Quantitative real-time PCR was then performed with a Roche LC96 Real-Time PCR Detection System, using SYBR Green Supermix (Roche, Basel, Switzerland) or ChamQ SYBR qPCR Master Mix (Vazyme, Nanjing, China) for relative mRNA quantification using β-actin or GAPDH as loading control. The sequences of all the primers used were listed in Supplementary Table 3.

### Western blot analysis

Whole-cell lysates were prepared from cultured cells. For western blots,^49^ total cell lysates (15–20 μg) were separated by SDS-PAGE, transferred to nitrocellulose membrane (Pall, East Hills, NY) and were subsequently blocked in LI-COR blocking buffer (LI-COR Biosciences, Lincoln, NE) at room temperature for 1 h. Then, the blots were incubated overnight at 4 °C with appropriate primary antibodies listed in Supplementary Table 4. After being washed three times with 1 × Tris buffered saline with 0.1% Tween-20 (TBST), membranes were incubated with IRDye® 680RD Goat anti-Mouse IgG (H + L) or IRDye® 800CW Goat anti-Rabbit IgG (H + L) (1:10,000 dilution in 1XTBST; LI-COR) at room temperature for 30 min. Images were visualized by using an LI-COR-CLx Infrared Imaging System (LI-COR).

### ELISA

Serum TNFα and IL-1β levels in control subjects and human patients were determined using human TNFα sandwich ELISA kit (#KE00068) and IL-1β ELISA kit (#KE00021) (ProteinTech, Rosemont, IL). In brief, samples were added and incubated with biotin-labelled antibody against IL-1β and TNFα. After that, streptoavidin-HRP was added for an additional incubation for 1 h. TMB substrate was added and incubated for 40 min before absorbance mesaurement at 450 nm with corrected wavelength at 630 nm using a microplate reader (Molecular Devices, Model iD3). Protein concentration of IL-1β and TNFα was calculated using standard curves.

### Assay of monocyte adhesion to endothelial cells

Human THP-1 monocyte adhesion assay was performed as we previously described.^49,50^ In brief, HUVECs were incubated with 20% patient serum (combined from two patients with 1:1 ratio) or control serum for 24 h before addition of THP-1 monocytic cells (1.5 × 10^4^) for 30 min. Nonadherent cells were washed and images were taken. Three images at different optic fields were taken for assessing the average number of adherent monocytes.

### Next-generation RNA-sequencing

HUVECs were treated with atorvastatin (10 µM) before treatment with COVID-19 patient serum for 24 h. After that, RNA was extracted using the RNeasy Mini-Kit (Qiagen). The sequencing data were filtered with SOAPnuke (v1.5.2) by (1) Removing reads containing sequencing adapter; (2) Removing reads whose low-quality base ratio (base quality less than or equal to 5) is more than 20%; (3) Removing reads whose unknown base (‘N’ base) ratio is more than 5%, afterwards clean reads were obtained and stored in FASTQ format. The clean reads were mapped to the reference genome using HISAT2 (v2.0.4). Bowtie2 (v2.2.5) was applied to align the clean reads to the reference coding gene set, then expression level of gene was calculated by RSEM (v1.2.12). The heatmap was drawn by pheatmap (v1.0.8) according to the gene expression in different samples. Essentially, differential expression analysis was performed using the DESeq2 (v1.4.5) with Q value ≤ 0.05. To take insight to the change of phenotype, GO (http://www.geneontology.org/) and KEGG (https://www.kegg.jp/) enrichment analysis of annotated different expressed gene was performed by Phyper (https://en.wikipedia.org/wiki/Hypergeometric_distribution) based on Hypergeometric test. The significant levels of terms and pathways were corrected by Q value with a rigorous threshold (Q value ≤ 0.05) by Bonferroni.

### Statistical analysis

Data are presented as means ± SD unless otherwise indicated. Statistical analysis was performed using GraphPad Prism Software Version 8.3 (GraphPad software, La Jolla, CA). Results were evaluated by *t*-test or by one-way analysis of variance (ANOVA) when appropriate. When multiple comparisons were made, a Bonferroni correction was performed for each test. A *P*-value less than 0.05 was considered to be statistically significant.

## Supplementary information

Supplemental materials

Supplemental Table 2

## Data Availability

The datasets generated during and/or analyzed in the current study are available from the corresponding author on reasonable request.
