# Supplementary material for: The zinc finger transcription factor, KLF2, protects against COVID-19 associated endothelial dysfunction
Source: Signal Transduct Target Ther. 2021 Jul 12;6:266. doi: 10.1038/s41392-021-00690-5 (PMC8273371; doi:10.1038/s41392-021-00690-5)

Supplementary Materials for

The zinc finger transcription factor, KLF2, protects against

COVID-19 associated endothelial dysfunction

Suowen Xu^1,#^ [
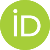
](https://orcid.org/0000-0002-5488-5217), Yujie Liu^2,#^, Yu Ding^1^, Sihui Luo^1^, Xueying Zheng^1^, Xiumei Wu^3^, Zhenghong Liu^1^, Iqra Ilyas^1^, Suyu Chen^3^, Shuxin Han^4^, Peter J. Little^5,6^, Mukesh K Jain^7,8^,

Jianping Weng^1^*[
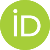
](https://orcid.org/0000-0002-5488-5217)

Correspondence to: wengjp@ustc.edu.cn

**This PDF file includes:**

Figures. S1 to S5

Tables S1 to S4

Uncropped full gel scan

Supplemental Figures


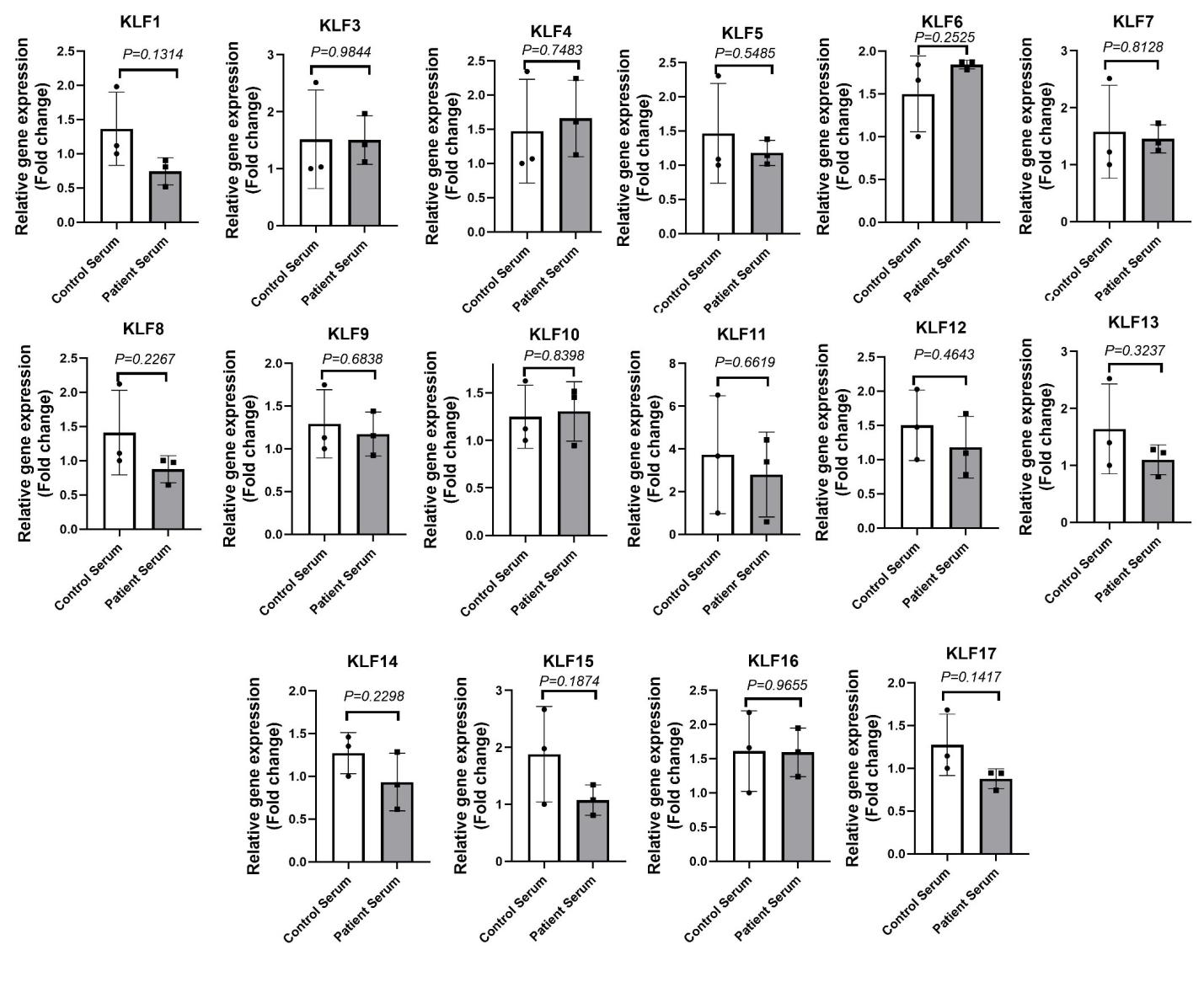


**Supplementary Fig. 1**. The effect of COVID-19 patient serum on expression of other KLF family members. HUVECs were treated with control serum (20%) or COVID-19 serum (20%) for 24 h before RNA was collected for the analysis of genes indicated.


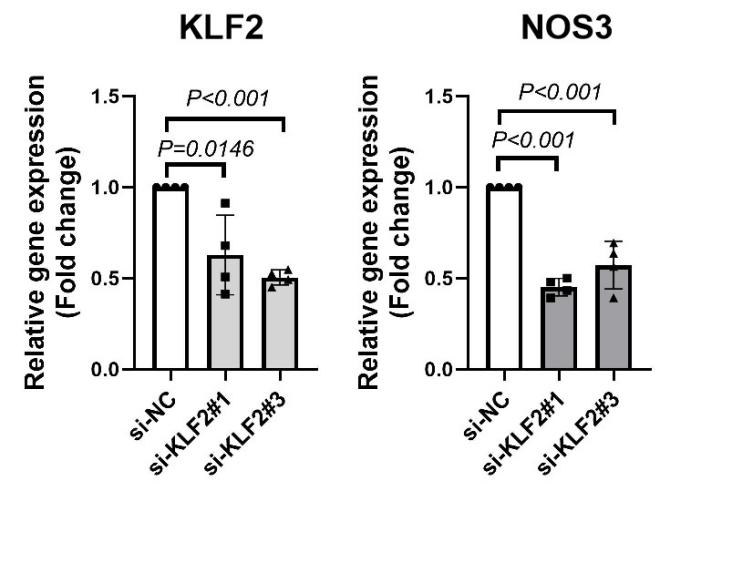


**Supplementary Fig. 2**. Silencing efficiency of KLF2 siRNA

HUVECs were treated with control siRNA (si-NC, 100 nM) or KLF2 siRNAs (si-KLF2, #1, and #3, 100 nM) for 48 h before RNA was collected for real-time PCR to detect gene expression of KLF2 and NOS3，**P<0.01, ***P<0.001 vs si-NC, N=4.


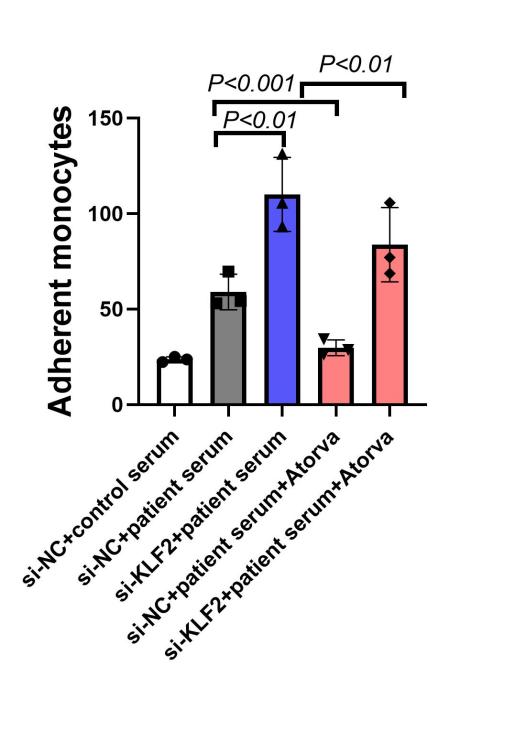


**Supplementary Fig. 3.** KLF2 deficiency aggravates monocyte adhesion to endothelial cells and reverses atorvastatin mediated protective effects.

HUVECs were treated with control siRNA (si-NC, 100 nM) or KLF2 siRNAs (si-KLF2, 100 nM) for 48 h with or without atorvastatin in the presence of control serum or patient serum. Then, monocyte adhesion assay was performed. N=3.


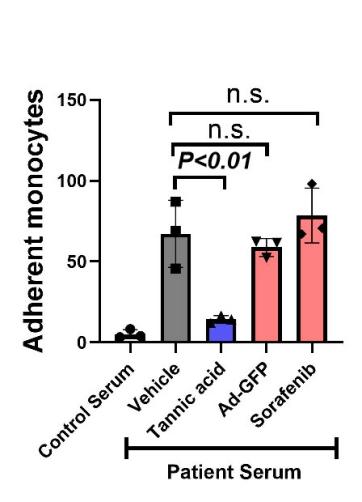


**Supplementary Fig. 4.** Tannic acid attenuates, but Ad-GFP or sorafenib does not affect patient serum induced monocyte adhesion to endothelial cells.

HUVECs were treated with KLF2 activator tannic acid, an irrelevant adenovirus (Ad-GFP) or an irrelevant drug sorafenib before monocyte adhesion assay was performed. n.s. denotes non-significant difference. N=3.


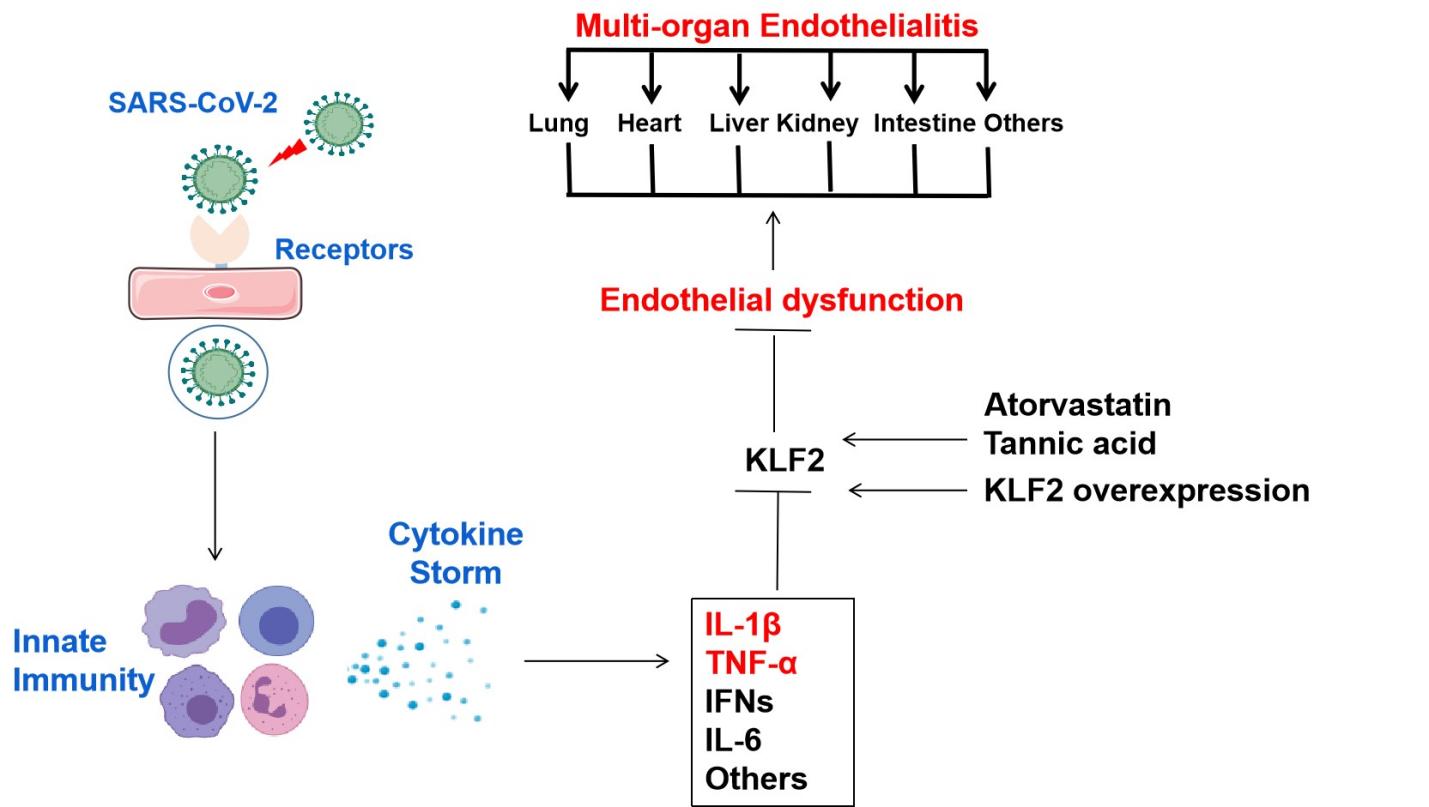


**Supplementary Fig. 5.**

# Endothelial dysfunction contributes to COVID-19-associated multi-organ endothelialitis: potential role of KLF2

The role of endothelial dysfunction in COVID-19 has gained intensive research interest. Growing evidence suggests that the angiotensin converting enzyme 2 receptor (ACE2 receptor) is expressed on endothelial cells (ECs) from small vessels (such as capillaries) in the lung, heart, kidney, and intestine. Upon viral infection of ECs by severe acute respiratory syndrome coronarvirus 2 (SARS-CoV-2), ECs become activated and dysfunctional. SARS-CoV-2 infection in endothelial cells is believed to trigger a cytokine storm that plays a critical role in the pathogenesis of endothelialitis and vascular injury, eventually leading to KLF2 downregulation, NF-kB activation, vascular dysfunctions and respiratory as well as multi-organ failure in COVID-19 patients. As a result of endothelial activation and endothelial dysfunction (ED), the levels of pro-inflammatory cytokines (IL-1β, IL-6, and TNF-α), chemokines (MCP-1), and acute phase reactants (IL-6, CRP, and D-dimer) are elevated. Therefore, ED contributes to COVID-19-associated vascular inflammation, particularly endothelialitis, coagulopathy in the lung, heart, kidney and intestine. Further research of the important role of ED in COVID-19 patients is warranted to offer targets for therapeutic intervention.

Supplementary Table 1. Patient demographic data

| ID | Age (yr) | WBC (10E9/L) | Lymphocyte (%) | ALT  (U/L) | AST  (U/L) | hs-CRP  (mg/L) | BUN  (mmol/L) | Sample collection since onset (day) | Hypertension | Diabetes |
| --- | --- | --- | --- | --- | --- | --- | --- | --- | --- | --- |
| XY1900358 | 70 | 4 | 17.6 | 33 | 20 | 7.4 | 3.74 | 17 | No | No |
| XY1900410 | 80 | 8.9 | 5 | 101 | 48 | 25.2 | 8.88 | 14 | No | No |
| XY1900429 | 55 | 10.54 | 16.7 | 18 | 13 | 20.7 | 9.25 | 18 | No | No |
| XY1900434 | 64 | 9.75 | 6.8 | 41 | 31 | 24.5 | NA | 19 | No | Yes |
| XY1900437 | 59 | 10.38 | 12.6 | 229 | 94 | 12.3 | 9.86 | 18 | No | Yes |
| XY1900503 | 93 | 9.84 | 22.1 | 34 | 37 | NA | 8.33 | 17 | Yes | No |
| XY1900509 | 70 | 5.48 | 23.4 | 72 | 40 | 3.9 | 11.72 | 13 | Yes | Yes |
| XY1900510 | 67 | 7.01 | 21.8 | NA | NA | 0.5 | NA | 29 | No | Yes |

Supplementary Table 2.

Please refer to a separate Excel spreedsheet in supplemental materials (separate file)

Supplementary Table 3. Primer list

| **Gene name** | **Forward primer** | **Reverse primer** |
| --- | --- | --- |
| KLF2 | CACGCACACAGGTGAGAA | ACAGATGGCACTGGAATGG |
| KLF4 | GAACCCACACAGGTGAGAAA | GTAGTGCCTGGTCAGTTCATC |
| NOS3 | CCGGAACAGCACAAGAGTTA | GTCTGTGTTACTGGACTCCTTC |
| VCAM1 | GGCTTGTGTGTTCGGTTTC | GGAGCTCTACTCATTCCCTAGA |
| CCL2 | GTCCCAAAGAAGCTGTGATCT | AGTCTTCGGAGTTTGGGTTTG |
| GCLM | GAGTTGCACAGCTGGATTCT | CCTCCCAGTAAGGCTGTAAATG |
| NQO1 | GGGATGAGACACCACTGTATTT | TCTCCTCATCCTGTACCTCTTT |
| EDN1 | AAGGCAACAGACCGTGAAA | GTCTTCAGCCCTGAGTTCTTT |
| ANGPT2 | GTGACTGCCACGGTGAATAA | GGGTCCTTAGCTGAGTTTGATG |
| THBD | ACGTGGATGACTGCATACTG | ACCAGGTCGTAGTTAGGGTAG |
| DKK1 | AGCACCTTGGATGGGTATTC | CTGATGACCGGAGACAAACA |
| SELE | GTGTATGTCCTCTGGAGAATGG | GAACCCATTGGCTGGATTTG |
| KLF1 | ATGAAGCGCCACCTTTGA | TGTGAAGAGACCACCAAACAG |
| KLF3 | AGTTCTTTGCCCTCCTTTGT | CATCACCCACCTAGATCGAAAG |
| KLF5 | CTTCCACAACAGGCCACTTA | GGAGCATCTCTGCTTGTCTATC |
| KLF6 | GGGTGTGGCTCTTTGCTTTA | AGCGTTAGTCACTGCTCATTTC |
| KLF7 | CTCACAGACCCACACACATAC | GGCTAGGGCAAGGCATAAA |
| KLF8 | CAGTGATGCTACCCTGAGTTAC | TGAGGCACAGAGAGGTGATA |
| KLF9 | AGAAGGAAAGGGAGCTGAAATAG | TTGCAGAGTCTCCTCTGAAATC |
| KLF10 | AGCCAGCATCCTCAACTATC | CGGCACATGGTATGTTCTTTC |
| KLF11 | CCCGAAGGAGGAACTATGTATG | CCTGTGTGAGTGCGAAGAT |
| KLF12 | AGGCATAGCCGGGAATTAAC | CACCATGTCGGGCTTTCTAT |
| KLF13 | GTCTGGGAGAGAGAGTGGAATA | TTTGGAACTGGGCATGAAGA |
| KLF14 | GTTGAGTTACATGTGGGAGGAG | CCCTAGCTGAAGAGACAAACAG |
| KLF15 | AACTGAAAGCGCCCTGAA | CCTCTGGAGGAGGCAAATAAA |
| KLF16 | CGAAAGCCATGGGAGTAGTT | ACTTGTGGACACCTGAGATTG |
| KLF17 | TCAGGAAGGGACTGGTAGAA | GGTGGGAGCGTTTGGTATAA |
| GAPDH | GATTCCACCCATGGCAAATTC | CTGGAAGATGGTGATGGGATT |

Supplementary Table 4. Source of antibodies

| **Antibody used** | **Vendor** | **Catlog No.** |
| --- | --- | --- |
| KLF2 | ZenBio Inc, Chengdu, China | 863587 |
| eNOS | ZenBio Inc, Chengdu, China | 250094 |
| ICAM1 | ZenBio Inc, Chengdu, China | 384648 |
| VCAM1 | ZenBio Inc, Chengdu, China | 220417 |
| Flag | Sigma-Aldrich,  [St. Louis,](https://www.google.com/search?sxsrf=ALeKk00EA0sAmfy3c0oVOD9RfT9Q40HiTA:1623428695941&q=St.+Louis&stick=H4sIAAAAAAAAAOPgE-LUz9U3sLC0SK5U4gAxzcoryrW0spOt9POL0hPzMqsSSzLz81A4VhmpiSmFpYlFJalFxYtYOYNL9BR88kszi3ewMgIA78eh6VIAAAA&sa=X&ved=2ahUKEwil_vSV_4_xAhVaL6YKHSR6DWQQmxMoATAregQINBAD) MI | F1804 |
| GAPDH | ProteinTech, Rosemont, IL | 60004-1-Ig |
| Tubulin | ProteinTech, Rosemont, IL | 11224-1-AP |

**Uncropped full gel scan for Figure 1A-1B**


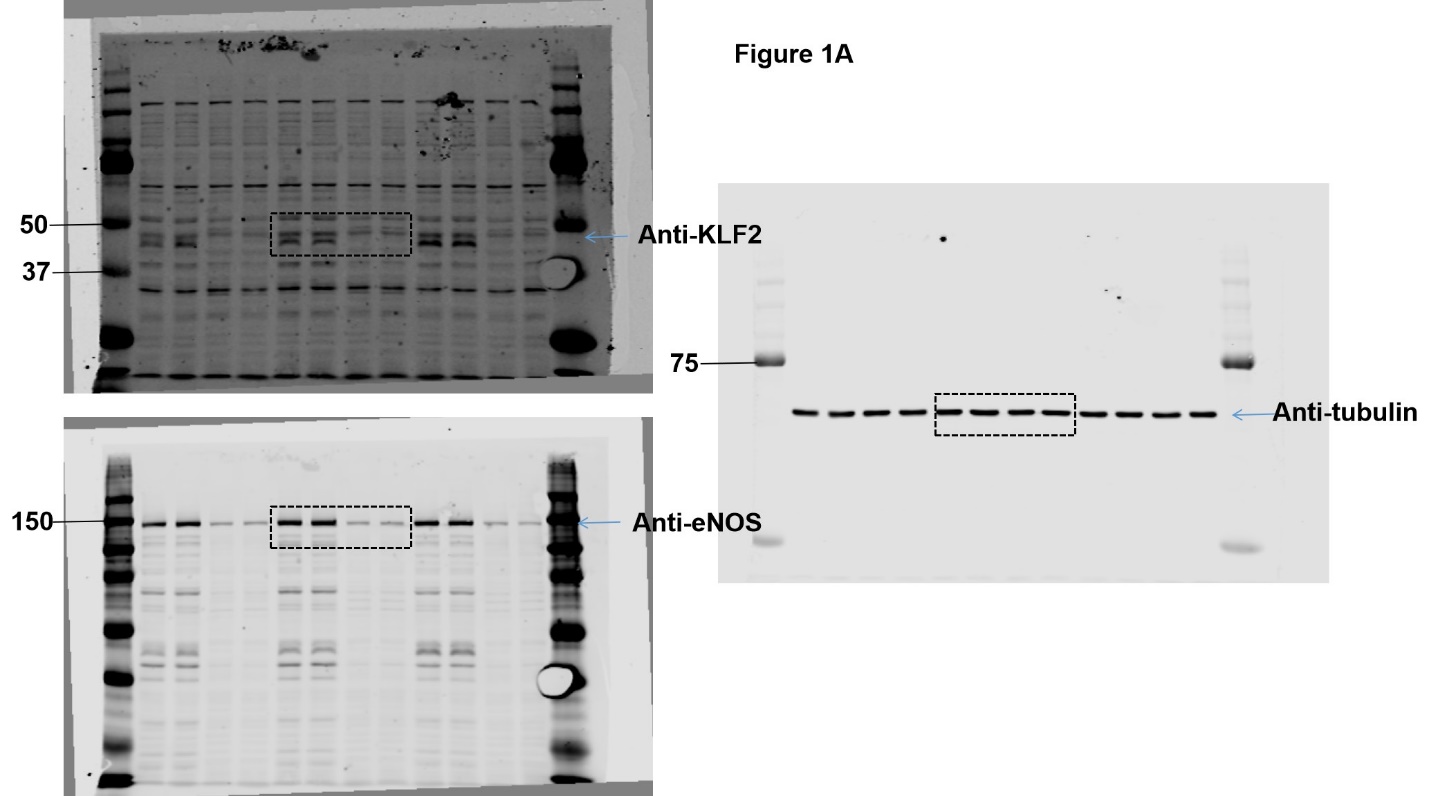


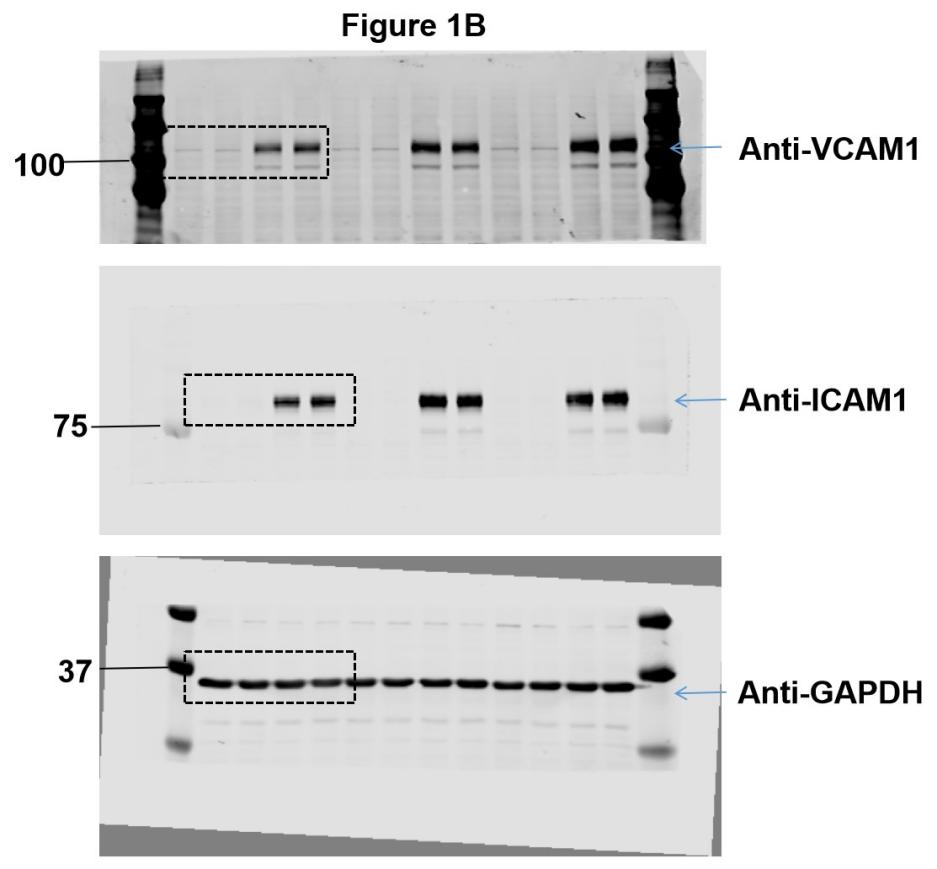


**Uncropped full gel scan for Figure 4C**

**
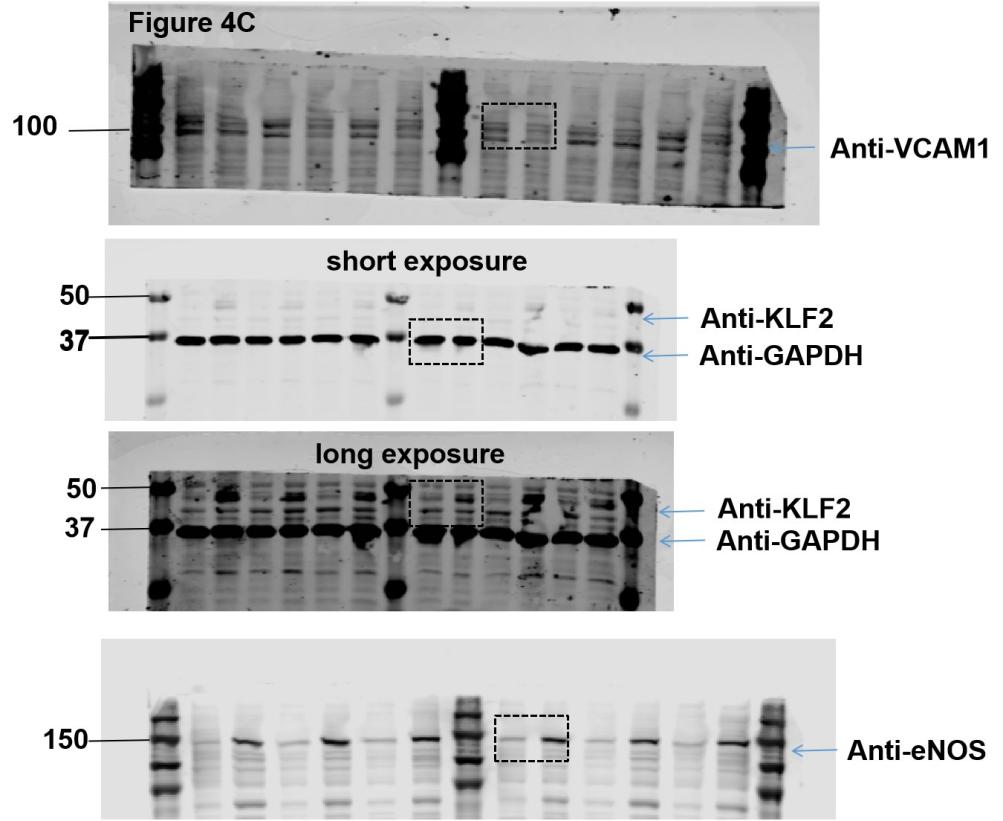
**

**Uncropped full gel scan for Figure 5C**


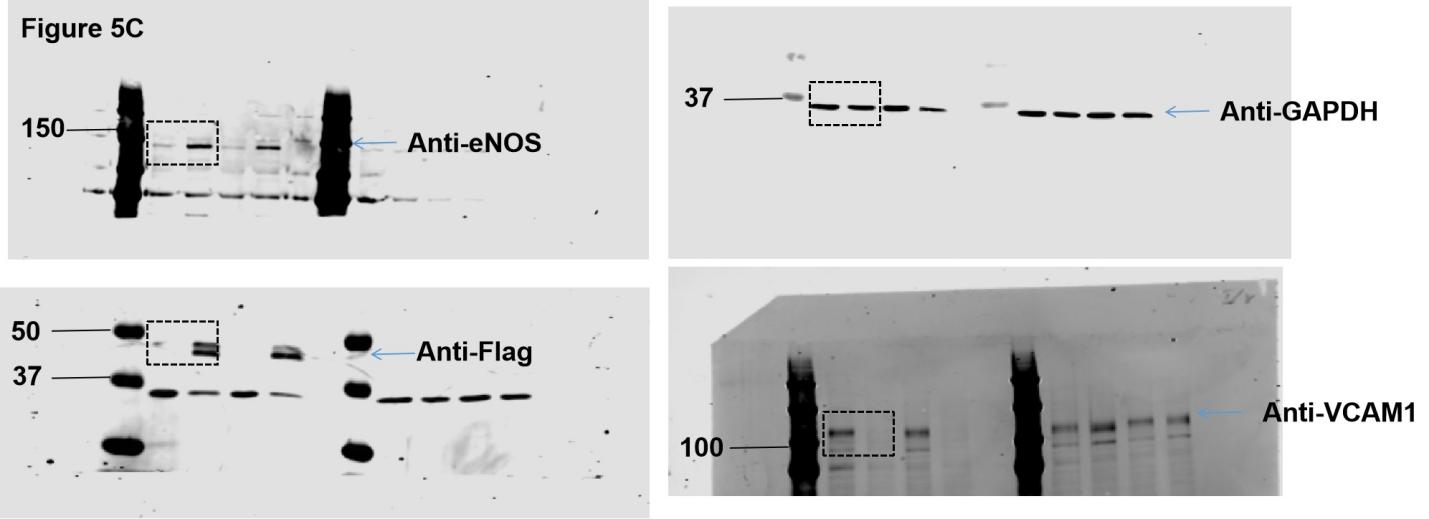

Supplement: Supplementary file 1 — Supplemental materials [file 41392_2021_690_MOESM1_ESM.docx]
