# Supplementary material for: The zinc finger transcription factor, KLF2, protects against COVID-19 associated endothelial dysfunction
Source: Signal Transduct Target Ther. 2021 Jul 12;6:266. doi: 10.1038/s41392-021-00690-5 (PMC8273371; doi:10.1038/s41392-021-00690-5)
Supplement: Supplementary file 2 — Supplemental Table 2 [file 41392_2021_690_MOESM2_ESM.pdf]

Table S2

Gene Symbol	Type	log2 (Atorvastatin / COVID19)	Qvalue (Atorvastatin / COVID19)
hsa_circ_0066883	circRNA	9.083313804	7.69E-13
RAMP3	mRNA	8.925866858	2.79E-07
hsa_circ_0072384	circRNA	8.526738246	4.83E-13
hsa_circ_0135692	circRNA	8.303350393	2.88E-12
hsa_circ_0085509	circRNA	8.287481138	4.32E-12
hsa_circ_0049378	circRNA	8.229758512	5.85E-12
hsa_circ_0072392	circRNA	8.204323468	9.44E-12
hsa_circ_0089104	circRNA	8.196153554	1.37E-11
PI16	mRNA	8.145641985	4.38E-27
hsa_circ_0089102	circRNA	8.107429466	2.56E-11
APLNR	mRNA	7.867697551	1.85E-31
hsa_circ_0072393	circRNA	7.830739154	4.00E-10
hsa_circ_0073037	circRNA	7.808261898	3.74E-10
hsa_circ_0070976	circRNA	7.791303328	1.18E-09
hsa_circ_0116649	circRNA	7.784870364	4.28E-10
SLC14A1	mRNA	7.763192517	7.23E-09
hsa_circ_0023365	circRNA	7.762297632	1.82E-09
hsa_circ_0085512	circRNA	7.728672812	5.93E-10
WNT9B	mRNA	7.698851992	1.25E-06
hsa_circ_0040843	circRNA	7.635098012	5.49E-09
hsa_circ_0070981	circRNA	7.599829374	1.19E-08

hsa_circ_0023372	circRNA	7.595994783	1.16E-08
hsa_circ_0028142	circRNA	7.594034488	3.58E-09
hsa_circ_0012669	circRNA	7.59201594	6.69E-09
hsa_circ_0125356	circRNA	7.591272793	1.70E-08
hsa_circ_0051712	circRNA	7.549885726	3.51E-08
hsa_circ_0031652	circRNA	7.483508909	2.41E-08
hsa_circ_0109019	circRNA	7.478223235	1.09E-08
hsa_circ_0049391	circRNA	7.441725803	1.90E-08
hsa_circ_0072382	circRNA	7.435696138	4.74E-08
hsa_circ_0116650	circRNA	7.421537259	2.55E-08
hsa_circ_0072381	circRNA	7.402078669	3.73E-08
hsa_circ_0066859	circRNA	7.380610606	2.78E-07
hsa_circ_0049389	circRNA	7.29976685	2.17E-07
hsa_circ_0072389	circRNA	7.255470769	4.09E-07
hsa_circ_0061352	circRNA	7.247678705	5.07E-07
hsa_circ_0073031	circRNA	7.188600206	4.52E-07
hsa_circ_0096117	circRNA	7.180701662	8.77E-07
hsa_circ_0078551	circRNA	7.098806625	3.02E-06
LYPD2	mRNA	7.072999245	6.35E-05
hsa_circ_0049380	circRNA	7.046916176	3.83E-06
hsa_circ_0022397	circRNA	7.032671355	4.93E-06
hsa_circ_0078552	circRNA	7.026952824	6.69E-06
hsa_circ_0012593	circRNA	7.02366479	4.63E-06
hsa_circ_0063695	circRNA	7.016652245	8.59E-06
hsa_circ_0049381	circRNA	7.010537004	5.93E-06

hsa_circ_0002085	circRNA	6.971985546	1.24E-05
hsa_circ_0118974	circRNA	6.925070823	1.77E-05
hsa_circ_0022367	circRNA	6.915233098	2.74E-05
PTGDS	mRNA	6.902616002	1.25E-09
hsa_circ_0059319	circRNA	6.853112827	7.52E-05
hsa_circ_0046342	circRNA	6.838500271	6.17E-05
STC1	mRNA	6.822389535	5.48E-11
hsa_circ_0012670	circRNA	6.819017255	9.78E-05
hsa_circ_0057582	circRNA	6.813578535	8.36E-05
hsa_circ_0133001	circRNA	6.811005764	9.44E-05
hsa_circ_0046340	circRNA	6.796971681	1.47E-04
hsa_circ_0107067	circRNA	6.785707493	1.37E-04
hsa_circ_0046332	circRNA	6.763216788	1.53E-04
hsa_circ_0129616	circRNA	6.761924222	1.98E-04
hsa_circ_0043110	circRNA	6.756972782	1.78E-04
hsa_circ_0065121	circRNA	6.755890407	1.87E-04
hsa_circ_0092106	circRNA	6.743804541	2.46E-04
hsa_circ_0116654	circRNA	6.730610346	3.22E-04
hsa_circ_0129918	circRNA	6.724269117	3.29E-04
hsa_circ_0131048	circRNA	6.723777013	2.68E-04
hsa_circ_0045571	circRNA	6.71069324	3.42E-04
hsa_circ_0124468	circRNA	6.701070841	0.004041322
hsa_circ_0117982	circRNA	6.699063699	4.13E-04
hsa_circ_0107790	circRNA	6.698432735	3.55E-04
hsa_circ_0118846	circRNA	6.696773192	4.64E-04

LCN10	mRNA	6.694198338	6.35E-05
hsa_circ_0028445	circRNA	6.691236751	4.54E-04
hsa_circ_0002181	circRNA	6.686850363	4.36E-04
hsa_circ_0070978	circRNA	6.675673616	5.09E-04
IGF1	mRNA	6.664789699	3.69E-07
hsa_circ_0046367	circRNA	6.664497147	5.23E-04
hsa_circ_0038969	circRNA	6.6641317	5.66E-04
hsa_circ_0131084	circRNA	6.656909731	0.005570055
hsa_circ_0066657	circRNA	6.647026469	6.20E-04
hsa_circ_0052593	circRNA	6.613411011	9.51E-04
hsa_circ_0057590	circRNA	6.613131451	0.00104695
hsa_circ_0031927	circRNA	6.59164838	0.010295267
hsa_circ_0008590	circRNA	6.589817811	0.001571234
hsa_circ_0109373	circRNA	6.589260705	0.009295291
hsa_circ_0022366	circRNA	6.586751688	0.001298463
hsa_circ_0129615	circRNA	6.584968901	0.001404631
hsa_circ_0034616	circRNA	6.583569732	0.009992095
hsa_circ_0127504	circRNA	6.581330361	0.001484661
hsa_circ_0039944	circRNA	6.579267997	0.001369657
hsa_circ_0046355	circRNA	6.569620247	0.001662903
hsa_circ_0130814	circRNA	6.568142143	0.002124251
hsa_circ_0028437	circRNA	6.549438192	0.002106136
hsa_circ_0053500	circRNA	6.548387716	0.002069243
hsa_circ_0111787	circRNA	6.543767608	0.002020602
hsa_circ_0137215	circRNA	6.537392898	0.013247105

hsa_circ_0137482	circRNA	6.528133082	0.002611907
hsa_circ_0083344	circRNA	6.525105373	0.014553601
hsa_circ_0026237	circRNA	6.506300994	0.00310414
hsa_circ_0093008	circRNA	6.49935351	0.017012251
hsa_circ_0058444	circRNA	6.497303705	0.003478101
hsa_circ_0033063	circRNA	6.493846978	0.003282839
hsa_circ_0106694	circRNA	6.49273739	0.003396258
hsa_circ_0084075	circRNA	6.48596917	0.003707153
hsa_circ_0066441	circRNA	6.484125184	0.020162475
hsa_circ_0029323	circRNA	6.48320037	0.004049817
hsa_circ_0027962	circRNA	6.475466945	0.021441401
hsa_circ_0024108	circRNA	6.475070197	0.02046482
hsa_circ_0137969	circRNA	6.473629877	0.004185228
hsa_circ_0094045	circRNA	6.473463406	0.004084694
hsa_circ_0048492	circRNA	6.466008306	0.00435372
hsa_circ_0083346	circRNA	6.463159134	0.004470026
hsa_circ_0095127	circRNA	6.460432011	0.005008875
hsa_circ_0073769	circRNA	6.457878827	0.004648741
hsa_circ_0054578	circRNA	6.450167518	0.024214353
hsa_circ_0118847	circRNA	6.449013238	0.006221082
hsa_circ_0106702	circRNA	6.447578747	0.005098241
hsa_circ_0083082	circRNA	6.433186476	0.006256371
hsa_circ_0064432	circRNA	6.432567778	0.027365877
hsa_circ_0002884	circRNA	6.418564289	0.029231486
hsa_circ_0006617	circRNA	6.415247259	0.006744034

ACKR1	mRNA	6.405625909	3.15E-20
hsa_circ_0040083	circRNA	6.404874618	0.031239515
hsa_circ_0069416	circRNA	6.395269302	0.008580363
hsa_circ_0046334	circRNA	6.385752836	0.008693714
hsa_circ_0062443	circRNA	6.3850174	0.008595462
hsa_circ_0053294	circRNA	6.382779423	0.008720533
hsa_circ_0123831	circRNA	6.374394725	0.009578232
hsa_circ_0071639	circRNA	6.366427979	0.010837734
hsa_circ_0002609	circRNA	6.364924672	0.038984901
hsa_circ_0045688	circRNA	6.361630963	0.010885303
hsa_circ_0064431	circRNA	6.361505201	0.040855585
hsa_circ_0070578	circRNA	6.358991961	0.040426358
hsa_circ_0113629	circRNA	6.34856351	0.041775448
hsa_circ_0138041	circRNA	6.347859441	0.042030674
hsa_circ_0064435	circRNA	6.335605612	0.046089535
LTF	mRNA	6.333641055	8.42E-06
hsa_circ_0089106	circRNA	6.329831797	0.048006277
hsa_circ_0110590	circRNA	6.329501266	0.014078056
hsa_circ_0098456	circRNA	6.317577709	0.015381553
hsa_circ_0032102	circRNA	6.312232659	0.015059445
hsa_circ_0116147	circRNA	6.306139327	0.015693019
hsa_circ_0085662	circRNA	6.295930514	0.016924811
hsa_circ_0046348	circRNA	6.295013472	0.017959634
hsa_circ_0035461	circRNA	6.284821108	0.018389717
hsa_circ_0105248	circRNA	6.276998017	0.01971967

hsa_circ_0131063	circRNA	6.262576506	0.022216785
hsa_circ_0071129	circRNA	6.258666876	0.022357021
hsa_circ_0067191	circRNA	6.257391644	0.022357021
hsa_circ_0057577	circRNA	6.253034626	0.024176646
hsa_circ_0028792	circRNA	6.251848297	0.02290569
hsa_circ_0060181	circRNA	6.25122002	0.023841572
hsa_circ_0039433	circRNA	6.245305059	0.023824715
hsa_circ_0016161	circRNA	6.243543322	0.026240322
hsa_circ_0019030	circRNA	6.235019826	0.025915174
hsa_circ_0087971	circRNA	6.227533525	0.027441597
hsa_circ_0043732	circRNA	6.22274795	0.027585818
hsa_circ_0070240	circRNA	6.197768076	0.032185139
hsa_circ_0127822	circRNA	6.192619636	0.034397993
FLJ30901	lncRNA	6.18267235	5.41E-11
hsa_circ_0100817	circRNA	6.178952433	0.037292408
hsa_circ_0080613	circRNA	6.172916055	0.038709216
hsa_circ_0042028	circRNA	6.167708661	0.038431808
hsa_circ_0104007	circRNA	6.163855099	0.040228232
hsa_circ_0067085	circRNA	6.16350288	0.040295251
hsa_circ_0036812	circRNA	6.149538137	0.043187929
hsa_circ_0046343	circRNA	6.149149681	0.042481448
hsa_circ_0118463	circRNA	6.12950106	0.04921866
LOC107987447	lncRNA	6.089936014	4.10E-05
FER1L6	mRNA	6.020087519	4.26E-04
NPPC	mRNA	5.959658032	7.58E-04

FCN3	mRNA	5.905745871	1.21E-47
UNC13A	mRNA	5.8779293	3.82E-19
BGIG9606_55309	mRNA	5.748190236	0.010460485
BGIG9606_45895	lncRNA	5.74765346	2.16E-04
CMKLR1	mRNA	5.723639372	4.96E-43
PLA1A	mRNA	5.571471834	2.57E-44
CADM3-AS1	lncRNA	5.557426214	1.91E-04
NPNT	mRNA	5.416559293	6.91E-07
KCNK3	mRNA	5.318790144	0.023295447
SCUBE1	mRNA	5.245141605	6.12E-33
LOC100128593	lncRNA	5.238802407	2.68E-04
BGIG9606_40064	lncRNA	4.924924352	0.016093354
LOC105369309	lncRNA	4.918318651	0.0011185
ABCC3	mRNA	4.883065113	2.60E-17
GJA9-MYCBP	lncRNA	4.804527348	0.029976676
RASD1	mRNA	4.791766877	1.00E-10
WFDC1	mRNA	4.756248466	9.20E-04
LOC107984376	lncRNA	4.756239216	0.005987723
LCN6	mRNA	4.686437158	0.002063913
PLA2G3	mRNA	4.553097596	5.74E-05
CRP	mRNA	4.548003487	0.015489828
CADM3	mRNA	4.533335014	1.86E-04
LUCAT1	lncRNA	4.532924402	8.33E-12
BGIG9606_45098	lncRNA	4.471173636	0.034470492
CDHR1	mRNA	4.374093204	4.38E-12

MYOM3	mRNA	4.288995898	3.31E-15
LOC107984751	mRNA	4.280109267	0.009225594
NDNF	mRNA	4.271552693	0.025778246
LINC01249	lncRNA	4.206672528	0.019327101
LOC107987228	lncRNA	4.199694993	0.001832557
PCSK9	mRNA	4.193824491	3.08E-14
BGIG9606_54754	mRNA	4.193503739	4.49E-04
TH	mRNA	4.160536714	0.018756284
FABP3	mRNA	4.128364717	2.23E-10
ZBTB7C	mRNA	4.117783506	0.048178219
TNXB	mRNA	4.088689942	2.12E-23
NGEF	mRNA	4.088676706	0.010512338
LINC02561	lncRNA	4.088192916	1.75E-05
BGIG9606_40936	lncRNA	4.087425289	0.001050554
KCNN2	mRNA	4.071623741	6.69E-08
LOC105379030	lncRNA	4.061188595	0.031214559
PMCH	mRNA	4.020600158	1.34E-04
L1CAM	mRNA	4.005309409	1.49E-04
hsa_circ_0049394	circRNA	3.963303758	0.018809734
KCNN4	mRNA	3.938601739	2.09E-04
LYPD5	mRNA	3.849004499	6.22E-05
S100P	mRNA	3.842598127	3.97E-04
CABP1	mRNA	3.818310838	0.006128309
ST6GALNAC1	mRNA	3.732560597	3.72E-10
NOV	mRNA	3.72644178	2.36E-06

hsa_circ_0031929	circRNA	3.703204264	0.018256487
TMEM151A	mRNA	3.66035722	6.68E-04
TESC	mRNA	3.558648267	7.34E-09
NMRAL2P	pseudo	3.551345543	7.40E-06
ADM2	mRNA	3.535737748	2.36E-10
BGIG9606_44938	lncRNA	3.529406909	0.03523272
BGIG9606_56233	lncRNA	3.525021052	2.83E-08
IGFBP5	mRNA	3.515908965	1.99E-05
PTPRR	mRNA	3.475979046	0.011147833
hsa_circ_0072391	circRNA	3.419949605	6.19E-35
hsa_circ_0049382	circRNA	3.414432897	5.21E-04
PNPLA3	mRNA	3.386399611	2.17E-14
NID2	mRNA	3.361663951	2.11E-04
CFP	mRNA	3.361391696	5.80E-21
COL17A1	mRNA	3.358459822	9.29E-06
HMGCS1	mRNA	3.345856079	3.48E-42
JPH4	mRNA	3.328612533	9.83E-11
CCL21	mRNA	3.327092203	3.28E-04
NQO1	mRNA	3.32047543	7.02E-19
AQP3	mRNA	3.314165929	0.001453929
hsa_circ_0125714	circRNA	3.306658437	0.008418521
ACP5	mRNA	3.280432852	4.44E-18
CFAP161	mRNA	3.273040884	2.53E-07
hsa_circ_0073041	circRNA	3.253202164	1.23E-06
hsa_circ_0004983	circRNA	3.242963082	1.60E-11

CDH23	mRNA	3.239253686	2.70E-14
SLCO4C1	mRNA	3.231790645	0.01646804
hsa_circ_0012671	circRNA	3.201038683	7.10E-12
ADAP2	mRNA	3.198156796	2.48E-07
hsa_circ_0129088	circRNA	3.185094552	0.004514168
hsa_circ_0133744	circRNA	3.174720787	1.91E-42
LOC100505664	lncRNA	3.151962872	1.04E-05
hsa_circ_0040084	circRNA	3.151075095	3.76E-11
IDI2-AS1	lncRNA	3.150859335	2.83E-08
BGIG9606_45664	lncRNA	3.130420554	0.004582272
hsa_circ_0002579	circRNA	3.130080279	6.51E-57
LYVE1	mRNA	3.114952004	5.59E-05
ADAMTS5	mRNA	3.098151683	9.31E-04
LDLR	mRNA	3.086425925	2.35E-28
hsa_circ_0085513	circRNA	3.082928109	0.005281932
ALPL	mRNA	3.074250457	1.06E-05
INSIG1	mRNA	3.071876309	1.05E-40
LINC00482	lncRNA	3.070293533	0.029406003
ADAMTS4	mRNA	3.067846022	1.93E-06
hsa_circ_0070980	circRNA	3.066167344	0.00701421
hsa_circ_0023377	circRNA	3.058825279	3.39E-38
BAALC	mRNA	3.030166518	8.55E-11
hsa_circ_0049383	circRNA	3.019900782	3.03E-23
hsa_circ_0072387	circRNA	3.002544452	0.003635015
hsa_circ_0049398	circRNA	2.993451997	3.26E-12

IZUMO1	mRNA	2.981450511	0.049030995
hsa_circ_0023375	circRNA	2.981290404	8.04E-36
hsa_circ_0131233	circRNA	2.97565799	4.06E-09
hsa_circ_0083081	circRNA	2.959294362	1.01E-17
hsa_circ_0049393	circRNA	2.956038583	1.02E-13
hsa_circ_0023374	circRNA	2.92548462	1.80E-44
hsa_circ_0008621	circRNA	2.919675443	4.04E-27
hsa_circ_0023376	circRNA	2.912899444	3.46E-11
HDAC9	mRNA	2.910441221	6.98E-10
ADAMTS1	mRNA	2.909820903	7.35E-13
hsa_circ_0023370	circRNA	2.909644316	1.28E-15
hsa_circ_0049395	circRNA	2.899830122	5.91E-10
hsa_circ_0003892	circRNA	2.897805556	0.001317067
MYOZ2	mRNA	2.896573923	0.001752416
hsa_circ_0049377	circRNA	2.894889289	9.96E-08
hsa_circ_0012673	circRNA	2.887780037	3.28E-12
hsa_circ_0133745	circRNA	2.884419887	1.62E-53
hsa_circ_0023371	circRNA	2.883167844	5.94E-08
hsa_circ_0049379	circRNA	2.882599247	0.009673898
hsa_circ_0070975	circRNA	2.876274913	2.10E-11
hsa_circ_0023369	circRNA	2.869198636	6.07E-47
hsa_circ_0049400	circRNA	2.868665246	2.08E-13
ACAT2	mRNA	2.85759713	3.17E-24
hsa_circ_0005360	circRNA	2.854094383	1.73E-30
DHCR24	mRNA	2.853053153	1.08E-27

hsa_circ_0012676	circRNA	2.853023723	7.26E-14
MSMO1	mRNA	2.849810327	8.13E-49
LAMA1	mRNA	2.846013797	8.15E-04
BGIG9606_38124	lncRNA	2.83753143	7.55E-04
LOC102723825	lncRNA	2.830354424	0.03787044
hsa_circ_0012668	circRNA	2.828101844	7.12E-09
RASGRF2	mRNA	2.825856914	0.012525889
hsa_circ_0049385	circRNA	2.823990983	9.19E-11
hsa_circ_0078553	circRNA	2.815558529	1.40E-20
hsa_circ_0006877	circRNA	2.805639712	8.50E-47
hsa_circ_0022395	circRNA	2.804675088	1.46E-20
CBFA2T3	mRNA	2.803512655	0.002297853
hsa_circ_0129619	circRNA	2.799459757	1.17E-55
WI2-87327B8.2	lncRNA	2.78907871	0.007014026
hsa_circ_0000313	circRNA	2.785716035	1.91E-42
GPR132	mRNA	2.783164723	2.93E-04
hsa_circ_0135694	circRNA	2.775361454	1.18E-45
SQLE	mRNA	2.762637546	5.28E-47
hsa_circ_0049390	circRNA	2.759767972	7.35E-21
KLF4	mRNA	2.754839195	7.07E-12
hsa_circ_0022391	circRNA	2.741043617	1.38E-18
SH3TC2	mRNA	2.738129278	2.62E-24
AREG	mRNA	2.733441421	0.005987723
ZNF503-AS1	lncRNA	2.728291993	0.020555793
LOC105378180	lncRNA	2.728139558	0.028955737

PTPRH	mRNA	2.723678976	6.07E-04
LOC101929583	pseudo	2.723587695	0.006990852
hsa_circ_0113656	circRNA	2.708382694	1.32E-15
hsa_circ_0135693	circRNA	2.704168113	1.71E-54
DHCR7	mRNA	2.68932297	4.05E-31
hsa_circ_0073036	circRNA	2.688555107	1.06E-12
hsa_circ_0022382	circRNA	2.686478413	9.92E-16
hsa_circ_0049392	circRNA	2.672599884	1.33E-09
SLC7A11	mRNA	2.661284471	1.13E-12
NEURL1B	mRNA	2.659905062	4.82E-49
C17orf107	mRNA	2.653389113	6.07E-09
F2RL3	mRNA	2.650882918	0.036868119
hsa_circ_0129618	circRNA	2.633198636	4.88E-16
FADS2	mRNA	2.629920274	2.20E-34
RIMBP3	mRNA	2.624212264	2.49E-04
LOC102724297	lncRNA	2.620585483	1.07E-04
NOSTRIN	mRNA	2.614832558	1.60E-08
SLC12A8	mRNA	2.614167985	1.06E-05
hsa_circ_0022392	circRNA	2.603133927	6.76E-45
PLAC9	mRNA	2.602059371	1.06E-11
OSGIN1	mRNA	2.600251801	5.66E-10
PCDH12	mRNA	2.598570881	1.00E-10
hsa_circ_0096042	circRNA	2.5959774	7.60E-62
LINC02544	lncRNA	2.595907274	4.26E-04
KLF2	mRNA	2.593022216	3.53E-10

hsa_circ_0022393	circRNA	2.585800349	5.95E-50
hsa_circ_0070979	circRNA	2.580872717	1.41E-08
hsa_circ_0022384	circRNA	2.578911895	2.66E-20
FGF18	mRNA	2.576466793	0.036444041
hsa_circ_0022388	circRNA	2.57311354	2.52E-46
hsa_circ_0085510	circRNA	2.566168692	3.28E-23
hsa_circ_0089103	circRNA	2.55584359	5.63E-16
hsa_circ_0022386	circRNA	2.55338855	3.75E-12
hsa_circ_0135691	circRNA	2.551043048	2.01E-41
EDNRB	mRNA	2.548038342	0.001892441
hsa_circ_0099426	circRNA	2.542699894	0.009138452
hsa_circ_0022394	circRNA	2.522464299	5.11E-13
LINGO1	mRNA	2.522180708	4.64E-09
HMGCR	mRNA	2.516943183	5.60E-43
hsa_circ_0089096	circRNA	2.516233928	5.32E-25
hsa_circ_0125357	circRNA	2.515099034	1.03E-07
PTHLH	mRNA	2.496689949	0.003479776
hsa_circ_0040847	circRNA	2.495044342	1.98E-10
hsa_circ_0089100	circRNA	2.492948387	2.07E-14
ANO1	mRNA	2.483147883	3.91E-04
PLA2G4C	mRNA	2.47800204	0.00166192
CLIC5	mRNA	2.476139666	8.86E-05
FGR	mRNA	2.463690361	3.35E-05
hsa_circ_0109760	circRNA	2.461190435	0.01920531
hsa_circ_0073039	circRNA	2.437717883	2.69E-50

BGIG9606_37996	lncRNA	2.436693286	3.63E-17
hsa_circ_0070972	circRNA	2.432974106	1.38E-05
hsa_circ_0014480	circRNA	2.423288198	7.66E-14
hsa_circ_0051709	circRNA	2.42030737	0.001861979
LOC286297	pseudo	2.404782684	0.021262491
hsa_circ_0070977	circRNA	2.399778638	4.58E-08
LINC00632	lncRNA	2.399697898	1.50E-08
CYP51A1	mRNA	2.397903554	1.17E-37
TNIP3	mRNA	2.39602193	0.01496164
GCKR	mRNA	2.393637931	3.31E-04
hsa_circ_0080999	circRNA	2.392622895	2.02E-34
hsa_circ_0073042	circRNA	2.390232536	1.21E-32
PCSK6	mRNA	2.389517622	0.005471176
LOC101927834	lncRNA	2.384380835	0.019869217
hsa_circ_0046358	circRNA	2.382099493	4.41E-15
hsa_circ_0073040	circRNA	2.376163369	1.31E-08
hsa_circ_0070971	circRNA	2.370096669	1.40E-04
SULT1A3	mRNA	2.351915105	2.12E-06
KITLG	mRNA	2.350566792	0.001616193
hsa_circ_0022383	circRNA	2.348311195	4.25E-09
GP6	mRNA	2.340941654	2.13E-04
ASS1	mRNA	2.329405723	2.14E-19
HES2	mRNA	2.328499662	1.79E-07
hsa_circ_0014481	circRNA	2.320173485	6.22E-14
PCAT1	lncRNA	2.320016069	5.57E-04

KCNMB1	mRNA	2.297423723	0.003330702
LINC00520	lncRNA	2.29629799	0.002928337
MVD	mRNA	2.294506504	3.00E-17
hsa_circ_0073032	circRNA	2.294341759	4.60E-26
hsa_circ_0081002	circRNA	2.293749909	1.33E-09
IL16	mRNA	2.287708626	1.57E-08
hsa_circ_0073033	circRNA	2.276438124	5.72E-06
LOC101929951	lncRNA	2.266833499	0.042012212
STOX2	mRNA	2.262479198	9.03E-37
EBF1	mRNA	2.259530801	9.20E-04
MIR4697HG	lncRNA	2.256719882	0.027492506
MALL	mRNA	2.24589092	3.65E-09
LOC100996351	lncRNA	2.232970023	9.69E-04
hsa_circ_0046345	circRNA	2.232872298	1.34E-05
LOC101928561	lncRNA	2.23062645	0.027327496
hsa_circ_0040845	circRNA	2.230459434	7.92E-07
ECM1	mRNA	2.223044715	3.27E-10
hsa_circ_0081000	circRNA	2.221761174	6.75E-28
ATF3	mRNA	2.219352987	6.59E-11
LOC100287846	lncRNA	2.21827701	0.037313007
hsa_circ_0081001	circRNA	2.210312651	1.90E-33
C3orf36	lncRNA	2.205231313	0.005077536
hsa_circ_0056017	circRNA	2.182367754	2.68E-05
MEI1	mRNA	2.181679442	1.14E-08
LOC105369724	lncRNA	2.173715178	0.012645233

hsa_circ_0046366	circRNA	2.167922988	6.37E-17
RASGRP3	mRNA	2.166991213	1.42E-06
BGIG9606_52209	lncRNA	2.163313043	4.71E-04
SLCO4A1	mRNA	2.161042467	2.17E-10
ATP10A	mRNA	2.149314259	0.012344135
CAPN14	mRNA	2.147462815	4.90E-04
PTAFR	mRNA	2.142183153	0.001616537
MYLK	mRNA	2.138563995	2.76E-10
LOC107984440	lncRNA	2.133889737	0.02831667
hsa_circ_0083347	circRNA	2.133064101	7.91E-36
hsa_circ_0046327	circRNA	2.132798539	1.38E-18
SYT7	mRNA	2.129080237	3.42E-11
SHISAL1	mRNA	2.128917589	0.01599232
hsa_circ_0067047	circRNA	2.1277809	4.78E-04
LINC00346	lncRNA	2.123508005	5.21E-07
hsa_circ_0110857	circRNA	2.119439668	5.17E-14
LRRC73	mRNA	2.110449347	1.42E-06
DUSP8	mRNA	2.108502159	4.22E-06
IDI1	mRNA	2.105366595	3.60E-26
hsa_circ_0062067	circRNA	2.102128968	3.20E-07
APOLD1	mRNA	2.098455555	0.007524731
hsa_circ_0135551	circRNA	2.088301303	2.32E-16
hsa_circ_0083349	circRNA	2.08805601	3.60E-23
hsa_circ_0062068	circRNA	2.083172967	8.38E-06
hsa_circ_0022378	circRNA	2.078611515	3.23E-22

hsa_circ_0083345	circRNA	2.077628574	4.27E-08
B4GALNT1	mRNA	2.077512024	0.010504764
hsa_circ_0046346	circRNA	2.077363344	1.88E-11
hsa_circ_0089101	circRNA	2.077044505	3.25E-17
hsa_circ_0046360	circRNA	2.072788292	3.88E-13
FDFT1	mRNA	2.072667714	1.05E-27
IGSF9B	mRNA	2.071645951	2.72E-04
hsa_circ_0046352	circRNA	2.070844065	3.79E-05
FASN	mRNA	2.068286024	3.39E-25
LOC105375591	lncRNA	2.067964721	4.34E-21
LOC105374062	lncRNA	2.06444735	0.046864188
MRVI1	mRNA	2.056870982	3.88E-08
TFF3	mRNA	2.055419697	9.92E-10
NR4A1	mRNA	2.053464007	2.92E-05
IL32	mRNA	2.050795833	7.72E-06
hsa_circ_0046353	circRNA	2.047828461	2.20E-06
hsa_circ_0022380	circRNA	2.043520877	8.66E-20
LSS	mRNA	2.030938273	1.57E-12
ELOVL6	mRNA	2.027838919	3.29E-14
MMAB	mRNA	2.026923435	3.17E-24
hsa_circ_0046370	circRNA	2.019547103	4.76E-10
hsa_circ_0046341	circRNA	2.013816117	3.75E-23
HSPA12B	mRNA	2.013800369	3.75E-11
FERMT3	mRNA	2.013627825	2.84E-06
BDKRB2	mRNA	2.013318678	0.02180423

hsa_circ_0014479	circRNA	2.008128062	2.56E-05
hsa_circ_0062062	circRNA	2.007403661	1.21E-07
hsa_circ_0046339	circRNA	2.00528733	0.001006862
DHCR24-DT	lncRNA	1.991177923	0.017989181
hsa_circ_0046359	circRNA	1.99100139	2.56E-08
NCR3LG1	mRNA	1.989742285	1.73E-13
MVK	mRNA	1.988686751	3.67E-21
hsa_circ_0057588	circRNA	1.986443124	0.006921116
ADAMTSL2	mRNA	1.972765712	0.004382428
LFNG	mRNA	1.970502258	1.60E-08
LOC107984316	lncRNA	1.96943625	1.08E-14
TBXA2R	mRNA	1.969047726	2.77E-07
hsa_circ_0135548	circRNA	1.965716864	4.17E-31
TMEM156	mRNA	1.95892511	0.008383397
LOC102723911	lncRNA	1.95867826	0.036644536
hsa_circ_0022376	circRNA	1.956054783	2.11E-25
hsa_circ_0083348	circRNA	1.93930837	1.31E-15
hsa_circ_0135549	circRNA	1.921885381	1.25E-10
LHX6	mRNA	1.915279242	9.50E-07
hsa_circ_0033065	circRNA	1.909517739	4.56E-21
ISG20	mRNA	1.908130897	6.62E-05
hsa_circ_0057571	circRNA	1.906765964	4.76E-04
IFI27	mRNA	1.906732453	9.83E-11
SLC9A3R2	mRNA	1.905867697	5.77E-08
RCOR2	mRNA	1.905213837	1.23E-05

hsa_circ_0022381	circRNA	1.903749725	2.28E-16
TM7SF2	mRNA	1.899155872	7.18E-13
hsa_circ_0092118	circRNA	1.898627205	3.17E-05
hsa_circ_0135550	circRNA	1.896383334	1.47E-13
BGIG9606_46975	lncRNA	1.89428479	0.033623386
GADD45G	mRNA	1.892653289	9.94E-04
ALDH3A1	mRNA	1.89227101	0.040332505
LTK	mRNA	1.891568636	0.040333586
CDCP2	mRNA	1.881532942	0.021644815
hsa_circ_0046364	circRNA	1.877570644	1.16E-08
SLC37A2	mRNA	1.87496736	3.45E-04
hsa_circ_0033064	circRNA	1.873468601	7.16E-05
hsa_circ_0118835	circRNA	1.872391205	0.00142553
ADAM15	mRNA	1.868034202	1.01E-15
ST8SIA6	mRNA	1.864570735	8.60E-09
hsa_circ_0097081	circRNA	1.863622312	0.004978369
hsa_circ_0046368	circRNA	1.860521331	2.58E-07
hsa_circ_0092396	circRNA	1.860409118	1.35E-18
PAQR5	mRNA	1.857675713	0.00425844
OAS2	mRNA	1.852170701	1.63E-06
MMP1	mRNA	1.850763755	1.11E-05
hsa_circ_0045690	circRNA	1.847974533	0.024273142
hsa_circ_0012592	circRNA	1.847442285	1.28E-10
LAMB3	mRNA	1.843840263	0.002385004
hsa_circ_0027956	circRNA	1.842087564	2.77E-09

hsa_circ_0077013	circRNA	1.836198736	0.01971967
HECW2	mRNA	1.835534579	1.91E-06
KCNJ12	mRNA	1.833391135	2.92E-08
SRXN1	mRNA	1.83189917	2.34E-06
BGIG9606_46407	lncRNA	1.8313111	0.011708545
TMEM154	mRNA	1.820787512	1.55E-08
FADS1	mRNA	1.81443859	1.94E-16
MMP10	mRNA	1.813285776	8.52E-04
NPW	mRNA	1.812561113	0.012226734
hsa_circ_0046357	circRNA	1.812463619	5.97E-16
LOC105376436	lncRNA	1.799842402	0.040539506
LOC105374454	lncRNA	1.794832065	0.008499769
hsa_circ_0046373	circRNA	1.793305598	6.30E-20
hsa_circ_0022770	circRNA	1.788148657	1.00E-05
TPBGL	mRNA	1.78402245	0.021873758
GSN	mRNA	1.783343623	2.12E-09
hsa_circ_0028143	circRNA	1.781250592	9.15E-05
ADAMTS9	mRNA	1.766999157	1.66E-10
KDELR3	mRNA	1.761391012	1.71E-17
hsa_circ_0092119	circRNA	1.761157656	2.09E-04
hsa_circ_0045686	circRNA	1.758334848	0.018254401
DRAXIN	mRNA	1.755468507	1.43E-06
CCRL2	mRNA	1.753159986	3.66E-08
C3orf70	mRNA	1.752153353	1.58E-04
hsa_circ_0107789	circRNA	1.747557325	9.79E-12

hsa_circ_0007674	circRNA	1.74636093	0.003923499
hsa_circ_0088342	circRNA	1.743398488	0.018809734
G6PD	mRNA	1.743074166	3.77E-10
hsa_circ_0135818	circRNA	1.743030784	3.83E-06
hsa_circ_0005424	circRNA	1.742408058	3.08E-05
SP6	mRNA	1.740490083	5.40E-04
ROPN1L	mRNA	1.740465143	1.39E-05
NR4A3	mRNA	1.738550569	0.026841515
HYAL1	mRNA	1.737646778	1.40E-16
HLX	mRNA	1.737128776	3.98E-04
hsa_circ_0057592	circRNA	1.733923209	0.004893452
CXCL12	mRNA	1.732529367	0.004811714
LRP8	mRNA	1.731438601	1.72E-09
MIAT	lncRNA	1.730955145	2.32E-04
hsa_circ_0019510	circRNA	1.730910379	1.28E-06
hsa_circ_0085656	circRNA	1.728716416	5.74E-21
HS6ST3	mRNA	1.728150513	0.006690197
hsa_circ_0088343	circRNA	1.726097118	2.29E-04
TXNRD1	mRNA	1.725395619	6.09E-09
SCD	mRNA	1.722688339	1.45E-13
LOC105374538	lncRNA	1.717276661	0.002217735
MYO7B	mRNA	1.71447175	4.39E-04
ADIRF	mRNA	1.713360141	8.80E-06
hsa_circ_0092124	circRNA	1.713156227	5.25E-08
FGFR3	mRNA	1.712548128	9.10E-06

hsa_circ_0092112	circRNA	1.71134947	8.88E-08
hsa_circ_0046344	circRNA	1.708589298	0.001480563
hsa_circ_0135821	circRNA	1.708201391	6.22E-17
hsa_circ_0092105	circRNA	1.707566255	2.09E-05
hsa_circ_0088339	circRNA	1.706405838	3.78E-09
MGAT5B	mRNA	1.700459042	6.43E-06
hsa_circ_0130815	circRNA	1.699586164	0.033120176
CHPF	mRNA	1.698085503	0.004306134
hsa_circ_0097082	circRNA	1.697596136	2.99E-05
hsa_circ_0073957	circRNA	1.69748702	0.026581813
hsa_circ_0057591	circRNA	1.697121134	0.003682852
hsa_circ_0118977	circRNA	1.691915683	0.016570356
hsa_circ_0063623	circRNA	1.690538021	8.92E-19
hsa_circ_0009124	circRNA	1.689676437	4.21E-05
hsa_circ_0066447	circRNA	1.688814776	3.70E-04
hsa_circ_0092116	circRNA	1.687002364	8.95E-04
HR	mRNA	1.679244149	4.13E-04
hsa_circ_0045698	circRNA	1.671149505	0.001097498
hsa_circ_0022371	circRNA	1.67095461	9.03E-13
hsa_circ_0022370	circRNA	1.665912246	4.75E-19
BCL6B	mRNA	1.661609586	8.98E-06
BGIG9606_44026	lncRNA	1.661196119	0.006249572
hsa_circ_0007044	circRNA	1.661130007	4.90E-08
hsa_circ_0092117	circRNA	1.660762315	9.94E-04
SLC7A11-AS1	lncRNA	1.657492955	1.78E-04

TMEM173	mRNA	1.656150016	8.35E-14
CEMIP	mRNA	1.656098482	6.61E-06
LOC101929846	lncRNA	1.655785081	0.028839426
hsa_circ_0091731	circRNA	1.653998667	4.33E-09
hsa_circ_0014478	circRNA	1.652979564	3.53E-12
MAP2	mRNA	1.652145109	2.54E-04
hsa_circ_0026553	circRNA	1.650402758	2.11E-04
S100A3	mRNA	1.649289129	0.003407091
hsa_circ_0002150	circRNA	1.649232741	0.034843649
hsa_circ_0004388	circRNA	1.649188004	0.00105734
hsa_circ_0066444	circRNA	1.642838999	4.09E-08
NDRG1	mRNA	1.642364085	5.62E-11
hsa_circ_0045687	circRNA	1.64218094	0.004084694
hsa_circ_0019512	circRNA	1.642056827	6.08E-19
hsa_circ_0045705	circRNA	1.639098431	0.028796401
BATF3	mRNA	1.631187322	0.039443809
SLCO4A1-AS1	lncRNA	1.629152808	1.75E-05
THBD	mRNA	1.626365108	6.47E-05
SSTR5	mRNA	1.624989624	0.032727052
CABP4	mRNA	1.624223708	3.05E-06
ITGB4	mRNA	1.623097707	1.22E-05
hsa_circ_0092123	circRNA	1.621736877	0.019440651
hsa_circ_0012716	circRNA	1.620287349	9.89E-05
HSD17B7	mRNA	1.61274227	3.15E-18
hsa_circ_0097083	circRNA	1.610517032	0.001345251

LOC100287036	mRNA	1.609040708	0.017401456
hsa_circ_0118838	circRNA	1.608873804	2.11E-09
hsa_circ_0014482	circRNA	1.607413667	6.83E-06
hsa_circ_0004564	circRNA	1.604602779	3.77E-04
hsa_circ_0085657	circRNA	1.602395139	1.13E-06
HSPC324	lncRNA	1.594496512	0.004513201
BGIG9606_51236	lncRNA	1.590891357	0.025849556
hsa_circ_0046328	circRNA	1.585945016	2.56E-04
hsa_circ_0066446	circRNA	1.584256742	4.54E-07
hsa_circ_0092128	circRNA	1.580136836	8.67E-05
CHRNE	mRNA	1.579236774	0.001632829
COL9A3	mRNA	1.579161763	4.71E-04
hsa_circ_0118468	circRNA	1.578372347	0.002015551
LOC105369378	lncRNA	1.578008861	0.001089624
SREBF2	mRNA	1.572897445	1.19E-30
ERG28	mRNA	1.571735196	1.05E-19
hsa_circ_0085651	circRNA	1.570701724	9.22E-15
ZNF295-AS1	lncRNA	1.570274373	0.025528965
IGFBP6	mRNA	1.569471361	7.33E-08
TUBA4A	mRNA	1.565578655	4.42E-09
LOC102723761	lncRNA	1.565503066	0.021754705
GDF15	mRNA	1.564245464	1.45E-04
hsa_circ_0046374	circRNA	1.557166834	2.04E-09
LOC105369194	lncRNA	1.553022929	0.001993163
DERL3	mRNA	1.55030506	2.08E-04

ALS2CL	mRNA	1.548568416	1.39E-10
TRIM7	mRNA	1.546695618	0.001354073
hsa_circ_0045706	circRNA	1.546131813	0.009741298
LOC102723733	lncRNA	1.542836528	1.82E-04
OLFM1	mRNA	1.536858587	0.00285412
FAM95B1	lncRNA	1.536834996	0.020888454
TMEM97	mRNA	1.53662413	2.81E-07
hsa_circ_0063614	circRNA	1.536150563	2.15E-06
DOC2GP	pseudo	1.533268306	0.016605846
LOC107984011	lncRNA	1.532243703	0.041670115
hsa_circ_0019513	circRNA	1.532037445	4.11E-04
hsa_circ_0044653	circRNA	1.52930749	6.87E-08
LOC105374836	lncRNA	1.526538582	0.005216402
hsa_circ_0022365	circRNA	1.524894922	5.20E-05
hsa_circ_0085647	circRNA	1.523283854	4.79E-15
TUBA1A	mRNA	1.517914689	1.68E-14
NUDT8	mRNA	1.515674433	4.31E-07
hsa_circ_0045708	circRNA	1.511156578	0.002734751
hsa_circ_0063619	circRNA	1.507018105	8.77E-24
hsa_circ_0042351	circRNA	1.505955576	9.35E-06
hsa_circ_0092113	circRNA	1.501231143	1.50E-05
EPDR1	mRNA	1.499941067	0.010055482
HSD17B7P2	pseudo	1.498426282	0.006171388
hsa_circ_0092122	circRNA	1.493257983	6.62E-06
MATN1	mRNA	1.485469935	0.026419769

DLGAP1-AS2	lncRNA	1.484551002	8.08E-05
SLC17A9	mRNA	1.482143239	8.78E-08
hsa_circ_0116652	circRNA	1.478889832	1.14E-20
TRIM16L	mRNA	1.475417741	5.99E-05
hsa_circ_0012715	circRNA	1.472021143	2.85E-04
ALDH1L2	mRNA	1.470821435	3.46E-07
LOC105371453	lncRNA	1.470469507	6.42E-04
S1PR4	mRNA	1.470425999	3.27E-09
hsa_circ_0075636	circRNA	1.469974328	3.60E-06
TRIM16	mRNA	1.467784663	3.05E-06
hsa_circ_0085653	circRNA	1.465821911	2.02E-04
SLC29A2	mRNA	1.462820527	6.73E-07
hsa_circ_0118845	circRNA	1.461963678	2.32E-16
RAPH1	mRNA	1.45806836	4.05E-18
CA13	mRNA	1.456361906	1.67E-04
TUBB3	mRNA	1.453457783	5.03E-06
HS3ST1	mRNA	1.449497704	0.004169892
GPAT3	mRNA	1.44847388	2.70E-06
PDE2A	mRNA	1.446369976	3.01E-20
CYP27C1	mRNA	1.443058432	0.045702171
NSDHL	mRNA	1.443006493	3.10E-13
hsa_circ_0022972	circRNA	1.442247288	9.59E-14
hsa_circ_0085646	circRNA	1.441902118	6.12E-12
BIRC3	mRNA	1.441782091	0.004888155
OAF	mRNA	1.439942952	2.22E-05

hsa_circ_0061480	circRNA	1.437505499	3.15E-04
hsa_circ_0043728	circRNA	1.430249302	1.52E-13
hsa_circ_0009733	circRNA	1.425812749	1.64E-04
hsa_circ_0092111	circRNA	1.425248935	0.004835736
hsa_circ_0058364	circRNA	1.425038094	4.93E-12
GCLM	mRNA	1.421678271	2.09E-05
hsa_circ_0092127	circRNA	1.421167171	5.14E-04
LOC101928465	lncRNA	1.419207584	7.43E-04
ANK2	mRNA	1.4187308	0.003000726
LOC105378726	lncRNA	1.415536141	1.30E-04
SLC16A13	mRNA	1.412860108	5.22E-05
hsa_circ_0061144	circRNA	1.411017485	2.94E-05
LOC105374732	lncRNA	1.410595566	0.035428066
PDZD4	mRNA	1.410552357	0.032786776
CDR2L	mRNA	1.409943617	4.13E-10
MAFG-DT	lncRNA	1.408924216	4.15E-08
HYAL2	mRNA	1.404104413	2.06E-08
hsa_circ_0136155	circRNA	1.403429062	4.79E-05
hsa_circ_0016295	circRNA	1.402477647	0.007498735
hsa_circ_0124463	circRNA	1.401365133	6.36E-04
DUSP5	mRNA	1.397199826	2.80E-05
GNAZ	mRNA	1.396277155	0.00145888
PYCR1	mRNA	1.394420657	3.15E-10
hsa_circ_0088341	circRNA	1.394187505	2.26E-06
hsa_circ_0029581	circRNA	1.392426466	1.18E-05

PCDHGC3	mRNA	1.391962811	6.01E-11
STARD4	mRNA	1.390489507	3.48E-14
hsa_circ_0013221	circRNA	1.386388126	6.75E-04
hsa_circ_0091727	circRNA	1.385705521	2.39E-11
hsa_circ_0088340	circRNA	1.38215892	4.53E-06
GPC1	mRNA	1.381815437	1.06E-05
hsa_circ_0019511	circRNA	1.379309863	0.015442499
DUSP10	mRNA	1.378063783	0.004305687
NPPA-AS1	lncRNA	1.376813056	0.009152458
hsa_circ_0043260	circRNA	1.374316542	7.10E-06
SC5D	mRNA	1.374042425	1.40E-20
AKAP12	mRNA	1.373678609	8.34E-05
TMEM171	mRNA	1.372033262	2.83E-05
hsa_circ_0043726	circRNA	1.371394257	2.09E-05
hsa_circ_0085655	circRNA	1.370368015	0.004113085
BGIG9606_49374	lncRNA	1.369208227	0.009569867
hsa_circ_0083855	circRNA	1.368155232	0.004793928
LOC107986178	lncRNA	1.36734076	1.52E-07
LOC102724580	pseudo	1.366895482	0.001245267
LINC02435	lncRNA	1.366596822	0.01883371
LOC105376205	lncRNA	1.36594704	0.045244276
hsa_circ_0005613	circRNA	1.36486932	5.66E-06
hsa_circ_0005714	circRNA	1.364788479	0.009402221
EBP	mRNA	1.36173489	1.46E-06
hsa_circ_0116655	circRNA	1.357367097	0.001707158

hsa_circ_0070752	circRNA	1.352990581	3.75E-12
PCYT2	mRNA	1.352014309	3.06E-09
APOL4	mRNA	1.351798183	0.00123917
hsa_circ_0124083	circRNA	1.351279631	1.78E-11
LOC105378526	lncRNA	1.351166312	9.71E-05
CDKN2B	mRNA	1.350621802	0.011166975
FAM3D	mRNA	1.350552945	0.030487421
BGIG9606_50266	lncRNA	1.348390008	0.047464597
APOB	mRNA	1.347722827	0.019921683
BGIG9606_55956	lncRNA	1.347401566	0.024197278
hsa_circ_0089374	circRNA	1.345490383	0.001318741
hsa_circ_0051428	circRNA	1.344645066	5.25E-04
PFKFB4	mRNA	1.343853117	4.62E-07
CDKN1A	mRNA	1.341175611	4.79E-06
hsa_circ_0118844	circRNA	1.340905052	2.30E-15
GRAMD2B	mRNA	1.339128085	3.90E-13
hsa_circ_0094049	circRNA	1.33899251	0.032029771
SLCO2A1	mRNA	1.338354552	0.008226885
CTSL	mRNA	1.334899281	3.96E-04
hsa_circ_0044645	circRNA	1.33463329	9.84E-06
hsa_circ_0029565	circRNA	1.333834401	0.013235405
NES	mRNA	1.333231211	3.38E-04
LINC02454	lncRNA	1.328082236	2.86E-11
hsa_circ_0090938	circRNA	1.327889939	4.29E-10
hsa_circ_0022965	circRNA	1.327828339	4.88E-16

hsa_circ_0043709	circRNA	1.326589722	0.007239942
hsa_circ_0023448	circRNA	1.324086883	1.17E-07
PHLDA2	mRNA	1.32346952	1.44E-04
hsa_circ_0068315	circRNA	1.322357202	0.01852778
TRNP1	mRNA	1.322255896	7.68E-05
MMP24	mRNA	1.32147258	1.15E-04
OAS1	mRNA	1.317672302	1.25E-05
hsa_circ_0097447	circRNA	1.316106745	0.001576361
hsa_circ_0045566	circRNA	1.315445199	8.12E-05
hsa_circ_0114538	circRNA	1.315113483	0.001595656
ANKRD44	mRNA	1.313155073	0.005615439
LOC102724740	lncRNA	1.313079917	1.22E-04
hsa_circ_0009732	circRNA	1.311652241	9.03E-04
KCNK1	mRNA	1.307197602	0.008647833
SLC16A9	mRNA	1.306575845	0.045790785
hsa_circ_0106839	circRNA	1.306143669	6.04E-06
DHH	mRNA	1.302426189	0.009088581
HES6	mRNA	1.300745313	0.00108395
hsa_circ_0013222	circRNA	1.29998886	0.003438284
hsa_circ_0138796	circRNA	1.299948893	0.00436705
ARHGDIG	mRNA	1.298069539	0.038964917
PANX2	mRNA	1.297667558	9.16E-05
ARL4C	mRNA	1.29711189	0.001531369
PLAUR	mRNA	1.296105977	8.26E-04
hsa_circ_0075323	circRNA	1.295290985	0.009165987

hsa_circ_0014421	circRNA	1.294918906	0.007582773
APOL3	mRNA	1.292091025	3.52E-19
YIF1A	mRNA	1.289768443	7.42E-14
hsa_circ_0003987	circRNA	1.288746298	0.022921154
hsa_circ_0106159	circRNA	1.288027643	4.07E-07
ITGA10	mRNA	1.287268105	0.002421727
PLPP3	mRNA	1.283508012	7.01E-06
EEF1AKMT4	mRNA	1.278146089	2.19E-06
RNF122	mRNA	1.276609251	0.011342335
hsa_circ_0106155	circRNA	1.275275967	3.07E-05
hsa_circ_0128328	circRNA	1.272795856	5.14E-04
SLC6A6	mRNA	1.271528349	0.002196855
hsa_circ_0118840	circRNA	1.270736474	0.002109955
HSPA1B	mRNA	1.269366676	1.67E-05
hsa_circ_0009724	circRNA	1.268983817	0.0351108
hsa_circ_0069770	circRNA	1.268536649	0.022357767
SPRY4	mRNA	1.26461702	5.52E-10
hsa_circ_0044220	circRNA	1.264025418	1.21E-05
IL10RA	mRNA	1.262030684	5.21E-04
TNFRSF11B	mRNA	1.261751888	0.034738642
hsa_circ_0045577	circRNA	1.259643412	9.25E-08
PGD	mRNA	1.259634102	5.60E-05
SH3BP5	mRNA	1.258258012	9.42E-08
RCN3	mRNA	1.256548876	2.72E-07
C11orf24	mRNA	1.256495402	5.72E-08

SPRY1	mRNA	1.256145347	1.37E-19
PAQR4	mRNA	1.255482549	9.04E-05
LOC100507564	lncRNA	1.253860682	5.39E-06
hsa_circ_0051351	circRNA	1.253095594	0.029231486
hsa_circ_0009735	circRNA	1.250478285	0.023672721
HID1-AS1	lncRNA	1.246548289	0.005524341
HYAL3	mRNA	1.246475202	1.39E-13
TFEC	mRNA	1.245833761	0.03609475
KLHL35	mRNA	1.244513768	0.001345424
hsa_circ_0051352	circRNA	1.24076603	0.034139165
TNFRSF10A	mRNA	1.239665216	5.92E-05
hsa_circ_0091212	circRNA	1.237560963	0.047236964
FXYD6-FXYD2	mRNA	1.237294556	0.034969622
hsa_circ_0003090	circRNA	1.235967948	1.64E-04
ADCY7	mRNA	1.23547155	4.96E-10
hsa_circ_0043722	circRNA	1.234875548	1.00E-07
hsa_circ_0118843	circRNA	1.233953879	1.07E-11
hsa_circ_0046273	circRNA	1.230743183	1.19E-09
hsa_circ_0129926	circRNA	1.230551897	0.007253908
SLC2A6	mRNA	1.230036006	1.63E-07
hsa_circ_0138691	circRNA	1.227785259	1.66E-05
hsa_circ_0071726	circRNA	1.226247936	1.82E-04
hsa_circ_0075322	circRNA	1.226136676	0.012056832
ME1	mRNA	1.225724442	9.68E-05
SLC41A2	mRNA	1.225282961	3.71E-09

hsa_circ_0043735	circRNA	1.224815741	0.036909429
SNAI1	mRNA	1.223771554	1.84E-04
hsa_circ_0018636	circRNA	1.222825597	0.005391349
hsa_circ_0066176	circRNA	1.221428233	0.005990161
hsa_circ_0006078	circRNA	1.220484083	2.63E-07
EFNB1	mRNA	1.220467386	1.51E-05
hsa_circ_0110931	circRNA	1.219872303	2.05E-05
hsa_circ_0003513	circRNA	1.21878731	0.03687346
hsa_circ_0005129	circRNA	1.218557485	2.24E-05
hsa_circ_0136327	circRNA	1.214582264	0.007780652
hsa_circ_0043730	circRNA	1.213801532	0.018409932
hsa_circ_0124342	circRNA	1.21119518	1.73E-08
hsa_circ_0140515	circRNA	1.210512784	6.44E-05
FRRS1	mRNA	1.210194172	0.047779299
hsa_circ_0022966	circRNA	1.210010666	5.17E-14
SQSTM1	mRNA	1.209427997	3.39E-04
hsa_circ_0075321	circRNA	1.208771735	0.005842615
hsa_circ_0066259	circRNA	1.207404421	1.86E-08
hsa_circ_0066579	circRNA	1.206706827	1.31E-06
LPCAT1	mRNA	1.206290798	8.29E-09
hsa_circ_0106699	circRNA	1.205454254	0.005998025
hsa_circ_0008610	circRNA	1.204175645	1.25E-05
RASA4DP	pseudo	1.203210535	0.001591439
hsa_circ_0061145	circRNA	1.203156646	0.001753632
AACS	mRNA	1.202544037	5.71E-12

SNAI3-AS1	lncRNA	1.201925683	2.67E-18
MFSD6	mRNA	1.200835351	1.25E-04
hsa_circ_0092081	circRNA	1.200372385	0.001108113
hsa_circ_0058476	circRNA	1.199562748	6.35E-07
hsa_circ_0077210	circRNA	1.198041968	0.009279338
hsa_circ_0002997	circRNA	1.191643449	2.96E-07
hsa_circ_0042525	circRNA	1.190885862	8.56E-05
TMEM217	mRNA	1.189782006	8.26E-04
hsa_circ_0069434	circRNA	1.189216617	3.36E-11
hsa_circ_0009728	circRNA	1.189118174	0.038497412
hsa_circ_0115809	circRNA	1.184735852	0.003385094
hsa_circ_0032281	circRNA	1.183454758	5.80E-04
hsa_circ_0013891	circRNA	1.183173071	0.031150679
hsa_circ_0078299	circRNA	1.182659813	0.001975964
hsa_circ_0083854	circRNA	1.182279496	0.023862771
hsa_circ_0075318	circRNA	1.181207905	0.024588703
hsa_circ_0087008	circRNA	1.181047201	1.66E-04
hsa_circ_0051740	circRNA	1.180867912	0.021128417
hsa_circ_0014538	circRNA	1.18000163	2.28E-04
hsa_circ_0029383	circRNA	1.179298067	4.28E-07
hsa_circ_0129539	circRNA	1.179238323	3.35E-04
ALDOC	mRNA	1.178314934	8.96E-06
hsa_circ_0131624	circRNA	1.177627073	1.76E-07
hsa_circ_0019712	circRNA	1.175644024	6.17E-04
hsa_circ_0043725	circRNA	1.173197591	0.009418207

DCST1-AS1	lncRNA	1.170647333	0.002879702
hsa_circ_0101181	circRNA	1.170604427	0.010318986
hsa_circ_0077014	circRNA	1.168928748	0.032865795
hsa_circ_0049435	circRNA	1.168545156	7.34E-04
ADCY1	mRNA	1.168484702	0.016998262
hsa_circ_0106689	circRNA	1.168478347	2.03E-05
hsa_circ_0106156	circRNA	1.168381915	3.12E-04
hsa_circ_0073995	circRNA	1.166941247	0.014340248
hsa_circ_0119233	circRNA	1.166787217	6.79E-07
hsa_circ_0090939	circRNA	1.164287049	0.011256036
hsa_circ_0073773	circRNA	1.162537044	0.036955937
hsa_circ_0043710	circRNA	1.162351877	0.002222171
MYC	mRNA	1.160131316	4.58E-08
hsa_circ_0138032	circRNA	1.158942859	0.001830131
EMILIN2	mRNA	1.158787373	7.16E-06
FAM114A1	mRNA	1.155437882	1.89E-13
TLCD1	mRNA	1.155122151	3.42E-04
CREB5	mRNA	1.15297704	0.030027363
hsa_circ_0006253	circRNA	1.152857487	3.70E-04
hsa_circ_0025419	circRNA	1.151371499	0.001213412
hsa_circ_0067254	circRNA	1.150488322	1.59E-06
TUBB2A	mRNA	1.150400064	3.63E-07
hsa_circ_0043729	circRNA	1.150252715	1.53E-14
hsa_circ_0106124	circRNA	1.149420557	0.022086721
hsa_circ_0007751	circRNA	1.148712474	0.01491131

CXCL8	mRNA	1.148171265	0.011968959
hsa_circ_0118839	circRNA	1.147092445	0.027219599
hsa_circ_0066584	circRNA	1.146695037	0.010728753
PLPP1	mRNA	1.146279391	4.47E-11
BCL2	mRNA	1.145247272	0.004111435
hsa_circ_0071730	circRNA	1.145146615	0.003107119
hsa_circ_0049424	circRNA	1.144891485	0.009814244
hsa_circ_0043744	circRNA	1.144105466	1.86E-13
hsa_circ_0106837	circRNA	1.143581973	9.02E-05
SLC25A1	mRNA	1.143010264	1.62E-08
NENF	mRNA	1.142957212	6.69E-05
hsa_circ_0043707	circRNA	1.142413326	2.87E-08
IL18BP	mRNA	1.142369555	1.41E-07
GBE1	mRNA	1.142233757	3.11E-04
hsa_circ_0031127	circRNA	1.141905961	1.17E-07
hsa_circ_0013175	circRNA	1.14176369	0.041671584
LOC101927825	lncRNA	1.140357218	4.14E-04
CGREF1	mRNA	1.138750883	0.031477313
hsa_circ_0066257	circRNA	1.137717529	0.00201697
SYT1	mRNA	1.137238062	0.044903641
hsa_circ_0003267	circRNA	1.136603257	0.016095614
hsa_circ_0097927	circRNA	1.136298124	1.25E-08
ZMYND15	mRNA	1.136241866	0.005203441
hsa_circ_0132273	circRNA	1.133697031	2.79E-09
hsa_circ_0001092	circRNA	1.133477433	0.034870941

ARHGAP27	mRNA	1.132999682	1.51E-05
hsa_circ_0097926	circRNA	1.132815716	1.97E-08
hsa_circ_0066258	circRNA	1.132247314	8.48E-04
CMPK2	mRNA	1.131199696	0.018628046
hsa_circ_0067255	circRNA	1.130483742	1.22E-04
hsa_circ_0097841	circRNA	1.12900606	1.11E-05
GPR3	mRNA	1.12684005	8.50E-07
hsa_circ_0074137	circRNA	1.126711284	0.024550838
hsa_circ_0131040	circRNA	1.126130051	3.03E-06
hsa_circ_0118841	circRNA	1.126026955	0.028238148
ANKRD20A1	mRNA	1.12585112	0.016018377
hsa_circ_0029374	circRNA	1.125797565	8.58E-09
hsa_circ_0027610	circRNA	1.125697897	0.038710036
hsa_circ_0005864	circRNA	1.125174714	0.001714066
hsa_circ_0038055	circRNA	1.123539311	0.001408146
hsa_circ_0088698	circRNA	1.120415228	2.75E-04
SLC25A37	mRNA	1.120051898	2.87E-05
hsa_circ_0127818	circRNA	1.11996418	1.50E-05
hsa_circ_0002994	circRNA	1.119900698	3.66E-14
hsa_circ_0115402	circRNA	1.119772317	4.35E-09
hsa_circ_0139847	circRNA	1.119298295	0.023441384
hsa_circ_0127831	circRNA	1.118576742	4.64E-04
hsa_circ_0136320	circRNA	1.118219922	0.005646299
NKX3-1	mRNA	1.117912153	0.005916587
hsa_circ_0043713	circRNA	1.117815575	2.08E-11

SOD2-OT1	lncRNA	1.116277322	0.022439881
DOCK5	mRNA	1.116044573	0.043050154
EMP3	mRNA	1.114897382	1.34E-06
hsa_circ_0106160	circRNA	1.113697991	8.16E-05
hsa_circ_0043720	circRNA	1.113571112	1.17E-07
hsa_circ_0069436	circRNA	1.113434616	0.003736176
OAS3	mRNA	1.112374835	2.90E-07
SERPINE2	mRNA	1.112179669	6.44E-07
hsa_circ_0069435	circRNA	1.111900477	4.82E-07
FAM107A	mRNA	1.111848164	2.13E-05
CATSPERG	mRNA	1.111360651	0.018680834
FXYD6	mRNA	1.111349724	3.02E-06
LOC107986558	lncRNA	1.110985232	0.007328162
hsa_circ_0066589	circRNA	1.109106711	0.014097793
hsa_circ_0110848	circRNA	1.108980206	0.001864437
TAF13	mRNA	1.10819086	5.25E-08
hsa_circ_0014887	circRNA	1.108002628	0.025345129
CD55	mRNA	1.107772296	3.55E-06
hsa_circ_0066586	circRNA	1.107734365	0.031014981
BGIG9606_46452	mRNA	1.107632291	0.034941534
hsa_circ_0110671	circRNA	1.107621928	0.008815393
ACLY	mRNA	1.106405083	9.41E-10
CASKIN2	mRNA	1.105658126	5.50E-05
hsa_circ_0050021	circRNA	1.104641723	0.01670908
IRAK2	mRNA	1.103125241	0.004739501

hsa_circ_0058350	circRNA	1.102691266	0.009447966
hsa_circ_0106693	circRNA	1.102415134	2.21E-06
hsa_circ_0066585	circRNA	1.102268697	0.015279255
MEF2A	mRNA	1.102056834	5.74E-04
hsa_circ_0077208	circRNA	1.101767597	0.001777335
hsa_circ_0028772	circRNA	1.101181545	0.003818917
SLC12A2	mRNA	1.101117868	8.07E-04
hsa_circ_0043737	circRNA	1.099964014	4.33E-09
CKB	mRNA	1.098862602	0.001952674
hsa_circ_0047177	circRNA	1.098559259	3.48E-04
hsa_circ_0136324	circRNA	1.097467876	0.007374376
hsa_circ_0043702	circRNA	1.097127173	0.001491688
hsa_circ_0007438	circRNA	1.096025505	2.56E-08
EXOC3L1	mRNA	1.095536343	0.00396687
hsa_circ_0043741	circRNA	1.094950536	2.13E-10
hsa_circ_0042561	circRNA	1.09306543	0.022288072
NFKB2	mRNA	1.09290815	2.21E-11
hsa_circ_0049427	circRNA	1.092595143	0.022105883
hsa_circ_0120168	circRNA	1.089475609	0.005296461
PALD1	mRNA	1.088054423	3.78E-04
hsa_circ_0128607	circRNA	1.087886541	7.05E-05
IER5	mRNA	1.087065065	9.82E-05
PNP	mRNA	1.086805747	8.86E-05
hsa_circ_0043731	circRNA	1.085971653	1.19E-04
CLDN15	mRNA	1.085808892	0.009946944

hsa_circ_0052677	circRNA	1.084785733	0.003699113
hsa_circ_0133005	circRNA	1.084743556	1.37E-04
PIM1	mRNA	1.084651736	6.08E-06
hsa_circ_0130811	circRNA	1.084641675	0.02955537
TMEM189	mRNA	1.084503216	1.53E-09
FUT1	mRNA	1.084294738	4.84E-11
hsa_circ_0088694	circRNA	1.084148373	2.64E-04
GSR	mRNA	1.083596851	8.38E-05
FLRT3	mRNA	1.082603228	0.049014973
STMN3	mRNA	1.081416416	0.04225039
hsa_circ_0001000	circRNA	1.079431881	0.016264508
ARF4	mRNA	1.077697408	1.16E-06
NCALD	mRNA	1.077106278	0.009568072
EPS8L2	mRNA	1.076891596	0.009256135
LOC100130283	lncRNA	1.07685182	0.013039288
PLAT	mRNA	1.075770482	0.014231466
TTC7A	mRNA	1.075589347	4.30E-06
hsa_circ_0032099	circRNA	1.07558074	0.001404631
hsa_circ_0048491	circRNA	1.075219654	1.10E-04
SLC17A5	mRNA	1.074464142	8.33E-15
hsa_circ_0014420	circRNA	1.074211329	0.015442499
hsa_circ_0085533	circRNA	1.07409531	0.013850162
hsa_circ_0106162	circRNA	1.073734405	0.001116536
hsa_circ_0110291	circRNA	1.073316183	3.56E-05
TBC1D8	mRNA	1.073148433	2.01E-06

hsa_circ_0047166	circRNA	1.071109744	2.39E-05
hsa_circ_0084097	circRNA	1.070348021	0.022216785
BGIG9606_54991	mRNA	1.066244746	3.51E-06
hsa_circ_0095107	circRNA	1.06554087	0.009428627
hsa_circ_0008107	circRNA	1.065194743	1.23E-09
SEMA6A	mRNA	1.064428636	7.68E-05
hsa_circ_0124717	circRNA	1.064176733	0.018266632
KLF8	mRNA	1.062427163	1.15E-04
hsa_circ_0042027	circRNA	1.062314835	1.60E-06
C9orf139	mRNA	1.061525731	0.014165716
hsa_circ_0083865	circRNA	1.059195359	2.22E-07
SPTBN5	mRNA	1.059081827	7.96E-06
hsa_circ_0107057	circRNA	1.058441097	0.011631338
hsa_circ_0081895	circRNA	1.05837209	0.044076412
hsa_circ_0044638	circRNA	1.058051124	8.23E-06
hsa_circ_0136321	circRNA	1.057787508	0.043187929
hsa_circ_0051847	circRNA	1.056749616	0.012841128
PPP1R14A	mRNA	1.056624093	0.039874547
NEDD9	mRNA	1.056472298	2.39E-09
hsa_circ_0062210	circRNA	1.055046741	2.17E-09
hsa_circ_0057750	circRNA	1.054991054	0.010218843
TRIB3	mRNA	1.054927114	0.001153321
PMAIP1	mRNA	1.054679627	0.039409006
hsa_circ_0073775	circRNA	1.054482868	4.36E-05
hsa_circ_0107066	circRNA	1.052981669	2.02E-05

hsa_circ_0088689	circRNA	1.052897307	4.89E-07
TLR6	mRNA	1.052595395	2.57E-05
hsa_circ_0047185	circRNA	1.051532945	4.83E-07
UCHL1	mRNA	1.050950661	2.06E-05
L3HYPDH	mRNA	1.050185929	0.001275163
hsa_circ_0043266	circRNA	1.050126898	5.14E-04
hsa_circ_0129931	circRNA	1.048215719	1.31E-07
KIAA1614	mRNA	1.047985034	0.006070617
hsa_circ_0014224	circRNA	1.047688831	3.06E-08
hsa_circ_0132275	circRNA	1.045918291	5.21E-07
BGIG9606_51708	lncRNA	1.045851399	3.12E-06
hsa_circ_0009049	circRNA	1.04550276	0.039945557
hsa_circ_0088763	circRNA	1.044955417	5.14E-04
EVA1C	mRNA	1.044713059	4.30E-06
hsa_circ_0101786	circRNA	1.044295383	0.028790513
hsa_circ_0014537	circRNA	1.044150338	2.46E-12
RUSC2	mRNA	1.043573233	1.45E-06
NPC1	mRNA	1.04129301	8.33E-14
hsa_circ_0037056	circRNA	1.040769597	6.66E-05
hsa_circ_0136323	circRNA	1.040671726	0.042559107
hsa_circ_0042123	circRNA	1.040424054	0.022495889
RADIL	mRNA	1.040329796	0.045358661
hsa_circ_0029170	circRNA	1.039372859	0.048667815
hsa_circ_0005595	circRNA	1.039209564	3.17E-06
KANK3	mRNA	1.039000213	2.08E-05

BGIG9606_52716	lncRNA	1.038949162	0.037215594
hsa_circ_0106688	circRNA	1.038128623	0.002732866
hsa_circ_0013559	circRNA	1.037282241	1.52E-07
hsa_circ_0077217	circRNA	1.036137442	1.67E-04
CD93	mRNA	1.035443619	3.17E-07
LOC101927472	lncRNA	1.033894452	0.00357303
hsa_circ_0075320	circRNA	1.03235819	0.025588992
hsa_circ_0014539	circRNA	1.029443362	0.027586074
hsa_circ_0053098	circRNA	1.029333664	2.03E-08
hsa_circ_0005614	circRNA	1.029110975	0.005592985
hsa_circ_0066177	circRNA	1.029070174	0.014374262
hsa_circ_0003522	circRNA	1.028883462	0.003158059
AK8	mRNA	1.028428015	0.023807426
hsa_circ_0022990	circRNA	1.028116748	0.032185139
GPR85	mRNA	1.028053417	0.004044952
MICA	mRNA	1.027592466	2.23E-05
SLC44A4	mRNA	1.027469748	0.034385819
ZFP36	mRNA	1.026841876	6.17E-14
hsa_circ_0075324	circRNA	1.026121943	0.0480124
ACTL10	mRNA	1.025904777	5.96E-04
hsa_circ_0077009	circRNA	1.024800785	9.89E-09
ABCB6	mRNA	1.022616293	3.19E-06
hsa_circ_0080169	circRNA	1.02252059	0.041032602
hsa_circ_0088695	circRNA	1.022053903	0.001201066
hsa_circ_0051358	circRNA	1.020694121	0.03313518

hsa_circ_0037398	circRNA	1.019984787	8.81E-04
BGIG9606_41392	lncRNA	1.019943477	0.003330702
LINC01537	lncRNA	1.019074688	6.16E-05
hsa_circ_0013558	circRNA	1.01803778	0.03878819
hsa_circ_0060832	circRNA	1.017582927	5.50E-07
hsa_circ_0037057	circRNA	1.017191456	5.86E-05
hsa_circ_0131036	circRNA	1.017131737	0.04148127
hsa_circ_0068616	circRNA	1.017105961	0.006573202
hsa_circ_0083858	circRNA	1.016314523	0.002370637
TMEM104	mRNA	1.016139529	9.83E-11
LINC00941	lncRNA	1.015996161	1.90E-04
hsa_circ_0016292	circRNA	1.015861184	7.52E-06
hsa_circ_0042524	circRNA	1.015749294	0.007108325
hsa_circ_0028971	circRNA	1.015251206	0.026485773
CDC42EP2	mRNA	1.015248518	2.04E-08
LOC105377663	lncRNA	1.013325954	2.45E-05
SEC24D	mRNA	1.012840272	9.84E-16
TBC1D2	mRNA	1.012126541	9.50E-07
hsa_circ_0045572	circRNA	1.011206677	1.21E-04
hsa_circ_0069575	circRNA	1.010703519	6.64E-08
hsa_circ_0098320	circRNA	1.010231343	0.007715395
hsa_circ_0081343	circRNA	1.00819114	9.25E-08
hsa_circ_0029569	circRNA	1.008168241	0.006837799
SPAG9	mRNA	1.007141318	5.93E-06
JUND	mRNA	1.006316689	4.93E-08

hsa_circ_0058455	circRNA	1.005948577	8.08E-04
hsa_circ_0134374	circRNA	1.005743748	0.032844494
CORO6	mRNA	1.005582562	0.020317805
hsa_circ_0020261	circRNA	1.005485971	0.036372042
hsa_circ_0042776	circRNA	1.003773058	2.32E-05
NUMBL	mRNA	1.003706874	1.42E-21
PMP22	mRNA	1.003323879	1.62E-06
CCM2L	mRNA	1.00325004	2.33E-10
hsa_circ_0007202	circRNA	1.002895828	0.041032602
hsa_circ_0093032	circRNA	1.002587874	0.021849267
PTP4A3	mRNA	1.002225943	0.004963257
TLNRD1	mRNA	1.001734675	2.23E-05
GOLGA3	mRNA	1.000769075	3.20E-10
hsa_circ_0078265	circRNA	1.000442262	0.022741176
hsa_circ_0107056	circRNA	1.000356989	6.34E-05
hsa_circ_0067252	circRNA	1.000178705	1.11E-05
ATP8B2	mRNA	0.999586655	1.06E-06
FAM167B	mRNA	0.999051982	1.39E-05
FAM177A1	mRNA	0.998689182	2.16E-06
TDRKH	mRNA	0.998588762	1.36E-11
hsa_circ_0118836	circRNA	0.997524301	2.07E-04
hsa_circ_0042560	circRNA	0.997254163	0.029460688
hsa_circ_0106838	circRNA	0.996289666	1.94E-08
hsa_circ_0083862	circRNA	0.995945005	1.22E-06
SLC1A1	mRNA	0.995002664	2.55E-04

CXCL16	mRNA	0.994756236	0.007858358
MCEE	mRNA	0.992739702	3.97E-07
hsa_circ_0039151	circRNA	0.99131498	0.037448212
hsa_circ_0132282	circRNA	0.99071264	1.87E-09
hsa_circ_0001974	circRNA	0.99014757	0.014681191
hsa_circ_0097098	circRNA	0.990146242	0.010835264
FDPS	mRNA	0.989358031	7.45E-09
hsa_circ_0009865	circRNA	0.989286261	0.036652909
hsa_circ_0013560	circRNA	0.989190636	0.024800195
GIT1	mRNA	0.988994709	1.38E-06
hsa_circ_0058353	circRNA	0.988105891	0.010176704
hsa_circ_0007281	circRNA	0.987576554	3.53E-12
hsa_circ_0121705	circRNA	0.987463768	0.022101168
hsa_circ_0138795	circRNA	0.987106882	0.0038962
hsa_circ_0083863	circRNA	0.98696569	1.58E-05
hsa_circ_0127082	circRNA	0.98690687	1.46E-06
hsa_circ_0083861	circRNA	0.986581596	0.020457766
hsa_circ_0088692	circRNA	0.986085179	3.42E-04
hsa_circ_0001216	circRNA	0.984887052	0.011743013
TMEM45A	mRNA	0.984841313	3.75E-11
STXBP1	mRNA	0.984765451	1.12E-12
NRCAM	mRNA	0.984150048	0.007854066
hsa_circ_0126362	circRNA	0.983764714	4.30E-10
LOC105369340	lncRNA	0.983322125	4.71E-04
hsa_circ_0060833	circRNA	0.983286625	0.024664218

hsa_circ_0051742	circRNA	0.983041698	0.003402305
hsa_circ_0014225	circRNA	0.981905041	5.86E-07
BHLHE40	mRNA	0.981294768	0.007432594
KCNN3	mRNA	0.980730873	0.012279772
hsa_circ_0081349	circRNA	0.980107797	1.26E-16
LOC105379443	pseudo	0.980002617	0.015603411
hsa_circ_0045497	circRNA	0.979790701	7.71E-08
hsa_circ_0136223	circRNA	0.979420718	3.27E-08
PODXL	mRNA	0.979313141	0.012283924
hsa_circ_0073992	circRNA	0.978529052	9.02E-05
hsa_circ_0102479	circRNA	0.978500243	6.03E-06
MARC1	mRNA	0.977600804	0.037084333
hsa_circ_0032800	circRNA	0.977363822	0.018964164
hsa_circ_0020695	circRNA	0.977131325	0.024484738
hsa_circ_0137954	circRNA	0.976479452	0.015428665
ARID5A	mRNA	0.976141677	0.005228332
CAVIN3	mRNA	0.976133164	3.77E-10
CFAP157	mRNA	0.976125567	0.033505673
hsa_circ_0019721	circRNA	0.975961805	3.16E-08
hsa_circ_0044644	circRNA	0.975489651	2.39E-05
hsa_circ_0050173	circRNA	0.974787176	0.040305852
hsa_circ_0009738	circRNA	0.974779455	0.002650549
hsa_circ_0045498	circRNA	0.974498291	2.53E-09
hsa_circ_0073759	circRNA	0.974368147	0.023592469
hsa_circ_0061481	circRNA	0.974272365	7.68E-04

hsa_circ_0005105	circRNA	0.973857995	0.01189572
hsa_circ_0114130	circRNA	0.97381481	0.020847128
hsa_circ_0069572	circRNA	0.972911207	0.008595247
TM4SF18	mRNA	0.971209136	6.54E-07
CDK5R1	mRNA	0.970680191	0.030817534
S100A6	mRNA	0.97051949	7.96E-06
HSPA1A	mRNA	0.969333499	1.75E-04
MAP3K14	mRNA	0.968690191	3.49E-08
hsa_circ_0073761	circRNA	0.968081116	0.001284497
hsa_circ_0106701	circRNA	0.96798861	7.50E-06
hsa_circ_0112237	circRNA	0.967505164	6.63E-08
SCFD2	mRNA	0.965532426	2.03E-04
hsa_circ_0100691	circRNA	0.965019942	4.14E-05
hsa_circ_0019261	circRNA	0.964858406	0.00465516
hsa_circ_0002259	circRNA	0.96453859	1.37E-04
hsa_circ_0126361	circRNA	0.963670222	5.11E-07
hsa_circ_0029577	circRNA	0.963426494	0.016523348
IFFO2	mRNA	0.962344515	0.005243241
hsa_circ_0055037	circRNA	0.961042044	2.97E-07
hsa_circ_0081348	circRNA	0.960961054	0.001374039
BGIG9606_47018	mRNA	0.960334121	0.046068239
CHN2	mRNA	0.959785492	5.46E-04
hsa_circ_0000754	circRNA	0.957435702	0.001039131
hsa_circ_0064481	circRNA	0.956906997	0.017214767
hsa_circ_0069735	circRNA	0.956588777	0.004781394

hsa_circ_0042781	circRNA	0.954463932	1.89E-05
hsa_circ_0014540	circRNA	0.9543815	4.35E-09
CCDC78	mRNA	0.954182357	4.14E-04
hsa_circ_0029580	circRNA	0.95360037	1.07E-05
CLIC2	mRNA	0.952841605	5.06E-04
hsa_circ_0132274	circRNA	0.952657831	1.78E-05
CDKN2D	mRNA	0.952012321	1.02E-06
hsa_circ_0107065	circRNA	0.951845532	0.045026768
SELENOI	mRNA	0.95168094	3.69E-07
hsa_circ_0121827	circRNA	0.95074896	0.004836798
hsa_circ_0069769	circRNA	0.950032105	0.046337313
GMFG	mRNA	0.949906561	5.92E-05
hsa_circ_0106157	circRNA	0.946146025	0.003610093
hsa_circ_0070246	circRNA	0.944763957	2.53E-05
hsa_circ_0116896	circRNA	0.942900097	0.011198979
hsa_circ_0107467	circRNA	0.942492394	1.46E-04
hsa_circ_0079563	circRNA	0.941637537	4.93E-04
hsa_circ_0014677	circRNA	0.941067598	7.59E-04
ENTPD7	mRNA	0.940954308	2.52E-08
hsa_circ_0019258	circRNA	0.940777176	0.040164927
RHOC	mRNA	0.940556356	9.97E-05
hsa_circ_0061340	circRNA	0.940210133	0.002726843
B4GALT5	mRNA	0.940039989	3.24E-04
hsa_circ_0073998	circRNA	0.940009707	4.49E-05
SELENOS	mRNA	0.939851916	7.10E-11

BGIG9606_40405	lncRNA	0.938026764	0.00607777
INKA2	mRNA	0.937318233	0.048676507
hsa_circ_0044639	circRNA	0.937104735	7.43E-05
hsa_circ_0129924	circRNA	0.936315167	9.98E-06
hsa_circ_0002863	circRNA	0.935372112	2.02E-05
SMAGP	mRNA	0.934937405	3.80E-04
hsa_circ_0115414	circRNA	0.934854186	3.87E-06
PLEKHG1	mRNA	0.934376271	0.002696166
hsa_circ_0067868	circRNA	0.933879649	0.040734076
hsa_circ_0012028	circRNA	0.933787053	8.12E-04
hsa_circ_0076027	circRNA	0.933608434	0.001671101
MYLK-AS1	lncRNA	0.93340089	2.20E-05
hsa_circ_0026796	circRNA	0.932403061	3.23E-04
SLC25A22	mRNA	0.932131212	4.34E-06
hsa_circ_0069581	circRNA	0.931437601	8.01E-08
GRIN3B	mRNA	0.9309666	0.013521093
hsa_circ_0042026	circRNA	0.930901723	8.38E-07
ACAP3	mRNA	0.930855814	1.21E-06
IQCJ-SCHIP1	mRNA	0.930663159	0.006922209
hsa_circ_0042784	circRNA	0.930080171	3.83E-06
hsa_circ_0107061	circRNA	0.929777955	9.41E-05
VEGFA	mRNA	0.929714894	0.004061285
hsa_circ_0043256	circRNA	0.929305979	8.78E-06
MFSD12	mRNA	0.929179699	7.16E-06
hsa_circ_0134507	circRNA	0.928610933	3.09E-05

hsa_circ_0044636	circRNA	0.928047806	1.35E-05
hsa_circ_0071409	circRNA	0.92757801	0.00481177
hsa_circ_0070757	circRNA	0.92732617	2.45E-06
LOC105376325	lncRNA	0.926765846	7.61E-07
hsa_circ_0138791	circRNA	0.926520404	1.02E-04
hsa_circ_0114133	circRNA	0.926486458	5.11E-07
FAS	mRNA	0.926060597	0.007342674
hsa_circ_0001939	circRNA	0.925996918	0.011941083
RPS6KL1	mRNA	0.925911092	0.02509908
hsa_circ_0074397	circRNA	0.925452365	0.001773835
LLGL2	mRNA	0.925046871	0.004656267
ACACA	mRNA	0.924891947	2.89E-18
hsa_circ_0136222	circRNA	0.924315034	8.02E-04
hsa_circ_0019260	circRNA	0.922842228	4.85E-05
BCAM	mRNA	0.921812975	4.17E-07
KLF13	mRNA	0.920315763	7.02E-11
hsa_circ_0115602	circRNA	0.920024161	0.013390215
hsa_circ_0065843	circRNA	0.919766496	1.42E-13
LOC101448202	lncRNA	0.919284961	0.034038154
hsa_circ_0079074	circRNA	0.919167136	7.20E-05
BGIG9606_55579	lncRNA	0.919116634	0.002377666
RETREG1	mRNA	0.918338101	0.001217903
hsa_circ_0014423	circRNA	0.918202296	6.39E-06
PLXNA3	mRNA	0.918053792	1.99E-24
hsa_circ_0110040	circRNA	0.917680768	4.14E-06

hsa_circ_0029340	circRNA	0.916347065	3.47E-04
hsa_circ_0062913	circRNA	0.915922244	0.029627365
DMTN	mRNA	0.91572849	2.12E-05
hsa_circ_0004518	circRNA	0.915638053	4.10E-10
hsa_circ_0028958	circRNA	0.915476364	0.033399794
hsa_circ_0002168	circRNA	0.915152042	7.52E-06
hsa_circ_0110035	circRNA	0.914647623	0.005823041
hsa_circ_0070750	circRNA	0.913888681	1.30E-04
SYT17	mRNA	0.913843098	7.78E-10
EIF2AK3	mRNA	0.913575981	5.04E-10
ARHGEF15	mRNA	0.913216303	9.57E-08
hsa_circ_0132271	circRNA	0.91275208	3.50E-05
hsa_circ_0060827	circRNA	0.912235856	1.28E-06
RPGRIP1L	mRNA	0.911914957	9.45E-08
hsa_circ_0115098	circRNA	0.911579978	3.77E-04
hsa_circ_0099712	circRNA	0.911538217	0.008514798
P4HA2	mRNA	0.910814821	1.47E-11
hsa_circ_0070759	circRNA	0.910354432	8.03E-09
TKT	mRNA	0.909773291	0.005832428
HLX-AS1	lncRNA	0.909724845	0.023913629
hsa_circ_0139545	circRNA	0.909149098	1.48E-04
hsa_circ_0032280	circRNA	0.909060456	0.002392478
ST6GALNAC3	mRNA	0.908596538	8.45E-09
hsa_circ_0062211	circRNA	0.908531132	0.022357021
hsa_circ_0079636	circRNA	0.907100104	0.028790513

hsa_circ_0043230	circRNA	0.906914185	4.85E-05
PCDHGA12	mRNA	0.906458868	0.004750115
hsa_circ_0011991	circRNA	0.906324436	0.029287234
hsa_circ_0001025	circRNA	0.90577058	0.002499761
DUSP4	mRNA	0.905596028	0.04812195
hsa_circ_0090882	circRNA	0.905532356	0.002129316
hsa_circ_0045501	circRNA	0.904853059	2.91E-04
hsa_circ_0100593	circRNA	0.904472856	0.017470149
HELZ2	mRNA	0.904382451	3.81E-07
LL0XNC01-250H12.3	lncRNA	0.904269641	1.27E-05
hsa_circ_0043724	circRNA	0.904094056	2.23E-07
SLFNL1-AS1	lncRNA	0.903294569	0.017618328
KDELR2	mRNA	0.902145766	6.73E-07
hsa_circ_0076610	circRNA	0.901570889	0.008726585
GOLT1B	mRNA	0.901468859	8.28E-06
CELSR3	mRNA	0.901148189	0.001336215
hsa_circ_0008432	circRNA	0.900660369	8.63E-07
hsa_circ_0105414	circRNA	0.900457453	0.002476328
hsa_circ_0110972	circRNA	0.899382526	0.002194254
hsa_circ_0063152	circRNA	0.898993806	0.01482698
ANKRD9	mRNA	0.897892833	4.40E-05
TRIB1	mRNA	0.897234172	0.002576756
hsa_circ_0044650	circRNA	0.896837639	0.022694254
hsa_circ_0003848	circRNA	0.896264206	0.002976639
H1FOO	mRNA	0.896237431	0.006589525

RNF125	mRNA	0.89617195	0.001520287
hsa_circ_0080074	circRNA	0.894906696	0.008870434
hsa_circ_0119219	circRNA	0.894744222	1.64E-05
hsa_circ_0030562	circRNA	0.89416586	0.024296575
hsa_circ_0097925	circRNA	0.893866181	7.58E-06
hsa_circ_0013547	circRNA	0.892026258	0.013624672
hsa_circ_0006452	circRNA	0.891667799	0.032185139
hsa_circ_0084546	circRNA	0.891545555	0.021138906
hsa_circ_0074547	circRNA	0.890873492	0.017418583
PRR7	mRNA	0.890424407	0.02509908
GPR160	mRNA	0.889708695	0.043795505
hsa_circ_0055892	circRNA	0.889516023	0.022461612
hsa_circ_0129545	circRNA	0.889116858	1.51E-04
hsa_circ_0064280	circRNA	0.889047769	0.029597428
hsa_circ_0088688	circRNA	0.888688064	1.18E-05
hsa_circ_0007046	circRNA	0.888629576	8.29E-07
RELB	mRNA	0.888440298	0.001551521
BGIG9606_39888	lncRNA	0.887636457	0.00180502
METTL1	mRNA	0.887196353	5.31E-05
hsa_circ_0106158	circRNA	0.88650369	0.004577516
hsa_circ_0014676	circRNA	0.88631486	0.028956854
hsa_circ_0049860	circRNA	0.886053227	1.20E-08
hsa_circ_0067283	circRNA	0.885649836	8.80E-09
ARL4D	mRNA	0.885538441	1.70E-04
hsa_circ_0112099	circRNA	0.885534929	0.007498735

hsa_circ_0076025	circRNA	0.884861139	1.36E-04
NDEL1	mRNA	0.884404583	1.00E-10
MAP3K6	mRNA	0.884319624	2.53E-05
hsa_circ_0109642	circRNA	0.882793462	5.04E-04
DIAPH2	mRNA	0.882691847	3.78E-04
GFPT1	mRNA	0.882285572	8.35E-11
hsa_circ_0009007	circRNA	0.881014724	0.010837734
BLZF1	mRNA	0.880976977	5.32E-10
KDR	mRNA	0.879086101	3.19E-07
hsa_circ_0136329	circRNA	0.878521127	0.022950097
TMEM38A	mRNA	0.878049349	0.020738187
KLF11	mRNA	0.877556331	1.53E-04
hsa_circ_0129544	circRNA	0.877553087	4.58E-06
PRRC1	mRNA	0.877478493	1.04E-11
ITPR3	mRNA	0.877301469	0.003076417
hsa_circ_0005013	circRNA	0.877149515	8.98E-09
hsa_circ_0138036	circRNA	0.876894286	0.001453702
hsa_circ_0090885	circRNA	0.876013025	1.33E-04
hsa_circ_0036791	circRNA	0.875160575	0.020338076
hsa_circ_0002914	circRNA	0.875144956	0.008676982
GNE	mRNA	0.874882062	1.20E-23
hsa_circ_0008983	circRNA	0.874034773	2.79E-04
hsa_circ_0008341	circRNA	0.873866526	1.60E-11
hsa_circ_0056865	circRNA	0.873602458	0.004177422
hsa_circ_0129546	circRNA	0.873558701	8.44E-05

hsa_circ_0081340	circRNA	0.873473251	0.025484698
hsa_circ_0040503	circRNA	0.872364155	0.001660892
hsa_circ_0100692	circRNA	0.872249021	3.73E-04
hsa_circ_0045502	circRNA	0.871028862	2.57E-05
TBC1D8B	mRNA	0.871017129	1.49E-08
hsa_circ_0073997	circRNA	0.869614956	0.014975604
APOL6	mRNA	0.869548472	1.47E-06
hsa_circ_0054535	circRNA	0.869159824	4.44E-04
TRIM2	mRNA	0.869125075	0.010463489
hsa_circ_0127098	circRNA	0.868628699	4.03E-06
LIPG	mRNA	0.868547512	7.75E-07
hsa_circ_0004868	circRNA	0.868140152	2.51E-04
hsa_circ_0083751	circRNA	0.86798539	0.004244552
PDLIM3	mRNA	0.867126563	0.022638611
TMTC1	mRNA	0.867061463	0.029000407
hsa_circ_0136225	circRNA	0.866989915	5.32E-07
hsa_circ_0014422	circRNA	0.866963268	0.001714066
TSKU	mRNA	0.866788546	1.43E-05
hsa_circ_0127083	circRNA	0.866135037	3.03E-05
SULT1A4	mRNA	0.865170687	0.001340991
hsa_circ_0002071	circRNA	0.865082072	0.02856605
LRRC3	mRNA	0.864761761	2.21E-08
NOCT	mRNA	0.864305192	0.00667866
KLHL5	mRNA	0.862734086	0.003179779
hsa_circ_0032520	circRNA	0.862620218	3.35E-04

hsa_circ_0050974	circRNA	0.862131623	0.001476785
CALM1	mRNA	0.860244463	2.17E-10
hsa_circ_0034083	circRNA	0.860223709	0.008283971
MRAS	mRNA	0.859978891	0.002858281
hsa_circ_0106161	circRNA	0.859669002	0.015693019
UGDH	mRNA	0.859482215	8.15E-07
ADAMTS9-AS1	lncRNA	0.85909489	0.00285412
LOC107984338	lncRNA	0.858798553	0.001699381
hsa_circ_0099658	circRNA	0.858655854	0.004991829
CCND1	mRNA	0.858627762	0.010119556
hsa_circ_0070254	circRNA	0.858623912	7.04E-06
hsa_circ_0138033	circRNA	0.858083223	4.54E-05
hsa_circ_0095124	circRNA	0.857556139	0.016929527
hsa_circ_0014679	circRNA	0.857364954	2.22E-07
hsa_circ_0090894	circRNA	0.857131145	0.015427171
ZBTB47	mRNA	0.856414945	9.24E-10
ADAMTSL4	mRNA	0.855524718	0.018594778
hsa_circ_0038985	circRNA	0.855425371	0.005091733
hsa_circ_0045496	circRNA	0.855285469	3.96E-07
BGIG9606_66354	lncRNA	0.855232406	0.003907148
hsa_circ_0050587	circRNA	0.854855209	3.70E-04
hsa_circ_0102480	circRNA	0.854739483	0.00140294
LMO7	mRNA	0.854695136	0.020599882
hsa_circ_0128562	circRNA	0.85405568	0.014613435
hsa_circ_0081338	circRNA	0.853968308	8.36E-05

hsa_circ_0129538	circRNA	0.852303824	0.002823004
hsa_circ_0005192	circRNA	0.851881911	0.014143391
DOCK6	mRNA	0.851844032	1.39E-04
RHBDF2	mRNA	0.850722002	0.002131477
ARPC1B	mRNA	0.850715711	5.63E-16
hsa_circ_0008163	circRNA	0.8506771	2.56E-08
hsa_circ_0013556	circRNA	0.850649714	1.11E-06
hsa_circ_0061151	circRNA	0.849374804	2.68E-04
MAFF	mRNA	0.848931506	0.015662256
hsa_circ_0061103	circRNA	0.848400275	0.030950515
KCNK6	mRNA	0.848172507	8.77E-06
hsa_circ_0076019	circRNA	0.847614949	0.00543788
hsa_circ_0127091	circRNA	0.847519031	1.32E-04
hsa_circ_0049861	circRNA	0.84734933	1.33E-09
hsa_circ_0002357	circRNA	0.846926671	2.52E-06
hsa_circ_0116440	circRNA	0.846418863	1.66E-04
hsa_circ_0040848	circRNA	0.845800626	0.018043986
hsa_circ_0025293	circRNA	0.845352844	1.58E-05
hsa_circ_0047779	circRNA	0.845300314	0.01670908
hsa_circ_0049857	circRNA	0.844805533	9.44E-05
SH3PXD2A	mRNA	0.844670142	0.004502689
hsa_circ_0054562	circRNA	0.844440922	0.033935026
hsa_circ_0063602	circRNA	0.844068636	4.24E-04
PALM	mRNA	0.843567177	0.0016953
hsa_circ_0016291	circRNA	0.84308493	0.015843294

hsa_circ_0030187	circRNA	0.843030049	0.034098996
hsa_circ_0003716	circRNA	0.842814938	0.002783223
EHD3	mRNA	0.842204649	6.34E-04
POLR3D	mRNA	0.840142547	4.07E-08
hsa_circ_0013549	circRNA	0.839804478	8.23E-04
hsa_circ_0083214	circRNA	0.839657895	1.89E-05
IDH1	mRNA	0.839217707	1.44E-09
SLC46A1	mRNA	0.839187709	0.001764044
SPRYD7	mRNA	0.838790263	4.03E-05
hsa_circ_0136075	circRNA	0.838504293	0.011104283
LOC284454	lncRNA	0.838249047	0.009193191
GIPC3	mRNA	0.838106537	0.001993163
MAFG	mRNA	0.83734305	6.50E-04
hsa_circ_0007940	circRNA	0.837228478	3.76E-12
PPM1F	mRNA	0.837147059	9.39E-06
UFSP1	mRNA	0.836770122	0.037534028
hsa_circ_0067422	circRNA	0.836721265	0.020485242
hsa_circ_0043250	circRNA	0.836512353	5.91E-05
hsa_circ_0081339	circRNA	0.835900834	4.52E-09
hsa_circ_0121134	circRNA	0.835731069	0.007101278
hsa_circ_0129523	circRNA	0.835323697	9.51E-04
PTGER4	mRNA	0.835315516	0.002889586
hsa_circ_0107811	circRNA	0.835081154	1.05E-06
hsa_circ_0059406	circRNA	0.834161874	0.014917608
hsa_circ_0064274	circRNA	0.833626076	5.21E-10

ARL1	mRNA	0.833374752	4.27E-09
hsa_circ_0127081	circRNA	0.832848962	0.007220825
hsa_circ_0132626	circRNA	0.832421907	0.018204327
hsa_circ_0074003	circRNA	0.832011667	9.41E-05
hsa_circ_0052674	circRNA	0.83186149	0.004632907
hsa_circ_0026173	circRNA	0.830818949	8.58E-04
PMM2	mRNA	0.830537602	8.37E-07
hsa_circ_0125299	circRNA	0.830266227	0.047695988
CAMKK1	mRNA	0.829850824	0.018594778
hsa_circ_0088562	circRNA	0.829709934	0.021696929
hsa_circ_0136212	circRNA	0.829366051	0.001857811
hsa_circ_0029579	circRNA	0.829359407	0.027724654
hsa_circ_0016703	circRNA	0.828230386	9.78E-05
STN1	mRNA	0.828145162	0.003179779
hsa_circ_0004086	circRNA	0.827995589	8.61E-06
YEATS2-AS1	lncRNA	0.827855258	0.044270253
SPRYD3	mRNA	0.827041044	4.86E-12
hsa_circ_0069771	circRNA	0.825399397	1.57E-05
hsa_circ_0073754	circRNA	0.824778781	8.13E-06
SSH1	mRNA	0.823552118	3.18E-04
LOC100996660	lncRNA	0.82317504	1.91E-07
HSPB8	mRNA	0.823140251	9.21E-05
hsa_circ_0067261	circRNA	0.821890015	4.32E-05
SH3PXD2B	mRNA	0.821772447	0.001954715
LOC102724193	lncRNA	0.821682049	0.045514509

hsa_circ_0003374	circRNA	0.821397347	9.71E-05
hsa_circ_0108929	circRNA	0.821194139	0.006550969
hsa_circ_0004253	circRNA	0.82087749	6.62E-06
SLC35A2	mRNA	0.820456225	1.43E-05
SEC13	mRNA	0.819873636	1.87E-07
LMNA	mRNA	0.819738739	6.15E-04
hsa_circ_0067282	circRNA	0.819530447	4.11E-11
GOLGA2	mRNA	0.819522406	1.88E-15
MLKL	mRNA	0.819212129	6.22E-07
ARFGAP1	mRNA	0.8190769	5.32E-10
hsa_circ_0065846	circRNA	0.818754126	0.002108123
hsa_circ_0055051	circRNA	0.81811993	7.24E-05
hsa_circ_0029336	circRNA	0.818093553	1.26E-04
TCTEX1D1	mRNA	0.817692813	5.90E-04
YWHAH	mRNA	0.816834581	2.58E-05
hsa_circ_0066181	circRNA	0.8163821	2.60E-04
BGIG9606_51347	lncRNA	0.816080104	0.013949671
hsa_circ_0070998	circRNA	0.815220049	2.35E-04
MEF2C	mRNA	0.814950197	2.17E-04
MED8	mRNA	0.814927668	5.19E-08
hsa_circ_0076323	circRNA	0.81421903	4.45E-04
SLC27A3	mRNA	0.813987504	7.51E-07
PGM3	mRNA	0.813765312	1.01E-07
hsa_circ_0055883	circRNA	0.812851397	0.041070799
ACBD3	mRNA	0.812708979	3.34E-12

hsa_circ_0110037	circRNA	0.812380383	0.045499205
hsa_circ_0004682	circRNA	0.812328029	0.006553758
SCARB1	mRNA	0.812189043	3.74E-06
COPG1	mRNA	0.811943637	1.28E-09
FUS	mRNA	0.811571717	9.83E-08
WIPI1	mRNA	0.811266029	3.27E-09
hsa_circ_0090890	circRNA	0.811029291	0.012309371
JAK3	mRNA	0.811001584	0.001171346
DLGAP1-AS1	lncRNA	0.810190788	3.89E-04
GPR180	mRNA	0.809881895	1.38E-05
ACSL3	mRNA	0.809694145	1.61E-06
hsa_circ_0103280	circRNA	0.809113787	8.31E-05
SELENOM	mRNA	0.808990068	5.05E-04
SLFN11	mRNA	0.80868095	4.93E-11
hsa_circ_0081345	circRNA	0.808325032	0.035160387
SH3RF3	mRNA	0.808101689	1.72E-06
hsa_circ_0070241	circRNA	0.808022607	9.63E-05
hsa_circ_0060807	circRNA	0.807709156	0.002811776
hsa_circ_0073978	circRNA	0.807357219	5.12E-04
hsa_circ_0083983	circRNA	0.806755164	0.03687346
SLCO3A1	mRNA	0.806365942	0.009976802
MICA-AS1	lncRNA	0.806355327	0.00697635
MINDY2	mRNA	0.806211801	1.32E-14
HEG1	mRNA	0.806183089	1.50E-04
hsa_circ_0067281	circRNA	0.806123934	1.74E-04

hsa_circ_0044646	circRNA	0.805906327	3.86E-04
SCN1B	mRNA	0.804937102	3.46E-07
hsa_circ_0117005	circRNA	0.803353194	0.003413311
hsa_circ_0102842	circRNA	0.802844637	0.045958912
RDH11	mRNA	0.802184105	2.90E-07
SH2D3C	mRNA	0.802050304	0.001245267
SMCO4	mRNA	0.802026983	6.09E-06
hsa_circ_0120814	circRNA	0.800788021	1.01E-05
hsa_circ_0042844	circRNA	0.800739823	0.007524425
hsa_circ_0029887	circRNA	0.799976557	0.006298437
ELOVL1	mRNA	0.799958513	2.09E-10
FAM151B	mRNA	0.79983788	0.002073748
hsa_circ_0077832	circRNA	0.799623477	0.032024714
hsa_circ_0031707	circRNA	0.79962326	0.012984201
hsa_circ_0029421	circRNA	0.799107328	0.014164086
CCDC69	mRNA	0.798605272	8.48E-04
MSTO2P	pseudo	0.798572669	0.006320156
OSBPL3	mRNA	0.797867392	0.003979019
SPRED3	mRNA	0.797699548	1.73E-05
hsa_circ_0120818	circRNA	0.797487599	9.97E-06
hsa_circ_0088720	circRNA	0.796948426	0.018417482
hsa_circ_0053112	circRNA	0.796656528	1.27E-04
hsa_circ_0081342	circRNA	0.796131968	6.27E-09
hsa_circ_0018637	circRNA	0.796087419	6.76E-05
hsa_circ_0073976	circRNA	0.795843736	1.05E-04

hsa_circ_0076627	circRNA	0.794576109	0.030703325
hsa_circ_0127917	circRNA	0.793997052	0.01126407
CPNE8	mRNA	0.793994302	8.80E-06
hsa_circ_0004299	circRNA	0.793605494	3.98E-07
JMJD6	mRNA	0.793386826	7.61E-07
DLL1	mRNA	0.793316547	1.38E-04
hsa_circ_0062889	circRNA	0.791808913	0.042824532
TMEM184B	mRNA	0.791767725	1.55E-07
MPV17L	mRNA	0.79140192	0.022718794
SLC48A1	mRNA	0.791014416	0.001912902
hsa_circ_0003948	circRNA	0.790994337	1.72E-06
ARFGEF3	mRNA	0.790833977	3.63E-09
hsa_circ_0127092	circRNA	0.79032782	0.002534451
hsa_circ_0102840	circRNA	0.789793363	1.56E-05
hsa_circ_0031716	circRNA	0.789350594	0.00161381
hsa_circ_0090888	circRNA	0.788880548	5.66E-05
hsa_circ_0049432	circRNA	0.788615291	0.001707158
hsa_circ_0070245	circRNA	0.786323426	1.13E-09
DAGLA	mRNA	0.786316743	0.01070261
hsa_circ_0055061	circRNA	0.785862591	2.10E-04
CFAP410	mRNA	0.785414038	1.38E-06
hsa_circ_0004345	circRNA	0.785117963	1.00E-05
hsa_circ_0059948	circRNA	0.785027109	0.01677778
BGIG9606_51524	lncRNA	0.784443547	0.039888404
PITPNM2	mRNA	0.783680757	1.06E-04

hsa_circ_0082184	circRNA	0.782981921	7.12E-04
hsa_circ_0064821	circRNA	0.782851583	0.04917849
hsa_circ_0127097	circRNA	0.78206241	7.34E-04
TXN	mRNA	0.781473002	0.001673676
DNAJC25	mRNA	0.781405307	4.02E-06
FAM219A	mRNA	0.780929484	5.57E-04
hsa_circ_0067278	circRNA	0.780289981	6.96E-08
hsa_circ_0007629	circRNA	0.779516898	0.014069396
hsa_circ_0044582	circRNA	0.779219717	4.21E-05
hsa_circ_0039957	circRNA	0.778192966	0.014941229
ICAM1	mRNA	0.777756526	0.009404888
hsa_circ_0128012	circRNA	0.777219278	3.76E-05
hsa_circ_0136218	circRNA	0.777125906	0.001607515
hsa_circ_0124912	circRNA	0.777045379	0.014554396
FTH1	mRNA	0.77640812	0.006905957
hsa_circ_0054577	circRNA	0.775660591	0.048524329
BSN	mRNA	0.774940822	0.008281368
hsa_circ_0019257	circRNA	0.774509824	6.58E-04
hsa_circ_0070250	circRNA	0.774499384	2.97E-04
hsa_circ_0048507	circRNA	0.773105691	0.041671584
hsa_circ_0070249	circRNA	0.772925522	2.07E-04
hsa_circ_0068629	circRNA	0.77144635	0.006056351
hsa_circ_0112129	circRNA	0.770749698	0.033132085
MID1IP1	mRNA	0.770708443	0.002368739
hsa_circ_0079037	circRNA	0.770438348	0.019117274

hsa_circ_0031708	circRNA	0.76967712	7.25E-04
hsa_circ_0114987	circRNA	0.769580849	3.59E-04
TMEM106A	mRNA	0.769312879	0.001149992
hsa_circ_0000684	circRNA	0.768486299	0.002289291
hsa_circ_0003908	circRNA	0.766831848	4.54E-07
CLIC4	mRNA	0.766592581	4.57E-05
MX1	mRNA	0.766016029	0.023490021
hsa_circ_0049269	circRNA	0.765925034	0.009295291
hsa_circ_0037515	circRNA	0.765924481	0.00342596
hsa_circ_0068636	circRNA	0.765637264	0.018382168
TAGLN2	mRNA	0.765403263	0.002534086
hsa_circ_0119173	circRNA	0.765115612	0.044376405
hsa_circ_0101729	circRNA	0.764998051	0.001669438
LPIN1	mRNA	0.764321784	0.001280216
hsa_circ_0024577	circRNA	0.76422528	2.18E-07
hsa_circ_0039955	circRNA	0.764175961	0.001225292
hsa_circ_0031556	circRNA	0.764011001	0.022405658
hsa_circ_0121735	circRNA	0.763787577	0.00668646
LOC389641	lncRNA	0.763749905	8.94E-05
ALDH18A1	mRNA	0.763465262	8.62E-06
hsa_circ_0029335	circRNA	0.763382553	0.007598335
hsa_circ_0005773	circRNA	0.763132027	0.010490103
GPR68	mRNA	0.762128745	0.023899157
hsa_circ_0068431	circRNA	0.761775481	0.035276261
hsa_circ_0024815	circRNA	0.761713784	5.22E-05

hsa_circ_0079068	circRNA	0.761573933	9.42E-05
hsa_circ_0033579	circRNA	0.761534643	4.20E-05
SAMD14	mRNA	0.761517423	5.20E-05
hsa_circ_0049438	circRNA	0.760901655	0.001298463
CEACAM19	mRNA	0.759490407	0.00857795
ABCA3	mRNA	0.759405386	6.56E-17
hsa_circ_0006278	circRNA	0.758723498	9.89E-05
hsa_circ_0128606	circRNA	0.758573855	0.017590664
CHST11	mRNA	0.758539192	3.69E-05
hsa_circ_0039153	circRNA	0.758022259	1.08E-04
hsa_circ_0106690	circRNA	0.75792877	0.018289837
PLK3	mRNA	0.757797972	1.73E-05
LOC107986119	lncRNA	0.757304522	0.00117184
SEC24A	mRNA	0.756983861	4.23E-08
GCH1	mRNA	0.756909367	5.13E-04
hsa_circ_0025869	circRNA	0.756715955	0.038476902
CRIP2	mRNA	0.756627921	1.46E-06
hsa_circ_0028088	circRNA	0.756549864	0.040697088
SLC35G2	mRNA	0.755770063	3.31E-05
hsa_circ_0102931	circRNA	0.75472672	1.49E-05
GOLIM4	mRNA	0.753408748	4.28E-09
hsa_circ_0138040	circRNA	0.753251737	0.007981522
hsa_circ_0064085	circRNA	0.753037261	0.001111378
SRPRB	mRNA	0.752890515	1.19E-05
FAM214B	mRNA	0.752811595	6.66E-05

hsa_circ_0022417	circRNA	0.752256901	0.030415478
hsa_circ_0002671	circRNA	0.751854608	2.68E-05
CRACR2A	mRNA	0.751451239	2.13E-04
hsa_circ_0054558	circRNA	0.751310437	4.00E-04
hsa_circ_0020536	circRNA	0.751256286	0.001021989
hsa_circ_0007307	circRNA	0.751133882	0.03169328
hsa_circ_0024480	circRNA	0.750525008	0.003324172
SLC52A2	mRNA	0.750435038	1.90E-06
FAM129A	mRNA	0.749863986	0.006771665
FAM98A	mRNA	0.749646636	7.83E-07
ERLEC1	mRNA	0.749540547	9.19E-07
MTHFD2	mRNA	0.749268261	0.00117184
SRPRA	mRNA	0.748963675	5.60E-05
hsa_circ_0070242	circRNA	0.748937234	0.001070405
MUC1	mRNA	0.747939734	0.030700405
hsa_circ_0024477	circRNA	0.747703632	4.03E-07
GLUD1P3	pseudo	0.747610412	0.013501893
hsa_circ_0112295	circRNA	0.74751859	4.18E-05
NFKBIZ	mRNA	0.747141606	0.002190346
hsa_circ_0019259	circRNA	0.74666624	7.92E-04
GPR135	mRNA	0.746661757	0.00637838
hsa_circ_0067554	circRNA	0.746408316	0.03313518
LOC220729	pseudo	0.745790994	1.95E-04
hsa_circ_0128558	circRNA	0.745584105	9.80E-06
PANK3	mRNA	0.744992718	3.51E-07

hsa_circ_0069442	circRNA	0.744862765	0.001491875
ICAM3	mRNA	0.744329712	0.002629531
hsa_circ_0002018	circRNA	0.744305549	0.029825338
QPCT	mRNA	0.743916946	8.70E-04
hsa_circ_0059702	circRNA	0.743883773	4.36E-04
BAG3	mRNA	0.743407252	5.66E-06
hsa_circ_0063287	circRNA	0.743388957	1.00E-05
hsa_circ_0031724	circRNA	0.743236995	4.16E-05
RAC3	mRNA	0.743150858	7.86E-05
STIM2	mRNA	0.742623324	3.89E-04
hsa_circ_0026240	circRNA	0.742620215	0.020092096
SRA1	mRNA	0.74227553	5.46E-05
hsa_circ_0116682	circRNA	0.741924607	0.014583421
hsa_circ_0121694	circRNA	0.741859451	0.01048376
B3GAT3	mRNA	0.740858462	8.57E-04
hsa_circ_0029332	circRNA	0.740014011	5.13E-04
hsa_circ_0014202	circRNA	0.739090234	0.036474997
PPRC1	mRNA	0.738770271	1.44E-04
hsa_circ_0062448	circRNA	0.738518525	3.76E-05
hsa_circ_0083750	circRNA	0.737979568	1.12E-04
hsa_circ_0033623	circRNA	0.737641385	0.005535562
hsa_circ_0006543	circRNA	0.737558487	0.008433191
TMEM214	mRNA	0.737504615	5.37E-08
hsa_circ_0079564	circRNA	0.737254914	0.012803994
ACSL5	mRNA	0.736967793	0.001486947

DZIP1L	mRNA	0.736104057	0.014672213
hsa_circ_0049864	circRNA	0.735699721	4.35E-09
hsa_circ_0128441	circRNA	0.735598585	0.047636836
hsa_circ_0060575	circRNA	0.735492797	0.047934229
CRNDE	mRNA	0.735251806	0.033509526
P2RY1	mRNA	0.734749874	0.002105837
DBI	mRNA	0.734028851	1.05E-04
FTL	mRNA	0.733883837	0.026751008
ST3GAL1	mRNA	0.733185927	5.92E-05
hsa_circ_0067013	circRNA	0.732656297	0.008899928
FLVCR1	mRNA	0.732479425	0.025368155
CHSY3	mRNA	0.7318117	0.003418494
hsa_circ_0074542	circRNA	0.731481391	0.006385675
PTPRN2	mRNA	0.731448519	7.07E-05
hsa_circ_0004559	circRNA	0.731147082	0.045026768
hsa_circ_0029006	circRNA	0.730724227	0.046053873
hsa_circ_0139546	circRNA	0.730637932	8.39E-04
hsa_circ_0071386	circRNA	0.730450137	0.0184085
hsa_circ_0050394	circRNA	0.729495399	0.012229252
hsa_circ_0016306	circRNA	0.727947343	0.010678106
SNHG20	lncRNA	0.727789783	0.034560579
hsa_circ_0101550	circRNA	0.727591415	0.032933255
LRRC59	mRNA	0.727233165	0.001437247
hsa_circ_0005609	circRNA	0.726923107	5.76E-06
hsa_circ_0081344	circRNA	0.726155911	0.01710763

PLEKHO2	mRNA	0.725979671	2.32E-05
hsa_circ_0031651	circRNA	0.72559348	0.046964637
hsa_circ_0079075	circRNA	0.725581394	5.73E-06
hsa_circ_0133939	circRNA	0.724913072	0.020342608
hsa_circ_0063635	circRNA	0.724803226	0.012301819
hsa_circ_0051808	circRNA	0.724776846	1.17E-04
hsa_circ_0048486	circRNA	0.724443073	0.034859647
hsa_circ_0064277	circRNA	0.723514423	0.006946309
hsa_circ_0061105	circRNA	0.723198285	0.014619524
LOC100286925	lncRNA	0.722612941	0.002218247
hsa_circ_0003762	circRNA	0.721830524	0.00826626
hsa_circ_0050585	circRNA	0.721725713	1.11E-06
hsa_circ_0032763	circRNA	0.721616884	0.009775389
hsa_circ_0079320	circRNA	0.721383909	0.027618871
hsa_circ_0007129	circRNA	0.721347423	0.029828008
hsa_circ_0000058	circRNA	0.721215573	5.24E-04
hsa_circ_0014859	circRNA	0.721073516	1.01E-05
hsa_circ_0054563	circRNA	0.721055314	0.039914779
hsa_circ_0037516	circRNA	0.720894067	0.001051865
hsa_circ_0036202	circRNA	0.720874906	0.001993681
hsa_circ_0031368	circRNA	0.720669514	0.001317067
CSF2RB	mRNA	0.720145849	3.39E-07
TFRC	mRNA	0.719974444	0.00746706
hsa_circ_0067259	circRNA	0.719403945	0.003989981
PPP2R5B	mRNA	0.719394639	3.73E-04

hsa_circ_0003808	circRNA	0.719064106	8.33E-04
hsa_circ_0062875	circRNA	0.718981941	0.005931012
SLC4A5	mRNA	0.717774435	0.002541536
SLC25A45	mRNA	0.717375118	1.54E-04
SRP54	mRNA	0.717324498	1.61E-04
hsa_circ_0087501	circRNA	0.717075205	0.046740573
hsa_circ_0089922	circRNA	0.71707198	2.49E-05
MAP1B	mRNA	0.716811964	0.001892441
hsa_circ_0023882	circRNA	0.716152276	0.025472333
MCAM	mRNA	0.716067055	1.32E-05
CALU	mRNA	0.716011691	2.38E-06
hsa_circ_0090901	circRNA	0.715274514	2.39E-05
UBALD2	mRNA	0.715236624	1.53E-04
hsa_circ_0129527	circRNA	0.715233016	1.44E-04
hsa_circ_0021886	circRNA	0.715011056	8.71E-04
ATG9B	mRNA	0.714318787	0.021786055
hsa_circ_0090887	circRNA	0.714075961	0.011675637
ACSS2	mRNA	0.713213219	0.00211629
hsa_circ_0107786	circRNA	0.712633184	0.001045777
ELMSAN1	mRNA	0.712627054	0.001332857
TPM4	mRNA	0.712563221	4.25E-10
hsa_circ_0049428	circRNA	0.712479691	0.007455409
CEP85	mRNA	0.712375083	0.005603892
hsa_circ_0025415	circRNA	0.712158137	0.003078231
hsa_circ_0028845	circRNA	0.712082201	0.010837734

hsa_circ_0126264	circRNA	0.711419466	9.22E-04
hsa_circ_0050911	circRNA	0.711373054	0.014178345
hsa_circ_0073993	circRNA	0.711304079	0.002409843
COPB2	mRNA	0.711166255	2.02E-06
SRM	mRNA	0.711001549	0.001748114
FAM156B	mRNA	0.710845749	5.50E-09
hsa_circ_0007083	circRNA	0.708611649	3.21E-07
CDR2	mRNA	0.708515804	1.51E-04
hsa_circ_0042824	circRNA	0.708182014	0.023484368
hsa_circ_0003728	circRNA	0.708021904	0.006046503
TTLL12	mRNA	0.70739086	0.048929647
CEP83	mRNA	0.70676107	0.003218308
CEBPG	mRNA	0.706243764	2.45E-05
hsa_circ_0115597	circRNA	0.705788098	0.012815721
hsa_circ_0077830	circRNA	0.705590357	0.00327116
hsa_circ_0025431	circRNA	0.705145503	9.15E-04
hsa_circ_0084753	circRNA	0.705035044	0.004677111
hsa_circ_0122203	circRNA	0.705034318	2.34E-04
hsa_circ_0029325	circRNA	0.704636826	1.46E-04
C17orf51	lncRNA	0.704524851	0.004927829
SLFN13	mRNA	0.703945689	5.29E-05
hsa_circ_0105625	circRNA	0.703908918	0.001595656
hsa_circ_0079565	circRNA	0.703680996	0.02498847
UBE2J1	mRNA	0.703617312	0.010463489
hsa_circ_0030564	circRNA	0.703476363	0.003305303

hsa_circ_0007626	circRNA	0.702954548	0.014078056
hsa_circ_0067253	circRNA	0.702826743	1.10E-05
hsa_circ_0062876	circRNA	0.702299218	0.013063634
PDXK	mRNA	0.702203295	1.24E-05
hsa_circ_0098688	circRNA	0.701901393	0.012000727
RSPH3	mRNA	0.701652096	2.07E-04
PDGFA	mRNA	0.700346088	0.036794906
hsa_circ_0094626	circRNA	0.700189361	0.015693019
PCDH1	mRNA	0.699912109	0.0434775
LOC102724143	lncRNA	0.699831476	0.028089513
hsa_circ_0104004	circRNA	0.69926979	0.049879218
hsa_circ_0110665	circRNA	0.699194004	0.0381414
hsa_circ_0041267	circRNA	0.699029554	0.014209689
hsa_circ_0070234	circRNA	0.698886876	2.63E-09
hsa_circ_0029341	circRNA	0.698292317	0.005901017
hsa_circ_0023883	circRNA	0.697807125	0.004475924
hsa_circ_0004271	circRNA	0.697535237	4.82E-05
hsa_circ_0013693	circRNA	0.697256196	0.03425846
hsa_circ_0059422	circRNA	0.697210493	0.001610825
TP53INP2	mRNA	0.696931158	6.70E-07
GOSR2	mRNA	0.69685199	1.98E-06
GOLGA5	mRNA	0.696821906	3.57E-08
hsa_circ_0133562	circRNA	0.696814108	0.007661012
KLF10	mRNA	0.696481279	6.74E-04
POLD2	mRNA	0.696177904	0.001535968

hsa_circ_0031732	circRNA	0.696158455	9.59E-04
hsa_circ_0070247	circRNA	0.696055791	0.021696929
hsa_circ_0120356	circRNA	0.695359936	5.09E-06
DPYSL2	mRNA	0.695213626	8.94E-05
hsa_circ_0064279	circRNA	0.694720437	4.43E-05
FAM126A	mRNA	0.69397339	0.016872385
PRDX5	mRNA	0.693572866	3.79E-06
APOL2	mRNA	0.693152583	9.11E-05
CACNB1	mRNA	0.693077792	0.024209897
KIFC3	mRNA	0.692465683	0.002079846
hsa_circ_0125712	circRNA	0.69229276	0.022144694
hsa_circ_0094398	circRNA	0.691818624	0.034537877
hsa_circ_0124321	circRNA	0.691759875	0.044374932
SLC7A1	mRNA	0.691164617	2.99E-04
HCLS1	mRNA	0.690454731	0.017023595
hsa_circ_0136214	circRNA	0.690147483	2.13E-04
hsa_circ_0082149	circRNA	0.689954345	0.021204366
SEC31A	mRNA	0.689893773	1.02E-14
KALRN	mRNA	0.68975419	2.47E-06
hsa_circ_0024580	circRNA	0.689731909	1.40E-04
hsa_circ_0012121	circRNA	0.689677724	0.015191114
hsa_circ_0092069	circRNA	0.689404102	0.010216553
hsa_circ_0061892	circRNA	0.68940063	0.007375184
hsa_circ_0120361	circRNA	0.688979414	2.85E-04
SLC39A7	mRNA	0.688132937	4.71E-05

hsa_circ_0008530	circRNA	0.688128276	0.002670996
hsa_circ_0068992	circRNA	0.688062402	1.29E-04
RAPGEF5	mRNA	0.68766243	0.018127224
hsa_circ_0036200	circRNA	0.687353037	3.27E-04
DNM3	mRNA	0.687100797	0.018137633
TICAM1	mRNA	0.686323683	3.22E-04
SH3RF3-AS1	lncRNA	0.686090966	2.24E-04
hsa_circ_0058382	circRNA	0.685614165	0.005460628
CD59	mRNA	0.68558289	8.90E-06
GK5	mRNA	0.685401599	1.88E-05
hsa_circ_0074892	circRNA	0.684680725	0.002999265
LOC101929204	lncRNA	0.684085482	0.020380113
hsa_circ_0096173	circRNA	0.683738573	0.005646299
hsa_circ_0026128	circRNA	0.683535566	0.00430618
hsa_circ_0027902	circRNA	0.683064946	0.039723101
hsa_circ_0019036	circRNA	0.683003052	7.42E-05
PRAF2	mRNA	0.681752604	1.22E-07
hsa_circ_0099900	circRNA	0.681546287	0.007780652
hsa_circ_0067420	circRNA	0.681359319	0.001468434
hsa_circ_0005855	circRNA	0.680764915	0.021441401
hsa_circ_0060831	circRNA	0.680513356	0.006056351
HEATR5A	mRNA	0.679942385	2.55E-09
hsa_circ_0076003	circRNA	0.679912659	0.045327291
PCDHGA10	mRNA	0.679440698	0.010736496
hsa_circ_0008661	circRNA	0.679393714	9.74E-06

hsa_circ_0061078	circRNA	0.679345891	0.038709216
C12orf57	mRNA	0.678851276	8.46E-04
CDC42EP3	mRNA	0.678666414	2.58E-05
DESI1	mRNA	0.678492323	0.003555826
DNHD1	mRNA	0.678291796	0.010779166
NUAK2	mRNA	0.678010767	0.026875619
LOC112268301	lncRNA	0.677700103	2.83E-04
LOC105369332	lncRNA	0.67740309	9.29E-04
hsa_circ_0116681	circRNA	0.677037444	3.26E-04
hsa_circ_0039537	circRNA	0.676880932	0.005068808
hsa_circ_0132630	circRNA	0.675704935	0.010894072
hsa_circ_0074002	circRNA	0.675667916	0.02788788
ZBTB21	mRNA	0.675241737	2.45E-06
hsa_circ_0060012	circRNA	0.675183957	0.011755614
hsa_circ_0120815	circRNA	0.675154784	7.19E-04
PHYHIP	mRNA	0.674697516	0.006728445
hsa_circ_0004596	circRNA	0.674448676	4.32E-05
ETS2	mRNA	0.673487817	0.004289343
NAMPT	mRNA	0.673388163	1.48E-04
TVP23B	mRNA	0.673178002	9.20E-05
hsa_circ_0107680	circRNA	0.672604675	0.033110565
LOC101926956	lncRNA	0.672575479	0.024904859
RTL8C	mRNA	0.672522281	1.63E-08
hsa_circ_0005742	circRNA	0.672148337	0.001485756
hsa_circ_0054585	circRNA	0.671006206	2.02E-04

hsa_circ_0127093	circRNA	0.670955085	1.97E-07
BGIG9606_41928	lncRNA	0.670838922	4.50E-04
hsa_circ_0079079	circRNA	0.670739661	1.55E-04
LINC00921	lncRNA	0.670689033	0.007755051
LGALSL	mRNA	0.670562272	3.53E-05
TALDO1	mRNA	0.670213994	0.012953158
hsa_circ_0006670	circRNA	0.670040111	0.04477533
UCP2	mRNA	0.67000714	0.032596625
hsa_circ_0096185	circRNA	0.66974665	0.019237932
GMPPA	mRNA	0.6696336	1.37E-05
hsa_circ_0024583	circRNA	0.668843055	0.013040988
HABP4	mRNA	0.668786696	0.003572503
CD276	mRNA	0.667829178	0.012806579
LOC107985200	lncRNA	0.667747795	0.022147036
TAF4B	mRNA	0.667466936	9.68E-04
CETN2	mRNA	0.66740429	6.48E-07
ZBTB42	mRNA	0.667353222	4.00E-08
hsa_circ_0134442	circRNA	0.66726341	0.001443135
hsa_circ_0135681	circRNA	0.667092218	0.014078056
hsa_circ_0000954	circRNA	0.667071647	0.002392036
hsa_circ_0002703	circRNA	0.666311159	0.014990324
FNTB	mRNA	0.666094435	0.006444562
IL15RA	mRNA	0.666045723	6.65E-05
hsa_circ_0081341	circRNA	0.66598735	0.008949684
hsa_circ_0024581	circRNA	0.665361665	9.37E-04

GUK1	mRNA	0.665146264	1.00E-10
KRT10	mRNA	0.665123605	1.55E-04
LIMK2	mRNA	0.664148866	5.68E-04
hsa_circ_0001329	circRNA	0.664132198	0.010646152
JAG2	mRNA	0.663941927	0.006468765
hsa_circ_0093013	circRNA	0.66387535	0.007422306
SLC30A5	mRNA	0.663147311	5.59E-04
MYO7A	mRNA	0.662982638	0.018136338
hsa_circ_0004716	circRNA	0.662914306	0.001716749
hsa_circ_0101727	circRNA	0.662392989	0.010116461
hsa_circ_0129530	circRNA	0.661993211	0.009449907
MARVELD1	mRNA	0.661500948	6.18E-10
RABEPK	mRNA	0.661421551	0.001489154
hsa_circ_0058273	circRNA	0.660092349	0.018293194
hsa_circ_0054561	circRNA	0.660011458	0.001047711
PGP	mRNA	0.659747929	0.018673611
hsa_circ_0088768	circRNA	0.659462285	0.003647447
FXYD5	mRNA	0.659275703	5.68E-04
hsa_circ_0067592	circRNA	0.658743447	0.039689246
hsa_circ_0053115	circRNA	0.658684778	2.77E-05
BGIG9606_45075	lncRNA	0.657914885	0.020329353
SPTSSA	mRNA	0.65759711	6.72E-06
ZNF526	mRNA	0.657447111	5.13E-04
hsa_circ_0088701	circRNA	0.657383697	0.037202055
ZFP69B	mRNA	0.657154186	0.03470128

LOC112268225	lncRNA	0.657051692	0.001089624
GNAI2	mRNA	0.656937284	3.23E-07
QPCTL	mRNA	0.656849706	6.68E-04
SLC35C1	mRNA	0.656687049	1.15E-04
CREB3	mRNA	0.656520934	6.54E-07
hsa_circ_0120359	circRNA	0.656426365	1.74E-06
SUN1	mRNA	0.655666407	1.91E-07
hsa_circ_0029354	circRNA	0.655185029	1.41E-06
C16orf45	mRNA	0.655070271	0.004616766
hsa_circ_0009749	circRNA	0.654889857	0.023326068
SH3BGRL3	mRNA	0.654225684	1.73E-05
hsa_circ_0025728	circRNA	0.654125661	0.029627365
hsa_circ_0079082	circRNA	0.653794366	4.64E-04
HRAT92	lncRNA	0.653740142	0.023295447
ACSL4	mRNA	0.653644095	0.001931596
hsa_circ_0055054	circRNA	0.653405568	0.01331942
XBP1	mRNA	0.653275785	5.09E-04
COTL1	mRNA	0.653228864	3.97E-04
hsa_circ_0001850	circRNA	0.653131424	0.006410468
DNAJA4	mRNA	0.652839082	0.02047637
PTPRM	mRNA	0.652710653	0.0014594
hsa_circ_0062885	circRNA	0.652191778	0.039808867
hsa_circ_0036775	circRNA	0.652072892	0.002851506
GORASP2	mRNA	0.651912446	2.14E-06
FLOT1	mRNA	0.65119007	0.006242485

SLC43A2	mRNA	0.651164469	7.21E-05
hsa_circ_0053116	circRNA	0.651104148	6.87E-05
hsa_circ_0132629	circRNA	0.650699008	0.001491688
hsa_circ_0077467	circRNA	0.650693339	0.033076375
ZDHHC18	mRNA	0.65012686	1.45E-05
hsa_circ_0018635	circRNA	0.649641025	5.25E-04
hsa_circ_0003319	circRNA	0.649633214	0.001990179
IER2	mRNA	0.649488243	0.001478237
hsa_circ_0023338	circRNA	0.64926857	0.027441597
MRPL17	mRNA	0.648977805	8.53E-06
hsa_circ_0021621	circRNA	0.648969282	7.50E-06
hsa_circ_0014866	circRNA	0.648894503	0.015789389
CLIC1	mRNA	0.648007732	0.003919603
hsa_circ_0067872	circRNA	0.647828309	0.017388354
DAAM1	mRNA	0.647693442	8.61E-04
hsa_circ_0019697	circRNA	0.647584226	0.0125096
hsa_circ_0042807	circRNA	0.64723343	0.018508192
YIPF2	mRNA	0.647111618	3.82E-06
hsa_circ_0007432	circRNA	0.646791465	0.046387573
UBC	mRNA	0.646700549	1.02E-05
hsa_circ_0120352	circRNA	0.646459602	4.78E-06
SAR1A	mRNA	0.645789549	2.18E-05
hsa_circ_0101402	circRNA	0.64527129	0.002324543
hsa_circ_0091322	circRNA	0.645241304	0.001893902
hsa_circ_0127076	circRNA	0.644595709	2.06E-05

GPAT4	mRNA	0.644555833	7.84E-04
hsa_circ_0108936	circRNA	0.644413529	0.010116461
LAMA5	mRNA	0.644309192	0.002375111
TMF1	mRNA	0.643813398	5.05E-09
hsa_circ_0061092	circRNA	0.643644272	0.04628003
hsa_circ_0009140	circRNA	0.643564394	0.028187112
hsa_circ_0125389	circRNA	0.643138667	5.87E-04
WDR45	mRNA	0.642854763	4.71E-04
SLC31A1	mRNA	0.642766272	1.47E-04
RAP2B	mRNA	0.642547025	0.009638733
hsa_circ_0105245	circRNA	0.642499028	8.56E-06
hsa_circ_0028950	circRNA	0.642352181	0.002554266
hsa_circ_0023884	circRNA	0.642304515	0.005130608
MSN	mRNA	0.641321529	2.34E-05
hsa_circ_0019422	circRNA	0.641316424	0.005187928
hsa_circ_0005504	circRNA	0.641181338	0.002251709
hsa_circ_0109024	circRNA	0.641115037	0.025915174
hsa_circ_0111071	circRNA	0.640968077	0.001737925
hsa_circ_0016770	circRNA	0.640765886	1.65E-06
SEPT6	mRNA	0.639958304	0.003688177
hsa_circ_0003307	circRNA	0.639903542	0.006009065
hsa_circ_0014208	circRNA	0.639871802	0.003413311
DHDDS	mRNA	0.639756535	3.36E-06
PLEKHA1	mRNA	0.63927415	0.004774499
hsa_circ_0028949	circRNA	0.63927227	0.006830087

MSTO1	mRNA	0.639155034	1.79E-04
hsa_circ_0004470	circRNA	0.638935677	0.028255625
TMEM167A	mRNA	0.638445184	9.88E-05
hsa_circ_0104878	circRNA	0.63839358	0.004339815
CMTM3	mRNA	0.638337353	0.021873758
BGIG9606_54989	mRNA	0.638313302	1.61E-04
hsa_circ_0000653	circRNA	0.638090399	9.51E-04
hsa_circ_0014910	circRNA	0.638089576	0.044451627
hsa_circ_0031729	circRNA	0.637736358	0.00668815
SLC7A6	mRNA	0.637151074	9.44E-08
NACAD	mRNA	0.636746179	0.011114668
hsa_circ_0084613	circRNA	0.634570432	0.020003806
hsa_circ_0038054	circRNA	0.634360238	0.030505626
hsa_circ_0067275	circRNA	0.634203098	7.76E-05
hsa_circ_0079080	circRNA	0.633605379	0.005217455
HID1	mRNA	0.633528184	7.81E-10
hsa_circ_0070253	circRNA	0.633338147	0.007674614
hsa_circ_0025046	circRNA	0.633244357	0.022876334
hsa_circ_0074661	circRNA	0.633065181	3.64E-04
ZBED8	mRNA	0.632955238	4.39E-04
AEN	mRNA	0.632940859	0.009358401
TBC1D9	mRNA	0.632188746	0.006569125
hsa_circ_0020696	circRNA	0.632119047	0.005787
GLRX	mRNA	0.631735344	0.006905957
hsa_circ_0003549	circRNA	0.631362066	8.23E-06

hsa_circ_0058391	circRNA	0.631195479	0.017959634
SSH2	mRNA	0.630921397	0.010512338
hsa_circ_0042820	circRNA	0.63089659	0.010885303
MOSMO	mRNA	0.630769236	0.006998568
OGFOD1	mRNA	0.630718425	7.29E-04
SRD5A3	mRNA	0.630475363	1.06E-04
hsa_circ_0046816	circRNA	0.630245545	0.026546082
hsa_circ_0111658	circRNA	0.630208663	7.82E-04
hsa_circ_0108932	circRNA	0.629960245	0.00668815
hsa_circ_0106447	circRNA	0.629626491	0.003064532
MAGED1	mRNA	0.62951227	0.001782116
hsa_circ_0127088	circRNA	0.629317311	8.02E-04
hsa_circ_0047776	circRNA	0.629136328	0.003279744
SLC10A3	mRNA	0.629100273	1.55E-07
ATAD3A	mRNA	0.628981221	0.004964272
hsa_circ_0126963	circRNA	0.628312991	0.047636836
hsa_circ_0033548	circRNA	0.628226391	0.025614217
SCARF2	mRNA	0.628155154	0.001398948
NPHP4	mRNA	0.628101232	0.001520035
hsa_circ_0054978	circRNA	0.627677786	6.74E-06
hsa_circ_0079065	circRNA	0.627501769	0.001974056
MAGEH1	mRNA	0.627454796	4.74E-06
hsa_circ_0042799	circRNA	0.627001018	0.048669619
hsa_circ_0003107	circRNA	0.626990745	5.47E-04
PWWP2B	mRNA	0.62663709	0.009399885

hsa_circ_0129528	circRNA	0.62606783	0.044107877
hsa_circ_0106506	circRNA	0.625977369	0.026881972
hsa_circ_0042810	circRNA	0.625949374	0.001487875
hsa_circ_0129531	circRNA	0.625543451	0.037202055
hsa_circ_0008955	circRNA	0.625540936	0.005281932
hsa_circ_0079064	circRNA	0.625493436	0.001528119
hsa_circ_0077207	circRNA	0.625454666	0.01148743
hsa_circ_0110427	circRNA	0.625410198	0.02735311
hsa_circ_0067705	circRNA	0.625332544	0.039895567
CHPF2	mRNA	0.625252355	1.34E-04
ARHGAP27P1-BPTFP1-KPNA2P3	pseudo	0.625226212	3.61E-05
hsa_circ_0065847	circRNA	0.625089889	1.22E-06
BGIG9606_42210	lncRNA	0.624910848	0.012256154
POMT2	mRNA	0.624544908	0.00204087
SPTBN1	mRNA	0.624438039	3.30E-05
hsa_circ_0009497	circRNA	0.624418739	0.008664153
hsa_circ_0019421	circRNA	0.624373503	0.040186187
hsa_circ_0004261	circRNA	0.623983788	0.016628322
hsa_circ_0115395	circRNA	0.62308608	0.012483156
hsa_circ_0004618	circRNA	0.622679622	0.016021655
AHRR	mRNA	0.622676376	0.039409006
hsa_circ_0000753	circRNA	0.622625203	0.021272295
hsa_circ_0014888	circRNA	0.622540208	0.027619437
hsa_circ_0101724	circRNA	0.622447014	0.002795016
hsa_circ_0026130	circRNA	0.622214461	0.021138906

EHBP1L1	mRNA	0.622009005	0.036477952
CORO7	mRNA	0.621852581	0.004514569
hsa_circ_0045937	circRNA	0.621850433	0.045245238
hsa_circ_0003121	circRNA	0.621693207	0.005390466
PPFIBP1	mRNA	0.620983731	2.33E-08
hsa_circ_0096402	circRNA	0.620935794	0.002497738
OAT	mRNA	0.620915652	9.21E-06
CIB1	mRNA	0.620422711	3.48E-07
DGKA	mRNA	0.620289825	3.85E-04
LRFN4	mRNA	0.619964031	1.23E-04
SYNC	mRNA	0.61987892	0.002055753
SPAG5-AS1	lncRNA	0.619479269	0.01706378
hsa_circ_0007451	circRNA	0.619290864	0.004138322
LIPA	mRNA	0.619203497	3.06E-14
hsa_circ_0136221	circRNA	0.618772707	0.00263322
hsa_circ_0136213	circRNA	0.618618186	0.006460929
hsa_circ_0128010	circRNA	0.617966572	0.00357183
hsa_circ_0057332	circRNA	0.617833089	0.001141912
SEC23A	mRNA	0.617780645	2.48E-05
hsa_circ_0053118	circRNA	0.617451315	0.005210999
TMEM140	mRNA	0.617244064	0.02985415
hsa_circ_0000339	circRNA	0.617191476	0.003842903
DNAJC10	mRNA	0.617082348	2.85E-06
CCND3	mRNA	0.6169679	9.68E-05
LY6E	mRNA	0.616852857	0.006117644

TBL2	mRNA	0.616590999	2.39E-05
hsa_circ_0018634	circRNA	0.616554416	1.62E-04
STX2	mRNA	0.616512235	7.20E-10
AGAP2-AS1	lncRNA	0.616286986	0.009974154
hsa_circ_0067274	circRNA	0.616002961	3.70E-04
BET1L	mRNA	0.615691501	3.19E-05
RAB6A	mRNA	0.614364359	2.27E-13
LINC01128	lncRNA	0.614324719	0.006348892
hsa_circ_0039448	circRNA	0.614072519	0.025081735
ESYT2	mRNA	0.613757369	1.90E-05
CD46	mRNA	0.613741529	1.85E-07
hsa_circ_0011992	circRNA	0.613639874	0.007129887
EML6	mRNA	0.613420243	4.22E-04
FIGNL2	mRNA	0.612800557	0.006469895
P2RY2	mRNA	0.612781237	0.042925898
hsa_circ_0107722	circRNA	0.612742027	0.041555069
hsa_circ_0140372	circRNA	0.612365584	0.001777335
ZNF598	mRNA	0.61196137	0.005716891
MORF4L2	mRNA	0.611960614	1.79E-04
hsa_circ_0006894	circRNA	0.611607879	0.009741082
hsa_circ_0014901	circRNA	0.611236171	0.002139247
RUSC1-AS1	lncRNA	0.611089763	0.002499666
hsa_circ_0120779	circRNA	0.610900991	6.57E-06
DPH2	mRNA	0.610321623	0.004842361
hsa_circ_0023508	circRNA	0.610169251	0.008625659

IMPDH1	mRNA	0.610011993	5.74E-04
hsa_circ_0004088	circRNA	0.608978928	0.019893757
NAGA	mRNA	0.608799991	2.18E-04
hsa_circ_0098197	circRNA	0.608782781	0.02849211
PCBD2	mRNA	0.608649162	8.66E-04
MANF	mRNA	0.608631359	0.011486176
hsa_circ_0129526	circRNA	0.60792081	0.008430997
UFM1	mRNA	0.607660455	5.90E-04
hsa_circ_0083196	circRNA	0.607204142	0.022250974
INF2	mRNA	0.607185802	5.82E-04
hsa_circ_0069368	circRNA	0.607123568	0.00392823
RASSF1	mRNA	0.606630268	1.69E-05
CAMKK2	mRNA	0.606433789	0.0011395
YKT6	mRNA	0.606281912	0.001968986
hsa_circ_0011207	circRNA	0.604826618	0.030999246
hsa_circ_0088129	circRNA	0.604701364	0.032019588
hsa_circ_0022689	circRNA	0.604205883	0.006344075
hsa_circ_0001225	circRNA	0.604025975	0.002914893
REEP3	mRNA	0.603714205	0.023068772
GLUD1	mRNA	0.603705367	6.34E-07
hsa_circ_0070997	circRNA	0.603542838	0.001022424
hsa_circ_0061089	circRNA	0.603453725	0.023183805
hsa_circ_0090886	circRNA	0.603386777	7.24E-05
IL4R	mRNA	0.603356353	1.77E-12
hsa_circ_0039445	circRNA	0.603056151	0.015102124

hsa_circ_0097078	circRNA	0.602693744	0.021113208
hsa_circ_0080218	circRNA	0.602637477	0.007660044
hsa_circ_0088767	circRNA	0.602632108	0.02431567
hsa_circ_0023503	circRNA	0.602332032	1.18E-04
P3H4	mRNA	0.602303664	0.001753985
LINC00888	lncRNA	0.601543121	0.022509946
APAF1	mRNA	0.601396731	2.66E-06
ZEB1	mRNA	0.601301674	0.018431547
hsa_circ_0134373	circRNA	0.601299536	0.010318986
SEC61G	mRNA	0.601278082	0.002625743
CEBPB	mRNA	0.601138509	0.001377555
CFLAR	mRNA	0.599857891	2.28E-06
SIAH2	mRNA	0.599753605	6.59E-04
LYSMD4	mRNA	0.599543685	8.95E-04
hsa_circ_0036197	circRNA	0.599334009	0.003662344
hsa_circ_0057015	circRNA	0.599263397	0.030891206
ATG101	mRNA	0.599185363	1.91E-07
TULP3	mRNA	0.599041405	5.49E-08
PKM	mRNA	0.598987377	0.013126816
DUSP7	mRNA	0.598752891	2.10E-05
BGIG9606_54350	mRNA	0.59860325	0.047571523
LAPTM5	mRNA	0.598531766	0.04836633
MCM3AP-AS1	lncRNA	0.598031845	0.030372292
TMEM120B	mRNA	0.597970076	0.001993163
hsa_circ_0004106	circRNA	0.597719961	0.006171983

hsa_circ_0001776	circRNA	0.597196891	0.004241963
ORAI2	mRNA	0.597050511	2.03E-06
hsa_circ_0022828	circRNA	0.596863053	0.021711922
RECQL	mRNA	0.596651822	1.05E-09
hsa_circ_0098604	circRNA	0.596234231	0.01204008
hsa_circ_0106504	circRNA	0.595965695	0.046401286
SLC36A4	mRNA	0.595847257	0.001149992
RNF141	mRNA	0.595627172	0.028959411
SLC2A10	mRNA	0.595135548	0.019808035
hsa_circ_0087225	circRNA	0.594821913	0.003315745
hsa_circ_0075045	circRNA	0.594318263	0.004495211
TCTA	mRNA	0.594294247	1.59E-13
KIAA1522	mRNA	0.594030649	0.006272016
hsa_circ_0022582	circRNA	0.59383301	0.013747319
hsa_circ_0029349	circRNA	0.593657889	2.94E-05
hsa_circ_0025438	circRNA	0.593063849	0.011882044
hsa_circ_0018803	circRNA	0.592874386	0.027619437
ZDHHC1	mRNA	0.592651522	0.008500912
hsa_circ_0128551	circRNA	0.592596381	0.017556376
hsa_circ_0063634	circRNA	0.592365751	0.016453947
CSTB	mRNA	0.592285222	8.05E-08
hsa_circ_0031712	circRNA	0.592132145	0.001404631
hsa_circ_0004426	circRNA	0.591735811	0.011725572
hsa_circ_0047780	circRNA	0.591576787	0.005281932
hsa_circ_0127086	circRNA	0.590926972	0.002313442

hsa_circ_0079278	circRNA	0.590784908	0.038260925
hsa_circ_0000458	circRNA	0.590612591	8.58E-04
hsa_circ_0116684	circRNA	0.590411383	0.003108846
CDK2AP2	mRNA	0.59013011	0.008125436
hsa_circ_0074658	circRNA	0.590114343	0.002486727
hsa_circ_0042946	circRNA	0.589872254	0.035551348
TXNDC11	mRNA	0.589671263	1.91E-05
hsa_circ_0014924	circRNA	0.58898721	0.00218306
CALCRL	mRNA	0.58896041	0.008025879
hsa_circ_0083205	circRNA	0.588913336	0.008289431
hsa_circ_0002872	circRNA	0.588693465	0.039556116
PDE4DIP	mRNA	0.58850448	5.54E-04
hsa_circ_0042843	circRNA	0.588456673	0.027619437
hsa_circ_0009142	circRNA	0.588372547	0.027496654
hsa_circ_0024766	circRNA	0.588259674	0.001439963
DUSP1	mRNA	0.588039064	2.61E-07
NPR1	mRNA	0.587087136	0.001062716
RAP1B	mRNA	0.587041896	9.71E-04
GPR173	mRNA	0.586562107	0.001014751
MYBBP1A	mRNA	0.586295792	0.006008657
PLCB1	mRNA	0.585567542	5.97E-11
NDE1	mRNA	0.58531604	4.28E-05
hsa_circ_0026896	circRNA	0.585310411	0.007251928
COQ10B	mRNA	0.584792095	0.006171388
SLC35E1	mRNA	0.584535487	6.59E-06

VSIG2	mRNA	0.584322676	0.024431547
SH3BP5-AS1	lncRNA	0.584108559	0.001172143
hsa_circ_0043683	circRNA	0.583610371	0.001828438
hsa_circ_0120358	circRNA	0.583504327	0.02162586
hsa_circ_0070836	circRNA	0.583400277	0.046980874
hsa_circ_0091324	circRNA	0.583283969	0.045326933
N6AMT1	mRNA	0.583076043	0.005710589
hsa_circ_0054979	circRNA	0.582675248	0.008984773
CLCN6	mRNA	0.582015179	1.91E-07
hsa_circ_0092068	circRNA	0.582005899	0.011182134
SH3BGRL2	mRNA	0.581785216	0.001089624
hsa_circ_0037869	circRNA	0.581682079	0.013928438
hsa_circ_0009750	circRNA	0.581509419	0.009328307
ARFGAP3	mRNA	0.581255349	3.75E-11
NLN	mRNA	0.581056924	0.008171546
ETV5	mRNA	0.580905532	5.08E-04
MYADM	mRNA	0.580566833	7.21E-05
SLC35C2	mRNA	0.580019696	6.48E-10
hsa_circ_0127094	circRNA	0.579969487	1.96E-04
hsa_circ_0089921	circRNA	0.579798664	5.28E-04
hsa_circ_0003927	circRNA	0.579581933	0.002808203
LPCAT4	mRNA	0.579475545	0.00225627
ATP1B3	mRNA	0.579239641	0.003067971
ARHGAP1	mRNA	0.579071833	9.83E-11
hsa_circ_0058280	circRNA	0.578846915	0.012761167

hsa_circ_0130911	circRNA	0.578694457	0.037905137
TMSB10	mRNA	0.578638368	1.95E-06
hsa_circ_0024579	circRNA	0.578398898	0.049183851
PLCB3	mRNA	0.578377963	5.19E-05
hsa_circ_0025625	circRNA	0.578039852	0.014359458
SEC23IP	mRNA	0.577876178	2.77E-04
hsa_circ_0011985	circRNA	0.577601878	0.003910929
hsa_circ_0139651	circRNA	0.577589739	0.013662901
EMP1	mRNA	0.577563862	0.006689655
NOS3	mRNA	0.577480284	0.023203493
hsa_circ_0102937	circRNA	0.577267098	0.00295689
hsa_circ_0080219	circRNA	0.576843067	0.002831258
MSRB3	mRNA	0.576262732	6.39E-06
SLC26A1	mRNA	0.57610372	0.011345931
hsa_circ_0116546	circRNA	0.576101116	0.024995357
SLC39A14	mRNA	0.576088736	0.029369075
hsa_circ_0127505	circRNA	0.576054957	2.60E-04
TRAK2	mRNA	0.575967342	0.00245839
GNPNAT1	mRNA	0.575923155	0.001043953
FRMD8	mRNA	0.575799284	2.32E-04
TMEM263	mRNA	0.574632878	4.95E-04
YIF1B	mRNA	0.574082087	0.008166806
ARHGAP27P1	pseudo	0.573373959	0.012739825
TIMM10B	mRNA	0.573277816	7.40E-06
MED31	mRNA	0.573222296	3.99E-04

PHETA2	mRNA	0.572519854	1.15E-04
hsa_circ_0096355	circRNA	0.572337945	0.015892126
hsa_circ_0046808	circRNA	0.572242611	0.044461959
hsa_circ_0018810	circRNA	0.572048115	0.004454615
IKBIP	mRNA	0.572046702	2.13E-08
A2M	mRNA	0.571902135	0.002050635
P4HA1	mRNA	0.571417937	3.07E-04
KIAA1841	mRNA	0.571346894	6.74E-04
hsa_circ_0040696	circRNA	0.571332679	0.011636689
ANKRD40	mRNA	0.570985441	0.00975883
FKBP14	mRNA	0.570677585	0.010492623
ZNF799	mRNA	0.570364089	0.001302545
hsa_circ_0107780	circRNA	0.570221694	0.041630541
SLC39A13	mRNA	0.570001032	2.10E-05
EBLN3P	pseudo	0.56982745	8.07E-07
hsa_circ_0069243	circRNA	0.569784059	0.013083524
hsa_circ_0094256	circRNA	0.56964693	0.007498735
hsa_circ_0067024	circRNA	0.569303399	0.007405982
C15orf39	mRNA	0.568664009	0.042178912
HEMK1	mRNA	0.568654221	5.57E-04
COG6	mRNA	0.568542948	1.89E-05
GRK5	mRNA	0.568476028	0.015481943
CYP20A1	mRNA	0.568258828	4.99E-10
RRAS	mRNA	0.568211586	0.001117808
LARP1B	mRNA	0.567788795	8.04E-05

hsa_circ_0028951	circRNA	0.567778869	0.0086266
hsa_circ_0068221	circRNA	0.567566729	0.010894714
ZFYVE28	mRNA	0.567526846	2.17E-10
hsa_circ_0134988	circRNA	0.567404231	0.013244599
KLF9	mRNA	0.56712806	0.046757085
hsa_circ_0014934	circRNA	0.567097089	0.011636689
C2CD2L	mRNA	0.567070996	0.009212654
hsa_circ_0004467	circRNA	0.566994735	0.037962713
hsa_circ_0101132	circRNA	0.565836405	0.035346271
hsa_circ_0027461	circRNA	0.565573197	0.007498735
IQCE	mRNA	0.56548266	1.38E-04
PPP1R18	mRNA	0.564937136	0.039821756
TNFAIP1	mRNA	0.564478008	7.09E-07
hsa_circ_0036780	circRNA	0.563501225	0.005689146
hsa_circ_0096172	circRNA	0.563483429	0.00676003
PTGES2-AS1	lncRNA	0.563474131	0.036891901
hsa_circ_0067189	circRNA	0.563094953	6.00E-04
hsa_circ_0057333	circRNA	0.56283638	0.007811709
CD81	mRNA	0.562401986	6.17E-04
CSNK1G2	mRNA	0.561996094	4.86E-05
hsa_circ_0101788	circRNA	0.56176235	0.001296719
ATP8B1	mRNA	0.561570823	0.005096109
COPA	mRNA	0.561446288	2.42E-08
hsa_circ_0007000	circRNA	0.561027839	0.002422279
hsa_circ_0061707	circRNA	0.560784243	0.043187929

hsa_circ_0031054	circRNA	0.560664802	0.027721962
SREBF1	mRNA	0.560640495	0.021963044
TRPM4	mRNA	0.56018849	0.037184711
PLPPR2	mRNA	0.559130461	1.40E-06
hsa_circ_0127087	circRNA	0.55887305	0.001756843
SLC26A6	mRNA	0.558849523	0.004355556
hsa_circ_0068306	circRNA	0.558578964	0.032844494
hsa_circ_0009357	circRNA	0.55849089	0.020706617
hsa_circ_0025292	circRNA	0.558448563	0.002313442
hsa_circ_0096403	circRNA	0.55838952	0.022461612
ITPRIPL2	mRNA	0.558317305	0.00511032
hsa_circ_0043244	circRNA	0.557998746	0.017171743
hsa_circ_0002676	circRNA	0.557863204	0.002741436
hsa_circ_0001848	circRNA	0.557631672	0.015023785
TTLL7	mRNA	0.556943454	0.016797255
hsa_circ_0095123	circRNA	0.556589163	0.00707892
UBE2D4	mRNA	0.556281587	0.003329689
DCLK2	mRNA	0.556229674	0.001676746
MMP15	mRNA	0.555721393	0.009789511
hsa_circ_0016550	circRNA	0.555677222	0.048667815
hsa_circ_0009356	circRNA	0.555477729	0.033110565
hsa_circ_0014930	circRNA	0.555416827	0.00226014
hsa_circ_0136217	circRNA	0.555067804	0.001983687
POGLUT1	mRNA	0.554873115	5.39E-04
ATP13A1	mRNA	0.55472185	5.18E-05

SYNE1	mRNA	0.554166934	0.022933752
TIMP1	mRNA	0.554058152	2.44E-06
hsa_circ_0020802	circRNA	0.553625412	0.00137368
SH2B3	mRNA	0.553501129	8.52E-04
hsa_circ_0097529	circRNA	0.55347487	0.005553795
hsa_circ_0067555	circRNA	0.552780725	8.72E-04
C2orf76	mRNA	0.552700014	0.005963302
hsa_circ_0083216	circRNA	0.552461656	0.011842673
hsa_circ_0042308	circRNA	0.55165206	0.018295239
hsa_circ_0115730	circRNA	0.551160197	0.036292609
hsa_circ_0086397	circRNA	0.550888377	0.039740662
MGST2	mRNA	0.550363797	7.02E-06
PPAN-P2RY11	mRNA	0.550042997	0.002526666
hsa_circ_0101790	circRNA	0.5497169	0.001332982
hsa_circ_0007264	circRNA	0.5493764	0.006277854
hsa_circ_0014361	circRNA	0.549365047	0.025397611
hsa_circ_0109158	circRNA	0.548563645	0.008869339
hsa_circ_0003376	circRNA	0.547805661	0.021992611
SPATA2L	mRNA	0.547767989	0.002534473
SGTB	mRNA	0.5477559	0.013019932
MINAR1	mRNA	0.54741399	0.039840706
hsa_circ_0059050	circRNA	0.547392545	0.030822295
IER3IP1	mRNA	0.547089823	0.006249572
IFT20	mRNA	0.546545085	3.77E-04
hsa_circ_0024051	circRNA	0.546066833	0.022180757

hsa_circ_0090447	circRNA	0.545807992	0.037941183
SERPINB8	mRNA	0.545310348	0.00947183
hsa_circ_0096643	circRNA	0.54528666	0.031325204
hsa_circ_0094354	circRNA	0.54494339	0.001472761
PVRIG2P	pseudo	0.54487661	0.01489954
hsa_circ_0078098	circRNA	0.544737226	0.045843499
PDIA5	mRNA	0.543896457	7.57E-04
hsa_circ_0096354	circRNA	0.543712437	8.79E-04
hsa_circ_0029345	circRNA	0.543703068	0.032668037
hsa_circ_0079282	circRNA	0.543437027	0.011801801
GBF1	mRNA	0.543407713	1.07E-04
MLEC	mRNA	0.543260211	0.026843007
SEC11C	mRNA	0.54319792	0.02992686
hsa_circ_0026131	circRNA	0.543187741	0.022422157
hsa_circ_0075174	circRNA	0.543100292	0.018387706
hsa_circ_0055345	circRNA	0.543092432	0.007055848
GARS	mRNA	0.543053379	0.004842361
hsa_circ_0001227	circRNA	0.542791094	0.02853803
ZNF70	mRNA	0.542684601	0.001904273
hsa_circ_0039546	circRNA	0.54265304	0.00668815
hsa_circ_0074886	circRNA	0.542270209	0.024687657
SHISA5	mRNA	0.542243234	4.33E-05
GLT8D1	mRNA	0.542098354	4.01E-08
hsa_circ_0006791	circRNA	0.541884591	0.008768531
HPCAL1	mRNA	0.541550971	0.018920711

hsa_circ_0036203	circRNA	0.541138277	0.041032602
KLF6	mRNA	0.541058008	8.55E-06
hsa_circ_0026136	circRNA	0.540486208	0.027373375
LOC100506571	lncRNA	0.540448572	0.013785833
hsa_circ_0059701	circRNA	0.540418353	0.007331861
HM13	mRNA	0.539850441	2.38E-06
GNL3	mRNA	0.539630177	0.004537643
ZNF275	mRNA	0.539268651	2.83E-04
hsa_circ_0067556	circRNA	0.538814234	0.03313518
hsa_circ_0067014	circRNA	0.53841848	0.024762495
hsa_circ_0059699	circRNA	0.538408879	0.00357183
hsa_circ_0119649	circRNA	0.538372299	0.032435572
ALG3	mRNA	0.53820986	0.003330702
PNPLA8	mRNA	0.537784383	9.43E-04
RNF216P1	pseudo	0.537570482	2.82E-05
ZNFX1	mRNA	0.537531723	1.61E-05
hsa_circ_0065844	circRNA	0.537207833	5.26E-04
SLC36A1	mRNA	0.537052877	0.00838653
hsa_circ_0056490	circRNA	0.536958249	0.010722404
CNOT9	mRNA	0.536374273	0.012245075
MOB3C	mRNA	0.536317408	0.011169518
UVSSA	mRNA	0.536099286	0.001336925
hsa_circ_0134661	circRNA	0.535837954	0.00362502
CAP1	mRNA	0.535435617	0.001127167
hsa_circ_0132969	circRNA	0.535251165	0.036371598

hsa_circ_0038986	circRNA	0.5346062	0.001605755
hsa_circ_0001421	circRNA	0.534415912	0.002106136
TMEM208	mRNA	0.534379889	3.59E-04
ITGA6	mRNA	0.534256698	0.00604998
hsa_circ_0022670	circRNA	0.5338614	0.026954203
TTLL11	mRNA	0.533554192	1.73E-05
hsa_circ_0011989	circRNA	0.53319873	0.009302894
hsa_circ_0006618	circRNA	0.532308386	0.002903763
hsa_circ_0094124	circRNA	0.532177414	0.036050386
GPX8	mRNA	0.532049856	0.042525181
CYB561D2	mRNA	0.531837422	0.032841329
GRAMD4	mRNA	0.53177589	2.33E-04
hsa_circ_0062359	circRNA	0.531747013	0.026881972
DPP9	mRNA	0.531585751	6.06E-04
NXPE3	mRNA	0.531526919	0.015651164
hsa_circ_0059697	circRNA	0.531218034	0.001272316
C1orf198	mRNA	0.531007126	1.25E-05
hsa_circ_0023885	circRNA	0.530467872	0.043332833
hsa_circ_0121196	circRNA	0.53033352	0.012899173
hsa_circ_0019068	circRNA	0.530137422	0.001041202
CBX2	mRNA	0.52990255	0.006171388
hsa_circ_0029350	circRNA	0.529856202	0.00325145
hsa_circ_0036796	circRNA	0.529545641	0.03891606
TMEM273	mRNA	0.529540244	0.029369075
hsa_circ_0098198	circRNA	0.529487137	0.006622995

SLC30A1	mRNA	0.529435112	1.05E-04
hsa_circ_0068346	circRNA	0.529257426	0.033639362
hsa_circ_0021889	circRNA	0.528924037	0.018198725
B4GALT2	mRNA	0.528741274	0.022439881
hsa_circ_0019715	circRNA	0.528654781	0.008122375
hsa_circ_0075208	circRNA	0.52863346	0.034740498
RPL23AP53	pseudo	0.52852379	0.010853037
KLF16	mRNA	0.528230579	0.012857171
hsa_circ_0101148	circRNA	0.52805846	0.007606906
hsa_circ_0111785	circRNA	0.528037131	0.001108113
CPEB4	mRNA	0.527555104	0.01600153
hsa_circ_0041800	circRNA	0.527452671	0.01852778
hsa_circ_0087553	circRNA	0.527428667	0.010678106
GTPBP2	mRNA	0.52700124	0.001352567
SLC20A1	mRNA	0.52653267	0.013314944
hsa_circ_0069371	circRNA	0.526528383	0.018274245
hsa_circ_0009000	circRNA	0.526271806	0.014854053
hsa_circ_0061344	circRNA	0.525852846	0.014475145
AMOTL1	mRNA	0.525685505	0.001486947
MFSD14A	mRNA	0.525340024	5.01E-06
hsa_circ_0020197	circRNA	0.525176413	0.01670908
hsa_circ_0087223	circRNA	0.524641946	0.008765722
hsa_circ_0061339	circRNA	0.524361113	0.003346558
hsa_circ_0067033	circRNA	0.524197963	0.004172526
ACKR3	mRNA	0.52391556	0.038998391

SPTY2D1	mRNA	0.523491433	2.70E-05
hsa_circ_0023935	circRNA	0.522986188	0.007811709
hsa_circ_0024719	circRNA	0.522854994	0.015731173
SLK	mRNA	0.522706611	0.045326452
hsa_circ_0074807	circRNA	0.522625918	0.019899479
HTT	mRNA	0.52248901	5.31E-05
PIR	mRNA	0.521766728	0.022804419
ORMDL3	mRNA	0.521590106	5.80E-05
DTX3L	mRNA	0.521216586	0.001281937
TRIM35	mRNA	0.521120234	6.01E-05
ADPRH	mRNA	0.520579647	4.85E-04
hsa_circ_0051989	circRNA	0.520222207	0.033639362
hsa_circ_0078439	circRNA	0.520166638	0.004334107
RAB33B	mRNA	0.519365955	0.001147731
hsa_circ_0009020	circRNA	0.519364489	0.003142239
MOGS	mRNA	0.518912613	3.59E-04
hsa_circ_0003183	circRNA	0.518105264	0.025918147
TMED7-TICAM2	mRNA	0.517915067	1.85E-05
CPNE2	mRNA	0.517167899	8.11E-07
KLC2	mRNA	0.517068206	0.024489308
BNIP1	mRNA	0.516907303	6.67E-04
hsa_circ_0027462	circRNA	0.516471718	0.016969279
hsa_circ_0002885	circRNA	0.516127513	0.031508736
hsa_circ_0016769	circRNA	0.516057838	0.033066803
ANO6	mRNA	0.516020138	6.34E-04

hsa_circ_0101119	circRNA	0.515504571	0.003131674
SHROOM4	mRNA	0.514602108	7.68E-04
P3H1	mRNA	0.514418913	0.010875168
PHLDA3	mRNA	0.514091058	0.040043139
STEAP3	mRNA	0.513987912	0.030492088
SCAMP2	mRNA	0.513615498	5.46E-07
PCYT1A	mRNA	0.513182831	4.27E-06
LOC642852	lncRNA	0.513102062	0.005214513
S100A10	mRNA	0.51300805	0.01599232
BAK1	mRNA	0.512796898	0.00516974
C22orf23	mRNA	0.512674471	0.001964922
IFT27	mRNA	0.51251793	1.96E-04
PJA2	mRNA	0.512488141	1.75E-07
TECPR1	mRNA	0.512295504	6.99E-05
CD109	mRNA	0.511964973	0.014446378
CDYL2	mRNA	0.511651364	0.013714339
hsa_circ_0038984	circRNA	0.511643977	0.015111569
hsa_circ_0128358	circRNA	0.511258287	0.007994848
CDC42SE1	mRNA	0.511080926	0.004364412
LOC101927979	lncRNA	0.510907226	0.00346323
hsa_circ_0098199	circRNA	0.510785952	0.040164139
CENPBD1P1	pseudo	0.510585594	0.001149992
hsa_circ_0042617	circRNA	0.510270601	0.002380564
IRF7	mRNA	0.509707837	0.00511032
hsa_circ_0014892	circRNA	0.509595534	0.026992389

VPS37C	mRNA	0.509567601	0.00529158
hsa_circ_0105072	circRNA	0.509368145	0.030509234
hsa_circ_0075202	circRNA	0.508901047	0.035760882
DSTN	mRNA	0.50878024	1.57E-04
LOC101927809	lncRNA	0.507715975	0.034615593
hsa_circ_0121907	circRNA	0.507623618	0.012369028
EDEM1	mRNA	0.507410642	0.002275436
SNHG15	lncRNA	0.507193315	0.031111497
DEGS1	mRNA	0.506854002	2.82E-06
hsa_circ_0116686	circRNA	0.506470332	0.008622301
hsa_circ_0023342	circRNA	0.506415919	0.006385788
hsa_circ_0044441	circRNA	0.506306051	0.009295291
hsa_circ_0000976	circRNA	0.506233881	0.031014981
SEC23B	mRNA	0.506034661	5.46E-04
ZBTB11-AS1	lncRNA	0.505781546	0.0015953
hsa_circ_0031725	circRNA	0.50572921	0.006831727
hsa_circ_0014925	circRNA	0.505615093	9.50E-05
hsa_circ_0055389	circRNA	0.505492192	1.91E-04
KLHL2	mRNA	0.505472011	7.70E-06
hsa_circ_0000008	circRNA	0.5053653	0.005018172
RIOX2	mRNA	0.505320256	0.006975051
JOSD1	mRNA	0.505045711	0.00670701
UBN1	mRNA	0.504824596	6.06E-04
DAP	mRNA	0.504708162	1.34E-10
KIAA1671	mRNA	0.50395197	0.047019247

TMED2	mRNA	0.503881284	0.001436338
hsa_circ_0024767	circRNA	0.503712079	0.003627708
hsa_circ_0053047	circRNA	0.503350905	0.041046313
SNX9	mRNA	0.503283646	0.004889114
MPZL1	mRNA	0.503170525	0.001280216
hsa_circ_0038992	circRNA	0.503143455	0.0010897
hsa_circ_0004700	circRNA	0.503106494	0.013658273
hsa_circ_0119498	circRNA	0.502739519	0.006553758
ARCN1	mRNA	0.502646849	3.43E-05
hsa_circ_0050905	circRNA	0.502397117	0.03745765
hsa_circ_0065649	circRNA	0.502289663	3.83E-04
SEC14L2	mRNA	0.502181519	8.62E-06
BCL3	mRNA	0.501924556	0.014057404
NCDN	mRNA	0.501827907	0.012057252
IL6R	mRNA	0.501562166	0.001442639
ZNF398	mRNA	0.501009247	2.79E-05
ESRRA	mRNA	0.500469002	0.036754637
NXT1	mRNA	0.500211219	0.036968257
APOL1	mRNA	0.499540929	0.00667866
DNAJC2	mRNA	0.499536001	0.001663343
TMEM243	mRNA	0.499181412	1.22E-04
ZDHHC16	mRNA	0.499145316	0.002017158
hsa_circ_0007984	circRNA	0.498731154	0.030916745
NANS	mRNA	0.49861869	0.003179779
CBR1	mRNA	0.498484402	0.043707024

ZNF841	mRNA	0.498387068	2.19E-04
RABL3	mRNA	0.498353067	0.002455251
GDI1	mRNA	0.498198873	3.74E-05
NPDC1	mRNA	0.498083482	3.30E-06
TYRO3	mRNA	0.49768302	0.02509908
TMEM267	mRNA	0.497521123	0.007758782
DPM2	mRNA	0.497518574	0.013386303
TUT1	mRNA	0.497269441	1.81E-04
CYTH1	mRNA	0.497147693	0.003266078
PLPBP	mRNA	0.497051448	1.98E-07
NOL6	mRNA	0.496753663	0.024177724
hsa_circ_0011877	circRNA	0.496637706	0.014276655
ARHGEF3	mRNA	0.496324436	0.032231143
hsa_circ_0004882	circRNA	0.495600242	0.03697731
PIBF1	mRNA	0.495441204	2.63E-08
SLC2A13	mRNA	0.495205552	0.042481714
TBC1D24	mRNA	0.494884559	0.010483651
TUBB6	mRNA	0.494625541	0.032727052
ARFIP2	mRNA	0.494150734	1.89E-04
RIPOR1	mRNA	0.493548432	4.02E-06
hsa_circ_0020799	circRNA	0.493537215	0.018198725
CLINT1	mRNA	0.493487268	0.001506895
TPD52L2	mRNA	0.49332716	7.71E-07
SURF4	mRNA	0.493239329	0.00277115
hsa_circ_0124517	circRNA	0.493221886	0.044507398

DRAM1	mRNA	0.492762455	5.51E-04
ANKRD13D	mRNA	0.49259487	0.00168839
COG3	mRNA	0.491458373	3.00E-06
B3GNTL1	mRNA	0.491402433	0.013483886
hsa_circ_0084601	circRNA	0.491388301	0.040734076
DVL1	mRNA	0.491271759	0.008429503
HIVEP3	mRNA	0.491270531	8.27E-05
hsa_circ_0089542	circRNA	0.491230043	0.018262448
EEF2KMT	mRNA	0.49099923	0.011138188
hsa_circ_0059695	circRNA	0.49095864	0.002811776
NAA80	mRNA	0.490699053	0.028299413
PROSER3	mRNA	0.490482509	0.015991755
DPH3	mRNA	0.490410874	0.004304977
CIC	mRNA	0.489918763	0.003382681
BTG3	mRNA	0.489541091	0.034043983
B4GALT7	mRNA	0.489301562	0.003330194
ALDOA	mRNA	0.489288329	0.001453551
SCYL1	mRNA	0.489194419	5.28E-04
KDELC2	mRNA	0.488977012	0.002734992
hsa_circ_0067317	circRNA	0.488185487	0.00499562
PELO	mRNA	0.488139167	0.010020892
PTP4A1	mRNA	0.488017245	0.001985633
S1PR2	mRNA	0.487886802	0.033711074
STAM2	mRNA	0.487429854	1.64E-05
hsa_circ_0092075	circRNA	0.487106666	0.005180533

ABHD15	mRNA	0.486955227	0.007976783
hsa_circ_0061338	circRNA	0.486877228	0.014581404
hsa_circ_0007841	circRNA	0.486819026	0.02190769
hsa_circ_0064089	circRNA	0.486757334	0.032625784
hsa_circ_0085282	circRNA	0.486665116	0.009578232
DYNC1LI2	mRNA	0.48645278	6.92E-04
AMPD2	mRNA	0.486243635	0.017112845
HILPDA	mRNA	0.486136523	0.016357326
OXR1	mRNA	0.485826094	0.009120883
RASIP1	mRNA	0.485372972	4.22E-07
hsa_circ_0057016	circRNA	0.485363494	0.008913798
hsa_circ_0011994	circRNA	0.485179001	0.009325262
ZC3HAV1L	mRNA	0.485146218	0.011494938
hsa_circ_0085431	circRNA	0.485075845	0.035012283
hsa_circ_0024787	circRNA	0.484691391	0.035022915
TAP2	mRNA	0.484085718	6.00E-04
hsa_circ_0075311	circRNA	0.483980116	0.013956158
hsa_circ_0046237	circRNA	0.483879069	0.043791765
IKBKG	mRNA	0.483636166	0.006759386
hsa_circ_0051923	circRNA	0.483191439	0.007468776
hsa_circ_0055387	circRNA	0.483039768	6.02E-04
hsa_circ_0026895	circRNA	0.48284189	0.013255935
LOC100506544	lncRNA	0.482627834	0.044815957
RAB1A	mRNA	0.482553677	4.71E-05
LOC107987141	lncRNA	0.482551711	0.018528139

FKBP7	mRNA	0.482367601	0.022072044
TREX1	mRNA	0.482322127	0.035816474
CYB5B	mRNA	0.482144562	0.032015113
PRXL2B	mRNA	0.48204316	9.19E-07
hsa_circ_0007581	circRNA	0.482016437	0.020099604
hsa_circ_0002450	circRNA	0.481791884	0.010585962
SCFD1	mRNA	0.481438328	9.55E-08
hsa_circ_0110971	circRNA	0.481406969	8.23E-04
ARHGAP21	mRNA	0.48135771	1.90E-06
PI4K2B	mRNA	0.48131637	0.003533234
UTP6	mRNA	0.480590339	9.51E-04
hsa_circ_0090451	circRNA	0.480461915	0.001977901
NAAA	mRNA	0.480461654	0.001529997
PYGB	mRNA	0.479762983	1.84E-06
ZBTB7A	mRNA	0.479419328	9.07E-04
hsa_circ_0069778	circRNA	0.479384997	0.034163868
OSTF1	mRNA	0.479207273	0.016509196
hsa_circ_0083932	circRNA	0.479133481	0.030505626
hsa_circ_0109465	circRNA	0.479091218	0.038803904
GORAB	mRNA	0.479079868	0.003104675
SLC27A4	mRNA	0.478692186	0.00629434
CRLS1	mRNA	0.478592501	0.001382415
EPM2A	mRNA	0.478385975	0.036871972
ITGA3	mRNA	0.477716547	3.75E-04
hsa_circ_0031705	circRNA	0.477712746	0.00556922

C10orf88	mRNA	0.477459904	0.043762926
DOK4	mRNA	0.477246963	6.15E-04
hsa_circ_0085278	circRNA	0.477161213	0.045326933
CTU2	mRNA	0.477063796	0.011193001
hsa_circ_0111190	circRNA	0.477060412	0.029819458
TMEM50B	mRNA	0.476764242	0.039065188
ARHGEF10	mRNA	0.476505362	0.034460082
LINC01465	lncRNA	0.476343034	0.033932816
hsa_circ_0000652	circRNA	0.476255218	0.048878554
TNIP2	mRNA	0.476046928	6.82E-04
hsa_circ_0044445	circRNA	0.47582668	0.038843756
DERL1	mRNA	0.475525712	0.001532215
SEL1L3	mRNA	0.475416666	0.018606332
PLEKHM1	mRNA	0.475211266	0.005967476
hsa_circ_0104862	circRNA	0.475210182	0.028816929
hsa_circ_0008895	circRNA	0.474824431	0.034667457
UBE2E2	mRNA	0.4747453	0.009190483
hsa_circ_0086620	circRNA	0.474741345	0.00130444
TEAD4	mRNA	0.474641366	0.007917534
hsa_circ_0128731	circRNA	0.473838716	0.017145413
SLC9B2	mRNA	0.473615234	0.004708654
hsa_circ_0074676	circRNA	0.473256807	0.011881443
GNL2	mRNA	0.473255717	0.011511807
SERF2	mRNA	0.47321536	1.23E-04
ROM1	mRNA	0.472947337	0.038983736

MANCR	lncRNA	0.472764579	0.019942874
hsa_circ_0024789	circRNA	0.472362237	0.019745806
RTN4	mRNA	0.472082324	0.024177724
hsa_circ_0014932	circRNA	0.471862599	0.002156578
GIMAP6	mRNA	0.47128268	1.08E-05
hsa_circ_0101146	circRNA	0.470882021	0.03624142
HMOX2	mRNA	0.470677387	0.010236875
SEC61A1	mRNA	0.470325657	2.11E-04
GCN1	mRNA	0.469758459	0.009472065
TSPAN14	mRNA	0.469751718	1.88E-05
hsa_circ_0125390	circRNA	0.469311322	0.018059318
hsa_circ_0079716	circRNA	0.469188048	0.040380972
TCP11L1	mRNA	0.469158778	2.65E-07
hsa_circ_0074659	circRNA	0.469037334	0.013624672
hsa_circ_0024975	circRNA	0.468927588	0.045755063
hsa_circ_0073984	circRNA	0.468923413	0.037561177
hsa_circ_0055388	circRNA	0.468829283	0.001847128
PTRH2	mRNA	0.468824212	0.012645233
CKAP4	mRNA	0.46870585	0.020730735
COG5	mRNA	0.468685694	3.85E-05
LYSMD3	mRNA	0.468523177	0.00208844
GRASP	mRNA	0.468504138	0.008235778
hsa_circ_0023894	circRNA	0.468262232	0.034752977
FICD	mRNA	0.468116752	0.001616135
hsa_circ_0056489	circRNA	0.467667369	0.044677584

ALAS1	mRNA	0.467497401	0.019888109
hsa_circ_0123690	circRNA	0.467453843	0.035329212
ZNF805	mRNA	0.467293504	2.01E-05
NAT9	mRNA	0.467213597	8.07E-06
hsa_circ_0079726	circRNA	0.466664792	0.01852778
hsa_circ_0063142	circRNA	0.466458056	0.015642334
hsa_circ_0008982	circRNA	0.466409438	0.0173046
TFE3	mRNA	0.466226578	3.59E-04
hsa_circ_0099944	circRNA	0.465998589	0.0311716
ENSA	mRNA	0.465798126	1.29E-05
RARS	mRNA	0.465445873	0.00127287
hsa_circ_0057018	circRNA	0.465047691	0.012833241
hsa_circ_0008702	circRNA	0.464778658	0.010894072
hsa_circ_0024710	circRNA	0.464414285	0.015381553
hsa_circ_0005589	circRNA	0.464201521	0.003662344
SEC16A	mRNA	0.464071118	0.003437504
PCAT19	lncRNA	0.463595063	0.007174106
SBNO2	mRNA	0.463393713	2.77E-04
MPC1	mRNA	0.463152536	0.001368254
DMPK	mRNA	0.463004708	0.015616169
hsa_circ_0116690	circRNA	0.462969432	0.014178345
hsa_circ_0004406	circRNA	0.462596858	0.039289188
TIMMDC1	mRNA	0.462530854	0.001247235
CYB5A	mRNA	0.462494353	0.001168871
hsa_circ_0089559	circRNA	0.462157296	0.012022164

KEAP1	mRNA	0.461974923	0.001289198
LOC100996724	pseudo	0.461916528	0.028319775
RAB11A	mRNA	0.461497982	4.25E-07
hsa_circ_0089362	circRNA	0.461384206	0.030713784
hsa_circ_0027734	circRNA	0.461233038	0.029729905
hsa_circ_0056007	circRNA	0.461125331	0.009992095
ZC3H12A	mRNA	0.461108771	0.043886609
ATP6V0A2	mRNA	0.460840725	0.004726018
hsa_circ_0008368	circRNA	0.46046912	0.041610007
FEZ1	mRNA	0.459697521	0.001489154
TOMM5	mRNA	0.459599681	0.028155281
MAP4	mRNA	0.459504074	2.64E-05
hsa_circ_0038971	circRNA	0.459458538	0.041126301
hsa_circ_0055144	circRNA	0.458894852	0.044338902
ZFPL1	mRNA	0.458456153	5.75E-04
SCML1	mRNA	0.458319991	0.039635591
ABCA2	mRNA	0.458013869	1.25E-04
TAP1	mRNA	0.457975632	0.012115963
SH3BP2	mRNA	0.457950766	0.029196052
ZNF25	mRNA	0.45766603	0.031111497
LOC105369623	lncRNA	0.457636483	0.014828176
JMJD4	mRNA	0.457439055	0.032786776
PUM3	mRNA	0.456814489	0.045326452
SMCHD1	mRNA	0.456694953	0.006589546
PDE8A	mRNA	0.456384048	0.02269353

RAP1A	mRNA	0.455810828	0.030067911
hsa_circ_0014201	circRNA	0.455671397	0.021390664
SGSM3	mRNA	0.455571111	4.59E-05
hsa_circ_0104875	circRNA	0.455282556	0.012722816
ZNF438	mRNA	0.455110256	0.016207522
UGGT2	mRNA	0.454867587	0.014328254
WDR44	mRNA	0.454662348	6.97E-04
hsa_circ_0128546	circRNA	0.454428735	0.025511133
hsa_circ_0046122	circRNA	0.453769592	0.043232449
hsa_circ_0089363	circRNA	0.453767257	0.016799152
hsa_circ_0096138	circRNA	0.453493176	0.038206071
NAB2	mRNA	0.453169988	0.016605846
ICMT	mRNA	0.45316809	0.019942874
FBXO8	mRNA	0.453162518	5.07E-05
hsa_circ_0084762	circRNA	0.45315668	0.017418583
ANXA7	mRNA	0.453035473	0.002418203
CSRNP1	mRNA	0.452948502	0.011525857
hsa_circ_0006694	circRNA	0.452851961	0.011556589
TMEM41B	mRNA	0.45211564	0.005036647
hsa_circ_0025626	circRNA	0.451941445	0.028796401
hsa_circ_0058214	circRNA	0.451865158	0.02290569
RNF145	mRNA	0.451798144	0.029036492
hsa_circ_0090452	circRNA	0.451759787	0.006821813
RIN2	mRNA	0.451696528	0.00948554
TMEM231	mRNA	0.451517539	0.010315771

ATOX1	mRNA	0.45069378	3.70E-05
hsa_circ_0061345	circRNA	0.449484521	0.022105883
EGFL7	mRNA	0.449051188	0.00975883
hsa_circ_0126057	circRNA	0.448407208	0.048600557
PRCP	mRNA	0.448266681	0.002135947
hsa_circ_0002948	circRNA	0.448265885	0.012372677
hsa_circ_0024338	circRNA	0.448184285	0.011080071
hsa_circ_0037742	circRNA	0.448120285	0.015896611
LARS	mRNA	0.447590087	0.025192459
DAG1	mRNA	0.447356475	5.45E-09
MTCL1	mRNA	0.447150126	0.041703067
ZNF622	mRNA	0.447139753	0.001211464
DNAJC24	mRNA	0.446999698	0.043742211
hsa_circ_0003029	circRNA	0.446099209	0.009741082
ZMYND19	mRNA	0.445155012	0.013224474
MRPL33	mRNA	0.444933406	0.032250579
KIF26A	mRNA	0.444895763	0.038417591
DDA1	mRNA	0.444512996	0.009391378
INCA1	mRNA	0.444486239	0.04048403
hsa_circ_0038989	circRNA	0.444261226	0.014290402
PDLIM7	mRNA	0.444238328	0.02180423
SSR1	mRNA	0.44400039	0.013586703
ITPRID2	mRNA	0.443995144	5.41E-05
OSTC	mRNA	0.443991735	0.001184382
GNA12	mRNA	0.443783753	2.15E-04

hsa_circ_0019716	circRNA	0.443449553	0.034104706
NFIL3	mRNA	0.443388882	0.009318079
TMED3	mRNA	0.443333348	0.001051412
NOL3	mRNA	0.442915091	0.041799331
hsa_circ_0063088	circRNA	0.442836685	0.024266099
MICALL1	mRNA	0.442792595	4.71E-04
RAB10	mRNA	0.442691007	3.26E-04
L3MBTL3	mRNA	0.442686385	0.011097625
hsa_circ_0001006	circRNA	0.442624338	0.010895491
CMPK1	mRNA	0.442210275	0.00899248
hsa_circ_0023933	circRNA	0.442085658	0.03720775
FASTK	mRNA	0.441439094	3.83E-04
MALINC1	lncRNA	0.44138052	0.005763294
WDR5B	mRNA	0.441158511	7.17E-04
hsa_circ_0095840	circRNA	0.441111813	0.039944272
MDM2	mRNA	0.440657275	0.020738187
SLC15A3	mRNA	0.440083447	0.04579236
TNKS1BP1	mRNA	0.439698974	0.001039687
ARHGEF1	mRNA	0.439462207	0.005135036
TMEM39A	mRNA	0.439237201	9.30E-04
NDUFAF2	mRNA	0.438914608	0.018891832
DIS3	mRNA	0.438780726	5.75E-04
ZFP69	mRNA	0.438696614	0.048851093
SERP1	mRNA	0.438690164	0.00462834
PHPT1	mRNA	0.43842694	0.01747924

ZNF460	mRNA	0.438118186	0.005287351
BBS7	mRNA	0.438009212	7.07E-05
RABAC1	mRNA	0.437690388	7.39E-04
PSMG3	mRNA	0.437528783	0.018386459
hsa_circ_0116538	circRNA	0.43746181	0.016509994
MTF1	mRNA	0.436819049	0.001557683
CSNK1E	mRNA	0.436578915	0.037925278
hsa_circ_0084757	circRNA	0.43574934	0.012488638
C7orf43	mRNA	0.435628399	0.008455459
hsa_circ_0058641	circRNA	0.435545953	0.032607033
SPRED2	mRNA	0.434885093	0.001954751
PICALM	mRNA	0.434865378	0.012255854
OPA3	mRNA	0.434755971	0.00272915
MAP3K13	mRNA	0.434340868	0.006884817
TRIQK	mRNA	0.434196542	0.001522567
hsa_circ_0009358	circRNA	0.434114036	0.032625784
RPS6KC1	mRNA	0.433906861	0.003520179
JAM3	mRNA	0.433698764	0.032629923
MVP	mRNA	0.433675782	0.045835589
TNFRSF10B	mRNA	0.433660646	0.001677625
hsa_circ_0038982	circRNA	0.433383013	0.008545687
LOC105373786	lncRNA	0.432931054	0.005716891
hsa_circ_0013691	circRNA	0.432807718	0.022231474
ASPH	mRNA	0.432429237	0.013906966
EIF2AK1	mRNA	0.432424337	0.022509946

RAB5IF	mRNA	0.432056584	0.004971314
SORBS3	mRNA	0.432014444	1.04E-04
IFITM3	mRNA	0.431891046	0.041797072
NAPG	mRNA	0.430676191	0.039931304
SELENOK	mRNA	0.430625054	0.005957666
TRAM1	mRNA	0.430607649	2.45E-04
hsa_circ_0110689	circRNA	0.43023505	0.022652738
hsa_circ_0131796	circRNA	0.430086928	0.037503325
FNDC4	mRNA	0.429787855	4.81E-04
hsa_circ_0004733	circRNA	0.428872513	0.01852778
DENND4B	mRNA	0.428762489	0.009892516
PECAM1	mRNA	0.428721694	0.003012376
ABLIM3	mRNA	0.428454161	0.048638348
ABCC10	mRNA	0.428352207	8.95E-07
TFG	mRNA	0.427801784	0.001505002
MNT	mRNA	0.427472009	3.04E-04
IARS	mRNA	0.427433915	0.048551258
PGRMC2	mRNA	0.426540353	1.97E-04
hsa_circ_0023925	circRNA	0.426491732	0.027848088
hsa_circ_0059700	circRNA	0.426460334	0.032029771
LOC105370792	lncRNA	0.426143694	2.79E-05
NFE2L2	mRNA	0.425999208	0.006849072
hsa_circ_0061347	circRNA	0.425998829	0.027373375
TRIOBP	mRNA	0.425940873	0.006008657
hsa_circ_0061341	circRNA	0.425912246	0.01856113

hsa_circ_0038980	circRNA	0.425847688	0.015797859
hsa_circ_0089560	circRNA	0.424680947	0.00677559
MIDN	mRNA	0.424283032	0.030700405
hsa_circ_0002590	circRNA	0.424231329	0.042689432
FSCN1	mRNA	0.424150158	0.023822355
G6PC3	mRNA	0.424098063	0.007912167
hsa_circ_0061346	circRNA	0.423959026	0.021221587
DIAPH1	mRNA	0.423553319	1.33E-04
PRPF3	mRNA	0.423426483	2.21E-04
hsa_circ_0061518	circRNA	0.423079948	0.041476854
KTI12	mRNA	0.423027044	0.022332447
SLC37A3	mRNA	0.422890469	0.023490021
SMIM13	mRNA	0.422856169	0.044911993
NBL1	mRNA	0.422467401	0.001645054
CTTN	mRNA	0.422356059	0.007918357
SECISBP2	mRNA	0.421242983	0.018082745
ZDHHC9	mRNA	0.421094985	2.63E-05
PLPP5	mRNA	0.420896918	0.022566656
YIPF5	mRNA	0.42087677	0.001153321
LMAN1	mRNA	0.420452788	0.009082399
hsa_circ_0060035	circRNA	0.420208069	0.040594967
EML1	mRNA	0.419974053	4.50E-04
PLEKHB2	mRNA	0.419363612	0.022742139
hsa_circ_0063686	circRNA	0.419181397	0.009390125
hsa_circ_0018809	circRNA	0.418920419	0.028997077

TMEM165	mRNA	0.418902416	0.002125202
TMEM127	mRNA	0.4187886	8.83E-04
LRRC8A	mRNA	0.418476705	1.59E-04
ANKRD11	mRNA	0.417978734	0.0026015
EP400P1	pseudo	0.417847633	0.00803242
BMS1P20	pseudo	0.417419797	0.009675607
ILK	mRNA	0.417335381	3.75E-04
hsa_circ_0123982	circRNA	0.417214904	0.02395251
hsa_circ_0104326	circRNA	0.416692982	0.026450321
MPHOSPH10	mRNA	0.416368159	4.25E-04
SBDS	mRNA	0.416304956	5.22E-06
MORC4	mRNA	0.416142114	5.60E-05
MGAT4B	mRNA	0.415593361	0.004642327
PTPDC1	mRNA	0.415441876	0.0098656
ACOT9	mRNA	0.415414637	3.24E-05
hsa_circ_0059638	circRNA	0.41536731	0.025793774
KCTD21	mRNA	0.415305788	5.07E-05
DEAF1	mRNA	0.415206721	0.00595508
CAPN10	mRNA	0.415160162	0.025846037
MGAT1	mRNA	0.415012642	4.04E-10
hsa_circ_0082150	circRNA	0.414908839	0.037941183
NAGK	mRNA	0.414667701	2.70E-05
PIAS3	mRNA	0.414415403	8.35E-05
LONRF1	mRNA	0.414219006	0.032536043
SLC35F5	mRNA	0.414193371	0.001454983

APP	mRNA	0.413657947	0.003704866
CCDC9	mRNA	0.413118434	5.95E-04
DNASE1L1	mRNA	0.412906795	0.012255854
SEC22B	mRNA	0.412539279	0.001790427
TPM3	mRNA	0.412494785	0.025461186
hsa_circ_0008201	circRNA	0.41234406	0.031311138
HGS	mRNA	0.412139862	0.002555047
hsa_circ_0059057	circRNA	0.411934995	0.005019517
OSBP	mRNA	0.411783524	4.31E-07
NDUFA12	mRNA	0.411613776	0.043527154
hsa_circ_0099896	circRNA	0.411242624	0.036344003
ITGB1BP1	mRNA	0.410860807	1.53E-05
hsa_circ_0104874	circRNA	0.410775771	0.007498735
hsa_circ_0115726	circRNA	0.410619773	0.01102465
RHOA	mRNA	0.410491859	2.58E-05
VPS25	mRNA	0.409879702	0.004848848
IQGAP1	mRNA	0.409573879	9.24E-06
hsa_circ_0089583	circRNA	0.409536031	0.049644284
hsa_circ_0020625	circRNA	0.409195659	0.046358415
MAPK7	mRNA	0.409158767	8.76E-04
KIF1BP	mRNA	0.409041038	0.019017107
FBXL12	mRNA	0.40787212	0.0067565
TRIM47	mRNA	0.407037393	0.038964917
LIG3	mRNA	0.406767199	0.00863567
NFKBIA	mRNA	0.406471578	0.025846037

FTSJ1	mRNA	0.406359474	0.014817409
CAMTA2	mRNA	0.406246903	4.22E-06
hsa_circ_0038976	circRNA	0.405098269	0.023841572
ARNTL2	mRNA	0.404944743	0.019942874
TGFB1I1	mRNA	0.404598164	0.015946832
SYVN1	mRNA	0.404224374	3.11E-04
TMEM102	mRNA	0.403960035	0.004771805
MORN4	mRNA	0.403326261	0.039888404
BRPF1	mRNA	0.403019875	0.005532865
RTL6	mRNA	0.402995922	0.00103846
hsa_circ_0088735	circRNA	0.402790723	0.026728444
WBP2	mRNA	0.402604009	0.013761837
NUMB	mRNA	0.40229342	0.001994174
hsa_circ_0061336	circRNA	0.402156012	0.032625784
CAMK1	mRNA	0.401649809	0.009608087
hsa_circ_0068347	circRNA	0.401347942	0.046966717
hsa_circ_0087224	circRNA	0.401189151	0.042694697
EIF4G1	mRNA	0.401122023	0.006025342
ALG2	mRNA	0.401098653	0.005737114
MYO1C	mRNA	0.401031947	0.048929647
AGPAT4	mRNA	0.400863124	0.003818834
INPP1	mRNA	0.400381262	0.014965972
ARHGDIA	mRNA	0.400124125	0.039687919
TBC1D17	mRNA	0.400103765	0.004888155
C16orf58	mRNA	0.399982647	0.005615439

TP53I13	mRNA	0.399765466	0.023862226
RNPEPL1	mRNA	0.399658853	8.18E-04
hsa_circ_0128761	circRNA	0.399449903	0.039808867
hsa_circ_0074887	circRNA	0.399392834	0.039825528
TP53	mRNA	0.399171744	0.008352324
CHST7	mRNA	0.398764371	0.016650356
KIAA0232	mRNA	0.398718517	0.035510131
KIF3C	mRNA	0.398550692	0.020169963
hsa_circ_0036792	circRNA	0.397734298	0.025770124
TSPO	mRNA	0.397609937	3.00E-04
TBC1D20	mRNA	0.397486437	0.002635191
hsa_circ_0036810	circRNA	0.397464825	0.010133565
hsa_circ_0115727	circRNA	0.397176947	0.035675516
CYFIP1	mRNA	0.397019278	0.008196781
HOXB6	mRNA	0.396947949	0.012176762
ENTPD4	mRNA	0.396916913	0.001105621
ZNF550	mRNA	0.396780285	0.022439881
CMAS	mRNA	0.396669888	0.032231143
SOX13	mRNA	0.3965659	0.036648341
TBC1D23	mRNA	0.396286547	0.00146542
hsa_circ_0003203	circRNA	0.395127986	0.049711721
MIER2	mRNA	0.394171483	0.028771981
SLC38A10	mRNA	0.393964402	0.007463972
WDR92	mRNA	0.393696551	0.028368635
LZTR1	mRNA	0.393477477	0.001463375

ZCCHC9	mRNA	0.393434101	0.002280972
RNF13	mRNA	0.392942234	0.013696402
TWF2	mRNA	0.392749355	0.02942904
NR1H2	mRNA	0.392694684	0.001651942
SOGA1	mRNA	0.392632439	0.008259817
TPCN2	mRNA	0.39171327	9.18E-04
POR	mRNA	0.391607939	0.011231437
KIAA0100	mRNA	0.391522479	0.003183918
STK10	mRNA	0.391369965	0.040527257
ZNF672	mRNA	0.391170487	0.021897048
HGSNAT	mRNA	0.390877265	0.003567727
C9orf16	mRNA	0.390649971	0.023566248
hsa_circ_0016554	circRNA	0.390075129	0.028402708
SEH1L	mRNA	0.389146146	0.006195271
TOR4A	mRNA	0.388828553	0.025606964
YIPF3	mRNA	0.388813279	1.57E-04
STT3A	mRNA	0.388610282	0.003149735
hsa_circ_0084756	circRNA	0.387737089	0.017171743
PIP5K1C	mRNA	0.387585032	0.042476974
hsa_circ_0028517	circRNA	0.387409121	0.039204768
MOB2	mRNA	0.387069243	0.008005215
ARHGAP23	mRNA	0.38698754	1.67E-04
PWP1	mRNA	0.386901416	6.12E-04
hsa_circ_0009004	circRNA	0.38656013	0.035652366
PRKAA2	mRNA	0.386311194	0.025368155

ARMCX3	mRNA	0.385940208	7.94E-05
KCTD20	mRNA	0.385811841	0.021657633
hsa_circ_0026722	circRNA	0.385604741	0.034581602
MYL12A	mRNA	0.385591251	0.004289343
DOCK9	mRNA	0.385208636	0.006011417
hsa_circ_0096190	circRNA	0.385017744	0.025765131
MGRN1	mRNA	0.384594385	7.35E-05
RUBCN	mRNA	0.384241977	5.57E-04
hsa_circ_0093430	circRNA	0.383763916	0.031963096
TMEM250	mRNA	0.383338387	0.02704882
TSPAN18	mRNA	0.382735532	0.005031033
DDX41	mRNA	0.382500554	0.012915508
AZIN1	mRNA	0.382485435	0.013521093
PPARA	mRNA	0.382359658	0.033969552
ITPKC	mRNA	0.382279245	0.006781445
TATDN2	mRNA	0.382261653	0.037236186
ELMO2	mRNA	0.381650988	0.009946407
RAB4B	mRNA	0.381189549	0.007931052
NAA10	mRNA	0.381073575	0.017793374
STYX	mRNA	0.380924467	8.18E-04
hsa_circ_0006869	circRNA	0.380260773	0.040164139
hsa_circ_0104880	circRNA	0.380194444	0.046354475
hsa_circ_0045948	circRNA	0.380161943	0.043504817
USP36	mRNA	0.379925741	0.016257868
MICB	mRNA	0.379898691	0.039555095

GLYR1	mRNA	0.379259056	0.001514026
GOLPH3	mRNA	0.378659953	6.74E-04
SF3B2	mRNA	0.37819768	0.008429503
ARPC2	mRNA	0.378147059	5.24E-05
SRF	mRNA	0.37788839	0.001331126
ANAPC5	mRNA	0.377105172	0.003957536
CBARP	mRNA	0.376739152	0.00737943
FAM3C	mRNA	0.376490707	0.01568587
SACM1L	mRNA	0.376315371	0.002444276
ARL3	mRNA	0.375861744	0.005535427
hsa_circ_0137075	circRNA	0.375673554	0.027584665
CCDC149	mRNA	0.375237951	0.005094157
RNF7	mRNA	0.374344841	6.58E-04
USP14	mRNA	0.373916473	0.007854127
RPN1	mRNA	0.373837573	0.011505212
ELOVL5	mRNA	0.373428522	0.032084303
BLOC1S4	mRNA	0.373142107	0.012053019
VASP	mRNA	0.37298794	0.003927401
MOV10	mRNA	0.372810932	0.014049446
RAB5B	mRNA	0.372657097	1.10E-06
PKNOX1	mRNA	0.371998067	0.011514055
hsa_circ_0061342	circRNA	0.371582244	0.034890377
JTB	mRNA	0.371436343	0.019873844
TSPAN9	mRNA	0.369792351	0.003638431
RRP36	mRNA	0.369633019	0.003710673

TMEM63B	mRNA	0.369377433	0.035960441
LZTFL1	mRNA	0.369293347	7.83E-04
RP2	mRNA	0.369290492	0.019370909
G3BP1	mRNA	0.369095773	0.044336582
SUCO	mRNA	0.368907849	0.009568072
hsa_circ_0012869	circRNA	0.368752135	0.027175326
ECE1	mRNA	0.368627079	8.81E-04
ASCC3	mRNA	0.368306393	0.034688307
JARID2	mRNA	0.368147367	0.002161888
CXorf40B	mRNA	0.367887166	0.009022441
CRYBG3	mRNA	0.367618169	0.006676849
SNHG12	lncRNA	0.367499663	0.03345367
FAM129B	mRNA	0.367420901	0.014754949
hsa_circ_0069779	circRNA	0.367267107	0.039723101
JAK1	mRNA	0.366822243	0.001404465
ATP5MD	mRNA	0.366206739	0.01531839
hsa_circ_0000651	circRNA	0.364765728	0.031230989
SAMD4B	mRNA	0.364296621	0.04025715
ZNF276	mRNA	0.364234722	0.024268502
IRF1	mRNA	0.36356387	0.005615439
GSTP1	mRNA	0.363467439	0.022804419
EIF1AD	mRNA	0.363450796	0.024071113
SDHAF2	mRNA	0.362956269	0.002596489
FAR1	mRNA	0.36276737	4.19E-04
TTC19	mRNA	0.362470866	0.001678114

YME1L1	mRNA	0.362264377	7.13E-04
hsa_circ_0051254	circRNA	0.361967851	0.023387812
C1orf174	mRNA	0.361570925	0.045644608
IFNGR2	mRNA	0.361130361	0.012504702
RAB2A	mRNA	0.361033415	0.007758782
TPRN	mRNA	0.360621695	0.032581072
TXNDC9	mRNA	0.360005059	0.037341113
BAG5	mRNA	0.359487264	6.85E-06
HDAC6	mRNA	0.358079674	0.010434201
ALKBH5	mRNA	0.357146464	0.001280235
hsa_circ_0051251	circRNA	0.356774417	0.038389557
hsa_circ_0065344	circRNA	0.356546344	0.045502678
EIF4E2	mRNA	0.355788841	0.003965189
ZNF865	mRNA	0.3552278	0.004226613
ASB13	mRNA	0.35515898	0.019942874
MDC1	mRNA	0.354819439	6.61E-04
REEP5	mRNA	0.354442671	0.001784145
ITCH	mRNA	0.354167763	1.60E-04
PIK3CB	mRNA	0.353550696	0.017401456
OIP5-AS1	lncRNA	0.352440499	8.86E-04
ADIPOR1	mRNA	0.352266151	0.015796028
ARL8A	mRNA	0.351472823	0.015431599
hsa_circ_0009348	circRNA	0.350948103	0.040165538
KIF1C	mRNA	0.35070681	0.006641705
KLC1	mRNA	0.350556004	0.01086994

KIF3B	mRNA	0.350478645	0.030826699
ELOF1	mRNA	0.349804586	0.01234666
TRIM11	mRNA	0.349754406	0.035960441
DMWD	mRNA	0.34905364	0.002190503
CAST	mRNA	0.348741994	0.011065263
SAYSD1	mRNA	0.348686936	0.006272016
hsa_circ_0036799	circRNA	0.34822125	0.024732774
NSUN4	mRNA	0.348195356	0.039706352
SETD7	mRNA	0.347778331	0.014894327
NRBP1	mRNA	0.347762464	0.007674985
STX5	mRNA	0.346708147	8.81E-05
RTL8B	mRNA	0.346495587	0.03398006
MRPL24	mRNA	0.346088637	0.034043983
PTPRA	mRNA	0.34485548	0.00145404
FAM76A	mRNA	0.344456971	0.023085156
YIPF6	mRNA	0.344035537	0.011764307
GOLGA4	mRNA	0.343927463	9.21E-04
DXO	mRNA	0.343425955	0.048241245
hsa_circ_0082132	circRNA	0.343323007	0.043450978
PUSL1	mRNA	0.342865749	0.043946723
LPIN2	mRNA	0.342564747	0.009022441
COPB1	mRNA	0.342401489	6.33E-04
LTN1	mRNA	0.341735227	0.026040743
INTS3	mRNA	0.34141476	0.001379534
NADSYN1	mRNA	0.340575547	0.010594175

RTL8A	mRNA	0.340502754	0.036320096
hsa_circ_0009353	circRNA	0.3402662	0.025604732
DNAJC1	mRNA	0.339760498	9.73E-04
PRDX1	mRNA	0.339456478	0.022933752
FAM8A1	mRNA	0.339136165	0.010578263
ARHGEF7	mRNA	0.33896887	0.007829821
GZF1	mRNA	0.338819959	0.002736684
LRP11	mRNA	0.338706665	0.027801143
SRRT	mRNA	0.338162845	0.043725503
FBXW5	mRNA	0.337880703	0.010862061
MAP7D1	mRNA	0.336317536	0.042925898
ZCCHC2	mRNA	0.334996091	0.001523283
RIOK1	mRNA	0.334540684	0.004877625
C14orf119	mRNA	0.334442302	8.69E-04
RAB3GAP2	mRNA	0.334187538	0.00131907
ARF3	mRNA	0.33406348	0.008566287
ZBTB11	mRNA	0.334042246	0.035808405
SCAMP3	mRNA	0.333721141	0.015435911
FERMT2	mRNA	0.33364073	0.001419054
NECAP2	mRNA	0.332821559	9.91E-05
AUP1	mRNA	0.332788878	0.033623355
NDUFA6	mRNA	0.332699734	0.048405168
ARHGAP26	mRNA	0.332692621	0.039236443
TM9SF1	mRNA	0.332296252	2.88E-04
SND1	mRNA	0.331601878	9.68E-04

TRIM52	mRNA	0.331286142	0.018137633
ZSCAN22	mRNA	0.330796547	0.043102269
hsa_circ_0045947	circRNA	0.330547818	0.04720965
REXO2	mRNA	0.330414254	0.005077536
MBNL1	mRNA	0.329870357	0.002360524
MON2	mRNA	0.329195676	0.006010948
TMPPE	mRNA	0.329097206	0.04771321
SLC30A4	mRNA	0.328642038	0.007527003
KLF7	mRNA	0.328627003	0.027299334
ECSCR	mRNA	0.327766172	0.030120489
BMS1	mRNA	0.32773598	0.021549386
UBFD1	mRNA	0.327614469	0.005013664
GAS6	mRNA	0.327065244	0.048475951
UBAP2	mRNA	0.326613541	0.03885033
hsa_circ_0010503	circRNA	0.325065895	0.0405317
TADA2B	mRNA	0.324094582	0.039421244
MAPKAPK2	mRNA	0.323940045	0.005936198
ATG9A	mRNA	0.3233999	7.96E-05
POFUT2	mRNA	0.323337154	0.048880481
GOSR1	mRNA	0.323043639	0.011166975
PCMT1	mRNA	0.32242072	0.035205967
ARL2BP	mRNA	0.320640135	0.009118387
SKAP2	mRNA	0.32031079	0.010862061
CCNYL1	mRNA	0.320232744	0.040195738
CEP104	mRNA	0.319908906	0.045476539

ORMDL2	mRNA	0.319320454	0.034720285
TOM1L2	mRNA	0.319043392	5.82E-05
EPRS	mRNA	0.318305444	0.011338423
hsa_circ_0119142	circRNA	0.318126271	0.042415816
HPS6	mRNA	0.317753782	0.026856889
VPS4A	mRNA	0.316466126	0.003487821
RAVER1	mRNA	0.316386162	0.043504369
DUSP14	mRNA	0.315895412	0.036705654
STARD7-AS1	lncRNA	0.314911094	0.022258826
RER1	mRNA	0.314867678	0.011175941
TRAPPC10	mRNA	0.313853011	0.042925898
NRBF2	mRNA	0.313604585	0.018209005
PJA1	mRNA	0.313536088	0.002196855
ZDHHC5	mRNA	0.313023159	0.005663409
ARL6IP6	mRNA	0.313019436	0.010524061
INTS2	mRNA	0.313002015	0.003967194
USP35	mRNA	0.311229386	0.030029222
LRFN3	mRNA	0.311196028	0.007420802
FEM1C	mRNA	0.309890412	0.049022729
HEATR6	mRNA	0.30966655	0.025527781
NDST2	mRNA	0.308403512	5.48E-04
ATXN7	mRNA	0.308364057	0.026053172
AP1M1	mRNA	0.308360772	0.049805724
GNB1	mRNA	0.308123666	0.004178399
TNK2	mRNA	0.308079071	0.02126841

JKAMP	mRNA	0.307965369	0.023798652
NME6	mRNA	0.306822069	0.02484857
FAM219B	mRNA	0.306524357	0.01297433
LOC648987	lncRNA	0.305691209	0.023173159
LPGAT1	mRNA	0.304910003	0.008650566
SEC22C	mRNA	0.304518385	0.001514026
CC2D1B	mRNA	0.3044319	0.001880193
FUT4	mRNA	0.304365949	0.003314318
SS18	mRNA	0.30425556	0.002991312
DDX1	mRNA	0.304062276	0.032231143
RNF187	mRNA	0.302818012	0.017934969
EIF5B	mRNA	0.302786796	0.021024861
RAB11FIP5	mRNA	0.302344456	0.003250328
SUPT5H	mRNA	0.302324994	2.40E-04
KIF1B	mRNA	0.301324573	0.025039079
SETD3	mRNA	0.301236539	0.012098677
SLC35B3	mRNA	0.299948436	0.017190381
SOX7	mRNA	0.299773474	0.023745296
TDRD7	mRNA	0.299603325	0.011065263
SYNGR2	mRNA	0.299300622	0.043778336
ASCC2	mRNA	0.298941387	0.034531151
TRIM21	mRNA	0.298895893	0.015092764
DNAJC5	mRNA	0.298309497	0.017664578
EIF2B5	mRNA	0.297858187	0.020870324
SEPT2	mRNA	0.297092673	0.033390933

PI4K2A	mRNA	0.296559005	0.033401859
S1PR1	mRNA	0.295063813	0.001795781
SHC1	mRNA	0.294667703	4.62E-07
UTP14C	mRNA	0.293948765	0.007718722
POMK	mRNA	0.293829953	0.048301201
ANO10	mRNA	0.293020194	0.008045418
RFX1	mRNA	0.292915077	0.018824683
MIA2	mRNA	0.29262026	0.039254245
ZBTB4	mRNA	0.292100888	0.021897048
EXOC8	mRNA	0.291602193	0.045716519
PSMD1	mRNA	0.290268476	0.045702171
EPM2AIP1	mRNA	0.289924934	0.048846429
HERPUD2	mRNA	0.289881235	0.00127169
RNF216	mRNA	0.289856995	0.001208545
TIMP2	mRNA	0.289604988	0.020713407
CDC37	mRNA	0.289526263	0.037300508
FBXO34	mRNA	0.289020907	0.013362755
PKD1	mRNA	0.288590769	0.013634312
MORF4L1	mRNA	0.288575743	0.004249767
ST3GAL2	mRNA	0.288497569	0.002485598
INO80	mRNA	0.287487813	0.019310486
GNPDA2	mRNA	0.287129195	0.04048403
PSMA5	mRNA	0.286151433	0.032250579
DYNLL1	mRNA	0.285502366	0.027299334
R3HDM4	mRNA	0.285166559	0.006879904

BDP1	mRNA	0.283946362	0.017385218
TMEM8A	mRNA	0.28381141	0.016961857
PDPK1	mRNA	0.283632269	0.009860757
ZNF333	mRNA	0.282443179	0.048209966
UBE2O	mRNA	0.281662063	0.017860472
COPZ1	mRNA	0.281193207	0.01599232
YEATS2	mRNA	0.280909405	0.0434775
F8A1	mRNA	0.280555927	0.038630503
NAA35	mRNA	0.279437282	0.001478237
PITPNA	mRNA	0.278915722	0.020303228
PIP4P1	mRNA	0.278231763	0.012406364
ATL3	mRNA	0.278161893	4.87E-04
DDX5	mRNA	0.276533828	0.017101906
SLTM	mRNA	0.276257857	8.39E-04
TRIP4	mRNA	0.276153756	0.022118891
MKRN2	mRNA	0.274983934	0.047529758
EIF4ENIF1	mRNA	0.273340192	0.001377555
RIPK1	mRNA	0.273301529	6.18E-04
SLC35A4	mRNA	0.272218165	0.01318047
TUT7	mRNA	0.271379359	0.007625956
ZNF512	mRNA	0.270894347	0.013112261
STAT3	mRNA	0.270351948	0.026856889
PRKAB1	mRNA	0.269934497	0.017451213
ZCCHC8	mRNA	0.269564833	0.01747924
PIKFYVE	mRNA	0.268148505	0.035808405

RAB40C	mRNA	0.267216416	0.018136338
NCOA5	mRNA	0.267075557	0.049014973
SEC24C	mRNA	0.266204517	0.00756836
LYPLA2	mRNA	0.266001353	0.015641556
TPRG1L	mRNA	0.264035941	0.017853845
FYN	mRNA	0.263697254	0.021772846
MFNG	mRNA	0.262011055	0.01086994
EMC3	mRNA	0.261906978	0.033981929
PTP4A2	mRNA	0.261020494	0.039635591
NT5DC3	mRNA	0.260832145	0.049410122
STIMATE	mRNA	0.2585833	0.042925898
TXLNA	mRNA	0.258576558	0.016616606
SNIP1	mRNA	0.258177767	0.012843338
PPP1R15B	mRNA	0.258148608	0.043946723
ARPC4	mRNA	0.257178408	0.004888155
CHMP7	mRNA	0.254980677	0.025562983
ASB6	mRNA	0.254832651	0.032015113
ELOA	mRNA	0.254287673	0.049143042
ADCY6	mRNA	0.253517748	0.025715172
PPIC	mRNA	0.253050329	0.012597747
KAT5	mRNA	0.252975122	0.017027635
SH3GLB1	mRNA	0.250113946	0.003937194
PPP1R12C	mRNA	0.249970399	0.021455513
NFKB1	mRNA	0.249152655	0.018812867
CD47	mRNA	0.247949627	0.029552701

INTS5	mRNA	0.247597593	0.034470624
TERF2IP	mRNA	0.24708928	0.012705118
UBQLN2	mRNA	0.246751763	0.047505822
RETSAT	mRNA	0.246029304	0.007715758
TMEM259	mRNA	0.245226758	0.023790053
BCAP31	mRNA	0.245021451	0.016183039
TECPR2	mRNA	0.245006494	0.001797983
KRIT1	mRNA	0.244775473	0.011793361
GNB2	mRNA	0.24274016	0.031149809
AKAP9	mRNA	0.240942261	0.029136273
ZDHHC24	mRNA	0.240592345	0.024918378
MFSD10	mRNA	0.239513179	0.027277792
YWHAB	mRNA	0.239151235	0.034446758
FKBP15	mRNA	0.237276804	0.008103111
BMP1	mRNA	0.236320643	0.008260595
ATG13	mRNA	0.235234746	0.036173066
ATP2C1	mRNA	0.234818286	0.037673905
WDR47	mRNA	0.233660172	0.012646045
SLC39A9	mRNA	0.232916941	0.019481225
PLOD3	mRNA	0.230649189	0.018241285
FYCO1	mRNA	0.225130608	0.039753254
AIDA	mRNA	0.223741666	0.002498377
CDS2	mRNA	0.223653798	0.040202968
MIA3	mRNA	0.222522296	0.016741485
MOB3A	mRNA	0.221524843	0.049837238

PI4KA	mRNA	0.220097173	0.001984509
NDFIP1	mRNA	0.21668484	0.017616393
PTPN9	mRNA	0.216611796	0.04579236
SRP68	mRNA	0.216517724	0.029581
GAK	mRNA	0.214102951	0.036705654
NUB1	mRNA	0.214039916	0.04262732
ANKFY1	mRNA	0.209871381	0.048178219
FKBP1A	mRNA	0.209360131	0.02588482
CERS5	mRNA	0.207732003	0.015641556
TOB2	mRNA	0.20009055	0.022668399
BAHD1	mRNA	0.192819155	0.031149809
USO1	mRNA	0.192807212	0.020536259
HSF1	mRNA	0.192090677	0.032015113
RRAGA	mRNA	0.189535937	0.019440905
EGLN2	mRNA	0.179359564	0.035705778
TMBIM6	mRNA	0.166595406	0.04806737
LSM14A	mRNA	-0.185152492	0.032164417
TNRC6A	mRNA	-0.18865914	0.027894648
RBBP6	mRNA	-0.194824064	0.040969854
TAB2	mRNA	-0.19848636	0.012842252
USP22	mRNA	-0.199104331	0.037673905
SCD5	mRNA	-0.218071831	0.041441764
RABGAP1	mRNA	-0.221227021	0.041399953
BCL9	mRNA	-0.221751181	0.039095151
RBM22	mRNA	-0.222025917	0.031034108

MATR3	mRNA	-0.227034376	0.030489287
USP28	mRNA	-0.229074487	0.044056761
TRIM37	mRNA	-0.229815158	0.028319775
RBBP4	mRNA	-0.231649032	0.009125844
ARHGAP35	mRNA	-0.231689485	0.032864938
PPP6C	mRNA	-0.234898438	0.005135931
RBM4	mRNA	-0.235196209	0.030988271
KPNB1	mRNA	-0.235288649	0.015863461
ANKRD26	mRNA	-0.237014826	0.026407597
MAN2B1	mRNA	-0.237905844	0.03793949
SNX2	mRNA	-0.238649429	0.042726803
EIF5	mRNA	-0.240642939	0.010012053
METAP2	mRNA	-0.241654373	0.037246522
NAB1	mRNA	-0.242925762	0.03421771
RWDD2B	mRNA	-0.243079022	0.032390582
SPATA20	mRNA	-0.248132821	0.037948888
CAMK2D	mRNA	-0.248953399	0.001997371
LMTK2	mRNA	-0.249429145	0.005615439
NOC3L	mRNA	-0.249748938	0.049915677
EML4	mRNA	-0.255368234	0.007014026
ANKRD13A	mRNA	-0.256187796	0.046096343
PHLPP1	mRNA	-0.256808579	0.028396991
HKR1	mRNA	-0.258128278	0.03958397
TRAF7	mRNA	-0.258464834	0.010619546
STAG2	mRNA	-0.25920102	0.018524652

SMG6	mRNA	-0.259894914	0.012098677
NDUFB5	mRNA	-0.259936406	0.025849556
RANBP10	mRNA	-0.260157096	0.042927617
TRAPPC2L	mRNA	-0.260164927	0.036678157
DYRK1A	mRNA	-0.260694276	0.006372463
ZNF555	mRNA	-0.260731091	0.041189929
SLC25A3	mRNA	-0.262778184	0.040001746
ZNF45	mRNA	-0.262896742	0.012255854
PRUNE1	mRNA	-0.2652075	0.044080902
GTF2H1	mRNA	-0.266073931	0.010261011
DDX17	mRNA	-0.266511264	0.025715172
RAP2C	mRNA	-0.266987404	0.049014973
NAGLU	mRNA	-0.267450469	0.041072208
TNPO2	mRNA	-0.267873093	0.032320106
ARHGAP29	mRNA	-0.268619661	0.00803242
TTC21B	mRNA	-0.269529771	0.040196984
ZMYM2	mRNA	-0.269765425	0.038613005
GPR137B	mRNA	-0.272250192	0.03072471
SPG21	mRNA	-0.279759662	0.015930839
FAM53C	mRNA	-0.280290733	0.008190591
EIF3D	mRNA	-0.281220381	0.020074288
DEDD	mRNA	-0.281979607	0.003500434
ZZZ3	mRNA	-0.282751762	0.023801502
MAST3	mRNA	-0.284407034	0.046373055
RNF146	mRNA	-0.284921871	0.007524731

TIA1	mRNA	-0.28604664	0.011917715
CNOT7	mRNA	-0.286151649	0.006589525
AZI2	mRNA	-0.286296087	0.038737392
PCBP2	mRNA	-0.287261055	0.004830933
HIBCH	mRNA	-0.28784359	0.031353336
RARA	mRNA	-0.28795556	0.027168397
NSD3	mRNA	-0.288371593	6.50E-04
BTBD3	mRNA	-0.289717787	0.001790427
EDEM2	mRNA	-0.290609964	0.011231683
STK38L	mRNA	-0.291456483	0.016497553
TOPORS	mRNA	-0.292558795	0.006095514
RNF121	mRNA	-0.293850267	0.035096115
BRWD3	mRNA	-0.293994146	0.001655474
TEAD2	mRNA	-0.294790436	0.02519928
R3HCC1L	mRNA	-0.294843107	0.015250204
NEK7	mRNA	-0.29550777	0.014367972
REXO4	mRNA	-0.29620598	0.034468832
ZNF316	mRNA	-0.297257464	0.016924743
MED28	mRNA	-0.297353366	0.02811267
NT5DC1	mRNA	-0.299581116	0.002649805
ATG2A	mRNA	-0.299622762	0.024166305
FGD1	mRNA	-0.301109798	0.037062286
GBA	mRNA	-0.301755453	0.001330618
EGLN1	mRNA	-0.302068089	0.013068315
ZNF282	mRNA	-0.305725586	0.002748723

ADH5	mRNA	-0.306609164	0.00153345
MLYCD	mRNA	-0.30708577	0.015048362
LDLRAD3	mRNA	-0.307158592	0.038908155
CHD4	mRNA	-0.30774744	0.004526142
PCNX1	mRNA	-0.308582378	0.009027893
EIF3L	mRNA	-0.309315451	2.93E-04
GTF2IP1	pseudo	-0.309502695	4.14E-04
PEX19	mRNA	-0.310068291	6.48E-04
CCNC	mRNA	-0.310236223	0.036868119
FAM171B	mRNA	-0.31049502	0.021619357
COBLL1	mRNA	-0.31108381	0.005937419
CLCN7	mRNA	-0.313047083	0.017073995
ZFAND4	mRNA	-0.314983176	0.022742139
HSDL1	mRNA	-0.315624495	0.002300898
TEAD1	mRNA	-0.315841617	0.032872429
SPOP	mRNA	-0.316551336	0.029003431
UBL7	mRNA	-0.317396686	0.011881838
CCT4	mRNA	-0.317734158	0.010862061
DHX40	mRNA	-0.317997873	0.013098732
RPL6	mRNA	-0.318683878	0.003672694
hsa_circ_0026703	circRNA	-0.319002664	0.041052623
PHF20	mRNA	-0.320824747	1.95E-04
MRPS24	mRNA	-0.320921769	0.026922775
CHKA	mRNA	-0.321433965	0.032231143
ATF7IP2	mRNA	-0.321562953	0.029000407

ERLIN1	mRNA	-0.32222603	0.012685301
KCTD1	mRNA	-0.322273603	0.039236443
HEXB	mRNA	-0.323763035	3.49E-04
GLB1	mRNA	-0.326796847	6.49E-04
COPRS	mRNA	-0.327048066	0.023866442
CBX1	mRNA	-0.327133893	0.014936459
CDAN1	mRNA	-0.327270338	0.006218684
GRN	mRNA	-0.327775584	0.012798112
TNFAIP8L1	mRNA	-0.328794872	0.022854663
ZNF426	mRNA	-0.329061251	0.037673905
CSDE1	mRNA	-0.32919751	4.33E-04
CIAO2A	mRNA	-0.329811902	0.018867031
TAL1	mRNA	-0.330017268	0.01542439
PTCD2	mRNA	-0.330723677	0.012200182
ZNF776	mRNA	-0.331462434	0.006738646
SH3BP4	mRNA	-0.331730424	2.21E-04
hsa_circ_0063260	circRNA	-0.332688887	0.028170626
HEXA	mRNA	-0.333712379	0.003614366
hsa_circ_0063262	circRNA	-0.333769728	0.0365149
ZNF189	mRNA	-0.334624043	0.042680758
HIF1A	mRNA	-0.336367641	0.004838512
IPO5	mRNA	-0.336765935	0.032939267
EHMT2	mRNA	-0.337274568	0.014181042
PBX2	mRNA	-0.338578111	0.049443301
FBXO10	mRNA	-0.338642481	9.81E-04

HDAC7	mRNA	-0.339403533	0.001834247
PCCA	mRNA	-0.340135462	0.023802549
SUZ12P1	pseudo	-0.340934743	0.026462337
HADHB	mRNA	-0.341166505	0.016048101
SNHG14	lncRNA	-0.341700918	0.013362755
FOPNL	mRNA	-0.341873369	0.001623231
SNRNP48	mRNA	-0.342176209	0.005916587
ZDHHC2	mRNA	-0.343502957	0.001025128
NSD1	mRNA	-0.343755054	0.010594175
ATP6V1A	mRNA	-0.344318257	0.01189445
PAM	mRNA	-0.345219093	0.012255854
CDK14	mRNA	-0.345495635	0.048260462
ACAA2	mRNA	-0.346300847	0.006905957
PPP1CC	mRNA	-0.346529289	0.019873844
TMC6	mRNA	-0.347178124	0.014965972
PLEKHG2	mRNA	-0.347481501	0.013470061
hsa_circ_0074647	circRNA	-0.348277649	0.036212781
HOXD8	mRNA	-0.348426513	0.017101906
hsa_circ_0026704	circRNA	-0.348478805	0.036909429
OXA1L	mRNA	-0.349429733	1.88E-04
GALNS	mRNA	-0.349434907	0.021966864
TUT4	mRNA	-0.352253226	0.017386393
ZC4H2	mRNA	-0.352503664	0.011123963
hsa_circ_0035110	circRNA	-0.354162441	0.037962713
SLC25A13	mRNA	-0.354224678	3.77E-04

TPGS2	mRNA	-0.354304665	0.008235902
GPAM	mRNA	-0.356453699	0.006045713
SPARC	mRNA	-0.357172607	0.029807231
CTSD	mRNA	-0.357883637	0.016593706
ERCC2	mRNA	-0.357999066	0.043433754
hsa_circ_0008803	circRNA	-0.358235171	0.04885345
FOXO3	mRNA	-0.358530444	0.028850223
RRAS2	mRNA	-0.35875829	0.008281368
TMEM192	mRNA	-0.358797029	0.018617114
AIG1	mRNA	-0.359082045	0.031314645
FAM171A1	mRNA	-0.359959904	0.048178219
ST13	mRNA	-0.360161102	5.07E-05
GNS	mRNA	-0.360263893	0.014914982
NPEPPS	mRNA	-0.360321098	4.11E-04
SEPT11	mRNA	-0.360445861	0.015489828
ALKBH4	mRNA	-0.360521464	0.04579236
ZNF510	mRNA	-0.360854469	0.011815586
NT5C3A	mRNA	-0.363152113	0.034353224
TBC1D16	mRNA	-0.363187823	0.017992767
SLC27A1	mRNA	-0.363398244	0.044916415
RPL5	mRNA	-0.364044252	8.87E-04
UNKL	mRNA	-0.364341746	0.037948888
FLJ22447	lncRNA	-0.364852497	0.041295162
ADAT2	mRNA	-0.365520287	0.03156338
B3GLCT	mRNA	-0.367417425	2.81E-04

PCMTD2	mRNA	-0.367770972	0.029760754
ZNF292	mRNA	-0.368036898	0.002626065
B2M	mRNA	-0.368882485	0.02099464
hsa_circ_0003069	circRNA	-0.369261816	0.003649035
KCTD18	mRNA	-0.369549042	0.017425602
EHBP1	mRNA	-0.369850592	3.73E-04
MAML3	mRNA	-0.370238322	0.006377407
SPIN1	mRNA	-0.370824699	0.006646167
ABCB7	mRNA	-0.370929666	0.035860371
hsa_circ_0074652	circRNA	-0.371026473	0.011292767
PHF21A	mRNA	-0.374359053	0.026219166
ZKSCAN3	mRNA	-0.374525018	0.002190503
LY75	mRNA	-0.37597707	0.048923107
hsa_circ_0049591	circRNA	-0.375982519	0.024242716
ZNF232	mRNA	-0.375985326	0.030681184
CCDC88A	mRNA	-0.377240902	0.021026821
PRAG1	mRNA	-0.377330754	0.046096343
JCAD	mRNA	-0.377395313	0.002747159
SESTD1	mRNA	-0.378402089	4.51E-04
TMEM126B	mRNA	-0.378698862	0.017124721
hsa_circ_0074640	circRNA	-0.378914896	0.01852778
C20orf27	mRNA	-0.379118447	0.025227027
HOXA9	mRNA	-0.3794838	0.018209005
SCAI	mRNA	-0.379514234	0.044632407
UBA2	mRNA	-0.380196599	0.006120417

CCNJL	mRNA	-0.381650514	0.036657645
POLB	mRNA	-0.382474728	0.042012212
NIPSNAP2	mRNA	-0.383106546	0.002426576
CCDC120	mRNA	-0.383247836	0.044883063
ALPK1	mRNA	-0.383538346	0.013579096
hsa_circ_0052486	circRNA	-0.383940713	0.014340248
ZNF606	mRNA	-0.385179556	0.029797499
TGIF2	mRNA	-0.385382562	1.29E-05
GCDH	mRNA	-0.387391191	0.012739825
hsa_circ_0020457	circRNA	-0.387442693	0.045484457
ZNHIT3	mRNA	-0.387719433	0.037302993
LOC101927060	lncRNA	-0.387831053	0.02121114
UIMC1	mRNA	-0.388024308	0.004384808
RPUSD4	mRNA	-0.388329575	0.028669786
LAMA4	mRNA	-0.389747648	0.020440692
hsa_circ_0103154	circRNA	-0.389821471	0.035813455
TRIM68	mRNA	-0.390230526	0.027458008
PCGF5	mRNA	-0.390527947	0.045254572
hsa_circ_0129595	circRNA	-0.390720382	0.045715934
BCKDHB	mRNA	-0.391020089	7.06E-04
HMGN1	mRNA	-0.391266138	9.79E-04
hsa_circ_0063463	circRNA	-0.391299611	0.005952542
hsa_circ_0074648	circRNA	-0.391934574	0.003242139
ZNF211	mRNA	-0.393898589	0.025917754
FN3K	mRNA	-0.394480515	0.003862935

KANSL1L	mRNA	-0.394881098	0.042525181
PAWR	mRNA	-0.395238767	0.002495688
KATNA1	mRNA	-0.395273806	0.026506454
USP49	mRNA	-0.395770741	0.010462387
LANCL1	mRNA	-0.395851252	3.76E-04
TTBK2	mRNA	-0.39596035	0.019563504
MPND	mRNA	-0.396667764	0.024729252
ZNF615	mRNA	-0.39675469	0.020114775
WDR19	mRNA	-0.397254657	0.020730735
TRIM62	mRNA	-0.397263433	0.002279792
OTUD1	mRNA	-0.39756167	0.042478545
ZNF813	mRNA	-0.398834591	0.038109026
hsa_circ_0050515	circRNA	-0.399946628	0.037767485
BORCS5	mRNA	-0.400260427	5.59E-04
LPCAT3	mRNA	-0.401077953	0.006075129
hsa_circ_0050521	circRNA	-0.401130223	0.035813455
hsa_circ_0021292	circRNA	-0.401431126	0.00698213
ZNF532	mRNA	-0.401652816	0.002082806
hsa_circ_0084044	circRNA	-0.402653239	0.043071089
POLD4	mRNA	-0.403457416	0.012067086
SOCS5	mRNA	-0.40407288	0.009094014
hsa_circ_0026646	circRNA	-0.404849849	0.005701336
hsa_circ_0004293	circRNA	-0.405090204	0.027619437
CLCN5	mRNA	-0.40533332	0.011343834
STXBP2	mRNA	-0.405378355	0.012515134

FBXL20	mRNA	-0.40629485	0.014070756
CEP57	mRNA	-0.406879046	0.016492407
hsa_circ_0074643	circRNA	-0.408122511	0.038673635
LOC101930370	lncRNA	-0.408125951	0.049259008
LINC01578	lncRNA	-0.408170873	0.005314479
DYRK1B	mRNA	-0.408956695	8.83E-04
NPM1	mRNA	-0.409089014	0.002664302
XXYLT1	mRNA	-0.409105624	0.013086758
POLR2M	mRNA	-0.409497481	9.31E-07
STK17B	mRNA	-0.409904515	0.00168839
hsa_circ_0043385	circRNA	-0.410942584	0.043791765
WSB2	mRNA	-0.411549474	0.001495811
CYP2U1	mRNA	-0.411642942	0.04035601
HIP1R	mRNA	-0.411670048	0.016003793
RPS6KB2	mRNA	-0.41216453	0.002310647
EPB41L4A-AS1	lncRNA	-0.412875396	5.77E-04
HHAT	mRNA	-0.413132096	0.03058459
hsa_circ_0017874	circRNA	-0.413138693	0.016095614
hsa_circ_0075009	circRNA	-0.413307046	0.024680613
LINC01278	lncRNA	-0.413482684	6.06E-04
SPOPL	mRNA	-0.413667814	6.17E-04
ZFP3	mRNA	-0.413761054	0.049443301
DZIP1	mRNA	-0.413762456	1.59E-06
EVC	mRNA	-0.413921437	6.11E-05
hsa_circ_0026654	circRNA	-0.414506203	0.007026585

GTDC1	mRNA	-0.414720337	1.54E-04
hsa_circ_0075012	circRNA	-0.414945768	0.00435372
hsa_circ_0026652	circRNA	-0.41538339	0.006073807
SNX5	mRNA	-0.415935058	0.001611373
hsa_circ_0043675	circRNA	-0.416988873	0.041904534
NFATC2	mRNA	-0.417000485	0.005617282
MYO18A	mRNA	-0.417372353	0.003753318
hsa_circ_0044315	circRNA	-0.417381967	0.026485773
MYL6B	mRNA	-0.417492246	0.004309894
hsa_circ_0106997	circRNA	-0.419543334	0.015693019
hsa_circ_0044063	circRNA	-0.419865199	0.03673965
hsa_circ_0026645	circRNA	-0.420414198	0.003182206
ZNF518A	mRNA	-0.420613823	0.00339835
BCKDHA	mRNA	-0.421104701	0.009837373
hsa_circ_0008092	circRNA	-0.421493854	0.009718302
hsa_circ_0063171	circRNA	-0.421971339	0.007192557
TUBG2	mRNA	-0.422586626	0.018285779
hsa_circ_0023079	circRNA	-0.423299849	0.03342834
SUOX	mRNA	-0.423892622	0.002530145
hsa_circ_0060894	circRNA	-0.424400889	0.04370161
SLC25A38	mRNA	-0.424730203	0.001616135
hsa_circ_0047659	circRNA	-0.424735703	0.035941274
TIFA	mRNA	-0.425086642	0.030088849
TPP1	mRNA	-0.42514209	0.001520972
hsa_circ_0074656	circRNA	-0.425721929	0.014374262

DBT	mRNA	-0.425945345	4.59E-04
hsa_circ_0063268	circRNA	-0.426481866	4.78E-04
CTPS2	mRNA	-0.426651976	8.25E-04
PGPEP1	mRNA	-0.427289996	1.14E-04
hsa_circ_0044265	circRNA	-0.429309609	0.022652738
ARL5A	mRNA	-0.430981429	0.001936337
UPF3B	mRNA	-0.431128064	0.020713407
MRPL22	mRNA	-0.431265708	0.028456508
RFTN2	mRNA	-0.431502901	0.008834725
ING3	mRNA	-0.431786303	0.003224216
hsa_circ_0078558	circRNA	-0.432265475	0.042063118
ZBTB44	mRNA	-0.432266365	0.006905957
hsa_circ_0079970	circRNA	-0.432671674	0.009069308
hsa_circ_0064717	circRNA	-0.432854127	0.029524743
hsa_circ_0107564	circRNA	-0.433957606	0.024266099
FBXL5	mRNA	-0.434382234	1.51E-04
ZSCAN2	mRNA	-0.434736672	0.022810739
C1RL	mRNA	-0.434757584	0.01119989
hsa_circ_0054401	circRNA	-0.43488719	0.045500196
RFK	mRNA	-0.435332818	0.016998262
EFEMP2	mRNA	-0.436074763	0.003045897
hsa_circ_0026883	circRNA	-0.43621232	0.035941274
hsa_circ_0137631	circRNA	-0.436437269	0.021998896
RBBP9	mRNA	-0.436717077	1.72E-05
GALT	mRNA	-0.437208441	0.016782884

TMEM131L	mRNA	-0.437466592	0.001127167
hsa_circ_0064716	circRNA	-0.437655731	0.013266499
hsa_circ_0043382	circRNA	-0.437940995	0.029811942
hsa_circ_0028229	circRNA	-0.437943467	0.016469331
ZFPM2	mRNA	-0.438467955	0.029552701
hsa_circ_0021998	circRNA	-0.438609276	0.047697841
hsa_circ_0087831	circRNA	-0.4390762	0.0192961
hsa_circ_0003595	circRNA	-0.439127267	0.042229752
EPHB2	mRNA	-0.439656062	0.027243736
hsa_circ_0017835	circRNA	-0.439740692	0.043011788
STRBP	mRNA	-0.440907453	1.72E-05
BHLHB9	mRNA	-0.441103619	0.036947347
PPP3CA	mRNA	-0.441646057	0.009421278
hsa_circ_0075004	circRNA	-0.44204566	0.017590664
hsa_circ_0037483	circRNA	-0.443593535	0.03722613
VIM	mRNA	-0.44367763	0.019972214
HDAC10	mRNA	-0.443785626	0.028716568
PHTF2	mRNA	-0.443801481	0.02180423
SIPA1L1	mRNA	-0.444063892	8.89E-05
hsa_circ_0036431	circRNA	-0.444083794	0.041496477
hsa_circ_0003443	circRNA	-0.444237303	0.042063118
LOC101927556	lncRNA	-0.444648027	4.11E-04
hsa_circ_0109653	circRNA	-0.445108461	0.00435372
GAMT	mRNA	-0.445419608	0.018891832
hsa_circ_0002358	circRNA	-0.446270955	0.009418207

FZD1	mRNA	-0.446778586	0.046325724
hsa_circ_0057516	circRNA	-0.446802917	0.037855531
ZNF662	mRNA	-0.446906775	0.009391378
hsa_circ_0029150	circRNA	-0.447867692	0.032745575
hsa_circ_0057521	circRNA	-0.448042337	0.032865795
TAF9B	mRNA	-0.448651909	0.001419054
CHD3	mRNA	-0.449172455	0.015969054
SNX30	mRNA	-0.449274324	0.002281719
SLC25A29	mRNA	-0.449413678	0.001537123
hsa_circ_0137621	circRNA	-0.449808297	0.036751974
hsa_circ_0012213	circRNA	-0.449883914	0.020807852
APH1B	mRNA	-0.450035825	0.002056831
FAM214A	mRNA	-0.450670631	0.00494826
ZNF655	mRNA	-0.450979379	0.024889421
hsa_circ_0017879	circRNA	-0.451836594	0.006825012
MINDY4	mRNA	-0.451971996	0.004309083
AGPAT5	mRNA	-0.452465872	1.02E-04
hsa_circ_0057517	circRNA	-0.452749747	0.025472333
hsa_circ_0075001	circRNA	-0.453604325	0.030475574
hsa_circ_0075019	circRNA	-0.454111343	0.036613236
MLLT1	mRNA	-0.454237935	0.001564431
NCEH1	mRNA	-0.454550665	0.006970381
hsa_circ_0074995	circRNA	-0.454627003	0.010793689
hsa_circ_0024164	circRNA	-0.454628013	0.027969199
BMT2	mRNA	-0.454801112	0.005798206

GIMAP2	mRNA	-0.454860468	0.007182296
hsa_circ_0064863	circRNA	-0.455335571	0.04917849
SCMH1	mRNA	-0.455592829	6.33E-05
hsa_circ_0006662	circRNA	-0.456576668	0.032763955
hsa_circ_0061227	circRNA	-0.456773365	0.016000708
hsa_circ_0026649	circRNA	-0.457389781	0.001345251
hsa_circ_0093230	circRNA	-0.457826417	0.039723101
CDIP1	mRNA	-0.458403776	0.013103791
hsa_circ_0011885	circRNA	-0.458703278	0.029375906
hsa_circ_0112922	circRNA	-0.459500262	0.030221927
hsa_circ_0001007	circRNA	-0.459519686	0.042824532
hsa_circ_0112281	circRNA	-0.459822821	0.044581155
DCAF11	mRNA	-0.460073137	0.005473733
hsa_circ_0050526	circRNA	-0.460467663	0.018295239
MOSPD3	mRNA	-0.46083351	0.004879848
BOK	mRNA	-0.461339982	6.27E-04
RBMS3	mRNA	-0.461578149	0.039421244
TGFBRAP1	mRNA	-0.461768488	4.49E-04
hsa_circ_0059499	circRNA	-0.462067263	0.014018431
CEP162	mRNA	-0.462808814	0.008204337
ZMYM3	mRNA	-0.463864889	0.031477313
AGPAT2	mRNA	-0.464865524	0.010999706
hsa_circ_0007007	circRNA	-0.46601782	0.04257512
RCBTB2	mRNA	-0.466073962	0.006879904
SMURF2	mRNA	-0.467711585	4.93E-07

METRNL	mRNA	-0.468019687	0.027190117
hsa_circ_0115451	circRNA	-0.46804783	0.034350047
LINC00938	lncRNA	-0.468600311	0.006924793
PHC1	mRNA	-0.468999137	3.90E-04
ZNF620	mRNA	-0.469083258	0.045219245
BLOC1S5	mRNA	-0.469127256	0.004540122
hsa_circ_0085321	circRNA	-0.47025363	0.043071089
hsa_circ_0062521	circRNA	-0.470865853	0.0351108
hsa_circ_0110585	circRNA	-0.471101425	0.045114256
TAF6L	mRNA	-0.471245188	8.29E-05
hsa_circ_0013185	circRNA	-0.471308129	0.004506025
hsa_circ_0026908	circRNA	-0.472606479	0.018911328
PUS10	mRNA	-0.473269927	0.007021639
hsa_circ_0012210	circRNA	-0.473933379	0.010124157
hsa_circ_0017880	circRNA	-0.47403321	0.005331392
OAZ1	mRNA	-0.474261184	6.80E-04
hsa_circ_0045321	circRNA	-0.474285033	0.014034242
hsa_circ_0097671	circRNA	-0.475500599	0.037950933
STMN1	mRNA	-0.475542899	0.047671236
PINK1	mRNA	-0.47594605	0.027246259
ODC1	mRNA	-0.476191821	0.041644429
hsa_circ_0128537	circRNA	-0.477398862	0.01493257
IGF2R	mRNA	-0.477800443	0.022661755
DNMT3B	mRNA	-0.477860547	0.014915275
RNF8	mRNA	-0.477979124	0.00975883

hsa_circ_0075005	circRNA	-0.478524226	0.002726843
OXCT1	mRNA	-0.478654172	0.017393069
HTRA1	mRNA	-0.478841534	2.09E-05
ATP7A	mRNA	-0.479561392	0.018550769
EXTL2	mRNA	-0.479984355	4.16E-04
hsa_circ_0106585	circRNA	-0.480305575	0.030891206
RNF144A	mRNA	-0.480347998	0.014866109
PFKM	mRNA	-0.48094719	5.16E-08
EEF1AKMT1	mRNA	-0.481016544	0.00601365
hsa_circ_0026015	circRNA	-0.4813256	0.013274003
NID1	mRNA	-0.481643053	2.96E-04
ZNF91	mRNA	-0.481861628	1.03E-04
RAB4A	mRNA	-0.482103606	0.005937419
LYRM1	mRNA	-0.482256985	0.049963759
hsa_circ_0124869	circRNA	-0.482449068	0.033420468
FAM13A	mRNA	-0.482560998	0.005203441
MYO10	mRNA	-0.482684329	0.028155281
hsa_circ_0005671	circRNA	-0.482768376	0.036578677
NDRG3	mRNA	-0.48326994	0.002275436
SLC16A1	mRNA	-0.48336277	0.003854266
TSPYL4	mRNA	-0.483907847	7.68E-04
hsa_circ_0008081	circRNA	-0.484002717	0.001468434
CMTR2	mRNA	-0.484375741	2.29E-04
hsa_circ_0048178	circRNA	-0.484402933	0.004879908
hsa_circ_0050505	circRNA	-0.484545423	0.022588216

ABCA5	mRNA	-0.484927522	0.010443089
SNAPC1	mRNA	-0.485350446	0.009843902
LRRC37A2	mRNA	-0.485633359	0.006149601
MAP10	mRNA	-0.485978019	0.030305896
hsa_circ_0006987	circRNA	-0.486495099	0.019781362
hsa_circ_0030680	circRNA	-0.487144563	0.038526779
hsa_circ_0006385	circRNA	-0.488329758	0.022694254
GNPDA1	mRNA	-0.488438642	0.022790267
SPATA6	mRNA	-0.488672206	0.012645233
CACHD1	mRNA	-0.488810512	0.001240157
GAB1	mRNA	-0.488817732	0.047700694
hsa_circ_0027608	circRNA	-0.489073282	0.040525599
CSNK2A2	mRNA	-0.489332574	2.02E-04
hsa_circ_0075000	circRNA	-0.489475228	0.00337781
RIMKLB	mRNA	-0.489729328	7.77E-06
FLCN	mRNA	-0.489845353	0.032786776
hsa_circ_0107015	circRNA	-0.490486369	0.039740662
hsa_circ_0007050	circRNA	-0.491285517	0.047984701
hsa_circ_0004457	circRNA	-0.49169706	0.015351231
hsa_circ_0007591	circRNA	-0.49169706	0.015351231
KCNC3	mRNA	-0.492097992	0.041493626
MET	mRNA	-0.49211623	0.023729652
CYB5RL	mRNA	-0.492134177	6.11E-04
hsa_circ_0007434	circRNA	-0.492875358	0.015769883
FRAT1	mRNA	-0.493236626	0.024634985

HNMT	mRNA	-0.493296081	0.045651361
hsa_circ_0008212	circRNA	-0.493300868	0.036126175
LCORL	mRNA	-0.494625088	9.14E-05
hsa_circ_0087827	circRNA	-0.495613665	0.030505626
DTX3	mRNA	-0.495784409	6.63E-04
BCAS3	mRNA	-0.495793161	0.002133917
hsa_circ_0008820	circRNA	-0.496437967	0.02290569
hsa_circ_0031219	circRNA	-0.496784375	0.011195558
AHCYL2	mRNA	-0.497520172	0.011556316
ZNF346	mRNA	-0.497616971	1.32E-06
hsa_circ_0017095	circRNA	-0.498468199	0.001446633
MGLL	mRNA	-0.499734258	0.023338593
hsa_circ_0056386	circRNA	-0.499955559	0.044983106
hsa_circ_0119636	circRNA	-0.499993298	0.001623258
FAM124B	mRNA	-0.500153251	0.030615988
CACFD1	mRNA	-0.500557501	0.011097625
hsa_circ_0108334	circRNA	-0.500565734	0.018586409
hsa_circ_0109471	circRNA	-0.50101072	0.00375756
TFB1M	mRNA	-0.501033042	0.00357303
hsa_circ_0070502	circRNA	-0.501207402	0.032655151
hsa_circ_0015073	circRNA	-0.501365654	0.027577228
DIS3L	mRNA	-0.501397813	3.82E-06
hsa_circ_0106833	circRNA	-0.501679329	0.01971967
ZEB2	mRNA	-0.501840535	0.006128309
hsa_circ_0081813	circRNA	-0.50184512	0.023314861

ZNF43	mRNA	-0.502343551	3.11E-04
hsa_circ_0078651	circRNA	-0.50259449	0.007439092
LOC102723694	lncRNA	-0.503088974	0.031477313
LRP6	mRNA	-0.504016595	0.04579236
PABPC4L	mRNA	-0.504283893	0.04225039
hsa_circ_0048640	circRNA	-0.505227436	0.0365149
hsa_circ_0005341	circRNA	-0.506208818	0.014078056
SMO	mRNA	-0.506243896	0.043558066
hsa_circ_0050549	circRNA	-0.506644767	0.017941679
hsa_circ_0048177	circRNA	-0.506774997	0.005947637
hsa_circ_0027609	circRNA	-0.508375581	0.037941183
TUSC3	mRNA	-0.508582294	0.037127416
hsa_circ_0050947	circRNA	-0.508603852	0.034098996
hsa_circ_0058096	circRNA	-0.508745535	0.030414009
hsa_circ_0107465	circRNA	-0.510957894	0.009265506
SUMF1	mRNA	-0.511169025	0.008749227
hsa_circ_0100010	circRNA	-0.511698724	0.002787662
REL	mRNA	-0.511886277	0.041275395
MOCS1	mRNA	-0.512159585	7.60E-04
hsa_circ_0117035	circRNA	-0.51220155	0.019717117
hsa_circ_0029151	circRNA	-0.512306216	0.005570055
RHNO1	mRNA	-0.51232161	0.036444041
COL4A5	mRNA	-0.512631063	0.024275479
ZBED3	mRNA	-0.513186252	8.31E-06
hsa_circ_0118330	circRNA	-0.513224327	0.023887613

hsa_circ_0017099	circRNA	-0.513893986	0.007396553
FAM234B	mRNA	-0.514462366	1.83E-07
hsa_circ_0051287	circRNA	-0.514935067	0.010793689
hsa_circ_0136892	circRNA	-0.515007047	0.010008066
hsa_circ_0052601	circRNA	-0.515084116	0.021441401
hsa_circ_0007681	circRNA	-0.515253054	0.041794921
ELK4	mRNA	-0.515363759	0.001387357
hsa_circ_0078652	circRNA	-0.515434257	0.007811709
RHOD	mRNA	-0.515569173	0.008716012
NINL	mRNA	-0.515980878	0.001883751
USP30	mRNA	-0.516151144	0.002311047
hsa_circ_0007981	circRNA	-0.51629749	0.039821776
GM2A	mRNA	-0.516364148	2.88E-07
hsa_circ_0017878	circRNA	-0.517264301	0.00435372
MPP1	mRNA	-0.517941302	0.006917389
PLEKHA4	mRNA	-0.518110267	0.021966864
PTK7	mRNA	-0.518469031	0.049462024
hsa_circ_0122956	circRNA	-0.518737761	0.01971967
hsa_circ_0077671	circRNA	-0.518745586	0.002414008
PPT1	mRNA	-0.519071754	0.014754949
hsa_circ_0017094	circRNA	-0.519162552	0.033420468
ING4	mRNA	-0.519293281	7.83E-06
MTA3	mRNA	-0.519348819	1.67E-04
ARVCF	mRNA	-0.520213789	0.011817454
GGT7	mRNA	-0.520269691	0.042107204

hsa_circ_0077073	circRNA	-0.521095472	0.017556376
ZNF792	mRNA	-0.522128032	0.020385008
PRPSAP1	mRNA	-0.522631171	1.75E-05
RAD51B	mRNA	-0.522726074	0.001269474
CCDC171	mRNA	-0.523430472	0.037481662
hsa_circ_0004594	circRNA	-0.523458234	0.003568445
BGIG9606_43971	lncRNA	-0.523716127	0.034147885
YPEL5	mRNA	-0.524153943	0.019063232
LMO4	mRNA	-0.524545622	0.005483235
hsa_circ_0112916	circRNA	-0.524577379	0.041610007
DNMT3A	mRNA	-0.524675416	1.85E-07
hsa_circ_0011886	circRNA	-0.525272829	0.025081735
hsa_circ_0050522	circRNA	-0.525418005	0.004138322
hsa_circ_0118410	circRNA	-0.525666945	0.038364388
OSBPL1A	mRNA	-0.525930182	0.001067024
hsa_circ_0036214	circRNA	-0.526285806	6.17E-04
MCU	mRNA	-0.52675219	9.05E-06
ZNF30	mRNA	-0.527170698	0.001797983
hsa_circ_0134451	circRNA	-0.527398333	0.014554396
ARID5B	mRNA	-0.527971128	0.022636934
EPB41	mRNA	-0.528348256	2.22E-05
hsa_circ_0129206	circRNA	-0.528886894	0.041630541
hsa_circ_0037588	circRNA	-0.528942021	0.020201677
HSPBAP1	mRNA	-0.529228155	0.033981929
TJP2	mRNA	-0.529262103	0.022387019

hsa_circ_0110550	circRNA	-0.529426783	0.006791831
hsa_circ_0009686	circRNA	-0.530035485	0.022216785
LYRM9	mRNA	-0.530794191	0.006377407
hsa_circ_0094111	circRNA	-0.531012178	0.02723206
ZNF667	mRNA	-0.531707076	0.028146249
hsa_circ_0127586	circRNA	-0.532480243	0.023841572
hsa_circ_0016150	circRNA	-0.532529012	0.011801801
HIVEP1	mRNA	-0.532919413	0.00127287
hsa_circ_0045479	circRNA	-0.533673493	0.01400084
AMZ2P1	pseudo	-0.535886263	0.033623355
hsa_circ_0072013	circRNA	-0.536658593	0.005187928
hsa_circ_0066913	circRNA	-0.536800929	0.003007321
PPP2R3A	mRNA	-0.536994273	0.037236186
RCN2	mRNA	-0.537010608	0.028494879
hsa_circ_0016859	circRNA	-0.537389804	0.00790792
GMDS	mRNA	-0.53874926	0.003372833
hsa_circ_0020339	circRNA	-0.53927338	0.045821251
C7orf31	mRNA	-0.539705322	0.032786776
hsa_circ_0110015	circRNA	-0.539862511	0.00460902
hsa_circ_0123721	circRNA	-0.54008365	0.01975962
MMP16	mRNA	-0.540791988	8.94E-04
HOXA11	mRNA	-0.540993719	0.041266908
hsa_circ_0060205	circRNA	-0.541053582	0.029614262
TSPAN6	mRNA	-0.541098044	2.01E-04
hsa_circ_0083964	circRNA	-0.541190668	0.011801801

hsa_circ_0008252	circRNA	-0.541322806	0.047697841
RASSF4	mRNA	-0.541981171	0.015435911
hsa_circ_0017875	circRNA	-0.542309944	0.001491688
hsa_circ_0059508	circRNA	-0.54266257	0.002608424
hsa_circ_0016851	circRNA	-0.542749925	0.034843649
ABI2	mRNA	-0.543080637	4.04E-07
KANTR	lncRNA	-0.545141093	0.029552262
FSTL1	mRNA	-0.545453384	6.07E-05
CLN8	mRNA	-0.545501506	0.012093681
hsa_circ_0107031	circRNA	-0.545861751	0.014178345
hsa_circ_0066909	circRNA	-0.545964804	0.003438284
MAPRE2	mRNA	-0.546257057	9.87E-04
SPATA5	mRNA	-0.546358771	0.01895796
PIGK	mRNA	-0.546422263	0.00499266
CPPED1	mRNA	-0.547586053	9.60E-06
hsa_circ_0010697	circRNA	-0.547673622	0.035022915
hsa_circ_0064713	circRNA	-0.547800551	8.15E-04
PEX2	mRNA	-0.549428605	0.04225039
TMEM135	mRNA	-0.549736498	1.40E-04
hsa_circ_0109714	circRNA	-0.549779267	0.037448212
hsa_circ_0017865	circRNA	-0.550006504	0.003863288
hsa_circ_0116929	circRNA	-0.550022078	0.029460688
NFATC1	mRNA	-0.550133767	3.65E-04
hsa_circ_0125711	circRNA	-0.550438157	0.0365149
hsa_circ_0064037	circRNA	-0.550488632	0.015960934

UBE4B	mRNA	-0.550498289	2.27E-05
THAP8	mRNA	-0.550843304	0.00189418
hsa_circ_0066711	circRNA	-0.551029706	0.023751112
hsa_circ_0114368	circRNA	-0.551220879	0.031580566
KANK1	mRNA	-0.551359348	0.049837238
MAP2K5	mRNA	-0.551453427	0.011710787
hsa_circ_0051028	circRNA	-0.551479231	0.005480693
hsa_circ_0075018	circRNA	-0.551707777	0.032931036
C18orf54	mRNA	-0.552181424	0.014603391
hsa_circ_0075021	circRNA	-0.552284579	0.01644576
hsa_circ_0043673	circRNA	-0.553023405	0.016999395
ZNF462	mRNA	-0.553215775	3.52E-04
LOC107985544	lncRNA	-0.553378017	0.031477313
RNF41	mRNA	-0.554126417	0.026102589
hsa_circ_0110583	circRNA	-0.554457002	0.002995036
RBM43	mRNA	-0.554500275	0.011169518
SH3D19	mRNA	-0.554651985	0.011746545
hsa_circ_0015876	circRNA	-0.555620424	0.001478642
THRA	mRNA	-0.555732472	0.005483235
hsa_circ_0002720	circRNA	-0.555928641	0.011191609
LHFPL6	mRNA	-0.556520176	0.015880135
ACP2	mRNA	-0.557123313	1.60E-04
CCDC85C	mRNA	-0.557144385	0.004921482
hsa_circ_0002862	circRNA	-0.557423884	0.010585962
hsa_circ_0018762	circRNA	-0.557562996	0.013681834

hsa_circ_0010388	circRNA	-0.557627721	0.022950097
hsa_circ_0075685	circRNA	-0.558366843	0.006746665
CPQ	mRNA	-0.558524613	0.001091443
ZNF844	mRNA	-0.559089319	0.029859852
hsa_circ_0058150	circRNA	-0.559365826	0.016733263
hsa_circ_0075154	circRNA	-0.559423046	0.040300666
hsa_circ_0075590	circRNA	-0.559514881	0.030306714
hsa_circ_0123838	circRNA	-0.560602385	0.018823188
hsa_circ_0001260	circRNA	-0.561984823	0.011198979
GPRASP2	mRNA	-0.562548921	0.0075817
MICAL2	mRNA	-0.563150237	0.002554937
CERS6	mRNA	-0.56347014	8.60E-06
WASF3	mRNA	-0.563949693	0.048846429
hsa_circ_0072003	circRNA	-0.564300944	0.008545687
hsa_circ_0083670	circRNA	-0.564538486	0.001475407
hsa_circ_0010703	circRNA	-0.564705721	0.03912351
hsa_circ_0010701	circRNA	-0.56485523	0.024807331
FSD1	mRNA	-0.56494749	0.03693834
LDB1	mRNA	-0.564952074	2.40E-05
ZNF711	mRNA	-0.565168478	1.87E-04
hsa_circ_0112915	circRNA	-0.565434506	0.029973341
FAM78A	mRNA	-0.565668786	0.045567796
PBLD	mRNA	-0.565735212	1.75E-04
MEF2C-AS1	lncRNA	-0.565849941	0.03383259
GRIK5	mRNA	-0.565971205	0.006148937

hsa_circ_0088410	circRNA	-0.566929004	0.013059655
hsa_circ_0017111	circRNA	-0.567106066	1.37E-04
RHOJ	mRNA	-0.567108425	3.05E-04
hsa_circ_0048823	circRNA	-0.567315626	0.01853926
hsa_circ_0026493	circRNA	-0.568684392	0.029001703
CEP68	mRNA	-0.569017719	0.00396687
RBMX	mRNA	-0.569398258	8.50E-04
ARSB	mRNA	-0.569470349	0.021786055
hsa_circ_0097493	circRNA	-0.569715828	0.004825977
ZNF93	mRNA	-0.570046271	0.031297766
PDK3	mRNA	-0.570126176	0.024164533
KAT2B	mRNA	-0.570326438	5.07E-05
hsa_circ_0004940	circRNA	-0.570327268	0.023875906
LYL1	mRNA	-0.571031013	0.024853498
hsa_circ_0012248	circRNA	-0.571146488	0.015442499
CDKN1B	mRNA	-0.571463766	2.61E-04
hsa_circ_0023092	circRNA	-0.571661292	0.030303502
hsa_circ_0026909	circRNA	-0.572985039	0.04120107
MPP7	mRNA	-0.573210428	0.034778817
hsa_circ_0027436	circRNA	-0.573233851	0.023833786
hsa_circ_0017867	circRNA	-0.573382439	0.002714749
TMEM268	mRNA	-0.575220129	0.016324256
RMND1	mRNA	-0.575269239	0.004763129
hsa_circ_0130783	circRNA	-0.576466006	0.024517296
hsa_circ_0009052	circRNA	-0.576544063	0.035627066

ZNF827	mRNA	-0.576653909	6.80E-06
hsa_circ_0017882	circRNA	-0.577977614	3.21E-04
hsa_circ_0010607	circRNA	-0.578959995	0.020341011
hsa_circ_0032150	circRNA	-0.579286312	0.003122198
hsa_circ_0066912	circRNA	-0.579974826	3.48E-04
MBTD1	mRNA	-0.580119151	4.77E-04
hsa_circ_0010095	circRNA	-0.581183539	0.02735311
GMPR	mRNA	-0.581190038	6.39E-06
VEGFB	mRNA	-0.581206337	1.44E-05
MERTK	mRNA	-0.581338995	2.32E-06
hsa_circ_0071992	circRNA	-0.581473078	3.47E-04
hsa_circ_0025472	circRNA	-0.58229605	0.01855845
ABL2	mRNA	-0.582756528	4.85E-07
APLP1	mRNA	-0.582895456	0.037153818
S1PR3	mRNA	-0.58314903	0.043050154
hsa_circ_0022399	circRNA	-0.583168428	0.02290569
OSER1-DT	lncRNA	-0.583445877	0.002296118
YPEL3	mRNA	-0.583662468	0.003437048
hsa_circ_0108327	circRNA	-0.585060154	0.004977128
hsa_circ_0041978	circRNA	-0.585983379	0.001051865
hsa_circ_0061009	circRNA	-0.58618253	0.001437663
HHIP-AS1	lncRNA	-0.586399895	0.029279473
hsa_circ_0028938	circRNA	-0.587124926	0.018302621
hsa_circ_0003582	circRNA	-0.587375264	0.023183805
BGIG9606_56119	lncRNA	-0.588244145	0.036812772

hsa_circ_0026912	circRNA	-0.588514045	0.043752409
hsa_circ_0050556	circRNA	-0.58854322	0.004596715
hsa_circ_0066311	circRNA	-0.589108216	0.004558527
PTGFRN	mRNA	-0.589728018	7.47E-04
hsa_circ_0111798	circRNA	-0.59117651	0.014143391
hsa_circ_0027428	circRNA	-0.591627099	0.019982278
SEMA6C	mRNA	-0.591801754	0.028228645
hsa_circ_0044814	circRNA	-0.592367135	0.009677673
hsa_circ_0064030	circRNA	-0.592422587	0.007487573
hsa_circ_0006620	circRNA	-0.592855728	0.028796401
HIBADH	mRNA	-0.593099937	8.72E-06
hsa_circ_0051500	circRNA	-0.593384234	0.015581991
hsa_circ_0109473	circRNA	-0.593919639	0.016302139
LOXL2	mRNA	-0.594695447	1.86E-07
hsa_circ_0000369	circRNA	-0.595124707	0.009918998
hsa_circ_0020946	circRNA	-0.595468411	0.020050771
TRIM45	mRNA	-0.595623413	0.003481474
hsa_circ_0043670	circRNA	-0.596203592	0.027373375
TMCC3	mRNA	-0.59627699	0.01598238
hsa_circ_0030858	circRNA	-0.596872828	2.18E-04
hsa_circ_0047822	circRNA	-0.597229739	0.023259741
hsa_circ_0055982	circRNA	-0.598116132	0.026881972
ZNF521	mRNA	-0.59813638	0.012255854
MDK	mRNA	-0.598769859	0.014894327
hsa_circ_0107678	circRNA	-0.598896341	0.020650732

GTF2IRD2	mRNA	-0.598904369	0.016357326
hsa_circ_0064714	circRNA	-0.599682865	0.003676085
LOC389765	pseudo	-0.599828117	0.00302562
hsa_circ_0017884	circRNA	-0.600100389	6.00E-04
hsa_circ_0061010	circRNA	-0.600509321	0.006621776
hsa_circ_0004124	circRNA	-0.600673588	0.0351108
hsa_circ_0010677	circRNA	-0.600677787	0.042181654
MBNL3	mRNA	-0.600861576	7.28E-06
MXI1	mRNA	-0.6010044	0.005820699
PYCARD	mRNA	-0.60115828	0.030754608
ACADS	mRNA	-0.60119979	4.32E-05
SYNGR1	mRNA	-0.602798466	4.27E-04
hsa_circ_0030844	circRNA	-0.60296528	0.013641453
hsa_circ_0066365	circRNA	-0.603229926	0.037254018
ANKRD50	mRNA	-0.603319319	5.59E-04
USP51	mRNA	-0.603609576	0.008169955
hsa_circ_0076292	circRNA	-0.604037929	0.045326933
SEMA4D	mRNA	-0.604461716	0.001565876
ZNF821	mRNA	-0.60451318	2.74E-05
hsa_circ_0030850	circRNA	-0.604842886	0.007878116
hsa_circ_0112921	circRNA	-0.605005232	0.003603997
PBX1	mRNA	-0.605010939	6.47E-04
hsa_circ_0081567	circRNA	-0.605072769	0.015088425
hsa_circ_0130893	circRNA	-0.605377675	0.045874225
BGIG9606_66731	lncRNA	-0.605662775	0.02014644

hsa_circ_0065702	circRNA	-0.60570472	0.032202778
hsa_circ_0116839	circRNA	-0.60661714	0.027602952
CBS	mRNA	-0.606629883	0.014914982
hsa_circ_0074690	circRNA	-0.607160445	0.038330284
COL4A2	mRNA	-0.60736004	1.41E-06
hsa_circ_0086375	circRNA	-0.607596409	0.018949953
hsa_circ_0003705	circRNA	-0.607732076	0.013244599
FGD4	mRNA	-0.609048796	2.08E-04
hsa_circ_0066346	circRNA	-0.610130493	0.027505381
TXNRD2	mRNA	-0.610137871	0.04298137
hsa_circ_0030846	circRNA	-0.610636055	0.024221346
hsa_circ_0064711	circRNA	-0.610768964	1.31E-04
hsa_circ_0072007	circRNA	-0.61113907	0.014613435
hsa_circ_0087835	circRNA	-0.61133082	0.001284497
TWSG1	mRNA	-0.61147242	0.001373478
hsa_circ_0066345	circRNA	-0.612401609	0.022461612
CGNL1	mRNA	-0.613102469	0.024958415
EVA1A	mRNA	-0.613301788	0.033277584
hsa_circ_0126378	circRNA	-0.613436941	0.025915174
hsa_circ_0096397	circRNA	-0.613502576	0.021712416
CTNNBIP1	mRNA	-0.615171051	1.60E-04
TMEM25	mRNA	-0.615268449	0.007755051
RAB38	mRNA	-0.615415428	0.00244785
hsa_circ_0003006	circRNA	-0.616594908	0.004470026
LOC285074	pseudo	-0.6175185	9.51E-04

MACROD1	mRNA	-0.618943466	0.00217611
hsa_circ_0001018	circRNA	-0.619564625	0.011641494
hsa_circ_0020305	circRNA	-0.619639058	0.010917525
hsa_circ_0043671	circRNA	-0.62032345	0.002936228
hsa_circ_0128534	circRNA	-0.620534583	0.038307745
hsa_circ_0050533	circRNA	-0.620609706	4.87E-04
hsa_circ_0128535	circRNA	-0.620644078	0.003242139
hsa_circ_0035875	circRNA	-0.620982693	0.030641715
hsa_circ_0010686	circRNA	-0.621407753	0.016570356
PAFAH1B3	mRNA	-0.621643358	2.34E-05
MARCKS	mRNA	-0.622235424	0.004044952
PDE7B	mRNA	-0.623291046	0.042895515
hsa_circ_0075020	circRNA	-0.623308764	3.75E-04
hsa_circ_0010600	circRNA	-0.624699398	0.018409932
THEMIS2	mRNA	-0.624702014	0.002311047
hsa_circ_0055814	circRNA	-0.625271178	0.004084694
hsa_circ_0010097	circRNA	-0.625335541	0.019696682
hsa_circ_0066295	circRNA	-0.625454969	0.025509409
ANXA2R	mRNA	-0.625635289	0.030876254
CREBRF	mRNA	-0.625710232	0.019699772
hsa_circ_0066308	circRNA	-0.626691941	0.007236394
LXN	mRNA	-0.626762967	0.009147687
ZNF713	mRNA	-0.627130661	0.001289198
OPRL1	mRNA	-0.62788942	0.006489973
hsa_circ_0005351	circRNA	-0.628601968	0.012421973

hsa_circ_0070664	circRNA	-0.628681201	0.036292609
PIP4K2C	mRNA	-0.628979031	1.17E-04
CISH	mRNA	-0.629349697	0.005092511
BGIG9606_45434	lncRNA	-0.63044629	0.037141748
hsa_circ_0097495	circRNA	-0.630854308	0.00497594
hsa_circ_0054259	circRNA	-0.631304741	0.01172375
GTF2IRD2B	mRNA	-0.631591135	0.019649265
hsa_circ_0058098	circRNA	-0.631960928	0.010395205
hsa_circ_0010609	circRNA	-0.63220186	0.003868633
hsa_circ_0052598	circRNA	-0.632993773	0.002702957
TET1	mRNA	-0.6331522	2.06E-06
ATP8A1	mRNA	-0.633166675	0.038233929
hsa_circ_0110048	circRNA	-0.633954969	0.028255625
HOMER3	mRNA	-0.634613887	0.001714221
CTSC	mRNA	-0.635101328	4.01E-06
FOXO1	mRNA	-0.635495256	0.046858899
GLS	mRNA	-0.635541057	2.84E-07
hsa_circ_0071980	circRNA	-0.635664446	0.024099165
hsa_circ_0058138	circRNA	-0.635729975	0.007322884
ZNF850	mRNA	-0.635913964	0.004332433
ADGRL1	mRNA	-0.636465221	2.08E-04
hsa_circ_0114304	circRNA	-0.636746518	0.003242494
hsa_circ_0026490	circRNA	-0.637490245	7.18E-05
hsa_circ_0000061	circRNA	-0.6380678	0.045660896
LDLRAD4	mRNA	-0.638341407	0.001026797

NMRK1	mRNA	-0.639374941	1.86E-04
hsa_circ_0060416	circRNA	-0.639649515	0.014149215
FBXO42	mRNA	-0.640105175	2.21E-04
YAP1	mRNA	-0.642043463	0.003952064
hsa_circ_0007075	circRNA	-0.642252073	0.005759019
AXIN2	mRNA	-0.642267807	0.00948554
hsa_circ_0047390	circRNA	-0.642668916	9.81E-05
LINC01776	lncRNA	-0.643236625	0.006758179
hsa_circ_0115349	circRNA	-0.643297505	0.008319373
hsa_circ_0010601	circRNA	-0.643799598	0.003028845
hsa_circ_0137624	circRNA	-0.64392923	0.001829947
TP53INP1	mRNA	-0.644004792	7.84E-05
hsa_circ_0135246	circRNA	-0.644536349	0.025472333
AP1B1	mRNA	-0.644784478	0.017653916
CHST15	mRNA	-0.64495756	0.007546315
hsa_circ_0087289	circRNA	-0.645463084	0.02373636
hsa_circ_0010146	circRNA	-0.646059209	0.024456011
SORD	mRNA	-0.646223203	4.60E-05
hsa_circ_0070054	circRNA	-0.646273776	0.019187688
hsa_circ_0002198	circRNA	-0.646334951	0.016173303
hsa_circ_0091638	circRNA	-0.646454483	0.001040669
hsa_circ_0072014	circRNA	-0.647450231	2.88E-04
TMEM44	mRNA	-0.647566862	0.002595324
hsa_circ_0136471	circRNA	-0.64832664	0.015059445
DSP	mRNA	-0.648671003	0.02119597

hsa_circ_0010608	circRNA	-0.64890019	0.005330204
hsa_circ_0013332	circRNA	-0.648958839	0.027181388
CIRBP	mRNA	-0.649063083	8.86E-04
hsa_circ_0060889	circRNA	-0.65017059	0.011053854
C5	mRNA	-0.650674039	0.014221976
ARRDC4	mRNA	-0.65109582	0.009720561
PECR	mRNA	-0.652637711	0.02215003
hsa_circ_0015420	circRNA	-0.654268299	0.001517533
ZNF425	mRNA	-0.654860016	0.04884558
KLF12	mRNA	-0.654926956	0.002534458
BTG1	mRNA	-0.655169036	0.00132612
hsa_circ_0104124	circRNA	-0.65569458	0.002486727
FAM172A	mRNA	-0.655871119	2.61E-04
hsa_circ_0066067	circRNA	-0.656518253	0.01670908
PIK3CD	mRNA	-0.65652152	0.011793361
hsa_circ_0081574	circRNA	-0.656756513	0.002182609
SPINT1	mRNA	-0.656774207	0.007700964
MSI2	mRNA	-0.657124709	0.006017906
hsa_circ_0128403	circRNA	-0.657690339	0.003635015
hsa_circ_0006887	circRNA	-0.658691463	0.03687346
hsa_circ_0058140	circRNA	-0.659473524	0.006475741
hsa_circ_0109320	circRNA	-0.659585391	0.029063002
hsa_circ_0030847	circRNA	-0.659663183	9.23E-05
ZBED3-AS1	lncRNA	-0.659715714	0.00270801
hsa_circ_0120084	circRNA	-0.661889019	0.001071052

ZC3H12B	mRNA	-0.662035233	0.046571608
WNK3	mRNA	-0.662219799	6.54E-06
hsa_circ_0044436	circRNA	-0.66245423	0.001672027
TLE1	mRNA	-0.662847105	1.04E-06
TRIM59	mRNA	-0.663369171	0.001415599
hsa_circ_0010702	circRNA	-0.663634977	0.003603997
hsa_circ_0055981	circRNA	-0.663642692	2.93E-04
hsa_circ_0061567	circRNA	-0.664659022	0.043029095
LOC105369968	lncRNA	-0.664860088	0.001737288
ADM	mRNA	-0.664969399	0.039254668
hsa_circ_0066301	circRNA	-0.665153877	0.005560124
hsa_circ_0006969	circRNA	-0.665284506	0.018240234
PRRG2	mRNA	-0.665753178	0.040941399
hsa_circ_0128553	circRNA	-0.66590231	0.005249405
CCNJ	mRNA	-0.666090125	9.50E-05
HEPH	mRNA	-0.666163348	0.00127287
hsa_circ_0086376	circRNA	-0.667646638	0.00434195
ARHGEF28	mRNA	-0.667929944	0.008188938
LOC374443	pseudo	-0.668051859	0.001835168
TIPARP	mRNA	-0.668068551	0.015431599
hsa_circ_0042242	circRNA	-0.668169028	0.00244015
hsa_circ_0073120	circRNA	-0.668790908	0.016629964
PLCG2	mRNA	-0.669575286	0.002914313
hsa_circ_0047391	circRNA	-0.669662364	9.97E-06
H2AFZ	mRNA	-0.670240305	0.007091326

hsa_circ_0056395	circRNA	-0.670391043	0.004514168
hsa_circ_0066370	circRNA	-0.67122586	0.040697088
CERK	mRNA	-0.671736221	0.00934573
hsa_circ_0077626	circRNA	-0.673229815	0.044964598
ERCC6	mRNA	-0.673450861	5.41E-04
hsa_circ_0066302	circRNA	-0.673462028	0.004539567
MYOZ3	mRNA	-0.673566436	0.012071219
GALNT10	mRNA	-0.673765174	0.003301491
hsa_circ_0115107	circRNA	-0.673771091	0.010423523
hsa_circ_0072995	circRNA	-0.673959969	0.004084694
hsa_circ_0126694	circRNA	-0.674484766	0.004084694
hsa_circ_0094053	circRNA	-0.674658358	0.004852737
hsa_circ_0072994	circRNA	-0.674715273	0.006736439
hsa_circ_0087317	circRNA	-0.675154	2.38E-04
hsa_circ_0045476	circRNA	-0.675219378	0.010131547
FOXP4	mRNA	-0.675497322	0.004888155
TMEM132A	mRNA	-0.676189286	3.10E-04
PBXIP1	mRNA	-0.676189494	6.44E-05
PLA2R1	mRNA	-0.676377503	0.013650657
hsa_circ_0090061	circRNA	-0.676671327	4.93E-04
hsa_circ_0015424	circRNA	-0.676775145	0.022490348
hsa_circ_0043498	circRNA	-0.676948475	0.006298437
hsa_circ_0006310	circRNA	-0.677035143	0.005432091
RASL11A	mRNA	-0.677036523	0.0455665
hsa_circ_0010591	circRNA	-0.67736606	0.001484908

hsa_circ_0105104	circRNA	-0.677396061	0.004539567
hsa_circ_0058136	circRNA	-0.677999989	0.004495211
HSPG2	mRNA	-0.678027923	0.046325724
hsa_circ_0036064	circRNA	-0.678054944	8.28E-04
RRAGB	mRNA	-0.678073403	4.86E-05
hsa_circ_0041178	circRNA	-0.679716936	0.00483392
hsa_circ_0008753	circRNA	-0.680609274	1.35E-04
hsa_circ_0008348	circRNA	-0.680609274	1.35E-04
hsa_circ_0081923	circRNA	-0.681657698	0.021005539
hsa_circ_0039154	circRNA	-0.681902962	0.010215231
ARL6IP1	mRNA	-0.682417553	0.024259919
hsa_circ_0072996	circRNA	-0.682934005	0.004717058
ZNF608	mRNA	-0.683011806	6.25E-04
hsa_circ_0063841	circRNA	-0.683300792	0.005823041
hsa_circ_0093190	circRNA	-0.683398231	0.016999395
hsa_circ_0067185	circRNA	-0.683705445	0.022001684
hsa_circ_0046855	circRNA	-0.684337824	0.006271121
hsa_circ_0066330	circRNA	-0.684457649	0.019002115
hsa_circ_0080094	circRNA	-0.684687513	0.015368585
LOC100506731	lncRNA	-0.685067166	2.77E-04
hsa_circ_0061748	circRNA	-0.685115971	0.012649075
hsa_circ_0072515	circRNA	-0.685388253	0.006750892
hsa_circ_0010595	circRNA	-0.685522539	6.53E-04
PARD3	mRNA	-0.685899551	6.22E-07
hsa_circ_0010605	circRNA	-0.686124299	3.42E-04

hsa_circ_0058120	circRNA	-0.68634062	0.004369961
hsa_circ_0079629	circRNA	-0.686518823	0.017491457
ZMAT3	mRNA	-0.686947998	0.004562549
hsa_circ_0025491	circRNA	-0.687113467	0.002959365
MPZL2	mRNA	-0.687131154	0.016956245
hsa_circ_0015427	circRNA	-0.687180266	9.36E-04
hsa_circ_0068072	circRNA	-0.688067553	5.73E-04
hsa_circ_0057230	circRNA	-0.688447764	0.02431567
FAM171A2	mRNA	-0.688732	3.34E-04
GMFB	mRNA	-0.689085811	7.19E-06
hsa_circ_0075013	circRNA	-0.68937123	4.26E-04
hsa_circ_0058111	circRNA	-0.689491864	0.006121934
PPM1M	mRNA	-0.689595852	7.62E-05
hsa_circ_0010597	circRNA	-0.689750182	5.14E-04
hsa_circ_0010687	circRNA	-0.68997018	0.004241963
hsa_circ_0039155	circRNA	-0.690597498	0.004443958
hsa_circ_0116809	circRNA	-0.691629385	0.001639596
hsa_circ_0132990	circRNA	-0.693112288	0.042824532
hsa_circ_0066366	circRNA	-0.693200705	0.006460929
hsa_circ_0050183	circRNA	-0.693435073	6.76E-04
PARD6G	mRNA	-0.693621305	0.034460082
LOC100130987	lncRNA	-0.693940331	0.023790053
hsa_circ_0116928	circRNA	-0.694358233	2.56E-04
hsa_circ_0010606	circRNA	-0.694424646	8.95E-04
COL4A1	mRNA	-0.694781521	4.91E-05

LOC730268	pseudo	-0.695072976	0.022254535
SMIM10L2A	mRNA	-0.695215841	0.0330589
LIMS1	mRNA	-0.695734617	6.49E-04
hsa_circ_0136470	circRNA	-0.696081179	0.001265774
hsa_circ_0010612	circRNA	-0.697781232	3.71E-04
hsa_circ_0081575	circRNA	-0.697878888	0.003736176
hsa_circ_0126377	circRNA	-0.698267445	0.013800157
ARHGEF17	mRNA	-0.698340792	7.92E-04
hsa_circ_0066368	circRNA	-0.698630215	0.025327287
hsa_circ_0010598	circRNA	-0.69864771	6.57E-04
hsa_circ_0071981	circRNA	-0.698868308	0.024800195
SBK1	mRNA	-0.699568115	0.041082698
hsa_circ_0010593	circRNA	-0.699838638	5.00E-04
hsa_circ_0010577	circRNA	-0.700010528	0.013662901
TSPAN5	mRNA	-0.700207831	0.010462387
hsa_circ_0077824	circRNA	-0.700428703	0.001932248
hsa_circ_0001432	circRNA	-0.700440152	0.009106439
hsa_circ_0043666	circRNA	-0.702311607	5.57E-04
IPO5P1	pseudo	-0.703233713	0.004755018
hsa_circ_0058149	circRNA	-0.70332188	7.90E-04
ARHGEF9	mRNA	-0.703513122	0.001067507
KRT18	mRNA	-0.703578179	4.35E-09
hsa_circ_0075014	circRNA	-0.703813343	3.43E-04
hsa_circ_0010614	circRNA	-0.704167401	0.002151747
SMS	mRNA	-0.705318187	8.22E-05

LOC105378663	lncRNA	-0.705523789	0.00244785
hsa_circ_0115105	circRNA	-0.705877528	0.021998896
CUBN	mRNA	-0.706047834	0.001353073
hsa_circ_0093596	circRNA	-0.706370226	0.015381553
hsa_circ_0058139	circRNA	-0.706445532	0.00384998
hsa_circ_0010563	circRNA	-0.706759742	0.009301256
hsa_circ_0010604	circRNA	-0.706936358	4.36E-04
MDFI	mRNA	-0.707546875	0.002629531
hsa_circ_0023979	circRNA	-0.707595433	4.46E-04
hsa_circ_0062762	circRNA	-0.708101269	0.004565275
hsa_circ_0140039	circRNA	-0.708126562	0.046991634
hsa_circ_0083666	circRNA	-0.708221753	1.22E-06
SOCS3	mRNA	-0.708390466	4.71E-05
hsa_circ_0058137	circRNA	-0.708946235	0.001429292
PCDH10	mRNA	-0.709052883	0.035096115
FOXC1	mRNA	-0.709382855	0.018594778
HOXA11-AS	lncRNA	-0.709843431	0.001631688
hsa_circ_0113363	circRNA	-0.710095574	0.006898121
hsa_circ_0010602	circRNA	-0.710501039	2.37E-04
NR1D1	mRNA	-0.710501804	2.69E-04
MYCL	mRNA	-0.710693652	0.006969086
ZSCAN16	mRNA	-0.712501197	1.94E-05
hsa_circ_0010615	circRNA	-0.713348723	0.001291944
hsa_circ_0134719	circRNA	-0.71389801	0.044374932
hsa_circ_0090058	circRNA	-0.714621299	1.66E-05

hsa_circ_0010592	circRNA	-0.715307023	3.60E-04
hsa_circ_0017873	circRNA	-0.7153727	0.001429292
hsa_circ_0047389	circRNA	-0.716177501	0.001518953
ARRDC2	mRNA	-0.716629532	0.003254534
hsa_circ_0104348	circRNA	-0.717197476	0.001407287
hsa_circ_0026911	circRNA	-0.717816053	0.026881972
NR1D2	mRNA	-0.717855495	0.00102238
GCHFR	mRNA	-0.718274044	0.02397727
hsa_circ_0010603	circRNA	-0.718357636	2.37E-04
hsa_circ_0042241	circRNA	-0.718516053	0.00277432
hsa_circ_0083669	circRNA	-0.720049066	0.04120107
hsa_circ_0010688	circRNA	-0.720180689	7.20E-04
hsa_circ_0030835	circRNA	-0.720632115	9.78E-05
RGS3	mRNA	-0.72087284	0.003979019
SEMA6D	mRNA	-0.72138121	1.69E-08
hsa_circ_0045477	circRNA	-0.721402647	0.025397611
hsa_circ_0008727	circRNA	-0.721664132	0.012022164
hsa_circ_0003353	circRNA	-0.722174937	0.03169328
ARHGEF25	mRNA	-0.722176385	0.004859659
ADA	mRNA	-0.722409671	0.003320031
RND2	mRNA	-0.722468019	0.015690656
hsa_circ_0029158	circRNA	-0.722498906	0.005746886
CD302	mRNA	-0.722908946	0.042340322
USP44	mRNA	-0.722932561	0.015785205
LOC101927855	lncRNA	-0.723185387	0.025750408

hsa_circ_0036418	circRNA	-0.723894416	0.007112273
PTCH1	mRNA	-0.724031905	0.002629531
CTSK	mRNA	-0.724244911	6.33E-04
hsa_circ_0093191	circRNA	-0.724378033	0.001528119
hsa_circ_0073719	circRNA	-0.724922672	0.044815114
NXN	mRNA	-0.725188292	1.65E-05
CASC10	lncRNA	-0.72540225	0.017779863
hsa_circ_0010613	circRNA	-0.725446289	0.001188324
hsa_circ_0073737	circRNA	-0.726059164	0.018988939
hsa_circ_0010759	circRNA	-0.726185983	0.008870434
PDE8B	mRNA	-0.726642811	0.027492506
GLIPR1	mRNA	-0.727150209	0.022250304
ASH2L	mRNA	-0.727292861	3.33E-05
NIPBL-DT	lncRNA	-0.727541725	0.001067507
hsa_circ_0006773	circRNA	-0.7277792	6.87E-05
hsa_circ_0010584	circRNA	-0.728068666	0.001044662
hsa_circ_0061011	circRNA	-0.728096022	2.09E-05
TCEANC	mRNA	-0.728107222	0.027546966
hsa_circ_0030860	circRNA	-0.728683406	0.008870434
FOXN3	mRNA	-0.72920898	1.48E-14
hsa_circ_0005475	circRNA	-0.729628599	4.90E-05
hsa_circ_0056122	circRNA	-0.730384512	0.026908592
hsa_circ_0008135	circRNA	-0.730431563	4.46E-04
CD37	mRNA	-0.730798119	0.027707067
hsa_circ_0083941	circRNA	-0.731550186	0.003413311

hsa_circ_0010599	circRNA	-0.732889496	0.001809784
SLC45A3	mRNA	-0.732998309	1.20E-04
hsa_circ_0079665	circRNA	-0.733231465	3.56E-05
hsa_circ_0083290	circRNA	-0.733955997	0.01359331
MIR4435-2HG	lncRNA	-0.734347933	5.96E-04
hsa_circ_0054286	circRNA	-0.734350655	0.036292609
hsa_circ_0028873	circRNA	-0.734711815	0.021458675
PHACTR2	mRNA	-0.735266369	0.024372654
hsa_circ_0107466	circRNA	-0.735662331	2.37E-04
ZNF395	mRNA	-0.736585866	0.001487606
hsa_circ_0062759	circRNA	-0.736726188	0.014917608
ARL4A	mRNA	-0.737945597	0.02063051
IFITM10	mRNA	-0.738135839	6.19E-04
hsa_circ_0067593	circRNA	-0.738148051	0.010370964
hsa_circ_0030863	circRNA	-0.738777292	0.029811942
hsa_circ_0126381	circRNA	-0.739452171	0.041031438
PDCD4	mRNA	-0.739625812	0.004755018
ADA2	mRNA	-0.739974273	8.10E-05
hsa_circ_0069320	circRNA	-0.74048901	0.01504697
C9orf116	mRNA	-0.741576434	0.043202536
hsa_circ_0010766	circRNA	-0.741746807	0.033376989
ADGRA3	mRNA	-0.74189616	1.69E-08
hsa_circ_0008130	circRNA	-0.742751453	0.035998355
hsa_circ_0007304	circRNA	-0.742875825	0.002655274
hsa_circ_0060206	circRNA	-0.743449647	0.001262878

PTER	mRNA	-0.744709828	0.001525228
hsa_circ_0113002	circRNA	-0.744772545	3.39E-04
CALD1	mRNA	-0.745429258	0.00338705
hsa_circ_0010765	circRNA	-0.745530996	0.023592469
hsa_circ_0137021	circRNA	-0.746423445	0.003736176
hsa_circ_0130895	circRNA	-0.746885702	0.008676982
GPX3	mRNA	-0.746951913	0.028155281
ARHGEF26	mRNA	-0.747415741	0.013345112
LIMCH1	mRNA	-0.747922611	0.020718445
hsa_circ_0060203	circRNA	-0.748118031	5.15E-04
LOC101927752	lncRNA	-0.748204827	0.046373055
SELENBP1	mRNA	-0.748384486	4.15E-04
hsa_circ_0010673	circRNA	-0.749773281	0.006830087
hsa_circ_0005498	circRNA	-0.749974275	0.03848726
hsa_circ_0026518	circRNA	-0.750027898	0.004656023
ZNF596	mRNA	-0.750439541	0.002370888
BGIG9606_45304	lncRNA	-0.750733453	0.006660353
HMGN3	mRNA	-0.751034652	0.009367111
HOXD4	mRNA	-0.751697647	0.038433219
FAM43A	mRNA	-0.752521085	0.002288278
ANP32A	mRNA	-0.752564293	8.31E-05
hsa_circ_0010587	circRNA	-0.754533147	0.00405848
HOXA6	mRNA	-0.754861129	0.026751008
hsa_circ_0066296	circRNA	-0.754899191	0.047559736
hsa_circ_0058975	circRNA	-0.755618919	0.00792487

NR2F1-AS1	lncRNA	-0.755854509	0.041211347
hsa_circ_0050276	circRNA	-0.756768516	0.018578607
STAB1	mRNA	-0.757170881	0.004234663
hsa_circ_0088153	circRNA	-0.757902332	4.75E-04
AMOT	mRNA	-0.758022999	0.011492191
hsa_circ_0051260	circRNA	-0.758216988	0.004470026
SMARCD3	mRNA	-0.758682573	0.003868597
VWA5A	mRNA	-0.758966358	0.003331168
RAB3IL1	mRNA	-0.760566724	1.66E-05
hsa_circ_0134063	circRNA	-0.761317141	0.046697884
hsa_circ_0078303	circRNA	-0.761536569	0.039072024
hsa_circ_0015564	circRNA	-0.761664388	0.046354475
hsa_circ_0122926	circRNA	-0.761771306	0.03642235
hsa_circ_0010722	circRNA	-0.762635334	0.00212471
GPC2	mRNA	-0.762868993	0.029568482
hsa_circ_0000354	circRNA	-0.763961709	0.007548011
hsa_circ_0010543	circRNA	-0.765445596	5.73E-04
DPP4	mRNA	-0.766989397	0.007370141
PER2	mRNA	-0.766993568	1.66E-05
SNED1	mRNA	-0.766998401	0.0012571
hsa_circ_0019667	circRNA	-0.767123161	7.90E-07
hsa_circ_0112913	circRNA	-0.767149337	0.028794643
hsa_circ_0019670	circRNA	-0.767568251	3.20E-06
hsa_circ_0090998	circRNA	-0.768170539	0.007322884
C12orf75	mRNA	-0.769336056	0.019513347

UAP1L1	mRNA	-0.770609147	3.95E-10
KAZALD1	mRNA	-0.772186587	0.048638348
hsa_circ_0103996	circRNA	-0.77242871	0.030269568
hsa_circ_0010588	circRNA	-0.772439853	4.55E-04
hsa_circ_0117976	circRNA	-0.772460388	0.014440577
HOXA3	mRNA	-0.772747412	0.00329005
hsa_circ_0027603	circRNA	-0.772898529	0.006056347
ZBTB8A	mRNA	-0.772919197	5.19E-05
hsa_circ_0070488	circRNA	-0.77296257	0.006622995
hsa_circ_0026492	circRNA	-0.773006742	3.72E-07
hsa_circ_0048052	circRNA	-0.773508533	0.017145413
LOC107984660	lncRNA	-0.774372549	0.039753254
hsa_circ_0077072	circRNA	-0.774623059	0.024228166
hsa_circ_0010692	circRNA	-0.778297677	0.002337427
hsa_circ_0125865	circRNA	-0.778371563	0.008847944
MCCC1	mRNA	-0.778847353	2.90E-07
SHC2	mRNA	-0.778964825	0.002280008
hsa_circ_0010594	circRNA	-0.779448038	9.73E-05
FAM184A	mRNA	-0.779673082	0.037606746
GAB3	mRNA	-0.780703693	8.55E-06
hsa_circ_0066294	circRNA	-0.780746901	0.023833786
OTUD3	mRNA	-0.780858783	1.18E-04
MVB12B	mRNA	-0.780954925	7.96E-06
hsa_circ_0062754	circRNA	-0.781101779	0.006573202
SLC7A7	mRNA	-0.782114597	0.002650384

OAZ2	mRNA	-0.78247997	7.18E-04
BGIG9606_36118	lncRNA	-0.783244041	0.011494938
CDCA7L	mRNA	-0.78401157	1.10E-05
hsa_circ_0087325	circRNA	-0.784426756	7.02E-04
hsa_circ_0010564	circRNA	-0.784571217	7.52E-05
hsa_circ_0010723	circRNA	-0.784820045	0.011111713
hsa_circ_0115299	circRNA	-0.7855561	0.010730695
RNF208	mRNA	-0.785714138	0.001209727
RHPN2	mRNA	-0.786967261	0.002309281
TXNDC16	mRNA	-0.78766442	7.26E-09
hsa_circ_0010778	circRNA	-0.787916531	0.038848686
hsa_circ_0076291	circRNA	-0.788034291	0.024268523
hsa_circ_0115320	circRNA	-0.788225822	3.48E-04
hsa_circ_0058977	circRNA	-0.788924169	0.009301256
USP53	mRNA	-0.78901004	2.49E-07
SESN3	mRNA	-0.789395581	0.028919096
LIAS	mRNA	-0.791100979	0.002699783
FBLIM1	mRNA	-0.791119825	1.07E-04
hsa_circ_0004931	circRNA	-0.791420053	1.18E-05
TSPY26P	pseudo	-0.792322563	5.39E-05
hsa_circ_0008914	circRNA	-0.792360375	0.005006995
hsa_circ_0078302	circRNA	-0.792381357	0.011012522
LOC107987392	lncRNA	-0.79246117	0.005203441
ETNK2	mRNA	-0.792808561	4.24E-06
SLC9A9	mRNA	-0.793470484	8.73E-05

DENND5B	mRNA	-0.793561899	1.68E-06
hsa_circ_0010756	circRNA	-0.794056068	0.033299767
hsa_circ_0003964	circRNA	-0.794752665	0.021138906
hsa_circ_0010610	circRNA	-0.794910744	4.21E-05
hsa_circ_0092279	circRNA	-0.794947831	0.005019517
LOC105372401	lncRNA	-0.795416193	0.011830975
hsa_circ_0136553	circRNA	-0.795913045	0.028835095
hsa_circ_0122916	circRNA	-0.795933941	0.011985023
NMNAT3	mRNA	-0.797439918	0.006998568
hsa_circ_0056121	circRNA	-0.798094479	0.012094299
MYRIP	mRNA	-0.7981138	0.034572929
hsa_circ_0072358	circRNA	-0.798217029	0.019585377
hsa_circ_0030839	circRNA	-0.798565124	1.37E-04
hsa_circ_0026517	circRNA	-0.798954609	0.001158054
CD200	mRNA	-0.800466883	0.034621156
hsa_circ_0097618	circRNA	-0.800532992	0.003139023
hsa_circ_0077130	circRNA	-0.800693228	0.023862771
hsa_circ_0025476	circRNA	-0.801907265	0.007455409
DDIT4L	mRNA	-0.803004153	0.015490645
PDCD4-AS1	lncRNA	-0.80314512	0.033081296
hsa_circ_0054284	circRNA	-0.803179566	7.46E-05
hsa_circ_0026504	circRNA	-0.803187357	1.50E-04
hsa_circ_0128658	circRNA	-0.803362122	0.031014981
hsa_circ_0026533	circRNA	-0.803713244	3.49E-09
YPEL1	mRNA	-0.803875204	0.010808541

hsa_circ_0010596	circRNA	-0.80403265	0.021791097
SAT2	mRNA	-0.804304657	9.54E-08
hsa_circ_0010771	circRNA	-0.804636833	0.001519666
hsa_circ_0012758	circRNA	-0.804714737	0.001707158
hsa_circ_0124899	circRNA	-0.805153125	0.002568731
hsa_circ_0112548	circRNA	-0.805264225	0.002365142
hsa_circ_0085381	circRNA	-0.805297908	0.039651275
hsa_circ_0010143	circRNA	-0.806043354	0.005637714
DANCR	lncRNA	-0.80683896	6.19E-10
C1QTNF4	mRNA	-0.80752708	0.00516974
NUDT4	mRNA	-0.80766777	1.73E-05
FZD2	mRNA	-0.807826677	0.00516974
HCG11	lncRNA	-0.808194933	2.06E-04
TIGD7	mRNA	-0.808527235	8.21E-06
PGAP1	mRNA	-0.808743021	0.001569091
hsa_circ_0062757	circRNA	-0.809123693	3.52E-05
TSGA10	mRNA	-0.809775213	0.00556318
GSTM4	mRNA	-0.811145659	0.00382871
hsa_circ_0015055	circRNA	-0.812288931	0.003716906
hsa_circ_0110195	circRNA	-0.813595696	3.67E-06
hsa_circ_0040622	circRNA	-0.814265502	0.012921852
hsa_circ_0137830	circRNA	-0.814889571	0.04958186
CDK19	mRNA	-0.814902543	5.31E-05
hsa_circ_0029157	circRNA	-0.815919369	0.039916954
FRMD6	mRNA	-0.816284378	0.005282085

MANBA	mRNA	-0.816364528	1.02E-07
HERC2	mRNA	-0.81828345	0.019869217
RPS6KA6	mRNA	-0.819068042	0.009561102
LOC107984293	lncRNA	-0.81921925	0.001009221
hsa_circ_0010740	circRNA	-0.819327023	0.025915174
hsa_circ_0117515	circRNA	-0.819836426	0.019465849
hsa_circ_0009407	circRNA	-0.820401499	5.27E-04
IL13RA1	mRNA	-0.820597608	5.95E-04
hsa_circ_0080755	circRNA	-0.820910488	4.13E-04
FZD5	mRNA	-0.821640873	0.020218443
YPEL2	mRNA	-0.822264156	0.01134317
LOC101928570	lncRNA	-0.822449944	0.010392353
hsa_circ_0015400	circRNA	-0.822599625	0.00541829
hsa_circ_0030842	circRNA	-0.823207779	0.009520551
hsa_circ_0090060	circRNA	-0.823878164	0.002726843
EFNB2	mRNA	-0.824006124	5.05E-05
hsa_circ_0026523	circRNA	-0.824256118	0.001404631
hsa_circ_0057874	circRNA	-0.824716294	4.28E-06
hsa_circ_0125519	circRNA	-0.824770143	0.001440697
DHRS3	mRNA	-0.825243771	6.25E-04
hsa_circ_0035844	circRNA	-0.825555373	2.01E-04
LOC105375304	lncRNA	-0.825595003	0.001032407
ZC3HAV1	mRNA	-0.82578411	3.07E-04
hsa_circ_0014156	circRNA	-0.826381189	0.003334934
hsa_circ_0024096	circRNA	-0.82656486	3.74E-04

hsa_circ_0081924	circRNA	-0.827458167	8.41E-04
REPS2	mRNA	-0.828105424	5.36E-05
hsa_circ_0110977	circRNA	-0.82907752	3.31E-05
TMEM120A	mRNA	-0.829205519	1.05E-04
hsa_circ_0010582	circRNA	-0.829505481	0.043323806
SPOCK1	mRNA	-0.829675529	0.025461186
hsa_circ_0031835	circRNA	-0.830158311	0.034106626
hsa_circ_0099818	circRNA	-0.830410788	0.008433191
NSUN7	mRNA	-0.830711766	0.038769901
hsa_circ_0020274	circRNA	-0.830763357	0.033110565
LOC107984974	mRNA	-0.830946666	0.010020892
ROBO1	mRNA	-0.831195531	0.018594778
STK33	mRNA	-0.831315593	0.004541713
LOC100506804	lncRNA	-0.831837735	7.80E-04
hsa_circ_0088610	circRNA	-0.831838181	0.009652962
hsa_circ_0026532	circRNA	-0.831877371	0.001478924
VPS37D	mRNA	-0.832051478	0.001422597
hsa_circ_0069613	circRNA	-0.832102561	0.04588573
hsa_circ_0007889	circRNA	-0.83225239	7.06E-11
FLVCR2	mRNA	-0.832264231	0.002534385
hsa_circ_0073363	circRNA	-0.833725288	0.010466262
hsa_circ_0004161	circRNA	-0.835607515	0.013431524
RUNX1T1	mRNA	-0.836161071	0.03099164
hsa_circ_0006323	circRNA	-0.83656273	0.014319667
hsa_circ_0031908	circRNA	-0.837443784	0.004742786

PMEPA1	mRNA	-0.83860875	3.95E-05
hsa_circ_0123086	circRNA	-0.839001831	0.006385675
hsa_circ_0010568	circRNA	-0.839265772	0.003242139
SOCS2-AS1	lncRNA	-0.839313759	0.008527411
ZMYND8	mRNA	-0.839340175	1.73E-05
hsa_circ_0026505	circRNA	-0.841397077	2.05E-10
RNF150	mRNA	-0.8418578	0.010080155
NUDT4B	mRNA	-0.84277699	0.045716519
hsa_circ_0055976	circRNA	-0.842801355	2.86E-06
SPOCD1	mRNA	-0.84303342	4.75E-04
hsa_circ_0081907	circRNA	-0.843066269	2.80E-04
BGIG9606_37243	lncRNA	-0.843635808	0.024372654
hsa_circ_0026516	circRNA	-0.843756771	4.69E-09
hsa_circ_0030852	circRNA	-0.844194263	2.90E-06
HES4	mRNA	-0.845445715	0.009974154
GATA3-AS1	lncRNA	-0.846159722	0.002924838
hsa_circ_0026496	circRNA	-0.846575339	1.60E-12
MEGF6	mRNA	-0.847149714	0.017399635
hsa_circ_0066309	circRNA	-0.847657005	0.039956745
hsa_circ_0066568	circRNA	-0.848823812	0.029511885
LOC107987281	lncRNA	-0.849669526	0.033800393
hsa_circ_0134066	circRNA	-0.85001895	2.64E-05
hsa_circ_0030773	circRNA	-0.85086767	0.008622301
hsa_circ_0049587	circRNA	-0.851633168	0.023909895
TFDP2	mRNA	-0.851668018	9.66E-07

ADGRA2	mRNA	-0.852438589	3.63E-04
SPNS2	mRNA	-0.853037509	0.02521476
hsa_circ_0037943	circRNA	-0.853908867	0.028187112
IFI30	mRNA	-0.853982953	0.034708503
hsa_circ_0010624	circRNA	-0.854053446	3.48E-04
hsa_circ_0113762	circRNA	-0.854470908	1.66E-06
hsa_circ_0063864	circRNA	-0.854570694	0.023441384
ERRFI1	mRNA	-0.855284176	0.00560686
CCNG2	mRNA	-0.855798409	6.56E-07
hsa_circ_0115305	circRNA	-0.855966002	1.98E-04
FBXO32	mRNA	-0.856790193	6.07E-05
hsa_circ_0132899	circRNA	-0.857407038	0.019237932
F2RL1	mRNA	-0.857836253	1.30E-07
hsa_circ_0044669	circRNA	-0.858255439	0.024791425
hsa_circ_0060596	circRNA	-0.858590514	0.013662901
hsa_circ_0031912	circRNA	-0.860116043	0.011553426
hsa_circ_0049736	circRNA	-0.860330183	0.019402007
FABP5	mRNA	-0.861084708	1.43E-05
hsa_circ_0068550	circRNA	-0.862584431	9.89E-05
WWC2-AS2	lncRNA	-0.863203228	0.013826249
EIF4B	mRNA	-0.864145228	2.23E-15
ZSWIM5	mRNA	-0.864623382	6.85E-04
hsa_circ_0081908	circRNA	-0.865083244	0.013653071
hsa_circ_0108089	circRNA	-0.8652398	0.004850044
BNC2	mRNA	-0.86564407	5.12E-04

hsa_circ_0085444	circRNA	-0.866838453	0.035224063
hsa_circ_0045148	circRNA	-0.866911044	0.004041322
hsa_circ_0030772	circRNA	-0.867420934	0.011755614
hsa_circ_0093194	circRNA	-0.867735496	0.005443429
hsa_circ_0137993	circRNA	-0.867795422	0.002741436
MAN1C1	mRNA	-0.868925715	0.010814688
TRO	mRNA	-0.869321877	5.69E-04
JPH3	mRNA	-0.869577396	0.046807473
LOC102723409	lncRNA	-0.869787069	0.006905957
hsa_circ_0057338	circRNA	-0.870040264	0.036751974
TMEM86A	mRNA	-0.871460764	0.001422597
hsa_circ_0062760	circRNA	-0.871608846	9.02E-05
ABLIM1	mRNA	-0.872411879	0.010657046
hsa_circ_0026501	circRNA	-0.872500249	4.64E-05
hsa_circ_0044995	circRNA	-0.872992175	0.037222014
GLRB	mRNA	-0.87311432	3.49E-04
hsa_circ_0077942	circRNA	-0.873155971	3.70E-04
PTPRD	mRNA	-0.873179781	7.89E-06
hsa_circ_0010727	circRNA	-0.873289471	5.05E-04
hsa_circ_0109097	circRNA	-0.875208617	1.31E-06
hsa_circ_0026534	circRNA	-0.875548042	5.94E-11
SAMD13	mRNA	-0.875798753	2.85E-06
ZNF423	mRNA	-0.876202514	5.80E-05
hsa_circ_0130319	circRNA	-0.876825636	0.041463218
GSTM2	mRNA	-0.877087199	0.007080065

hsa_circ_0010681	circRNA	-0.8786712	3.00E-05
hsa_circ_0089627	circRNA	-0.879138662	0.008059849
hsa_circ_0011173	circRNA	-0.879421521	8.56E-05
hsa_circ_0026499	circRNA	-0.881097732	1.50E-05
hsa_circ_0054272	circRNA	-0.881640624	2.31E-05
LINC00612	lncRNA	-0.882234629	0.048929647
hsa_circ_0087881	circRNA	-0.882860821	4.85E-06
hsa_circ_0081916	circRNA	-0.883595454	0.001517533
hsa_circ_0115321	circRNA	-0.884148869	0.014359458
F8	mRNA	-0.884514179	0.004035592
hsa_circ_0024423	circRNA	-0.885715299	0.042416333
hsa_circ_0010557	circRNA	-0.885968939	7.36E-04
hsa_circ_0081931	circRNA	-0.886186934	1.97E-04
hsa_circ_0130058	circRNA	-0.886906466	7.37E-04
NEK6	mRNA	-0.887455023	2.61E-11
hsa_circ_0060622	circRNA	-0.888187655	6.74E-06
CAP2	mRNA	-0.888450321	0.001913237
hsa_circ_0007024	circRNA	-0.888714608	0.020338076
ZNF702P	pseudo	-0.88888599	1.37E-05
hsa_circ_0091397	circRNA	-0.889031123	0.012356391
hsa_circ_0095168	circRNA	-0.889079415	0.03850288
LOC100129034	lncRNA	-0.891160128	1.86E-08
hsa_circ_0010571	circRNA	-0.892254021	0.043450978
TNFRSF12A	mRNA	-0.892825164	3.10E-05
ECHDC2	mRNA	-0.893377569	0.019215018

hsa_circ_0031907	circRNA	-0.893443429	0.001961598
EFNA1	mRNA	-0.89385123	2.83E-04
hsa_circ_0041180	circRNA	-0.894341818	1.04E-06
ARMC4	mRNA	-0.894487386	0.022222186
TNFSF4	mRNA	-0.894618388	2.83E-05
hsa_circ_0081928	circRNA	-0.895373742	2.22E-07
hsa_circ_0115317	circRNA	-0.89742276	6.69E-06
hsa_circ_0010738	circRNA	-0.898312765	0.003662344
DMD	mRNA	-0.899311963	0.011166975
NFIA	mRNA	-0.899332797	1.99E-06
CHST1	mRNA	-0.899861427	6.95E-06
hsa_circ_0010695	circRNA	-0.900031629	0.026485668
hsa_circ_0081942	circRNA	-0.900592805	3.42E-04
hsa_circ_0081943	circRNA	-0.90066401	0.039552359
VDR	mRNA	-0.900939996	0.020814313
hsa_circ_0026497	circRNA	-0.90135227	1.22E-04
hsa_circ_0010721	circRNA	-0.903069421	0.041662926
hsa_circ_0010762	circRNA	-0.903459496	2.83E-05
hsa_circ_0130887	circRNA	-0.903495797	0.008565436
hsa_circ_0010567	circRNA	-0.90376151	4.47E-04
FIGN	mRNA	-0.903969708	2.56E-05
hsa_circ_0066065	circRNA	-0.904523524	0.021458675
ZNF771	mRNA	-0.904586482	4.40E-15
hsa_circ_0013259	circRNA	-0.907921096	4.17E-04
hsa_circ_0070533	circRNA	-0.910728741	0.004591676

hsa_circ_0002345	circRNA	-0.910892864	1.32E-05
hsa_circ_0026512	circRNA	-0.911555233	5.32E-10
hsa_circ_0004972	circRNA	-0.91222235	6.66E-19
hsa_circ_0007748	circRNA	-0.914185183	0.009471121
CA11	mRNA	-0.914868696	6.95E-06
hsa_circ_0014154	circRNA	-0.915071292	0.002259873
CTNNAL1	mRNA	-0.915468453	1.73E-05
hsa_circ_0010098	circRNA	-0.915936052	8.89E-08
SLC16A1-AS1	lncRNA	-0.916532268	0.049544865
hsa_circ_0010682	circRNA	-0.917711372	6.94E-04
CNNM2	mRNA	-0.917880688	1.38E-06
hsa_circ_0030837	circRNA	-0.918459419	1.62E-06
BGIG9606_51041	mRNA	-0.919078085	0.04949379
DTX4	mRNA	-0.919762807	0.008701308
LOC100506178	lncRNA	-0.919776273	0.018989286
hsa_circ_0113761	circRNA	-0.920283987	1.20E-06
hsa_circ_0006955	circRNA	-0.920331724	1.64E-07
hsa_circ_0090059	circRNA	-0.9219334	0.018779596
SLC29A4	mRNA	-0.923023177	2.57E-04
hsa_circ_0087442	circRNA	-0.923390176	0.003443728
RFX2	mRNA	-0.923414717	5.03E-04
hsa_circ_0026500	circRNA	-0.923454937	1.98E-05
hsa_circ_0092666	circRNA	-0.924730642	7.46E-05
hsa_circ_0126168	circRNA	-0.925561724	0.007028205
FOSB	mRNA	-0.925633198	0.016207522

HOMER3-AS1	lncRNA	-0.926185997	0.040653951
hsa_circ_0129828	circRNA	-0.926648694	0.046118005
hsa_circ_0010683	circRNA	-0.926720043	0.008870434
SLC40A1	mRNA	-0.926852835	0.040785252
hsa_circ_0115309	circRNA	-0.927593844	0.030596158
SPECC1	mRNA	-0.928514817	0.001451532
LAMB1	mRNA	-0.928615083	3.82E-08
CDKN1C	mRNA	-0.928771804	1.30E-05
hsa_circ_0060606	circRNA	-0.929273637	0.001600235
LOC101927359	lncRNA	-0.930044641	0.035633316
hsa_circ_0088485	circRNA	-0.931801254	5.04E-06
hsa_circ_0073399	circRNA	-0.932158854	2.98E-04
hsa_circ_0081927	circRNA	-0.932459872	8.12E-07
LOC107984805	lncRNA	-0.933149082	0.025527781
hsa_circ_0006855	circRNA	-0.933331314	5.83E-05
hsa_circ_0007358	circRNA	-0.934503526	0.014115704
NAXE	mRNA	-0.935071724	4.11E-06
hsa_circ_0026509	circRNA	-0.935620231	8.99E-05
hsa_circ_0067823	circRNA	-0.93568508	0.00425103
GCA	mRNA	-0.936933436	0.010905839
hsa_circ_0102326	circRNA	-0.938488388	0.001284497
LOC101927692	lncRNA	-0.938795251	0.013278046
IQCA1	mRNA	-0.938883379	0.005471176
hsa_circ_0034319	circRNA	-0.939601191	8.23E-04
CRISPLD1	mRNA	-0.94020155	6.50E-04

hsa_circ_0073398	circRNA	-0.940495057	0.021918672
hsa_circ_0026530	circRNA	-0.940923677	0.015712013
hsa_circ_0001790	circRNA	-0.941007348	8.29E-07
hsa_circ_0080752	circRNA	-0.941262254	2.06E-10
hsa_circ_0067991	circRNA	-0.944512687	0.007424641
hsa_circ_0081570	circRNA	-0.944749888	0.006455003
C1R	mRNA	-0.944827874	8.87E-05
EIF4EBP3	mRNA	-0.945019052	0.031758786
hsa_circ_0000834	circRNA	-0.945098927	0.026728444
NFATC4	mRNA	-0.945270868	0.002981567
hsa_circ_0137998	circRNA	-0.945390252	0.002811776
FAM84B	mRNA	-0.945750111	0.007788755
hsa_circ_0136473	circRNA	-0.94651037	1.19E-05
PIEZO2	mRNA	-0.946722119	0.001632451
hsa_circ_0036032	circRNA	-0.946780473	0.0142475
CCDC146	mRNA	-0.947044165	0.039308226
HMCN1	mRNA	-0.947264906	0.019010974
hsa_circ_0014452	circRNA	-0.948254106	1.29E-07
hsa_circ_0026529	circRNA	-0.948299729	1.01E-10
hsa_circ_0026514	circRNA	-0.948820066	4.52E-05
LOC105375751	lncRNA	-0.949331865	0.00653406
RAB30	mRNA	-0.949843485	1.54E-05
TFPI	mRNA	-0.950377985	0.001208545
CENPV	mRNA	-0.951191609	0.004355556
CFH	mRNA	-0.951652734	2.35E-04

KLHL4	mRNA	-0.95249248	6.20E-05
TACSTD2	mRNA	-0.952719538	0.009608087
hsa_circ_0026498	circRNA	-0.952731572	5.00E-06
hsa_circ_0002475	circRNA	-0.953049062	0.003600469
hsa_circ_0010559	circRNA	-0.954155821	2.16E-04
hsa_circ_0091395	circRNA	-0.955780179	3.48E-05
PWARSN	lncRNA	-0.956512201	1.15E-04
GPR19	mRNA	-0.956818426	0.011166975
LAPTM4B	mRNA	-0.956983276	0.001171346
hsa_circ_0026531	circRNA	-0.957721785	0.038984901
hsa_circ_0115314	circRNA	-0.957779685	2.15E-06
hsa_circ_0098217	circRNA	-0.95842355	0.016779082
hsa_circ_0088486	circRNA	-0.958619967	1.06E-05
hsa_circ_0087882	circRNA	-0.958832317	0.01887598
hsa_circ_0026520	circRNA	-0.958847437	2.02E-16
hsa_circ_0130059	circRNA	-0.959121856	7.03E-05
hsa_circ_0086371	circRNA	-0.959131264	0.049100487
hsa_circ_0073002	circRNA	-0.959671371	3.12E-05
hsa_circ_0010763	circRNA	-0.959775747	0.020295807
SGK1	mRNA	-0.959892558	1.58E-04
OTOGL	mRNA	-0.96001961	0.002514051
hsa_circ_0091301	circRNA	-0.960381112	0.001770782
hsa_circ_0010100	circRNA	-0.960653487	9.25E-08
BGIG9606_44531	lncRNA	-0.961071412	0.018866827
hsa_circ_0025822	circRNA	-0.96333596	0.031892387

hsa_circ_0118831	circRNA	-0.963949879	0.02290569
SOCS2	mRNA	-0.964095614	0.014860866
TBX1	mRNA	-0.96422118	0.004355556
hsa_circ_0088484	circRNA	-0.965800081	9.23E-06
CABLES1	mRNA	-0.966843247	1.48E-11
hsa_circ_0106062	circRNA	-0.967264331	0.042474019
HERC2P9	pseudo	-0.968028141	0.018812867
hsa_circ_0088494	circRNA	-0.970011231	2.23E-08
PRKACB	mRNA	-0.970590667	1.94E-12
hsa_circ_0064631	circRNA	-0.97391219	0.022045256
hsa_circ_0008879	circRNA	-0.974145466	4.93E-06
hsa_circ_0134073	circRNA	-0.975652468	0.027808718
hsa_circ_0073402	circRNA	-0.976058389	0.042136251
hsa_circ_0056662	circRNA	-0.976384803	0.047991411
TLE2	mRNA	-0.97697032	1.25E-05
PACSIN3	mRNA	-0.97711981	3.23E-15
hsa_circ_0081939	circRNA	-0.978579436	3.22E-06
DNASE2	mRNA	-0.978710058	2.82E-12
hsa_circ_0001952	circRNA	-0.979011789	6.17E-04
hsa_circ_0008176	circRNA	-0.979905512	0.022993872
hsa_circ_0077945	circRNA	-0.980343684	6.15E-04
hsa_circ_0057335	circRNA	-0.981512138	1.51E-05
BGIG9606_37320	lncRNA	-0.98157953	0.014231466
hsa_circ_0097573	circRNA	-0.981638734	0.022967161
FOXC2	mRNA	-0.983007474	0.00106445

hsa_circ_0026522	circRNA	-0.983825367	7.88E-07
hsa_circ_0091204	circRNA	-0.984433796	3.71E-06
hsa_circ_0060496	circRNA	-0.984925181	1.60E-05
hsa_circ_0069322	circRNA	-0.985072969	4.64E-04
hsa_circ_0087880	circRNA	-0.986563453	8.11E-10
GPRC5B	mRNA	-0.986669815	0.002651858
hsa_circ_0026513	circRNA	-0.987046862	4.38E-07
LOXL1-AS1	lncRNA	-0.98822903	0.009873049
hsa_circ_0066058	circRNA	-0.988243577	8.75E-05
BMF	mRNA	-0.988724751	0.015281913
hsa_circ_0126427	circRNA	-0.990012492	0.020150382
hsa_circ_0108099	circRNA	-0.990759625	0.012832231
GKAP1	mRNA	-0.9915178	8.45E-05
hsa_circ_0090870	circRNA	-0.991742934	0.004514168
hsa_circ_0078661	circRNA	-0.991861878	0.018274245
HSD3BP4	pseudo	-0.99206646	0.001153925
MAST1	mRNA	-0.994390668	0.015408748
TLCD2	mRNA	-0.994895682	2.81E-04
CFAP69	mRNA	-0.995546736	2.43E-05
hsa_circ_0088495	circRNA	-0.995548797	5.47E-04
hsa_circ_0003514	circRNA	-0.99649318	0.015797859
NLGN1	mRNA	-0.996782538	0.022118891
hsa_circ_0010755	circRNA	-0.997094893	0.002271902
hsa_circ_0115310	circRNA	-0.997674978	4.07E-07
hsa_circ_0026503	circRNA	-0.998617483	1.57E-06

hsa_circ_0003214	circRNA	-0.998908118	1.17E-07
hsa_circ_0010698	circRNA	-1.000813463	0.003457074
NATD1	mRNA	-1.001989097	1.22E-04
CETP	mRNA	-1.002555243	0.002538407
SLC22A18	mRNA	-1.003120255	0.022788956
LINC00607	lncRNA	-1.004908432	3.27E-09
BGIG9606_44193	mRNA	-1.005214189	0.004039922
TXNDC5	mRNA	-1.005942336	0.004755018
hsa_circ_0027344	circRNA	-1.007711265	0.001491688
hsa_circ_0081940	circRNA	-1.009447658	1.11E-05
hsa_circ_0026506	circRNA	-1.010864658	4.02E-08
hsa_circ_0026508	circRNA	-1.011310475	9.51E-07
hsa_circ_0055924	circRNA	-1.012126138	0.005413735
CLIP3	mRNA	-1.012238377	2.02E-04
hsa_circ_0122924	circRNA	-1.013893306	0.008210695
OR2A9P	pseudo	-1.01468487	0.003910034
hsa_circ_0081917	circRNA	-1.01478898	9.49E-05
hsa_circ_0049046	circRNA	-1.01529272	0.006271121
hsa_circ_0122923	circRNA	-1.015517974	0.003634918
hsa_circ_0075594	circRNA	-1.0164823	0.036751974
LOC100129940	lncRNA	-1.016722543	4.51E-04
hsa_circ_0088492	circRNA	-1.017682639	3.03E-10
hsa_circ_0080754	circRNA	-1.019441954	2.64E-04
hsa_circ_0113747	circRNA	-1.020620843	2.11E-04
MIR100HG	lncRNA	-1.020662903	3.07E-12

ITGA11	mRNA	-1.020962756	0.009720561
MLLT3	mRNA	-1.02172152	2.21E-07
ANKRD53	mRNA	-1.022334273	0.007580016
hsa_circ_0081919	circRNA	-1.024582723	2.68E-10
hsa_circ_0026511	circRNA	-1.026748278	0.003233767
BGIG9606_50074	lncRNA	-1.027657541	0.044570768
SELL	mRNA	-1.028530742	0.007367588
CXCL1	mRNA	-1.030860237	0.012916479
SLC45A4	mRNA	-1.031420339	9.51E-08
hsa_circ_0075900	circRNA	-1.032273865	0.00128504
hsa_circ_0025703	circRNA	-1.032320541	0.045828441
VWA1	mRNA	-1.032355194	3.30E-04
hsa_circ_0060624	circRNA	-1.032556814	4.02E-05
hsa_circ_0076299	circRNA	-1.032845256	0.001050585
hsa_circ_0004864	circRNA	-1.033196476	5.47E-09
hsa_circ_0010023	circRNA	-1.033409303	1.86E-06
hsa_circ_0115322	circRNA	-1.033878513	7.67E-05
KCNT2	mRNA	-1.035106034	0.005527192
hsa_circ_0089955	circRNA	-1.035826233	0.004807319
hsa_circ_0014768	circRNA	-1.036813734	2.53E-09
hsa_circ_0003085	circRNA	-1.037132561	0.019899479
hsa_circ_0118484	circRNA	-1.037557159	0.015368585
NR2F1	mRNA	-1.037744761	0.00240338
HAPLN3	mRNA	-1.038264715	6.88E-05
FGF2	mRNA	-1.03985608	0.032755374

hsa_circ_0081911	circRNA	-1.04042891	2.98E-06
DSEL	mRNA	-1.040581762	0.001091443
hsa_circ_0110378	circRNA	-1.041350099	4.13E-04
ITGB8	mRNA	-1.04224348	0.002920754
CADM1	mRNA	-1.042459156	0.001396811
SYN2	mRNA	-1.042661088	0.028864926
CELSR2	mRNA	-1.042910821	2.12E-09
CCZ1P-OR7E38P	pseudo	-1.043442325	5.54E-04
hsa_circ_0081937	circRNA	-1.043476701	3.52E-05
HMGA2	mRNA	-1.044924155	1.62E-08
LRIG3	mRNA	-1.044977291	0.004994351
KAT7	mRNA	-1.048388845	6.61E-04
GATA3	mRNA	-1.048975425	0.005967476
hsa_circ_0137997	circRNA	-1.049778036	2.55E-04
hsa_circ_0081932	circRNA	-1.051940702	3.38E-06
hsa_circ_0026507	circRNA	-1.052326542	1.04E-07
MTMR10	mRNA	-1.052906679	4.65E-06
hsa_circ_0075595	circRNA	-1.05295148	0.025919002
PURG	mRNA	-1.053270333	5.51E-06
KLHL13	mRNA	-1.053997383	2.12E-05
CEACAM1	mRNA	-1.055055652	0.034775931
FBN2	mRNA	-1.056074931	1.96E-11
HSD17B14	mRNA	-1.056122455	8.63E-13
ZFHX4	mRNA	-1.056639767	0.026090631
hsa_circ_0131149	circRNA	-1.056967947	0.044480223

SLC44A3-AS1	lncRNA	-1.057338892	0.02911009
hsa_circ_0070899	circRNA	-1.057881048	0.001296542
hsa_circ_0021121	circRNA	-1.057936087	0.036215403
ADGRG6	mRNA	-1.058938039	0.032727052
hsa_circ_0115313	circRNA	-1.060144243	0.024218339
BGIG9606_49735	lncRNA	-1.060534401	0.04579236
hsa_circ_0006243	circRNA	-1.061458448	0.015442499
SRRM3	mRNA	-1.061767987	6.14E-06
TEF	mRNA	-1.062925044	3.19E-05
hsa_circ_0002591	circRNA	-1.063140962	3.17E-06
ACKR4	mRNA	-1.063811637	0.002555327
hsa_circ_0075591	circRNA	-1.065109151	0.021421495
GAPLINC	lncRNA	-1.066322423	1.02E-04
MCTP1	mRNA	-1.066363171	5.58E-04
HERC2P3	pseudo	-1.066540121	0.006969086
HSBP1L1	mRNA	-1.069934686	6.82E-07
hsa_circ_0100870	circRNA	-1.070300738	0.022924171
HPSE	mRNA	-1.070614658	1.60E-04
hsa_circ_0102869	circRNA	-1.070618544	0.027496654
hsa_circ_0098379	circRNA	-1.071136918	0.025335459
hsa_circ_0081935	circRNA	-1.071215372	4.24E-06
hsa_circ_0137996	circRNA	-1.071622166	0.004516418
USP2	mRNA	-1.071776821	0.003994223
hsa_circ_0057525	circRNA	-1.074078605	0.028838591
hsa_circ_0018007	circRNA	-1.074938815	0.025919002

BGIG9606_51023	lncRNA	-1.075691549	1.25E-05
hsa_circ_0018184	circRNA	-1.075892924	0.001865749
FOXRED2	mRNA	-1.076224497	4.62E-15
SATB1	mRNA	-1.076241979	2.49E-07
PRICKLE2	mRNA	-1.076904839	0.011231437
TNFRSF10D	mRNA	-1.077363959	3.51E-06
hsa_circ_0010726	circRNA	-1.078429461	0.003402305
ZNF853	mRNA	-1.079002255	6.66E-04
hsa_circ_0114593	circRNA	-1.079003283	5.01E-05
BGIG9606_47663	lncRNA	-1.079910174	0.023336167
FHIT	mRNA	-1.080234869	6.22E-06
ODF3B	mRNA	-1.080329285	0.006553755
hsa_circ_0070536	circRNA	-1.080480166	0.007083612
PCDH18	mRNA	-1.081188109	1.50E-08
hsa_circ_0060803	circRNA	-1.085609828	0.002784227
FLRT2	mRNA	-1.085964229	2.98E-05
NAALAD2	mRNA	-1.086006201	0.001607288
ARHGEF5	mRNA	-1.086823421	0.004885936
CERNA1	lncRNA	-1.088311792	0.040547917
SLC44A3	mRNA	-1.088809927	0.002510067
SLIT2	mRNA	-1.089621589	0.002161888
hsa_circ_0060802	circRNA	-1.090004209	2.56E-04
hsa_circ_0093670	circRNA	-1.090035253	0.034843649
hsa_circ_0067186	circRNA	-1.090290935	0.001053014
hsa_circ_0026502	circRNA	-1.090702377	5.62E-11

hsa_circ_0070776	circRNA	-1.091854343	6.24E-05
hsa_circ_0124695	circRNA	-1.092144561	0.006640786
hsa_circ_0080757	circRNA	-1.092219855	0.003970736
SEPT5	mRNA	-1.092810231	3.91E-04
MME	mRNA	-1.093067205	8.70E-05
hsa_circ_0009402	circRNA	-1.093577834	1.22E-06
BGIG9606_45850	mRNA	-1.093922539	0.045702171
FABP4	mRNA	-1.094493078	0.009152973
CTSF	mRNA	-1.094632421	1.49E-04
hsa_circ_0115298	circRNA	-1.094780284	0.01126407
PCDHGB1	mRNA	-1.095462782	0.016518873
ANGPTL4	mRNA	-1.096607093	0.003770202
hsa_circ_0012759	circRNA	-1.09739608	4.44E-10
hsa_circ_0073396	circRNA	-1.09763937	8.75E-05
hsa_circ_0102749	circRNA	-1.098565333	3.77E-07
PCOLCE	mRNA	-1.100299937	0.003994223
hsa_circ_0034031	circRNA	-1.100665999	7.02E-04
hsa_circ_0121459	circRNA	-1.101114864	0.00556922
LOC107984993	lncRNA	-1.101759123	0.033522495
hsa_circ_0073360	circRNA	-1.101833027	0.0173046
RTL5	mRNA	-1.103499214	0.010853037
KCNQ1	mRNA	-1.10443829	0.042478545
MAP2K6	mRNA	-1.104609902	0.001611773
CAVIN2	mRNA	-1.106429553	0.003068514
RASL10B	mRNA	-1.1077163	0.008650309

TNIK	mRNA	-1.109487475	2.16E-06
hsa_circ_0081918	circRNA	-1.109509226	1.59E-04
hsa_circ_0081934	circRNA	-1.109584898	8.95E-04
LINC01614	lncRNA	-1.10964212	0.013066529
hsa_circ_0086488	circRNA	-1.112500571	7.56E-04
IL33	mRNA	-1.113421182	0.012332966
hsa_circ_0042034	circRNA	-1.113568605	0.029294723
hsa_circ_0010020	circRNA	-1.113937359	2.31E-08
hsa_circ_0031911	circRNA	-1.114061315	0.034890377
hsa_circ_0075698	circRNA	-1.116950693	0.013598665
AMPH	mRNA	-1.118411061	0.004892736
BDH1	mRNA	-1.118505806	2.23E-05
LINC00641	lncRNA	-1.118976317	1.28E-09
DPF3	mRNA	-1.119462152	0.001974651
hsa_circ_0103279	circRNA	-1.120250842	1.74E-06
hsa_circ_0042039	circRNA	-1.120426403	0.013975789
LOXL4	mRNA	-1.122762409	0.004642327
hsa_circ_0026510	circRNA	-1.123201714	3.42E-13
EPB41L1	mRNA	-1.124541943	3.95E-05
hsa_circ_0088614	circRNA	-1.124766773	0.001503071
ATOH8	mRNA	-1.124983939	0.015408748
MAPK8IP2	mRNA	-1.126332178	0.003000726
HERC2P2	pseudo	-1.126831164	0.003303203
ADGRF5	mRNA	-1.128888082	1.60E-10
TENM3	mRNA	-1.128962791	0.012318438

SNAP25	mRNA	-1.12998281	0.044191528
hsa_circ_0035615	circRNA	-1.131841967	0.01245731
hsa_circ_0000784	circRNA	-1.133223538	0.026396501
hsa_circ_0026494	circRNA	-1.133445215	4.42E-04
MYZAP	mRNA	-1.134160124	0.014734086
PTGIS	mRNA	-1.135899941	1.16E-10
C17orf58	mRNA	-1.136324084	6.34E-04
hsa_circ_0070509	circRNA	-1.136980412	0.002523192
hsa_circ_0004526	circRNA	-1.137620973	0.00227482
NREP	mRNA	-1.140112457	7.33E-08
ZNF610	mRNA	-1.140394429	0.004154077
WDFY4	mRNA	-1.141099084	2.39E-04
LOC105372141	lncRNA	-1.141186206	0.024693566
hsa_circ_0087600	circRNA	-1.141720812	0.001751642
BGIG9606_49048	lncRNA	-1.143165363	3.72E-04
CADM4	mRNA	-1.144018553	2.59E-04
hsa_circ_0004742	circRNA	-1.144211821	0.002392478
hsa_circ_0034316	circRNA	-1.144927565	9.81E-07
ZNF641	mRNA	-1.146097722	0.034470492
STARD4-AS1	lncRNA	-1.14703503	2.91E-06
LOC101929710	lncRNA	-1.149785666	1.35E-04
hsa_circ_0040626	circRNA	-1.150313812	0.001671101
LOC401021	lncRNA	-1.150457308	5.46E-04
MMP28	mRNA	-1.152258022	0.003789113
hsa_circ_0091401	circRNA	-1.153763064	0.026324187

hsa_circ_0030862	circRNA	-1.15414574	0.002851506
hsa_circ_0098358	circRNA	-1.154214231	0.003457074
DNMBP-AS1	lncRNA	-1.156458352	1.62E-04
hsa_circ_0126452	circRNA	-1.158436022	0.042402524
CERS4	mRNA	-1.158809584	6.11E-12
SALL2	mRNA	-1.159141403	1.07E-08
NAV3	mRNA	-1.159954334	1.60E-08
hsa_circ_0010167	circRNA	-1.162611619	0.003603997
GDF1	mRNA	-1.164362511	0.004810528
BGIG9606_39478	lncRNA	-1.166018784	0.035909074
ACSF2	mRNA	-1.166509859	6.75E-08
hsa_circ_0057534	circRNA	-1.168562434	0.008534426
hsa_circ_0003173	circRNA	-1.168909842	0.005601453
FRMD5	mRNA	-1.169661135	6.18E-05
hsa_circ_0133100	circRNA	-1.169806466	2.64E-04
BMPER	mRNA	-1.169898142	0.002250206
NR6A1	mRNA	-1.170870191	1.96E-05
BASP1	mRNA	-1.172189038	1.83E-07
hsa_circ_0122919	circRNA	-1.175635293	0.008913798
PLSCR4	mRNA	-1.177243159	0.00106445
PHYHD1	mRNA	-1.178908389	0.034043983
LOC102724951	mRNA	-1.180218704	0.031316038
HAAO	mRNA	-1.182214778	0.009825601
THSD7A	mRNA	-1.183851024	3.90E-04
ABCA6	mRNA	-1.184118127	0.004921482

SV2C	mRNA	-1.189682678	0.001644857
hsa_circ_0088482	circRNA	-1.191798197	0.008899928
ZNF204P	pseudo	-1.192207217	0.009882797
hsa_circ_0015758	circRNA	-1.192326824	2.12E-06
DNM1	mRNA	-1.192530435	0.005290707
hsa_circ_0055927	circRNA	-1.193191457	1.70E-10
DKK3	mRNA	-1.194683701	1.11E-04
hsa_circ_0083940	circRNA	-1.19779612	7.44E-08
PLAGL1	mRNA	-1.198723657	0.002875642
FHL2	mRNA	-1.199858982	1.72E-11
hsa_circ_0073390	circRNA	-1.200056208	7.30E-04
hsa_circ_0130056	circRNA	-1.201095469	0.001491688
hsa_circ_0081929	circRNA	-1.201687073	9.11E-09
LOC107985371	lncRNA	-1.201898532	0.045883622
hsa_circ_0116397	circRNA	-1.20211626	0.01389951
hsa_circ_0010092	circRNA	-1.204107531	2.06E-05
RNF152	mRNA	-1.204489991	3.68E-06
hsa_circ_0137166	circRNA	-1.204656493	0.002251795
IER3-AS1	lncRNA	-1.206200422	0.001360082
SLC16A5	mRNA	-1.207800643	2.28E-09
hsa_circ_0133102	circRNA	-1.208103502	0.001213183
LOC102724861	lncRNA	-1.210329016	0.004739501
NR1H3	mRNA	-1.211269943	2.64E-05
AR	mRNA	-1.214935422	5.37E-04
TMED10P1	pseudo	-1.215797462	0.013721788

NHLRC4	mRNA	-1.217867207	0.001885546
LDLRAD4-AS1	lncRNA	-1.218083649	0.003704866
CAMK2N1	mRNA	-1.218447482	0.001039851
hsa_circ_0026525	circRNA	-1.221242862	1.22E-08
hsa_circ_0006053	circRNA	-1.22196171	0.001128955
hsa_circ_0073397	circRNA	-1.22262644	0.007149407
hsa_circ_0114631	circRNA	-1.222792526	0.001738762
LOC102724397	lncRNA	-1.224035833	2.54E-05
SSSCA1-AS1	lncRNA	-1.224992805	0.00415635
hsa_circ_0057536	circRNA	-1.225617368	0.008913798
ACOX2	mRNA	-1.226082434	4.92E-06
hsa_circ_0122712	circRNA	-1.226561467	3.37E-05
IL11RA	mRNA	-1.227879663	6.33E-05
PLOD2	mRNA	-1.2291363	2.85E-15
hsa_circ_0007901	circRNA	-1.229702147	0.012925034
HOXD3	mRNA	-1.231231056	0.004236388
hsa_circ_0079445	circRNA	-1.231320723	0.047934229
LINC00987	lncRNA	-1.233052179	0.036402101
HOXA10	mRNA	-1.234480156	1.17E-10
LOC102723665	lncRNA	-1.235336274	0.036678157
hsa_circ_0116524	circRNA	-1.23544124	1.36E-04
hsa_circ_0088481	circRNA	-1.235643594	0.010462737
DCAF12L2	mRNA	-1.241525446	0.028670457
BCL11A	mRNA	-1.246032681	0.032688761
hsa_circ_0044589	circRNA	-1.246769515	1.14E-05

TNFSF10	mRNA	-1.247598012	2.87E-04
HYI	mRNA	-1.248968612	5.47E-04
hsa_circ_0042046	circRNA	-1.249922068	0.02657136
hsa_circ_0034318	circRNA	-1.251533871	6.66E-08
BGIG9606_51212	lncRNA	-1.252462855	0.00145404
APLN	mRNA	-1.252525123	0.017115223
FAXDC2	mRNA	-1.253228586	1.35E-05
ALDH6A1	mRNA	-1.254389899	6.76E-04
MYH10	mRNA	-1.25439477	0.002128313
RPSAP52	pseudo	-1.255044025	3.30E-07
hsa_circ_0013444	circRNA	-1.25659235	1.14E-07
NANOS3	mRNA	-1.257016747	0.00737943
hsa_circ_0008417	circRNA	-1.258131765	7.29E-08
hsa_circ_0073389	circRNA	-1.258292855	3.10E-04
hsa_circ_0055925	circRNA	-1.258342974	1.72E-09
SORT1	mRNA	-1.260822696	1.73E-11
hsa_circ_0062273	circRNA	-1.261594942	0.040734076
PAIP2B	mRNA	-1.264091948	1.75E-04
hsa_circ_0122709	circRNA	-1.265517382	0.001898004
NALCN	mRNA	-1.266288394	3.00E-04
SAMD12	mRNA	-1.266883336	0.001517688
hsa_circ_0118413	circRNA	-1.267407314	4.07E-07
STAT1	mRNA	-1.267990782	2.85E-10
hsa_circ_0008383	circRNA	-1.271291552	3.21E-04
NDRG2	mRNA	-1.273402199	0.002287099

hsa_circ_0003324	circRNA	-1.274991352	6.45E-05
hsa_circ_0050674	circRNA	-1.27506555	1.24E-04
RPS6KA5	mRNA	-1.275819389	5.79E-06
hsa_circ_0107814	circRNA	-1.277932496	0.032865795
hsa_circ_0039468	circRNA	-1.278846018	0.025925407
BGIG9606_36247	lncRNA	-1.280510208	0.028152426
PLEKHB1	mRNA	-1.281215307	3.81E-04
DPY19L2	mRNA	-1.283090035	0.004246175
PPP1R3C	mRNA	-1.283228712	0.002747159
hsa_circ_0049043	circRNA	-1.28464051	2.84E-06
hsa_circ_0021959	circRNA	-1.286037043	0.001271691
hsa_circ_0108146	circRNA	-1.287629472	0.007207706
hsa_circ_0091393	circRNA	-1.287670324	1.91E-05
GSDMC	mRNA	-1.288828645	0.005151475
hsa_circ_0107829	circRNA	-1.290615021	0.021027882
SCGB3A1	mRNA	-1.29287447	0.011672681
N4BP2L1	mRNA	-1.295231617	3.69E-05
ZNF704	mRNA	-1.29531462	8.50E-07
hsa_circ_0139056	circRNA	-1.295890897	0.003132356
hsa_circ_0080753	circRNA	-1.296568298	0.005325859
hsa_circ_0107813	circRNA	-1.297457803	0.039651275
hsa_circ_0021281	circRNA	-1.297883436	0.003032166
hsa_circ_0119102	circRNA	-1.299220759	4.15E-06
hsa_circ_0025654	circRNA	-1.302856248	0.015013211
hsa_circ_0134195	circRNA	-1.304331365	0.046422838

hsa_circ_0013438	circRNA	-1.304684564	0.01482385
hsa_circ_0048053	circRNA	-1.306152626	1.60E-04
MFAP2	mRNA	-1.307021989	7.94E-06
hsa_circ_0040621	circRNA	-1.309267527	5.54E-04
hsa_circ_0110387	circRNA	-1.311648003	1.45E-07
hsa_circ_0122212	circRNA	-1.311722231	7.60E-04
ANGPT2	mRNA	-1.3146801	0.008260595
PRRX1	mRNA	-1.316172402	0.012334526
EFNB3	mRNA	-1.318746228	0.029392664
hsa_circ_0110388	circRNA	-1.322457011	9.95E-08
SMPDL3B	mRNA	-1.322919932	0.002058453
LOC102724081	lncRNA	-1.324094559	4.77E-04
hsa_circ_0013441	circRNA	-1.327503373	1.77E-07
LMNTD2	mRNA	-1.330883403	0.00158213
hsa_circ_0010170	circRNA	-1.332052278	3.48E-04
105370045	mRNA	-1.334278798	0.020620262
hsa_circ_0105713	circRNA	-1.334554455	8.40E-04
APCDD1	mRNA	-1.335517341	0.003862935
hsa_circ_0110400	circRNA	-1.335822416	7.03E-05
PTTG1	mRNA	-1.335940894	0.02397727
C3orf86	mRNA	-1.336123093	0.035055121
hsa_circ_0130055	circRNA	-1.336948279	0.001763303
hsa_circ_0034531	circRNA	-1.33784465	0.016570356
KSR2	mRNA	-1.338332827	0.003645704
CDK18	mRNA	-1.338745487	0.045254572

SAT1	mRNA	-1.339471251	1.67E-10
hsa_circ_0069310	circRNA	-1.339998481	0.045440267
SGIP1	mRNA	-1.342738137	1.59E-06
SCUBE2	mRNA	-1.343056477	0.035104391
hsa_circ_0043136	circRNA	-1.343780137	2.90E-04
CEMP1	mRNA	-1.344075934	0.001753985
NHS	mRNA	-1.348296457	8.46E-04
BGIG9606_54927	mRNA	-1.349468898	0.005282249
hsa_circ_0107830	circRNA	-1.356377878	0.023628643
BGIG9606_45673	lncRNA	-1.357631983	0.026803928
C15orf54	lncRNA	-1.359025856	9.36E-09
ABCA9	mRNA	-1.366509471	0.00244785
ENPP2	mRNA	-1.366846282	7.19E-08
BEND5	mRNA	-1.366913232	0.003710802
hsa_circ_0013433	circRNA	-1.370328125	4.40E-09
ADAMTS9-AS2	lncRNA	-1.370936514	6.91E-04
SHF	mRNA	-1.373273863	8.81E-07
BGIG9606_51325	mRNA	-1.374872746	0.006905957
PLS3-AS1	lncRNA	-1.376285222	0.00595508
AMIGO1	mRNA	-1.379627941	1.49E-04
CCL2	mRNA	-1.380910249	0.023801502
CRHBP	mRNA	-1.38472926	0.00767211
EYS	mRNA	-1.385745053	2.76E-06
PTPRD-AS1	lncRNA	-1.387020849	0.010677434
FZD8	mRNA	-1.38737419	7.79E-06

BGIG9606_37053	lncRNA	-1.390314246	0.010976071
LINC00345	lncRNA	-1.390368471	2.48E-05
LOC102724322	lncRNA	-1.39153012	1.68E-06
LOC105377124	lncRNA	-1.395523593	0.009949483
hsa_circ_0134100	circRNA	-1.398709684	2.34E-04
THBS1	mRNA	-1.401454724	0.003179779
hsa_circ_0034486	circRNA	-1.402163225	0.046858166
C6orf132	mRNA	-1.406044458	0.014662171
hsa_circ_0034499	circRNA	-1.407263118	0.043090992
hsa_circ_0034476	circRNA	-1.407651693	0.03745765
LOC105373150	lncRNA	-1.407673583	0.014029096
THRB	mRNA	-1.409602424	2.16E-05
hsa_circ_0034489	circRNA	-1.410960052	0.040164139
hsa_circ_0034438	circRNA	-1.411364522	0.044374932
hsa_circ_0034477	circRNA	-1.411498555	0.038766012
RNF219-AS1	lncRNA	-1.414500466	0.019611075
ADSSL1	mRNA	-1.418140354	5.01E-06
hsa_circ_0102594	circRNA	-1.419538831	0.01389951
hsa_circ_0034506	circRNA	-1.420344135	0.045572613
GRB14	mRNA	-1.420392436	5.76E-10
hsa_circ_0105705	circRNA	-1.421362088	3.20E-05
CASKIN1	mRNA	-1.423685713	3.01E-06
CDH11	mRNA	-1.423868835	1.45E-06
hsa_circ_0075878	circRNA	-1.423929851	1.09E-06
LINC00886	lncRNA	-1.425385537	9.72E-04

hsa_circ_0031262	circRNA	-1.432043576	0.002295691
hsa_circ_0010171	circRNA	-1.434811125	0.004185228
SLC25A34-AS1	lncRNA	-1.435852505	0.001975831
ITM2C	mRNA	-1.436178439	3.51E-07
AJUBA	mRNA	-1.437708308	3.76E-12
hsa_circ_0034466	circRNA	-1.437777752	0.024464143
CXADR	mRNA	-1.439381534	6.74E-04
hsa_circ_0034468	circRNA	-1.440287939	0.043815
FBLN1	mRNA	-1.441046502	1.35E-04
hsa_circ_0048418	circRNA	-1.441157241	1.50E-05
SDC2	mRNA	-1.441650161	2.39E-04
hsa_circ_0034487	circRNA	-1.441910302	0.034298971
hsa_circ_0034478	circRNA	-1.444313171	0.048006277
hsa_circ_0085011	circRNA	-1.444700424	3.51E-06
hsa_circ_0010172	circRNA	-1.445442484	0.001893902
hsa_circ_0131683	circRNA	-1.446276483	7.14E-04
hsa_circ_0110394	circRNA	-1.448181848	6.56E-04
hsa_circ_0034504	circRNA	-1.449588929	0.03369194
NXPH3	mRNA	-1.451362848	0.003331906
NPAS1	mRNA	-1.452368774	0.028122249
ITGA7	mRNA	-1.453720226	0.013843359
FBLN5	mRNA	-1.455454484	1.29E-05
hsa_circ_0034455	circRNA	-1.455547574	0.027569847
hsa_circ_0034518	circRNA	-1.455669252	0.037670691
NNAT	mRNA	-1.455875231	1.86E-05

hsa_circ_0034513	circRNA	-1.45670935	0.046374403
hsa_circ_0034471	circRNA	-1.457460636	0.03314835
hsa_circ_0034429	circRNA	-1.459834136	0.044608706
LINC01354	lncRNA	-1.461870515	0.010154663
hsa_circ_0034522	circRNA	-1.462250171	0.045026768
hsa_circ_0034525	circRNA	-1.464932917	0.038389557
hsa_circ_0081933	circRNA	-1.465213434	3.60E-04
hsa_circ_0034507	circRNA	-1.46751686	0.006295721
LINC02407	lncRNA	-1.46837321	2.40E-05
hsa_circ_0034451	circRNA	-1.468887303	0.030950515
hsa_circ_0034500	circRNA	-1.46891689	0.027181388
hsa_circ_0034459	circRNA	-1.469579109	0.021441401
hsa_circ_0034491	circRNA	-1.470145939	0.049519698
MATN3	mRNA	-1.470950048	0.01077485
CTF1	mRNA	-1.470968686	9.83E-09
SYT16	mRNA	-1.472358647	0.00157032
FBXO2	mRNA	-1.472497438	9.73E-04
hsa_circ_0034450	circRNA	-1.472947597	0.019779583
hsa_circ_0034496	circRNA	-1.473135096	0.017083401
hsa_circ_0034457	circRNA	-1.473693233	0.019950776
hsa_circ_0034493	circRNA	-1.474509368	0.03508714
hsa_circ_0034511	circRNA	-1.475011658	0.046740573
hsa_circ_0034458	circRNA	-1.475715464	0.017205248
hsa_circ_0034452	circRNA	-1.476993806	0.020712312
MMP19	mRNA	-1.477227748	0.026841515

DCAF12L1	mRNA	-1.477588017	1.49E-05
hsa_circ_0034460	circRNA	-1.478671389	0.019982278
BGIG9606_47873	lncRNA	-1.478753372	0.048156252
GUCY1B1	mRNA	-1.481612833	2.32E-04
hsa_circ_0034539	circRNA	-1.481928574	0.006830087
BGIG9606_38067	mRNA	-1.482203305	0.03579455
hsa_circ_0034524	circRNA	-1.482640934	0.046844961
GADD45B	mRNA	-1.484993268	1.25E-06
hsa_circ_0110393	circRNA	-1.485251145	4.43E-05
hsa_circ_0034541	circRNA	-1.485413448	0.032207739
hsa_circ_0034534	circRNA	-1.486981414	0.025441716
hsa_circ_0034475	circRNA	-1.487008633	0.046844961
hsa_circ_0034433	circRNA	-1.487814549	0.032029771
hsa_circ_0034519	circRNA	-1.489420393	0.015013211
hsa_circ_0034453	circRNA	-1.491856617	0.019290435
CEP126	mRNA	-1.492597728	6.97E-10
NRG1	mRNA	-1.493230211	8.50E-04
LRCOL1	mRNA	-1.494291346	0.006975051
hsa_circ_0034424	circRNA	-1.495164929	0.035565357
GNG7	mRNA	-1.496662393	3.58E-07
hsa_circ_0034509	circRNA	-1.498220578	0.00139125
hsa_circ_0034498	circRNA	-1.498324986	0.036622497
PAK1	mRNA	-1.499503205	2.57E-09
LINC00622	lncRNA	-1.502391854	3.05E-06
hsa_circ_0034502	circRNA	-1.502557635	0.025793774

LOC105371934	lncRNA	-1.506925437	0.035808405
hsa_circ_0034435	circRNA	-1.507238093	0.024807331
CTSV	mRNA	-1.509822744	0.017618328
ANKRD18B	mRNA	-1.509904487	0.001302545
hsa_circ_0034449	circRNA	-1.510816366	0.020003806
BGIG9606_47872	lncRNA	-1.513577526	0.045254572
hsa_circ_0034420	circRNA	-1.515347403	0.020133152
hsa_circ_0034427	circRNA	-1.517828387	0.036444725
hsa_circ_0034456	circRNA	-1.518670283	0.005978068
ACSS3	mRNA	-1.51928277	0.006469895
FRAS1	mRNA	-1.520301479	0.001280235
RBP1	mRNA	-1.523439205	2.16E-12
hsa_circ_0034508	circRNA	-1.523781255	0.046873778
PLLP	mRNA	-1.524835724	2.78E-08
hsa_circ_0034474	circRNA	-1.525256417	0.025335973
KCNS1	mRNA	-1.525433085	8.48E-04
MPP2	mRNA	-1.526079464	0.0011395
IRF6	mRNA	-1.528085139	1.51E-04
DOCK2	mRNA	-1.528695706	0.004337452
hsa_circ_0034505	circRNA	-1.528929309	0.009584518
EPHX2	mRNA	-1.533091666	8.63E-06
hsa_circ_0025563	circRNA	-1.533157808	7.73E-07
CLDN10	mRNA	-1.533769591	0.018594778
FAM189A1	mRNA	-1.534043118	0.021786055
hsa_circ_0078047	circRNA	-1.535083547	0.001492593

SDC1	mRNA	-1.537311977	2.97E-10
CD38	mRNA	-1.541347014	3.00E-04
hsa_circ_0034442	circRNA	-1.541710178	0.023841572
hsa_circ_0034461	circRNA	-1.542551171	0.007239942
hsa_circ_0034436	circRNA	-1.542824188	0.02233544
ZNF365	mRNA	-1.546893304	0.01706378
hsa_circ_0034428	circRNA	-1.548906646	0.026902807
BGIG9606_48556	mRNA	-1.549786532	2.56E-04
hsa_circ_0034533	circRNA	-1.551502085	0.01126407
hsa_circ_0034454	circRNA	-1.552113834	0.019191424
SELENOP	mRNA	-1.553661154	2.88E-04
ENPP1	mRNA	-1.556261426	3.24E-04
ADAM11	mRNA	-1.55885857	3.36E-05
hsa_circ_0034537	circRNA	-1.558914588	0.028762611
hsa_circ_0034538	circRNA	-1.559256823	0.017171743
CTTNBP2	mRNA	-1.559822048	2.81E-04
hsa_circ_0006885	circRNA	-1.566401997	7.10E-06
CASP17P	pseudo	-1.571408088	2.73E-08
hsa_circ_0034434	circRNA	-1.572582205	0.024745146
FAM160A1	mRNA	-1.572754693	1.29E-06
hsa_circ_0034469	circRNA	-1.572909026	0.048783911
RAB11FIP1	mRNA	-1.574693823	0.041295162
hsa_circ_0034536	circRNA	-1.57638812	0.015797859
hsa_circ_0034443	circRNA	-1.578041983	0.039895567
BHLHE41	mRNA	-1.579476519	0.044983579

hsa_circ_0034485	circRNA	-1.580002803	0.040400383
SEPT5-GP1BB	lncRNA	-1.580561226	8.70E-04
DUSP15	mRNA	-1.583264342	4.88E-06
hsa_circ_0034431	circRNA	-1.585962521	0.014553601
hsa_circ_0034422	circRNA	-1.588493069	0.040301848
BGIG9606_55370	lncRNA	-1.590986302	0.003628988
hsa_circ_0034490	circRNA	-1.591223683	0.041768215
112268452	mRNA	-1.592851668	1.60E-04
PPP1R3B	mRNA	-1.59496658	2.04E-07
NAT8	mRNA	-1.5965514	0.008799099
hsa_circ_0136869	circRNA	-1.602782524	0.001170387
IL7R	mRNA	-1.605982661	0.002474204
SOX2	mRNA	-1.613831746	6.63E-04
hsa_circ_0067557	circRNA	-1.614071121	0.001986693
hsa_circ_0034426	circRNA	-1.620130055	0.003402305
hsa_circ_0039466	circRNA	-1.620256088	0.004648741
FAP	mRNA	-1.621396116	0.019542533
SH3BP1	mRNA	-1.631437427	2.21E-04
DAAM2	mRNA	-1.634320136	8.40E-08
hsa_circ_0079822	circRNA	-1.634711	2.60E-04
LOC105370094	lncRNA	-1.635419451	0.014312369
DCLK1	mRNA	-1.643467668	0.007355828
PTX3	mRNA	-1.644087523	0.026987966
SSTR1	mRNA	-1.644850452	0.00272915
CGN	mRNA	-1.649079486	0.00773321

IL31RA	mRNA	-1.653266228	0.00810597
FILIP1	mRNA	-1.656489468	2.31E-10
GAS1	mRNA	-1.65778023	0.001982844
COL14A1	mRNA	-1.660185417	0.038662619
BGIG9606_50370	lncRNA	-1.666465351	0.006641705
AATK	mRNA	-1.669496647	2.66E-05
CXCL11	mRNA	-1.672871422	2.46E-04
ANOS1	mRNA	-1.677704568	4.86E-04
IRS2	mRNA	-1.678905896	3.15E-09
SORBS1	mRNA	-1.679933332	0.029859852
BGIG9606_39026	lncRNA	-1.682361171	0.019649265
HTRA4	mRNA	-1.688335095	0.026939597
hsa_circ_0034419	circRNA	-1.695196063	0.001857811
IRS1	mRNA	-1.697389578	9.96E-04
BAMBI	mRNA	-1.702774088	6.24E-10
PNPLA7	mRNA	-1.703844522	3.86E-07
CDKL2	mRNA	-1.704609793	6.88E-07
IER3	mRNA	-1.707997257	2.39E-07
hsa_circ_0051196	circRNA	-1.71375159	1.71E-15
EDN1	mRNA	-1.715413452	0.028064113
4-Mar	mRNA	-1.717364322	0.001719372
hsa_circ_0034542	circRNA	-1.721399794	6.99E-05
RAB26	mRNA	-1.724429298	0.001598482
hsa_circ_0007279	circRNA	-1.726728894	0.023387812
ANKRD1	mRNA	-1.728583885	0.013097233

BEX2	mRNA	-1.732616566	9.44E-08
INSYN2B	mRNA	-1.734012608	9.45E-10
GCNT1	mRNA	-1.736536616	1.05E-04
GPR146	mRNA	-1.739686752	1.83E-11
hsa_circ_0051185	circRNA	-1.740191694	0.006653129
hsa_circ_0107820	circRNA	-1.744337781	0.036371598
TNFSF15	mRNA	-1.744625058	2.96E-04
hsa_circ_0055922	circRNA	-1.747188875	9.02E-05
ELFN2	mRNA	-1.748162257	0.040785252
GDF3	mRNA	-1.750393496	0.001993466
LOC101927811	lncRNA	-1.751070734	0.002256086
hsa_circ_0066470	circRNA	-1.753886537	0.031814684
MT1E	mRNA	-1.759481033	5.21E-07
RND1	mRNA	-1.761746573	8.95E-07
NUPR1	mRNA	-1.766464425	5.85E-05
MAPK13	mRNA	-1.770940668	2.15E-10
LOC105373381	mRNA	-1.775830966	0.003438782
DCN	mRNA	-1.779140326	0.007794205
RGN	mRNA	-1.78132721	0.015662256
hsa_circ_0003271	circRNA	-1.789234101	5.76E-23
LOC100505501	lncRNA	-1.790230637	5.51E-13
PDE3A	mRNA	-1.794871463	6.13E-05
RAP1GAP	mRNA	-1.796743307	0.004912476
UNC5B	mRNA	-1.803502279	6.81E-06
TRPM3	mRNA	-1.806797996	0.014029096

ADHFE1	mRNA	-1.818461125	2.82E-06
LINC02188	lncRNA	-1.825144301	6.09E-05
MOV10L1	mRNA	-1.827489835	0.005967476
SPOCK3	mRNA	-1.829470501	0.007950494
hsa_circ_0039467	circRNA	-1.831475728	4.61E-09
ENTPD8	mRNA	-1.832849817	0.00230598
KLRG1	mRNA	-1.842123588	4.11E-05
hsa_circ_0051182	circRNA	-1.849032002	4.24E-05
hsa_circ_0091746	circRNA	-1.849068385	2.33E-13
GYG2	mRNA	-1.856841049	0.006369056
AXL	mRNA	-1.883074343	9.51E-14
hsa_circ_0039465	circRNA	-1.903227925	4.27E-08
hsa_circ_0127834	circRNA	-1.912739764	0.015321962
MMP7	mRNA	-1.913537354	0.002093121
hsa_circ_0019089	circRNA	-1.914191333	0.025335973
hsa_circ_0051189	circRNA	-1.915048048	5.93E-10
CYP39A1	mRNA	-1.921625073	9.68E-04
hsa_circ_0091742	circRNA	-1.924207364	8.48E-08
CCDC80	mRNA	-1.925171225	9.21E-06
hsa_circ_0051200	circRNA	-1.931313898	1.42E-12
CA4	mRNA	-1.940798723	0.007862533
hsa_circ_0051165	circRNA	-1.94537455	4.34E-21
SOX5	mRNA	-1.949487847	2.00E-08
COL11A1	mRNA	-1.949704586	0.00443253
SEMA3G	mRNA	-1.953224157	6.13E-27

hsa_circ_0051190	circRNA	-1.958957625	1.92E-07
COL8A1	mRNA	-1.961341499	0.006377407
hsa_circ_0125602	circRNA	-1.965790523	2.66E-05
BGIG9606_52854	lncRNA	-1.977561307	0.038401489
hsa_circ_0039471	circRNA	-1.979168649	1.50E-08
MIR217HG	lncRNA	-1.983429976	1.06E-04
GDF7	mRNA	-1.987061935	0.006415827
LOC112267886	mRNA	-1.988684268	0.036111656
LINC01013	lncRNA	-1.990625337	0.014793721
hsa_circ_0091741	circRNA	-1.991204739	4.29E-15
PDGFC	mRNA	-1.991563234	2.69E-08
hsa_circ_0084717	circRNA	-1.998488342	0.011673098
hsa_circ_0025572	circRNA	-2.004987584	0.001690223
LOC105373989	mRNA	-2.005358136	0.008500912
DBP	mRNA	-2.012854821	4.47E-12
TMEM178A	mRNA	-2.013652586	0.017401456
LOC107985555	mRNA	-2.014447644	0.004020862
LOC102724687	lncRNA	-2.015734803	2.14E-04
FAM13C	mRNA	-2.017687388	0.029301336
AQP1	mRNA	-2.026239533	9.24E-10
SLC44A5	mRNA	-2.026449536	4.98E-07
BGIG9606_39257	lncRNA	-2.034544791	0.038455034
BGN	mRNA	-2.034573912	2.82E-15
hsa_circ_0103399	circRNA	-2.064912801	1.18E-04
BGIG9606_40202	lncRNA	-2.067025213	0.030489287

MYLIP	mRNA	-2.072148448	1.45E-15
LNX1	mRNA	-2.083606497	2.19E-06
BGIG9606_50661	lncRNA	-2.085637262	0.002488902
BGIG9606_42818	lncRNA	-2.088922941	1.48E-04
XKRX	mRNA	-2.092461433	0.010215442
hsa_circ_0039462	circRNA	-2.100281004	1.72E-07
CLEC10A	mRNA	-2.100839254	0.012726739
hsa_circ_0039461	circRNA	-2.103253126	1.00E-04
ANKRD18A	mRNA	-2.105973855	9.34E-04
LOC112268447	lncRNA	-2.130725807	3.59E-04
LOC101929028	lncRNA	-2.135271721	8.61E-04
BTK	mRNA	-2.14313788	3.70E-09
SMIM1	mRNA	-2.145096717	3.02E-05
LINC01235	lncRNA	-2.152274741	2.01E-04
LOC105376159	lncRNA	-2.165864923	0.013387339
VCAM1	mRNA	-2.16878104	3.28E-09
CYP2S1	mRNA	-2.169843154	0.002146336
hsa_circ_0039463	circRNA	-2.172803742	2.87E-08
MEIS1-AS2	lncRNA	-2.177619087	0.018212148
GPRIN3	mRNA	-2.179843736	1.00E-10
hsa_circ_0125601	circRNA	-2.183619252	1.97E-06
TENT5B	mRNA	-2.205454502	2.59E-04
MGARP	mRNA	-2.20735617	1.40E-04
ACE	mRNA	-2.216231722	1.05E-24
UGT2A1	mRNA	-2.218805195	0.019650265

HERC5	mRNA	-2.220795189	2.34E-22
OGDHL	mRNA	-2.225472104	5.22E-06
CXCL6	mRNA	-2.228295391	0.008204337
ABCG1	mRNA	-2.229412687	1.51E-06
BRWD1-AS1	lncRNA	-2.230544772	0.011065263
ADAD2	mRNA	-2.236434687	0.001247978
TGFB2	mRNA	-2.236926271	0.001511069
BGIG9606_37598	lncRNA	-2.238873736	0.014734086
DNAJC5G	mRNA	-2.241539579	0.01420731
hsa_circ_0039460	circRNA	-2.249046642	5.97E-09
INHBA	mRNA	-2.256152705	4.06E-19
hsa_circ_0091743	circRNA	-2.277936321	1.47E-07
GUCY1A1	mRNA	-2.28857314	1.05E-09
ADAMTS12	mRNA	-2.298435674	9.21E-06
LYPD1	mRNA	-2.302458199	0.038109026
MT2A	mRNA	-2.310104815	2.35E-12
AQP5	mRNA	-2.316600305	1.76E-05
PGAM1P5	pseudo	-2.316897168	0.04045609
BGIG9606_42940	mRNA	-2.325019749	0.040687383
TNFSF18	mRNA	-2.328840043	8.27E-10
INHBB	mRNA	-2.329167697	0.001488076
LRRC4	mRNA	-2.333174976	5.75E-11
LOC105375221	lncRNA	-2.33589861	0.00934573
hsa_circ_0051195	circRNA	-2.340528488	4.08E-19
SULF1	mRNA	-2.346625054	1.86E-11

MT1L	pseudo	-2.346818667	2.44E-06
OLFML1	mRNA	-2.348478075	0.005043221
RSPH10B2	mRNA	-2.350175358	0.040431306
hsa_circ_0039459	circRNA	-2.359723362	2.35E-10
LOC100506851	lncRNA	-2.367773653	0.002035517
KLF15	mRNA	-2.380961682	7.63E-08
PLXNA4	mRNA	-2.38127938	0.001529997
LOC100506258	lncRNA	-2.381524091	7.70E-13
FMO3	mRNA	-2.407128756	0.037296738
PPFIBP2	mRNA	-2.418888535	3.77E-17
hsa_circ_0085019	circRNA	-2.422251659	0.045327291
NOTCH3	mRNA	-2.432825758	4.30E-11
ANKRD2	mRNA	-2.432922175	0.011216986
BGIG9606_46646	lncRNA	-2.451596459	0.00145404
SERPINB2	mRNA	-2.453089296	5.52E-06
RSPH10B	mRNA	-2.46494055	2.48E-05
ELMOD1	mRNA	-2.467062334	3.53E-12
TLL1	mRNA	-2.469345952	0.005013335
PKNOX2	mRNA	-2.476278909	1.71E-09
RRN3P3	pseudo	-2.485632716	0.017618328
CPA3	mRNA	-2.498106152	2.37E-05
hsa_circ_0084719	circRNA	-2.500887341	2.31E-07
EPHB6	mRNA	-2.51053817	1.80E-04
DSC2	mRNA	-2.514350279	1.66E-05
LOC102724122	lncRNA	-2.515158791	0.007534247

PTH1R	mRNA	-2.527843726	0.01066793
SPDYE8P	pseudo	-2.528808681	0.015468118
APCDD1L-DT	lncRNA	-2.532181939	1.54E-04
CIART	mRNA	-2.538789923	3.89E-08
CLDN11	mRNA	-2.554801974	4.52E-09
TMEFF2	mRNA	-2.566828742	0.005815936
RHBDL3	mRNA	-2.56717906	0.032985618
TOX	mRNA	-2.582752484	4.82E-49
LRRC17	mRNA	-2.612677083	1.53E-38
BGIG9606_42073	lncRNA	-2.645977118	0.043886527
FAT3	mRNA	-2.651304834	0.014028003
PDE1A	mRNA	-2.707289687	3.10E-04
COL12A1	mRNA	-2.707312706	9.77E-04
HCP5	lncRNA	-2.719553462	0.011138188
SEPT4	mRNA	-2.734054791	0.014386906
PCSK1	mRNA	-2.752630387	1.28E-07
WIPF3	mRNA	-2.759369106	0.005860427
FST	mRNA	-2.764088166	0.006526503
NEGR1	mRNA	-2.765288465	1.32E-07
NPR3	mRNA	-2.766865407	3.72E-05
ADAMTS15	mRNA	-2.788634803	2.22E-04
BGIG9606_54773	mRNA	-2.793310065	0.002367281
LOC105373454	lncRNA	-2.808762521	0.002477977
PRSS35	mRNA	-2.84606675	0.048224609
SERPIND1	mRNA	-2.865381561	5.93E-06

PTPN22	mRNA	-2.88240385	5.62E-04
ANK3	mRNA	-2.897291465	1.09E-09
ADTRP	mRNA	-2.920863938	7.48E-06
PARD6B	mRNA	-2.933387351	0.015799752
LINC01592	lncRNA	-2.937023634	0.013778934
LOC101928358	lncRNA	-2.944631996	0.018239069
GPM6A	mRNA	-2.972532316	4.93E-08
HMCN2	mRNA	-2.998974283	0.002764756
KCNJ6	mRNA	-3.013736318	0.017474584
LOC105376196	lncRNA	-3.032573696	4.27E-04
XXYLT1-AS2	lncRNA	-3.03875346	8.11E-08
LOC105372814	lncRNA	-3.073045129	7.32E-07
SLC7A14	mRNA	-3.101592682	0.001117808
BCL2A1	mRNA	-3.11612178	0.026946583
ABCA1	mRNA	-3.157034972	4.12E-04
hsa_circ_0042033	circRNA	-3.219421554	0.040164927
STAT4	mRNA	-3.249632835	5.55E-09
LOC101927057	lncRNA	-3.251191363	1.47E-06
BGIG9606_43129	lncRNA	-3.284716826	0.030331333
hsa_circ_0066056	circRNA	-3.298999317	0.030365914
RGS4	mRNA	-3.312961624	2.11E-13
CYSLTR2	mRNA	-3.335759754	0.009342162
ANGPTL5	mRNA	-3.33985893	0.042868647
LOC101928202	lncRNA	-3.346317938	0.00114172
BGIG9606_46936	lncRNA	-3.375842214	0.001708529

BGIG9606_54793	lncRNA	-3.392214973	0.006634107
PNCK	mRNA	-3.423269965	0.031418248
CDH6	mRNA	-3.434680527	2.48E-04
KRT80	mRNA	-3.444150849	2.13E-13
PLIN5	mRNA	-3.488491665	3.30E-07
DKK1	mRNA	-3.541727013	2.83E-08
PIFO	mRNA	-3.546602833	4.13E-05
IRS4	mRNA	-3.592898015	0.009568072
LINGO2	mRNA	-3.664742854	3.97E-06
KCNH7	mRNA	-3.690681749	1.66E-05
CD207	mRNA	-3.72551664	0.045067107
MT1A	mRNA	-3.946744595	6.49E-05
LOC102724880	lncRNA	-3.947429236	0.049022729
GJA5	mRNA	-3.982066239	1.72E-05
BGIG9606_68341	mRNA	-4.101734863	1.97E-04
LOC107984113	lncRNA	-4.158288167	0.037606746
DAW1	mRNA	-4.164228402	1.46E-12
SCNN1B	mRNA	-4.330702925	6.76E-05
BGIG9606_43492	mRNA	-4.376755036	2.86E-08
BGIG9606_51068	lncRNA	-4.40771238	0.041483545
GPC4	mRNA	-4.4886118	0.006083201
FBP2	mRNA	-4.495145362	0.00272915
BGIG9606_54519	lncRNA	-4.642753894	0.041809578
GJA4	mRNA	-4.649628788	5.46E-11
CNTN6	mRNA	-4.65502862	0.043946723

MYEOV	mRNA	-4.718812444	9.13E-05
BGIG9606_54103	lncRNA	-4.988095643	0.048442598
PRND	mRNA	-5.013233123	4.40E-15
LOC107986566	lncRNA	-5.182812867	4.18E-04
FSTL5	mRNA	-5.262562982	0.008155414
ZDHHC8P1	pseudo	-5.427632978	1.13E-08
KRT7	mRNA	-5.509809907	8.42E-08
VIT	mRNA	-5.597915337	6.87E-05
BGIG9606_52767	lncRNA	-5.654217504	0.001022151
ITPRID1	mRNA	-5.727542638	0.007401488
LOC105376603	lncRNA	-5.799397352	2.13E-05
LOC100507642	lncRNA	-5.937358719	0.004656267
SCNN1G	mRNA	-6.027882782	8.16E-05
hsa_circ_0127636	circRNA	-6.223360307	0.03988616
hsa_circ_0015684	circRNA	-6.226277579	0.039945557
hsa_circ_0098223	circRNA	-6.240561368	0.036372042
hsa_circ_0120231	circRNA	-6.246933091	0.036292609
hsa_circ_0003633	circRNA	-6.256042991	0.033076375
hsa_circ_0081823	circRNA	-6.274780686	0.029639845
hsa_circ_0079442	circRNA	-6.281238984	0.028866526
hsa_circ_0137311	circRNA	-6.284511549	0.028463571
hsa_circ_0000087	circRNA	-6.291443699	0.027373375
hsa_circ_0125606	circRNA	-6.292203115	0.026485773
hsa_circ_0133123	circRNA	-6.294843906	0.026172486
hsa_circ_0120633	circRNA	-6.300784198	0.025770124

hsa_circ_0066299	circRNA	-6.311179969	0.024011174
hsa_circ_0040395	circRNA	-6.335716854	0.020670157
hsa_circ_0022267	circRNA	-6.33650927	0.019832587
hsa_circ_0066064	circRNA	-6.337217649	0.020050771
hsa_circ_0131562	circRNA	-6.337658873	0.02046482
hsa_circ_0115927	circRNA	-6.358870792	0.018783401
hsa_circ_0110383	circRNA	-6.368525704	0.016570356
hsa_circ_0089967	circRNA	-6.369264789	0.016453947
hsa_circ_0004391	circRNA	-6.369419778	0.016453947
hsa_circ_0005769	circRNA	-6.369419778	0.016453947
hsa_circ_0039645	circRNA	-6.378342522	0.0146839
hsa_circ_0138993	circRNA	-6.382062283	0.014359458
hsa_circ_0115452	circRNA	-6.382618407	0.014800208
hsa_circ_0025231	circRNA	-6.383609565	0.014617338
hsa_circ_0063528	circRNA	-6.403411188	0.012249447
hsa_circ_0004325	circRNA	-6.410179971	0.041070799
hsa_circ_0098645	circRNA	-6.411574924	0.011844723
hsa_circ_0010024	circRNA	-6.411876172	0.011953239
hsa_circ_0098215	circRNA	-6.415611908	0.011195558
hsa_circ_0061823	circRNA	-6.416640908	0.040228232
hsa_circ_0090098	circRNA	-6.420370704	0.011198979
hsa_circ_0059497	circRNA	-6.428659762	0.037292408
hsa_circ_0127253	circRNA	-6.431660236	0.009822201
hsa_circ_0072041	circRNA	-6.442552776	0.009160542
hsa_circ_0125607	circRNA	-6.443105262	0.008949684

hsa_circ_0092498	circRNA	-6.444895877	0.034958343
hsa_circ_0081207	circRNA	-6.454861049	0.032029771
hsa_circ_0035049	circRNA	-6.470719771	0.007173399
hsa_circ_0095280	circRNA	-6.481378494	0.029023172
hsa_circ_0081113	circRNA	-6.506044758	0.005317566
hsa_circ_0059519	circRNA	-6.507080513	0.023853076
hsa_circ_0089563	circRNA	-6.52859676	0.004541696
hsa_circ_0069308	circRNA	-6.53459587	0.019237932
CPA4	mRNA	-6.542345534	1.65E-06
hsa_circ_0100407	circRNA	-6.54709989	0.018389717
hsa_circ_0113440	circRNA	-6.57980142	0.002737533
hsa_circ_0138444	circRNA	-6.598713157	0.002462866
hsa_circ_0006874	circRNA	-6.599898871	0.00247468
hsa_circ_0034237	circRNA	-6.622584813	0.001751817
hsa_circ_0087803	circRNA	-6.628353719	0.010201429
hsa_circ_0081116	circRNA	-6.669265495	0.001111378
hsa_circ_0099254	circRNA	-6.688375198	9.04E-04
hsa_circ_0113446	circRNA	-6.691305475	9.02E-04
hsa_circ_0136474	circRNA	-6.716491759	6.71E-04
hsa_circ_0001368	circRNA	-6.719157687	5.59E-04
SEMA5B	mRNA	-6.720343004	3.97E-06
hsa_circ_0090099	circRNA	-6.764505104	4.14E-04
hsa_circ_0049059	circRNA	-6.823281858	1.79E-04
hsa_circ_0072759	circRNA	-6.849620962	1.50E-04
hsa_circ_0084722	circRNA	-6.863182348	1.56E-04

hsa_circ_0008864	circRNA	-6.877755141	9.34E-05
hsa_circ_0051179	circRNA	-6.903701089	5.91E-05
hsa_circ_0137612	circRNA	-6.944024035	3.94E-05
hsa_circ_0026362	circRNA	-6.958521418	7.97E-05
hsa_circ_0084720	circRNA	-7.02637242	1.33E-05
hsa_circ_0109177	circRNA	-7.107820237	4.15E-06
hsa_circ_0001725	circRNA	-7.11696547	2.76E-06
hsa_circ_0026369	circRNA	-7.218841095	2.26E-06
hsa_circ_0103603	circRNA	-7.261601534	7.14E-07
hsa_circ_0013450	circRNA	-7.343776111	1.27E-07
CCDC190	mRNA	-7.399882895	2.39E-08
hsa_circ_0084721	circRNA	-7.405645122	8.64E-08
hsa_circ_0087818	circRNA	-7.529585044	2.94E-08
hsa_circ_0026368	circRNA	-7.529874285	5.93E-08
hsa_circ_0026360	circRNA	-7.779253626	3.83E-06
